# Streptocyanine as an activation mode of amine catalysis for the conversion of pyridine rings to benzene rings†

**DOI:** 10.1039/d2sc06225a

**Published:** 2022-12-21

**Authors:** Tatsuya Morofuji, Shota Nagai, Airi Watanabe, Kota Inagawa, Naokazu Kano

**Affiliations:** a Department of Chemistry, Faculty of Science, Gakushuin University 1-5-1 Mejiro Toshima-ku Tokyo 171-8588 Japan tatsuya.morofuji@gakushuin.ac.jp moro.chemistry@gmail.com naokazu.kano@gakushuin.ac.jp

## Abstract

Amine catalysts have emerged as an invaluable tool in organic synthesis. Iminium, enamine, and enamine radical cation species are representative activation modes of amine catalysis. However, the development of new amine catalysis activation modes that enable novel synthetic strategies remains highly desirable. Herein, we report streptocyanine as a new amine catalysis activation mode, which enables the skeletal editing of pyridine rings to benzene rings. *N*-Arylation of pyridines bearing an alkenyl substituent at the 3-position generates the corresponding *N*-arylpyridiniums. The resulting pyridinum reacts with a catalytic amount of piperidine to afford a streptocyanine intermediate. Catalytically generated streptocyanine forms a benzene ring *via* a ring-closing reaction, thereby releasing the amine catalyst. Consequently, the alkene moiety in the starting pyridines is incorporated into the benzene ring of the products. Pyridiniums bearing various alkene moieties were efficiently converted to formyl-substituted benzene derivatives. Mechanistic studies support the postulation that the present catalytic process was intermediated by streptocyanine. In this reaction system, streptocyanine could be regarded as a new activation mode of amine catalysis.

## Abstract 2

We report conjugated methine compounds generated from *N*-arylpyridiniums and amines; streptocyanines can be used as a new activation mode for amine catalysis and applied to the conversion of pyridine rings to benzene rings.

## Graphical abstract

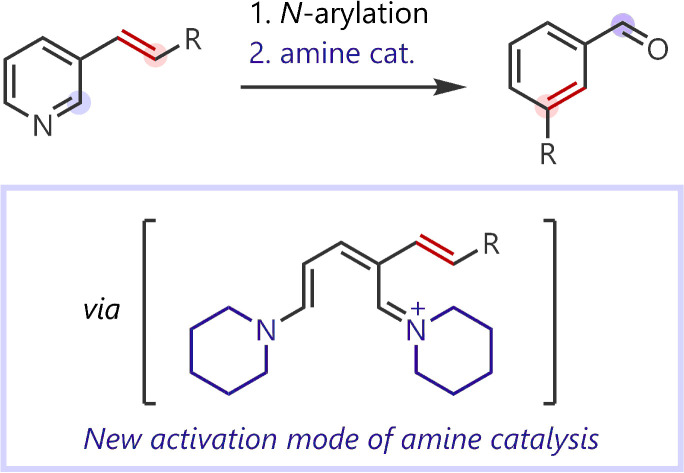

## Introduction

**Fig. 1:**
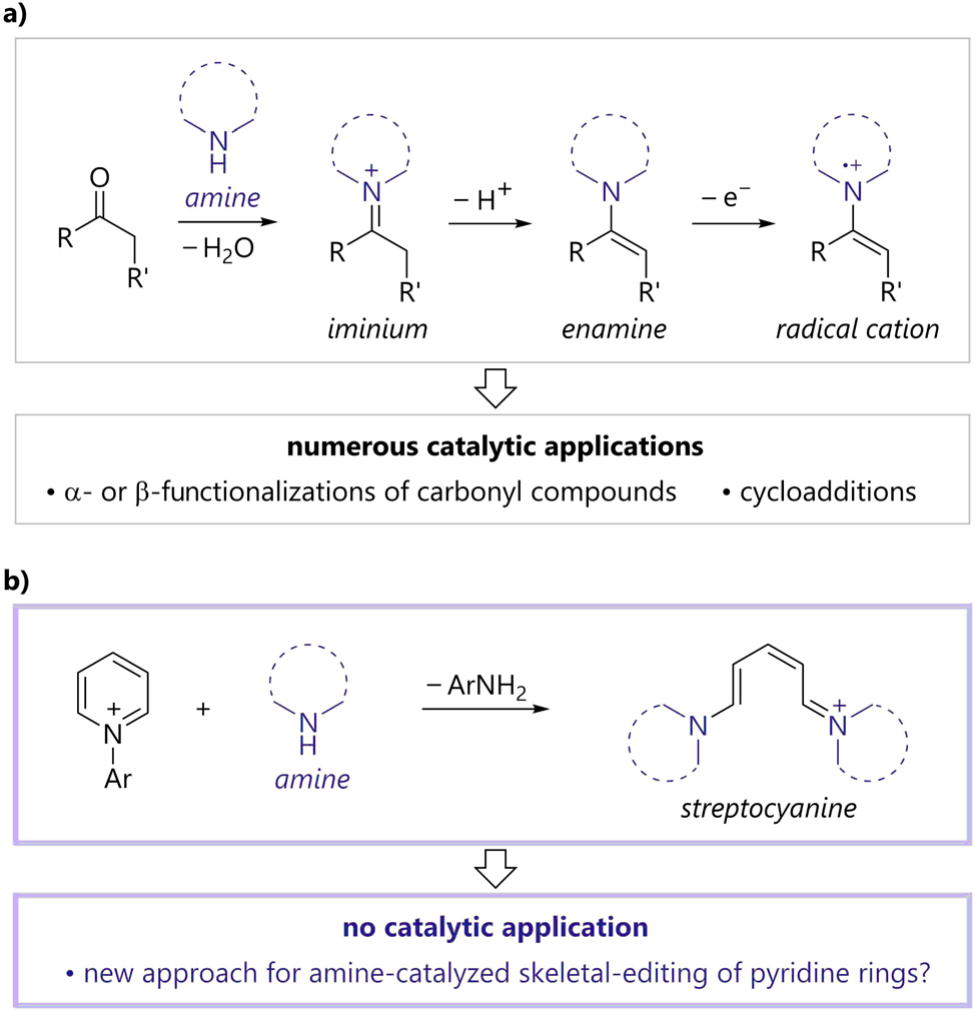
Reaction of secondary amines (a) with carbonyl compounds, (b) with *N*-arylpyridiniums.

Amine catalysts are among the most commonly utilized organocatalysts and have become an invaluable organic synthetic tool, as they enable unique and sustainable syntheses of various medicinal compounds and natural products.^1^ A representative application of amine catalysts is the activation of carbonyl compounds (Fig. 1a). Amines react with carbonyl compounds to afford iminiums, which facilitate conjugate additions, cycloadditions, and Friedel–Crafts alkylations.^1*a*,1*c*,1*f*^ Enamines generated by the deprotonation of iminiums are key intermediates in amine-catalyzed aldol reactions, α-alkylations, α-aminations, and α-oxygenations of carbonyl compounds.^1*b*,1*c*,1*f*^ Enamine radical cations, generated *via* single-electron oxidation, can react with various nucleophiles at the α-position of the original carbonyl group,^2^ thereby expanding the potential of amine catalysis as a new activation mode.^1*c*,1*e*^ Currently, tertiary amines are used in nucleophilic^3^ or hydrogen atom transfer catalysis.^4^ The development of new activation modes of amine catalysis remains highly desirable as it would contribute to the advancement of various fields of chemistry, such as drug discovery, agrochemistry, and material science.

In contrast to carbonyl compounds, aromatic rings generally cannot react with amines due to their thermodynamic stability imparted by their aromaticity. An exception is the reaction of *N*-arylpyridiniums and secondary amines, which is known to afford the corresponding conjugated methine compounds, streptocyanines (Fig. 1b).^5^ Streptocyanines are generally applicable in dye chemistry.^6^ In addition, several stoichiometric synthetic methods for generating and converting streptocyanines have been developed.^7^ However, to the best of our knowledge, strategies wherein streptocyanines participate in catalytic processes as an active intermediate have not been developed.

Thus, we contemplated the use of streptocyanine as a new amine catalysis activation mode. The utilization of streptocyanines generated from *N*-arylpyridiniums and amines in a catalytic process would enable unprecedented amine-catalyzed conversions of pyridinium pyridine rings to different aromatic rings. Importantly, the development of such a reaction would provide a new strategy for the skeletal editing of aromatic rings,^8,9^ which is one of the hottest topics in current organic chemistry.

**Fig. 2:**
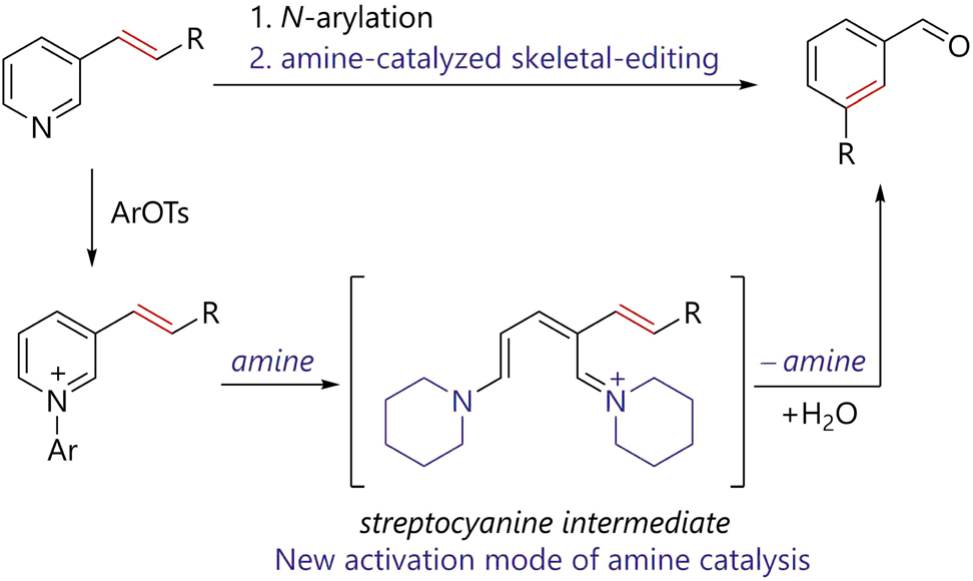
Working hypothesis for streptocyanine catalysis and its application to skeletal editing of pyridine rings to benzene rings. Ar = 2,4-dinitrophenyl.

Herein, we report the amine-catalyzed skeletal editing of pyridinium pyridine rings *via* streptocyanine intermediates to realize pyridine to benzene conversion. The working hypothesis of the reaction is outlined in Fig. 2. Reactions of pyridines bearing an alkenyl substituent at the 3-position with 2,4-dinitrophenyl tosylate affords the corresponding *N*-arylpyridiniums. The resulting pyridiniums react with a catalytic amount of piperidine to generate streptocyanine intermediates. The catalytically generated streptocyanine forms a benzene ring *via* ring-closing reaction with concomitant release of the amine catalyst. The process allows for the incorporation of the alkene moiety in the starting pyridine into the benzene ring of the product. The outcome suggests that streptocyanine can be regarded as a new amine catalysis activation mode.

## Results and discussion

### Optimization of reaction conditions

The reaction conditions were optimized for the amine-catalyzed conversion of *N*-arylpyridinium 1a, which was readily prepared from the corresponding pyridine (see ESI†), to the corresponding benzene derivative 2a. The reaction of 1a, a catalytic amount of piperidine, potassium carbonate, and water in THF at 40 °C for 18 h delivered 2a in 58% yield (Table 1, entry 1). The desired products were not formed in the absence of piperidine (entry 2), suggesting the catalytic role of piperidine in the present transformation. The use of several other secondary amines resulted in decreased yields (entries 3–5). Triethylamine and 1,8-diazabicyclo[5.4.0]undec-7-ene (DBU) did not enable product formation (entries 6 and 7). Potassium carbonate and water were found to be essential for reaction efficiency (entries 8 and 9). A higher reaction temperature and longer reaction time improved the yield of 2a (entry 10).

**Table 1:** Optimization of the reaction conditions^a^

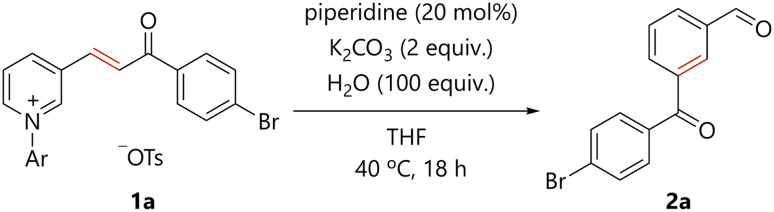				
Entry	Catalyst	Temp. (°C)	Time	Yield of 2a (%)
1	Piperidine	40	18	58^b^
2	None	40	18	0^b^
3	Et_2_NH	40	18	12^b^
4	Pyrrolidine	40	18	13^b^
5	Morpholine	40	18	50^b^
6	Et_3_N	40	18	0^b^
7	DBU	40	18	0^b^
8^c^	Piperidine	40	18	3^b^
9^d^	Piperidine	40	18	8^b^
10	Piperidine	120	42	98^e^

a Pyridinium 1a (0.1 mmol), amine catalyst (0.02 mmol), K_2_CO_3_ (0.2 mmol), H_2_O (10 mmol), and THF were stirred in a pressure tube under argon atmosphere.

b Yield determined by analysis of ^1^H NMR spectroscopy.

c The reaction was carried out in the absence of K_2_CO_3_.

d The reaction was carried out in the absence of H_2_O.

e Isolated yield. Ar = 2,4-dinitrophenyl.

### Benzene ring formation *via* amine-catalyzed activation of pyridine rings

**Scheme 1:**
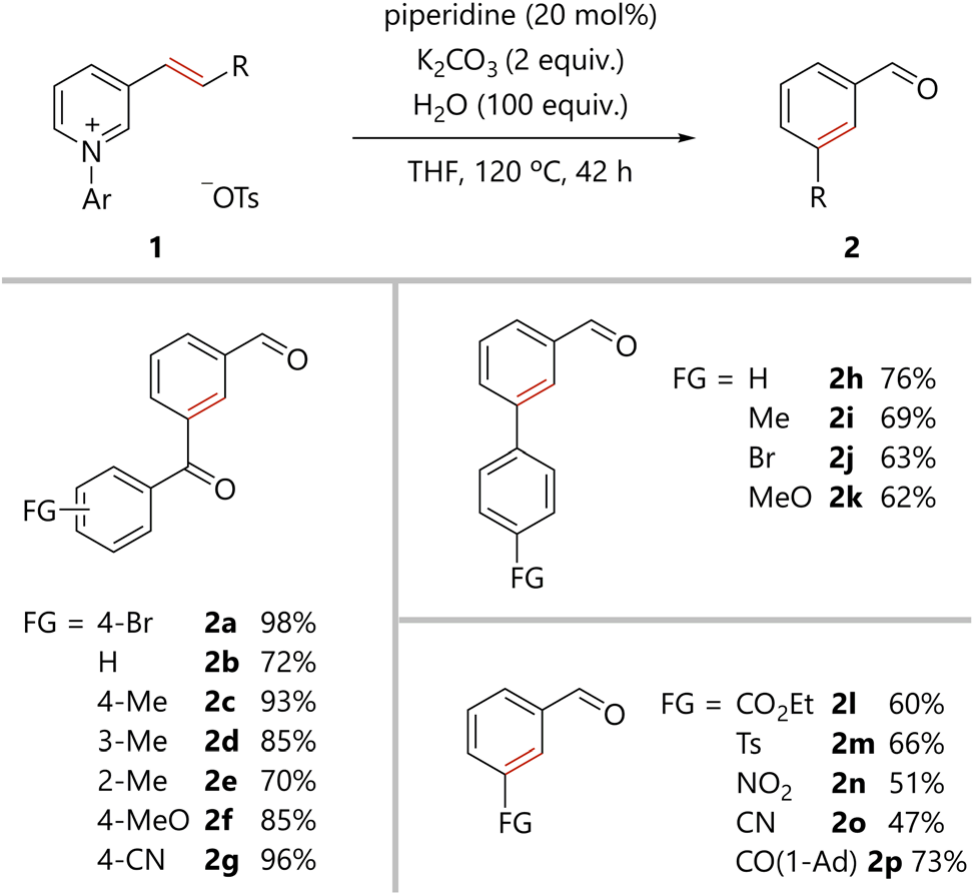
Scope of benzene ring formation *via* amine-catalyzed activation of pyridine rings. Pyridinium 1 (0.1 mmol), piperidine (0.02 mmol), K_2_CO_3_ (0.2 mmol), H_2_O (10 mmol), and THF were stirred in a pressure tube under argon atmosphere. Ar = 2,4-dinitrophenyl. Ad = adamantyl.

The scope of the present benzene ring formation *via* amine-catalyzed activation of pyridine was examined, as shown in Scheme 1. Pyridiniums 1 were readily prepared from the corresponding pyridines (see ESI†). Pyridiniums bearing aroylvinyl groups afforded the corresponding products in good to excellent yields (2a–2g). Notably, the synthetically useful bromo-substituted derivative 2a was tolerated in the present reaction. Benzoylvinyl-substituted pyridinium gave the product 2b in a good yield. All *para*-, *meta*-, and *ortho*-methyl substituted aroyl groups were tolerated in the reaction (2c–2e). *para*-Methoxy, an electron-donating group, and *para*-cyano, an electron-withdrawing group, were compatible with the reaction conditions (2f and 2g). Styrylpyridine derivatives were also converted to the corresponding biphenyl derivatives (2h–2k). Again, the desired compounds were obtained from substrates substituted with either an electron-withdrawing or electron-donating group. In addition, pyridiniums bearing an alkenyl substituent with a variety of functional groups afforded the corresponding *meta*-substituted benzaldehydes (2l–2p).

**Scheme 2:**
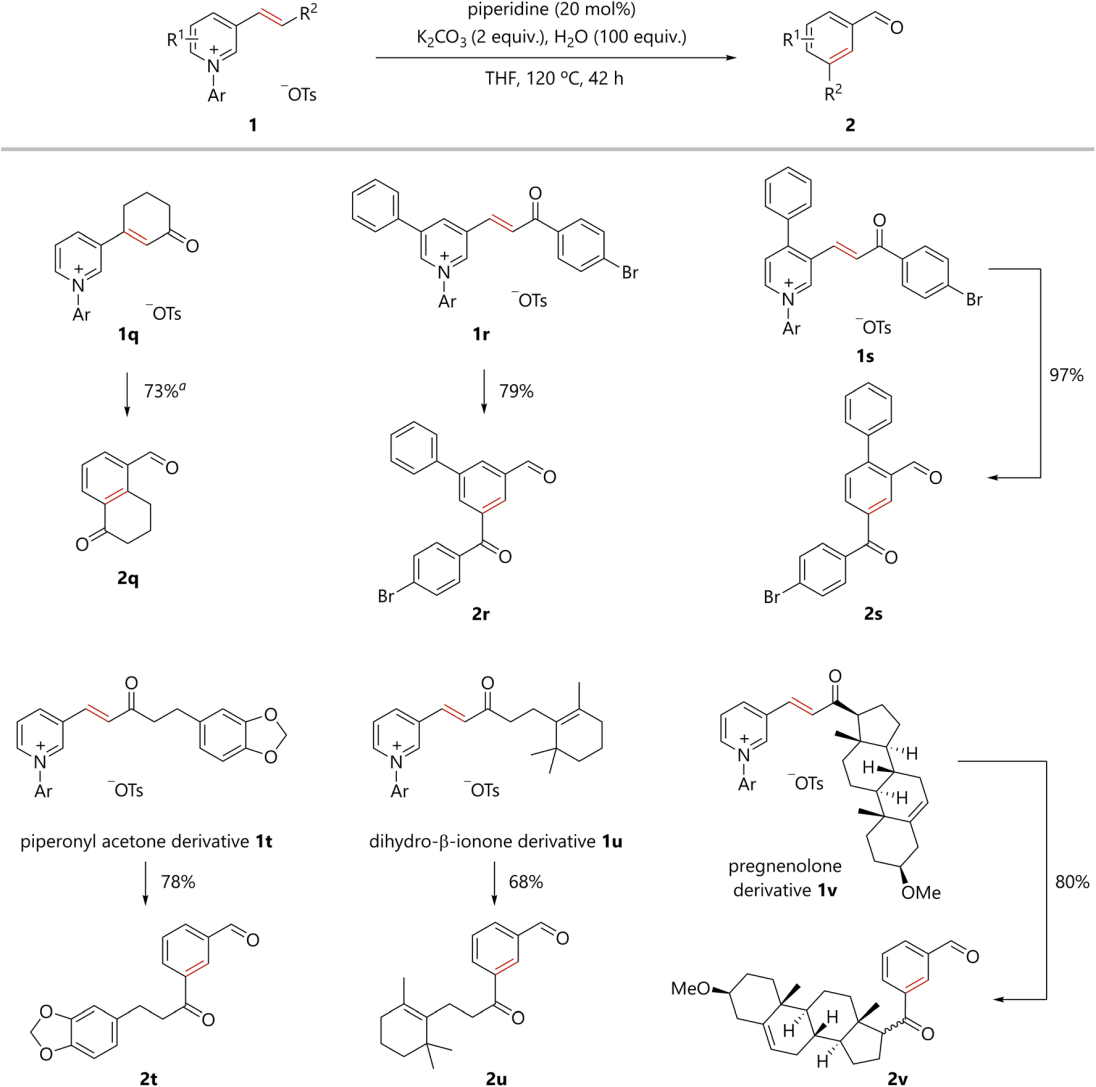
Conversion of pyridiniums to various benzene derivatives using an amine catalyst. Pyridinium 1 (0.1 mmol), piperidine (0.02 mmol), K_2_CO_3_ (0.2 mmol), H_2_O (10 mmol), and THF were stirred in a pressure tube under argon atmosphere. Ar = 2,4-dinitrophenyl. ^a^ The reaction was carried out at 40 °C for 16 hours.

The present organocatalytic conversion of pyridiniums was applied to relatively complex substrates, as illustrated in Scheme 2. Treatment of pyridinium 1q, bearing a cyclohexenone moiety, with a catalytic amount of piperidine afforded 5-formyl substituted tetralone 2q. Importantly, both the methylene and carbonyl groups that were substituted to the alkenyl carbons in 1q are located on *ortho*- and *meta*-carbon atoms of the formyl group, respectively, in benzene ring of 2q. The result confirms the incorporation of the alkene moiety into the benzene ring of the product. In addition, the reported synthesis of 2q based on electrophilic substitution cannot avoid the formation of undesired 7-formyl substituted tetralone.^10^ In contrast, 2q was obtained as a single regioisomer *via* the present method. Pyridiniums bearing both an aroylvinyl and phenyl group (1r and 1s) were also suitable substrates for the reaction, and the corresponding benzene derivatives (2r and 2s) were efficiently obtained. Notably, the synthesis of benzene derivatives bearing three different substituents at the 1,3,5- or 1,2,4-positions as in 2r and 2s would be challenging to achieve using conventional methods. The reaction of pyridinium 1t, derived from piperonyl acetone, a berry-type flavor, afforded the desired compound 2t in 78% yield. A derivative of the natural product, dihydro-β-ionone, 1u was transformed into 2u. The pyridinium synthesized from pregnenolone, an endogenous steroid, was committed to the present catalytic process to obtain the corresponding benzene derivative 2v.

**Scheme 3:**
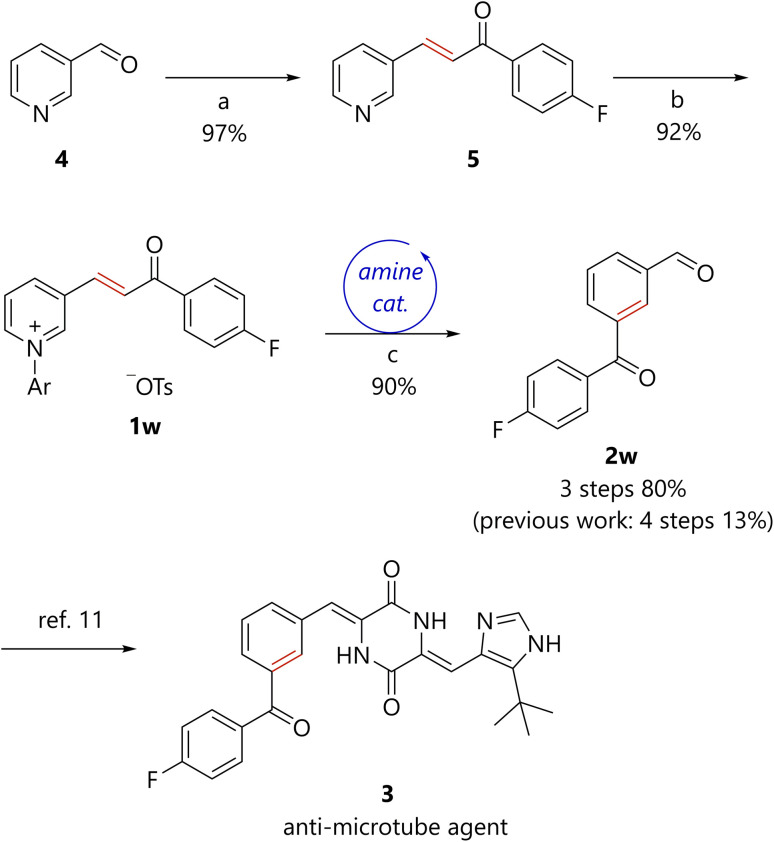
Formal synthesis of an anti-microtube agent. (a) 4′-Fluoroacetophenone, NaOH, MeOH/H_2_O, r.t., 17 h. (b) 2,4-(NO_2_)_2_C_6_H_3_OTs, toluene, reflux, 20 h. (c) Piperidine (20 mol%), K_2_CO_3_, H_2_O, THF, 120 °C, 42 h. Ar = 2,4-dinitrophenyl.

The synthesis of molecules with two different carbonyl groups often becomes complex and inefficient. By contrast, because the present method enables efficient access to benzene derivatives bearing a formyl group, when pyridines bearing an α,β-unsaturated ketone substituent were used as starting materials, benzene derivatives bearing both formyl and acyl groups in *meta*-positions were obtained in a concise manner (2a–2g, 2p–2v). Furthermore, the present method was applied to the formal synthesis of anti-microtube agent 3 (Scheme 3).^11^ Aldol condensation of nicotinaldehyde 4 with 4′-fluoroacetophenone afforded the corresponding pyridine 5. *N*-arylation of 5 generated pyridinium 1w, which was subsequently converted to 2w*via* the developed piperidine-catalyzed reaction. The total yield of the present three-step synthesis of 2w was 80%. A previously reported synthesis of 2w entailed four steps with a total yield of 13%.^11^ Thus, the synthetic efficiency of 2w has been dramatically improved by the present method. The conversion of 2w to 3 has been accomplished in one step.

### Mechanistic studies

**Scheme 4:**
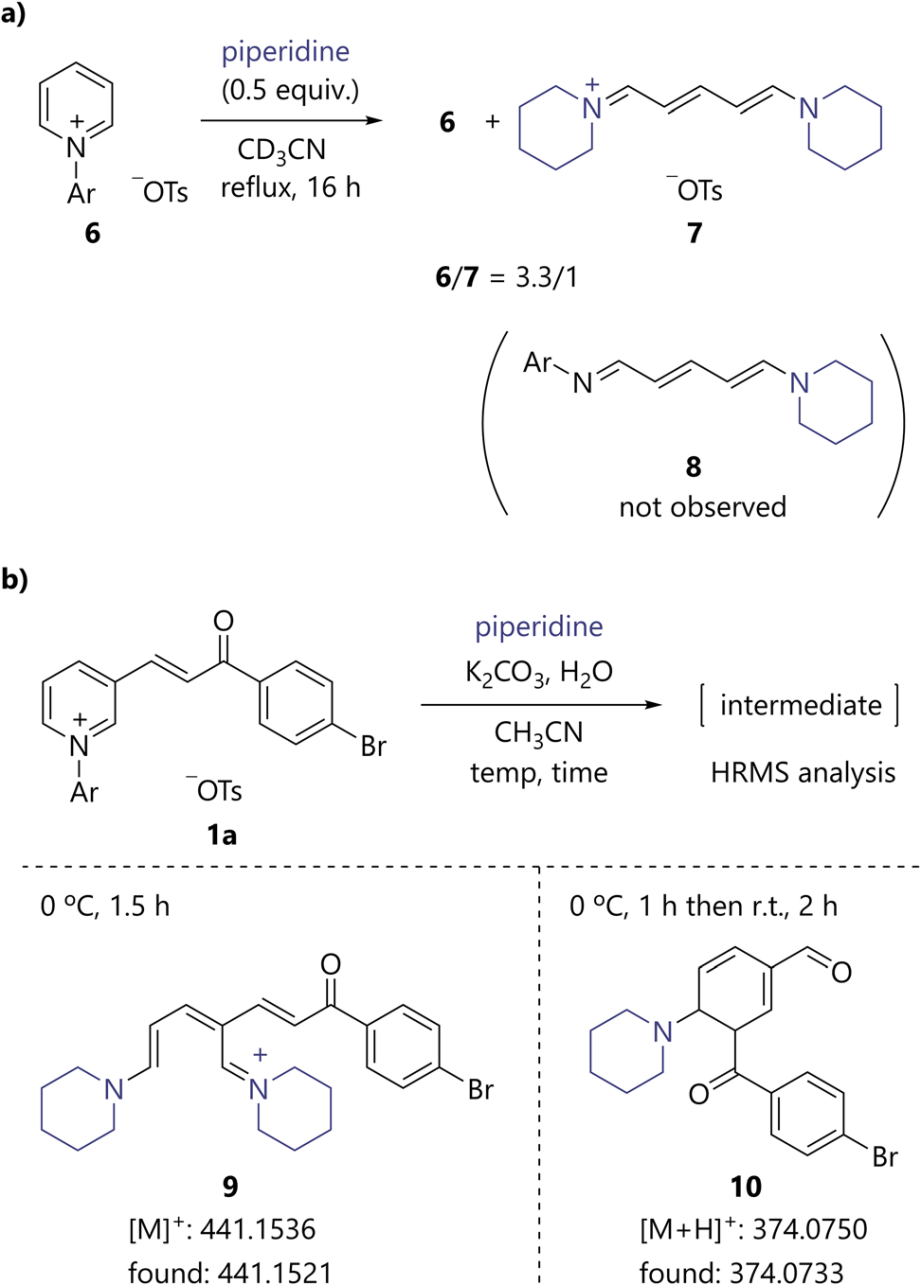
Mechanistic studies. (a) Reaction of *N*-arylpyridinium 6 with 0.5 equiv. of piperidine. (b) HRMS analysis. Ar = 2,4-dinitrophenyl.

To gain mechanistic insights into the present catalytic process, several preliminary experiments were performed. When *N*-arylpyridinium 6 was reacted with 0.5 equiv. of piperidine, the recovery of 6 and the formation of streptocyanine 7 were observed based on ^1^H NMR analysis (Scheme 4a). Interestingly, conjugated *N*-arylimine 8, which has been proposed as a reaction intermediate in the synthesis of streptocyanine,^5*a*,5*e*^ was not observed. The results support that the active species of the present benzene ring formation is streptocyanine, as described in Fig. 2. To further verify this hypothesis, 1a was treated with piperidine at 0 °C for 1.5 h and the reaction mixture was analyzed using HRMS, which confirmed the presence of streptocyanine intermediate 9 (Scheme 4b). In addition, an ion peak consistent with protonated cyclohexadiene 10 was found when the reaction mixture was analyzed after stirring at 0 °C for 1 h and then at room temperature for 2 h.

**Scheme 5:**
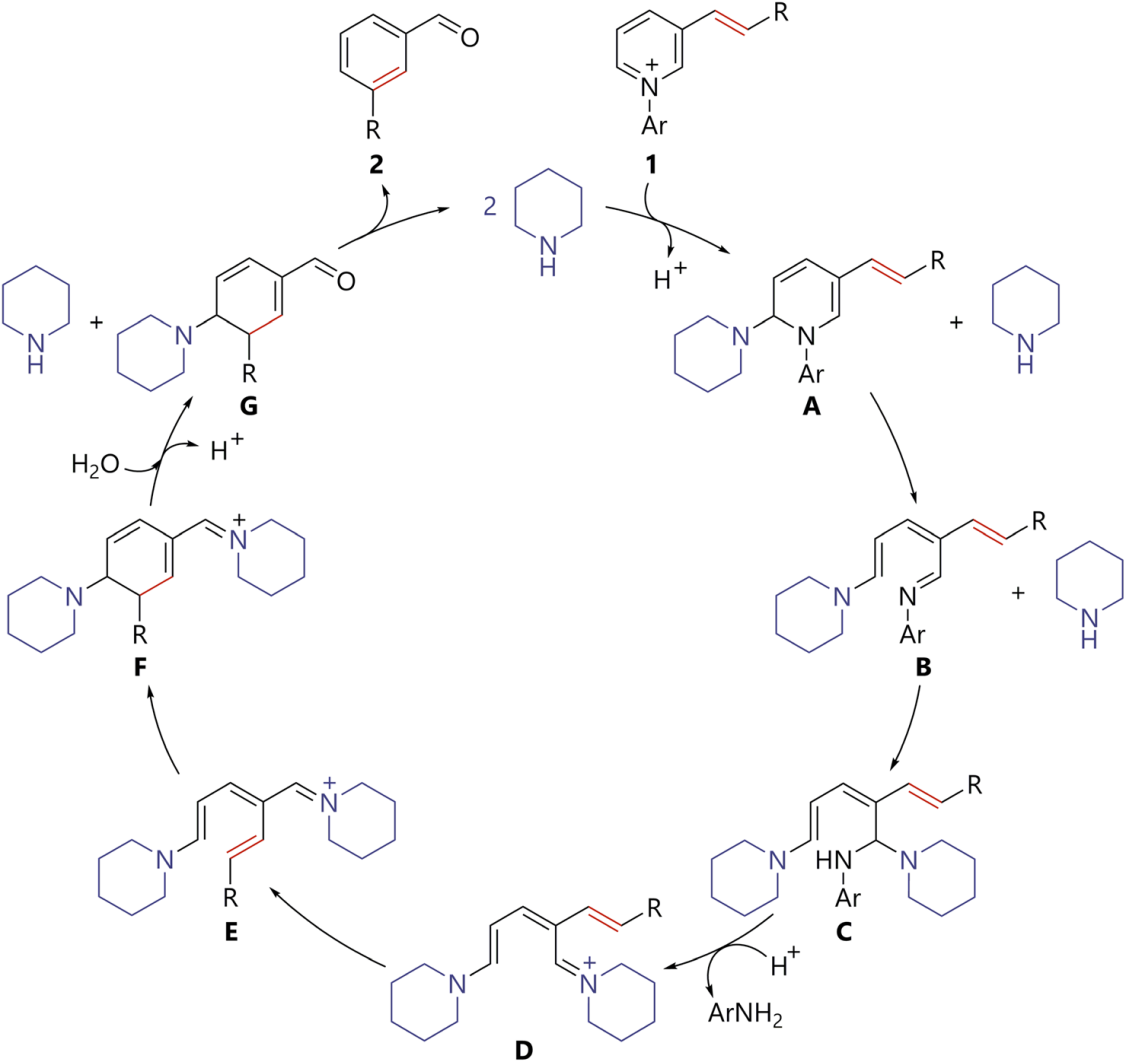
Plausible reaction mechanism for benzene ring formation *via* amine-catalyzed skeletal-editing of the pyridine ring in pyridiniums.

Based on the results, a plausible reaction mechanism for the amine-catalyzed skeletal-editing of pyridinium pyridine rings is described in Scheme 5. Piperidine attacks the 2-position of pyridinium 1 to generate piperidine adduct A. 6π-Electrocyclic ring-opening of A delivers B. A second nucleophilic addition of piperidine to the imine moiety of B generates streptocyanine D through aminal C. Streptocyanine D is then converted thermally to the *E*/*Z* isomer E because of the push–pull electronic structure.^5*d*^ Ring closing of E*via* a 6π-electrocyclic reaction and hydrolysis of resulting F affords cyclohexadiene G. It should be noted that streptocyanines are generally stable against hydrolysis under weakly basic conditions because their positive charge is delocalized. Therefore, although the hydrolysis of D or E could not be excluded, that of readily hydrolysable conjugated imine F would be more plausible. Aromatization of G results in the production of desired product 2 and regeneration of the piperidine catalyst. This mechanism could be regarded as an amine-catalyzed variation of ANRORC reactions.^12^ Overall, the use of streptocyanine as an amine catalysis activation mode enabled the present process, entailing ring opening of a pyridine ring, thermal *E*/*Z* isomerization due to the unique electronic structure of streptocyanine, and 6π-electrocyclization to form a benzene ring.

## Conclusions

In summary, we have demonstrated that streptocyanine can be used as a new activation mode of amine catalysis and applied to the conversion of pyridine rings to benzene rings. Pyridinium derivatives bearing various alkene moieties were efficiently converted to formyl benzene derivatives. Moreover, the developed process is applicable to complex molecules derived from a flavor or natural products. The synthetic utility of the method was demonstrated *via* the efficient syntheses of an anti-microtube agent and benzene derivatives bearing three different substituents. Mechanistic studies supported the hypothesis that streptocyanine is an intermediate in the present catalytic process. It is anticipated that streptocyanine catalysis will have an impact on various chemical areas, just as iminiums, enamines, and radical cations of enamines have done.

## Data availability

All the experimental procedures and characterization data are available in the ESI.†

## Author contributions

T. M. conceptualized the project and designed the experiments. T. M. and N. K. directed the project. S. N., A. W., and K. I. performed the experiments and analyzed the data. T. M. and N. K. co-wrote the manuscript. All authors discussed the experiments and commented on the manuscript.

## Conflicts of interest

There are no conflicts to declare.

## Supplementary Material

SC-014-D2SC06225A-s001
