# Supplementary material for: Streptocyanine as an activation mode of amine catalysis for the conversion of pyridine rings to benzene rings
Source: Chem Sci. 2022 Dec 21;14(3):485–90. doi: 10.1039/d2sc06225a (PMC9847661; doi:10.1039/d2sc06225a)
Supplement: SC-014-D2SC06225A-s001 [file SC-014-D2SC06225A-s001.pdf]

Electronic Supplementary Material (ESI) for Chemical Science.
This journal is © The Royal Society of Chemistry 2022

Supporting Information

Streptocyanine as an activation mode of amine catalysis for the conversion of pyridine rings to benzene rings

Tatsuya Morofuji,* Shota Nagai, Airi Watanabe, Kota Inagawa, Naokazu Kano*

Department of Chemistry, Faculty of Science, Gakushuin University, 1-5-1 Mejiro, Toshima-ku, Tokyo 171-8588, Japan

*tatsuya.morofuji@gakushuin.ac.jp
*naokazu.kano@gakushuin.ac.jp

1. **General** **S2**
2. **Synthesis of pyridines** **S3-S6**
3. **Synthesis of pyridiniums** **S6-S14**
4. **Procedure of benzene ring formation via amine-catalyzed activation of pyridine rings** **S14-S20**
5. **Unsuccessful substrates** **S20**
6. **NMR and HRMS spectra** **S21-S133**

1. General

3-Pyridylaldehyde, 4'-cyanoacetophenone, 4'-bromoacetophenone, piperonyl acetone, piperidine, acetic acid, dihydro-β-ionone, 4'-fluoroacetophenone, 3-(2-nitroethenyl)pyridine, NaOH, MeOH, toluene, and Et_2O were commercially available and were used without further purification. 4-Bromophenyl 2-(3-pyridyl)vinyl ketone,[1] phenyl 2-(3-pyridyl)vinyl ketone,[2] 4-methylphenyl 2-(3-pyridyl)vinyl ketone,[1] 3-methylphenyl 2-(3-pyridyl)vinyl ketone,[3] 2-methylphenyl 2-(3-pyridyl)vinyl ketone,[3] 4-methoxyphenyl 2-(3-pyridyl)vinyl ketone,[4] 3-styrylpyridine,[5] 3-[2-(4-methylphenyl)ethenyl]pyridine,[6] 3-[2-(4-bromophenyl)ethenyl]pyridine,[7] 3-[2-(4-methoxyphenyl)ethenyl]pyridine,[8] 3-[2-(ethoxycarbonyl)ethenyl]pyridine,[9] 3-[2-(toluenesulfonyl)ethenyl]pyridine,[10] 3-(2-cyanoethenyl)pyridine,[11] 3-(3-pyridinyl)-2-cyclohexen-1-one,[12] 5-phenylnicotinaldehyde,[13] 4-phenylnicotinaldehyde,[14] and *O*-methylated pregnenolone[15] were synthesized according to the reported literatures. Super dehydrated THF was used for solvent. Flash chromatography was carried out on a silica gel (Kanto Chem. Co., Silica Gel N, spherical, neutral, 40-100 μm). Preparative gel permeation chromatography (GPC) was carried out on Japan Analytical Industry LC-918 equipped with JAIGEL-1H and 2H using $CHCl_3$ as an eluent. All NMR spectra were measured on Resonance ECZ 400S (JEOL, 400 MHz for ^{1}H, 100 MHz for ^{13}C) or AVANCE III HD Nano Bay (Bruker Co., 400 MHz for ^{1}H, 100 MHz for ^{13}C) at 22 ºC using $CDCl_3$ as a solvent unless otherwise noted. Tetramethylsilane (TMS) (δ = 0), $CHCl_3$ (δ = 7.26), or DMSO (δ = 2.50) served as an internal standard for ^{1}H NMR spectra, and $CDCl_3$ (δ = 77.16) or DMSO-d_6 (δ = 39.52) was used as an internal standard for ^{13}C NMR spectra. MicrOTOF (Bruker Co., TOF type analyzer) was used for ESI-HRMS measurements.

[1] V. A. Mamedov, L. V. Mustakimova, O. A. Gerasimov and A. T. Gubaidullin, *Russ. Chem. Bull.*, 2020, **69**, 517-524.
[2] L. E. Downs, D. M. Wolfe and P. R. Schreiner, *Adv. Synth. Catal.*, 2005, **347**, 235-238.
[3] N. Kahriman, K. Peker, V. Serdaroğlu, A. Aydın, A. Usta, S. Fandaklı and N. Yaylı, *Bioorg. Chem.*, 2020, **99**, 103805.
[4] H. B. Lad, R. R. Giri and D. I. Brahmbhatt, *Chin. Chem. Lett.*, 2013, **24**, 227-229.
[5] X. Cui, J. Li, L. Liu and Q. X. Guo, *Chin. Chem. Lett.*, 2007, **18**, 625-628.
[6] M. L. Kantam, P. V. Redddy, P. Srinivas, A. Venugopal, S. Bhargava and Y. Nishina, *Catal. Sci Technol.*, 2013, **3**, 2550-2554.
[7] J.-S. Yang, S.-Y. Chiou and K.-L. Liau, *J. Am. Chem. Soc.*, 2002, **124**, 2518-2527.
[8] R. Martí-Centelles, J. Murga, E. Falomir, M. Carda and J. Alberto Marco, *Med. Chem. Commun.*, 2015, **6**, 1809-1815.
[9] S. Frattini, M. Quai and E. Cereda, *Tetrahedron Lett.*, 2001, **42**, 6827-6829.
[10] T. Nishimura, Y. Takiguchi and T. Hayashi, *J. Am. Chem. Soc.*, 2012, **134**, 9086-9089.
[11] K. Mei, J. Wang and X. Hu, *Synth. Commun.*, 2006, **36**, 2525-2532.
[12] K. Okada, T. Sato, Y. Kohno, and M. Nomura, *WO2010-JP54479.*
[13] N. Ullah, *Z. Naturforsch. B*, 2012, **67**, 75-84.
[14] J. Zhu, Y. Ye, M. Ning, A. Mándi, Y. Feng, Q. Zou, T. Kurtán, Y. Leng and J. Shen, *ChemMedChem*, 2013, **8**, 1210-1223.
[15] Y. Hirayama, K. Okuzumi, H. Masubuti, H. Uekusa, J.-P. Girault and Y. Fujimoto, *J. Org. Chem.*, 2014, **79**, 5471-5477.

2. Synthesis of pyridines

A 100 mL round-bottom flask equipped with a magnetic stirring bar and a septum was purged with argon gas. NaOH (0.42 g, 11 mmol), MeOH (2 mL), H_2O (10 mL) were added to the flask. 3-Pyridylaldehyde (1.9 mL, 20 mmol) and 4'-cyanoacetophenone (1.5 g, 10 mmol) were added to the mixture at 0 °C. The reaction mixture was stirred at room temperature for 17 hours. The resulting solid was collected by filtration and washed with cold water. The solid was dried over P_2O_5 under vacuum overnight to give 4-cyanophenyl 2-(3-pyridyl)vinyl ketone (**S1g**) as white solid (1.7 g, 74%).

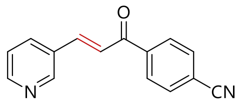 **S1g**	**4-cyanophenyl 2-(3-pyridyl)vinyl ketone (S1g)** ^{1}H NMR (400 MHz, $CDCl_3$): δ 8.86 (s, 1H), 8.66 (d, *J* = 4.8 Hz,1H), 8.09 (d, *J* = 8.0 Hz, 2H), 7.96 (d, *J* = 7.6 Hz, 1H), 7.85-7.78 (m, 3H), 7.54 (d, *J* = 15.6 Hz, 1H), 7.41-7.35 (m, 1H); ^{13}C NMR (100 MHz, $CDCl_3$): δ 188.6, 151.8, 150.3, 142.8, 141.0, 134.9, 132.7, 130.3, 129.0, 124.0, 123.0, 118.0, 116.4; HRMS (ESI, positive) *m/z* calcd for $C_{15}H_{11}N_2O$ $[M+H]^+$: 235.0866, found: 235.0874; m.p. 155-158 °C.

A 50 mL round-bottom flask equipped with a magnetic stirring bar and a septum was purged with argon gas. NaOH (1.1 g, 26 mmol), and MeOH (20 mL) were added to the flask. 3-Pyridylaldehyde (1.1 g, 10 mmol) and 1-acetyladamantane (1.8 g, 10 mmol) were added to the mixture at 0 °C. The reaction mixture was stirred at room temperature for 16 hours. The resulting solid was collected by filtration and washed with cold water. The solid was dried over P_2O_5 under vacuum overnight to give 1-adamantyl 2-(3-pyridyl)vinyl ketone (**S1p**) as yellow solid (1.4 g, 53%).

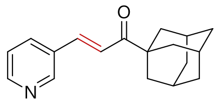 **S1p**	**1-adamantyl 2-(3-pyridyl)vinyl ketone (S1p)** ^{1}H NMR (400 MHz, $CDCl_3$): δ 8.85 (d, *J* = 1.6 Hz, 1H), 8.65 (dd, *J* = 4.6, 1.0 Hz, 1H), 7.94 (dt, *J* = 8.0, 1.8 Hz, 1H), 7.70 (d, *J* = 15.6 Hz, 1H), 7.42-7.37 (m, 1H), 7.31 (d, *J* = 15.6 Hz, 1H), 2.19-2.13 (m, 3H), 1.97-1.76 (m, 12H); ^{13}C NMR (100 MHz, $CDCl_3$): δ 203.2, 150.7, 149.8, 139.0, 134.4, 130.8, 123.6, 122.2, 45.5, 37.9, 36.5, 27.8; HRMS (ESI, positive) *m/z* calcd for $C_{18}H_{22}NO$ $[M+H]^+$: 268.1696, found: 268.1694; m.p. 50-53 °C.

A 50 mL round-bottom flask equipped with a magnetic stirring bar and a septum was purged with argon gas. NaOH (98 mg, 2.5 mmol), MeOH (10 mL), 5-phenylnicotinaldehyde (0.19 g, 1.1 mmol), and 4'-bromoacetophenone (0.20 g, 1.0 mmol) were added to the flask. The reaction mixture was stirred at room temperature for 16 hours. The reaction mixture was neutralized by HCl *aq*. (0.5 M). H_2O (20 mL) was added to the flask. The resulting solid was collected by filtration and washed with cold water. The solid was dried over P_2O_5 under vacuum overnight to give compound **S1r** as white solid (0.32 g, 88%).

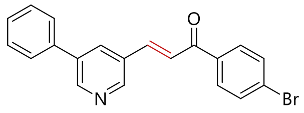 **S1r**	**Compound S1r** ^{1}H NMR (400 MHz, $CDCl_3$): δ 8.87 (s, 1H), 8.83 (s, 1H), 8.10 (s, 1H), 7.92 (d, *J* = 8.4 Hz, 2H), 7.87 (d, *J* = 16.0 Hz, 1H), 7.67 (d, *J* = 8.4 Hz, 2H), 7.65-7.58 (m, 3H), 7.52 (t, *J* = 7.2 Hz, 2H), 7.46 (t, *J* = 7.2 Hz, 1H); ^{13}C NMR (100 MHz, $CDCl_3$): δ

	188.8, 150.0, 148.7, 141.6, 137.2, 136.6, 133.1, 132.2, 130.5, 130.2, 129.4, 128.7, 128.5, 127.4, 123.6 (One peak is missing due to overlapping); HRMS (ESI, positive) *m/z* calcd for $C_{20}H_{15}BrNO$ $[M+H]^+$: 364.0332, found: 364.0334; m.p. 209-211 °C.

A 50 mL round-bottom flask equipped with a magnetic stirring bar and a septum was purged with argon gas. NaOH (0.18 g, 4.6 mmol), MeOH (10 mL), 4-phenylnicotinaldehyde (0.19 g, 1.1 mmol), and 4’-bromoacetophenone (0.22 g, 1.1 mmol) were added to the flask. The reaction mixture was stirred at room temperature for 16 hours. The reaction mixture was neutralized by HCl *aq*. (0.5 M). H_2O (20 mL) was added to the flask. The resulting solid was collected by filtration and washed with cold water. Synthesized pyridines were dried over P_2O_5 under vacuum overnight to give compound **S1s** as white solid (0.26 g, 66%).

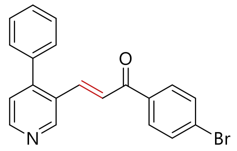<br>**S1s**	**Compound S1s**<br>^{1}H NMR (400 MHz, $CDCl_3$): δ 8.98 (s, 1H), 8.63 (d, *J* = 4.8 Hz, 1H), 7.86-7.76 (m, 3H), 7.60 (d, *J* = 8.4 Hz, 2H), 7.50-7.41 (m, 4H), 7.37-7.29 (m, 3H); ^{13}C NMR (100 MHz, $CDCl_3$): δ 188.5, 150.5, 150.0, 148.8, 141.2, 137.3, 136.4, 132.0, 130.1, 129.3, 129.04, 129.01, 128.9, 128.3, 124.6, 124.0; HRMS (ESI, positive) *m/z* calcd for $C_{20}H_{15}BrNO$ $[M+H]^+$: 364.0332, found: 364.0324; m.p. 150-153 °C.

A 100 mL round-bottom flask equipped with a magnetic stirring bar and Dean-Stark apparatus was purged with argon gas. 3-Pyridylaldehyde (0.54 g, 5.0 mmol), piperonyl acetone (0.96 g, 5.0 mmol), piperidine (0.43 g, 5.0 mmol), acetic acid (0.30 g, 5.0 mmol), and benzene (30 mL) were added to the flask. The reaction mixture was refluxed for 16 hours and was cooled to room temperature. $NaHCO_3$ *sat. aq*. (10 mL) was added to the mixture, and organic compounds were extracted with EtOAc (20 mL × 3). The organic layer was dried over Na_2SO_4. Evaporation of the solvents gave a crude material, which was purified by flash chromatography (Hex/EtOAc = 3/1) to give compound **S1t** as orange oil (0.68 g, 48%).

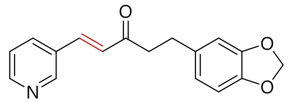<br>**S1t**	**Compound S1t**<br>^{1}H NMR (400 MHz, $CDCl_3$): δ 8.70 (s, 1H), 8.56 (d, *J* = 1.6 Hz, 1H), 7.80 (dt, *J* = 6.0, 1.6 Hz, 1H), 7.48 (d, *J* = 16.4 Hz, 1H), 7.34-7.27 (m, 1H), 6.79-6.61 (m, 4H), 5.86 (d, *J* = 2.0 Hz, 2H), 2.97-2.82 (m, 4H); ^{13}C NMR (100 MHz, $CDCl_3$): δ 198.6, 151.1, 149.9, 147.7, 145.9, 138.8, 134.7, 134.4, 130.3, 127.8, 123.8, 121.1, 108.9, 108.3, 100.8, 42.9, 29.7; HRMS (ESI, positive) *m/z* calcd for $C_{17}H_{16}NO_3$ $[M+H]^+$: 282.1125, found: 282.1116.

A 100 mL round-bottom flask equipped with a magnetic stirring bar and Dean-Stark apparatus was purged with argon gas. 3-Pyridylaldehyde (0.53 g, 5.0 mmol), dihydro-β-ionone (0.73 g, 5.0 mmol), piperidine (0.43 g, 5.0 mmol), acetic acid (0.20 g, 3.3 mmol), and benzene (30 mL) were added to the flask. The reaction mixture was refluxed for 16 hours and was cooled to room temperature. $NaHCO_3$ *sat. aq*. (10 mL) was added to the mixture, and organic compounds were extracted with EtOAc (20 mL × 3). The organic layer was dried over Na_2SO_4. Evaporation of the

solvents gave a crude material, which was purified by flash chromatography (Hex/EtOAc = 3/1) to give compound **S1u** as orange oil (0.62 g, 44%).

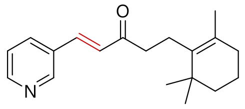 **S1u**	**Compound S1u** ^{1}H NMR (400 MHz, $CDCl_3$): δ 8.76 (d, *J* = 2.4 Hz, 1H), 8.60 (dd, *J* = 4.8, 1.6 Hz, 1H), 7.86 (dt, *J* = 7.9, 1.8 Hz, 1H), 7.53 (d, *J* = 16.4 Hz, 1H), 7.33 (dd, *J* = 8.0, 4.8 Hz, 1H), 6.78 (d, *J* = 16.4 Hz, 1H), 2.78-2.71 (m, 2H), 2.40-2.31 (m, 2H), 1.92 (t, *J* = 6.2 Hz, 2H), 1.63-1.52 (m, 5H), 1.48-1.41 (m 2H), 1.01 (s, 6H); ^{13}C NMR (100 MHz, $CDCl_3$): δ 199.8, 151.0, 149.9, 138.4, 136.1, 134.5, 130.5, 128.1, 128.0, 123.9, 42.0, 39.8, 35.1, 32.8, 28.6, 22.6, 19.9, 19.5; HRMS (ESI, positive) *m/z* calcd for $C_{19}H_{26}NO$ $[M+H]^+$: 284.2009, found: 284.2003.

A 100 mL round-bottom flask equipped with a magnetic stirring bar and a septum was purged with argon gas. *O*-methylated pregnenolone (0.38 g, 1.1 mmol), MeOH (10 mL), KOH (0.13 g, 2.3 mmol), and 3-pyridylaldehyde (0.15, 1.4 mmol) were added to the flask. The reaction mixture was stirred at room temperature for 16 hours. After the reaction, the flask was cooled to 0 °The resulting solid was collected by filtration and wash with cold water. The solid was dried over P_2O_5 under vacuum overnight to give compound **S1v** as white solid (0.30 g, 63%).

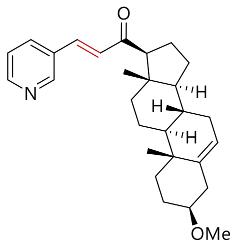 **S1v**	**Compound S1v** ^{1}H NMR (400 MHz, $CDCl_3$): δ 8.77 (s, 1H), 8.60 (d, *J* = 3.6 Hz, 1H), 7.85 (d, *J* = 8.0 Hz, 1H), 7.53 (d, *J* = 16.0 Hz, 1H), 7.36-7.30 (m, 1H), 6.83 (d, *J* = 16.0 Hz, 1H), 5.39-5.31 (m, 1H), 3.36 (s, 3H), 3.13-3.01 (m, 1H), 2.88-2.75 (m, 1H), 2.44-2.30 (m, 2H), 2.20-2.12 (m, 1H), 2.08-1.82 (m, 4H), 1.79-1.65 (m, 2H), 1.59-1.22 (m, 9H), 1.11-0.97 (m, 4H), 0.64 (s, 3H); ^{13}C NMR (100 MHz, $CDCl_3$): δ 200.0, 151.1, 150.0, 141.0, 137.8, 134.6, 130.8, 128.6, 123.9, 121.4, 80.4, 62.5, 57.3, 55.8, 50.2, 45.3, 39.3, 38.8, 37.3, 37.1, 32.1, 32.0, 28.1, 24.8, 22.8, 21.2, 19.5, 13.6; HRMS (ESI, positive) *m/z* calcd for $C_{28}H_{38}NO_2$ $[M+H]^+$: 420.2897, found: 420.2883; m.p. 103-106 °C.

A 100 mL round-bottom flask equipped with a magnetic stirring bar and a septum was purged with argon gas. NaOH (0.42 g, 11 mmol), MeOH (2 mL), H_2O (10 mL) were added to the flask. 3-Pyridylaldehyde (1.9 mL, 20 mmol) and 4'-fluoroacetophenone (1.4 g, 9.8 mmol) were added to the mixture at 0 °C. The reaction mixture was stirred at room temperature for 17 hours. The resulting solid was collected by filtration and wash with cold water. The solid was dried over P_2O_5 under vacuum overnight to give 4-cyanophenyl 2-(3-pyridyl)vinyl ketone (**5**) as white solid (2.2 g, 97%).

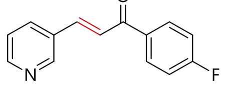 **5**	**4-fluorophenyl 2-(3-pyridyl)vinyl ketone (5)[16]** ^{1}H NMR (400 MHz, $CDCl_3$): δ 8.85 (s, 1H), 8.62 (d, *J* = 4.4, 1H), 8.05 (t, *J* = 6.6 Hz, 2H), 7.94 (d, *J* = 8.0 Hz, 1H), 7.78 (d, *J* = 16.0 Hz, 1H), 7.56 (d, *J* = 16.0 Hz, 1H), 7.35 (t, *J* = 6.2 Hz, 1H), 7.18 (t, *J* = 8.2 Hz, 2H); ^{13}C NMR (100 MHz, $CDCl_3$): δ 188.2, 165.9 (d, *J* =

[16] M. Shekarchi, M. Pirali-Hamedani, L. Navidpour, N. Adib and A. Shafiee, *J. Iran. Chem. Soc.*, 2008, **5**, 150-158.

	253.6 Hz), 151.3, 150.1, 141.3, 134.7, 134.2 (d, *J* = 2.9 Hz), 131.3 (d, *J* = 9.3 Hz), 130.7, 123.9, 123.5, 116.0 (d, *J* = 21.7 Hz); ^{19}F NMR (376 MHz, $CDCl_3$): δ –104. 75 - –104. 85 (m).

3. Synthesis of pyridiniums

A 100 mL round-bottom flask equipped with a magnetic stirring bar and a septum was dried under vacuum with heating. After cooling the flask to room temperature, it was purged with argon gas. Alkenylpyridine, toluene, and 2,4-dinitrophenyltosylate were added to the flask. The mixture was refluxed for 20 hours. The resulting mixture was opened to air and solvents were evaporated under vacuum to give a crude material. The resulting solid was collected by filtration, washed with Et_2O, and dried over P_2O_5 under vacuum overnight to give the title compounds.

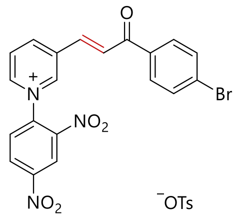<br>**1a**	**Compound 1a**<br>Reaction of 4-bromophenyl 2-(3-pyridyl)vinyl ketone (1.4 g, 5.0 mmol) with 2,4-dinitrophenyltosylate (2.5 g, 7.5 mmol) in toluene (15 mL) gave compound **1a** (1.9 g, 60%, gray solid).<br>^{1}H NMR (400 MHz, DMSO-*d*$_6$): δ 9.94 (s, 1H), 9.41 (dd, *J* = 7.2, 6.4 Hz, 2H), 9.12 (d, *J* = 2.4 Hz, 1H), 9.00 (dd, *J* = 8.4, 2.4 Hz, 1H), 8.53 (dd, *J* = 8.0, 6.4 Hz, 1H), 8.47 (d, *J* = 8.4 Hz, 1H), 8.33 (d, *J* = 15.6 Hz, 1H), 8.12 (d, *J* = 8.4 Hz, 2H), 7.89 (d, *J* = 15.6 Hz, 1H), 7.83 (d, *J* = 8.8 Hz, 2H), 7.42 (d, *J* = 8.0 Hz, 2H), 7.09 (d, *J* = 8.0 Hz, 2H), 2.28 (s, 3H); ^{13}C NMR (100 MHz, DMSO-*d*$_6$): δ 187.7, 149.2, 147.0, 146.1, 146.0, 145.6, 142.8, 138.5, 137.7, 136.1, 135.6, 134.8, 132.1, 132.0, 130.8, 130.3, 128.5, 128.3, 128.1, 127.9, 125.4, 121.6, 20.8; HRMS (ESI, positive) *m/z* calcd for $C_{20}H_{13}BrN_3O_5$ $[M–C_7H_7O_3S]^+$: 454.0033, found: 454.0025; m.p.: 287-289 °C.
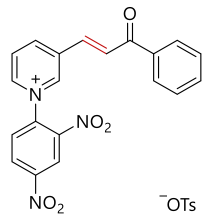<br>**1b**	**Compound 1b**<br>Reaction of phenyl 2-(3-pyridyl)vinyl ketone (1.6 g, 7.8 mmol) with 2,4-dinitrophenyltosylate (4.1 g, 12 mmol) in toluene (15 mL) gave compound **1b** (3.8 g, 88%, yellow solid).<br>^{1}H NMR (400 MHz, DMSO-*d*$_6$): δ 9.93 (s, 1H), 9.41 (t, *J* = 6.6 Hz, 2H), 9.13 (d, *J* = 2.0 Hz, 1H), 9.01 (dd, *J* = 8.4, 2.4 Hz, 1H), 8.53 (dd, *J* = 8.0, 6.4 Hz, 1H), 8.48 (d, *J* = 8.8 Hz, 1H), 8.36 (d, *J* = 15.6 Hz, 1H), 8.19 (d, *J* = 7.2 Hz, 2H), 7.88 (d, *J* = 16.0 Hz, 1H), 7.73 (t, *J* = 7.4 Hz, 1H), 7.62 (t, *J* = 7.6 Hz, 2H), 7.43 (d, *J* = 8.0 Hz, 2H), 7.09 (d, *J* = 8.0 Hz, 2H), 2.28 (s, 3H); ^{13}C NMR (100 MHz, DMSO-*d*$_6$): δ 188.5, 149.2, 147.0, 146.0, 145.9, 145.6, 142.8, 138.5, 137.7, 136.7, 135.7, 134.9, 134.0, 132.0, 130.3, 129.1, 128.9, 128.8, 128.1, 127.9, 125.5, 121.6, 20.8; HRMS (ESI, positive) *m/z* calcd for $C_{20}H_{14}N_3O_5$ $[M–C_7H_7O_3S]^+$: 376.0928, found: 362.0919; m.p.: 281-283 °C.

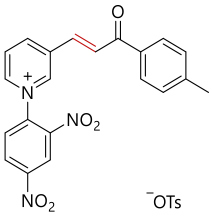<br>**1c**	**Compound 1c**<br>Reaction of 4-methylphenyl 2-(3-pyridyl)vinyl ketone (1.2 g, 5.0 mmol) with 2,4-dinitrophenyltosylate (2.5 g, 7.5 mmol) in toluene (15 mL) gave compound **1c** (2.8 g, 97%, pale yellow solid).<br>^{1}H NMR (400 MHz, DMSO-d_6): δ 9.92 (s, 1H), 9.41 (t, J = 6.6 Hz, 2H), 9.14 (d, J = 2.4 Hz, 1H), 9.02 (dd, J = 8.6, 2.6 Hz, 1H), 8.53 (dd, J = 8.0, 6.4 Hz, 1H), 8.48 (d, J = 8.8 Hz, 1H), 8.35 (d, J = 15.6 Hz, 1H), 8.10 (d, J = 8.0 Hz, 2H), 7.86 (d, J = 15.6 Hz, 1H), 7.43 (t, J = 7.4 Hz, 4H), 7.09 (d, J = 8.0 Hz, 2H), 2.42 (s, 3H), 2.28 (s, 3H); ^{13}C NMR (100 MHz, DMSO-d_6): δ 187.8, 149.2, 146.9, 145.9, 145.8, 145.7, 144.7, 142.8, 138.5, 137.6, 135.3, 135.0, 134.2, 132.0, 130.3, 129.6, 129.0, 128.9, 128.0, 127.8, 125.4, 121.5, 21.3, 20.8; HRMS (ESI, positive) *m/z* calcd for $C_{21}H_{16}N_3O_5$ [M–$C_7H_7O_3S]^+$: 390.1084, found: 390.1070; m.p.: 259-262 °C.
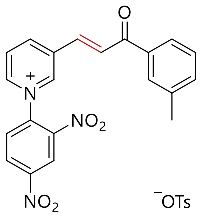<br>**1d**	**Compound 1d**<br>Reaction of 3-methylphenyl 2-(3-pyridyl)vinyl ketone (1.1 g, 5.0 mmol) with 2,4-dinitrophenyltosylate (2.5 g, 7.5 mmol) in toluene (15 mL) gave compound **1d** (2.4 g, 84%, pale yellow solid).<br>^{1}H NMR (400 MHz, DMSO-d_6): δ 9.92 (s, 1H), 9.46-9.37 (m, 2H), 9.14 (d, J = 2.4 Hz, 1H), 9.01 (dd, J = 8.4, 2.4 Hz, 1H), 8.53 (t, J = 7.0 Hz, 1H), 8.48 (d, J = 8.8 Hz, 1H), 8.34 (d, J = 16.0 Hz, 1H), 7.99 (brs, 2H), 7.87 (d, J = 16.0 Hz, 1H), 7.58-7.46 (m, 2H), 7.43 (d, J = 8.0 Hz, 2H), 7.09 (d, J = 8.0 Hz, 2H), 2.42 (s, 3H), 2.28 (s, 3H); ^{13}C NMR (100 MHz, DMSO-d_6): δ 188.5, 149.2, 146.9, 146.1, 145.7, 142.8, 138.6, 137.6, 136.7, 135.6, 134.9, 134.6, 132.0, 130.4, 130.2, 129.1, 128.9, 128.0, 127.8, 126.1, 125.5, 121.7, 121.5, 20.9, 20.8 (One peak is missing due to overlapping.); HRMS (ESI, positive) *m/z* calcd for $C_{21}H_{16}N_3O_5$ [M–$C_7H_7O_3S]^+$: 390.1084, found: 390.1079; m.p.: 198-201 °C.
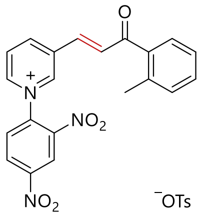<br>**1e**	**Compound 1e**<br>Reaction of 2-methylphenyl 2-(3-pyridyl)vinyl ketone (1.2 g, 5.2 mmol) with 2,4-dinitrophenyltosylate (2.5 g, 7.5 mmol) in toluene (15 mL) gave compound **1e** (2.7 g, 90%, brown solid).<br>^{1}H NMR (400 MHz, DMSO-d_6): δ 9.86 (s, 1H), 9.40 (d, J = 6.0 Hz, 1H), 9.33 (d, J = 8.4 Hz, 1H), 9.12 (d, J = 2.4 Hz, 1H), 8.99 (dd, J = 8.6, 2.6 Hz, 1H), 8.50 (dd, J = 8.2, 6.2 Hz, 1H), 8.44 (d, J = 8.8 Hz, 1H), 7.90 (d, J = 16.0 Hz, 1H), 7.78 (d, J = 8.0 Hz, 1H), 7.67 (d, J = 16.0 Hz, 1H), 7.51 (t, J = 7.4 Hz, 1H), 7.46-7.36 (m, 4H), 7.09 (d, J = 8.0 Hz, 2H), 2.44 (s, 3H), 2.28 (s, 3H); ^{13}C NMR (100 MHz, DMSO-d_6): δ 193.4, 149.2, 146.8, 146.0, 145.9, 145.7, 142.8, 138.5, 137.6, 137.42, 137.35, 136.1, 134.7, 132.3, 132.0, 131.7, 131.6, 130.3, 129.1, 128.0, 127.8, 125.9, 125.4, 121.5, 20.8, 20.3; HRMS (ESI, positive) *m/z* calcd for $C_{21}H_{16}N_3O_5$ [M–$C_7H_7O_3S]^+$:

	390.1084, found: 390.1072; m.p.: 195-198 °C.
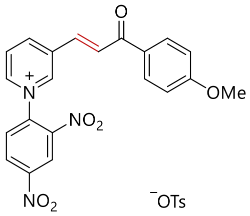<br>**1f**	**Compound 1f**<br>Reaction of 4-methoxyphenyl 2-(3-pyridyl)vinyl ketone (1.2 g, 5.0 mmol) with 2,4-dinitrophenyltosylate (2.5 g, 7.5 mmol) in toluene (15 mL) gave compound **1f** (2.8 g, 97%, pale yellow solid).<br>^{1}H NMR (400 MHz, DMSO-d_6): δ 9.92 (s, 1H), 9.44-9.36 (m, 2H), 9.14 (s, 1H), 9.02 (d, *J* = 8.8 Hz, 1H), 8.53 (t, *J* = 7.2 Hz, 1H), 8.48 (d, *J* = 8.8 Hz, 1H), 8.36 (d, *J* = 16.0 Hz, 1H), 8.19 (d, *J* = 8.0 Hz, 2H), 7.84 (d, *J* = 15.6 Hz, 1H), 7.44 (d, *J* = 7.2 Hz, 2H), 7.16-7.06 (m, 4H), 3.89 (s, 3H), 2.28 (s, 3H); ^{13}C NMR (100 MHz, DMSO-d_6): δ 186.5, 163.9, 149.3, 146.8, 145.8, 142.8, 138.5, 137.6, 135.1, 134.8, 132.0, 131.3, 130.3, 129.6, 129.0, 128.0, 127.8, 125.5, 121.6, 114.3, 55.8, 20.8 (Two peaks are missing due to overlapping.); HRMS (ESI, positive) *m/z* calcd for $C_{21}H_{16}N_3O_6$ [M–$C_7H_7O_3S$]$^+$: 406.1034, found: 406.1031; m.p.: 249-252 °C.
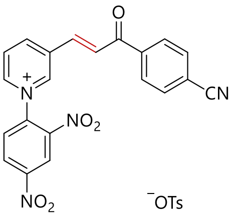<br>**1g**	**Compound 1g**<br>Reaction of 4-cyanophenyl 2-(3-pyridyl)vinyl ketone (1.2 g, 5.0 mmol) with 2,4-dinitrophenyltosylate (2.5 g, 7.5 mmol) in toluene (15 mL) gave compound **1g** (2.5 g, 87%, brown solid).<br>^{1}H NMR (400 MHz, DMSO-d_6): δ 9.94 (s, 1H), 9.43 (t, *J* = 8.6 Hz, 2H), 9.12 (s, 1H), 9.00 (dd, *J* = 8.8, 2.0 Hz, 1H), 8.54 (t, *J* = 7,2 Hz, 1H), 8.48 (d, *J* = 8.4 Hz, 1H), 8.38-8.29 (m, 3H), 8.10 (d, *J* = 8.4 Hz, 2H), 7.91 (d, *J* = 15.6 Hz, 1H), 7.42 (d, *J* = 8.0 Hz, 2H), 7.08 (d, *J* = 7.6 Hz, 2H), 2.27 (s, 3H); ^{13}C NMR (100 MHz, DMSO-d_6): δ 187.9, 149.2, 147.0, 146.2, 146.1, 145.7, 142.8, 139.8, 138.5, 137.6, 136.7, 134.7, 133.0, 132.0, 130.3, 129.4, 128.4, 128.0, 127.9, 125.4, 121.6, 118.1, 115.7, 20.8; HRMS (ESI, positive) *m/z* calcd for $C_{21}H_{13}N_4O_5$ [M–$C_7H_7O_3S$]$^+$: 401.0880, found: 401.0869; m.p.: 278-281 °C.
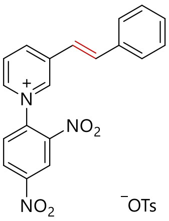<br>**1h**	**Compound 1h**<br>Reaction of 3-styrylpyridine (0.73 g, 4.0 mmol) with 2,4-dinitrophenyltosylate (2.0 g, 6.0 mmol) in toluene (10 mL) gave compound **1h** (1.93 g, 93%, yellow solid).<br>^{1}H NMR (400 MHz, DMSO-d_6): δ 9.64 (s, 1H), 9.23 (d, *J* = 5.6 Hz, 1H), 9.15 (s, 1H), 9.07 (d, *J* = 8.0 Hz, 1H), 9.01 (d, *J* = 8.8 Hz, 1H), 8.46 (d, *J* = 8.8 Hz, 1H), 8.42 (t, *J* = 6.8 Hz, 1H), 7.74 (d, *J* = 16.8 Hz, 1H), 7.67 (d, *J* = 7.2 Hz, 2H), 7.51-7.38 (m, 6H), 7.10 (d, *J* = 7.6 Hz, 2H), 2.28 (s, 3H); ^{13}C NMR (100 MHz, DMSO-d_6): δ 149.1, 145.6, 144.5, 143.6, 143.5, 142.9, 138.7, 137.6, 137.5, 136.3, 135.4, 131.9, 130.2, 129.6, 129.1, 128.0, 127.8, 127.3, 125.4, 121.4, 121.0, 20.8; HRMS (ESI, positive) *m/z* calcd for $C_{19}H_{14}N_3O_4$ [M–$C_7H_7O_3S$]$^+$: 348.0979, found: 348.0973; m.p.: 255-258 °C.

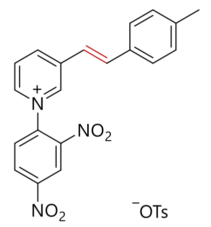<br>**1i**	**Compound 1i**<br>Reaction of 3-[2-(4-methylphenyl)ethenyl]pyridine (0.20 g, 1.0 mmol) with 2,4-dinitrophenyltosylate (0.51 g, 1.5 mmol) in toluene (5 mL) gave compound **1i** (0.48 g, 91%, yellow solid).<br>^{1}H NMR (400 MHz, DMSO-d_6): δ 9.61 (s, 1H), 9.20 (d, *J* = 5.6 Hz, 1H), 9.15 (s, 1H), 9.08-8.97 (m, 2H), 8.46 (d, *J* = 8.8 Hz, 1H), 8.40 (t, *J* = 7.0 Hz, 1H), 7.69 (d, *J* = 16.8 Hz, 1H), 7.56 (d, *J* = 7.6 Hz, 2H), 7.49-7.37 (m, 3H), 7.28 (d, *J* = 7.6 Hz, 2H), 7.10 (d, *J* = 7.6 Hz, 2H), 2.34 (s, 3H), 2.28 (s, 3H); ^{13}C NMR (100 MHz, DMSO-d_6): δ 149.2, 145.8, 144.3, 143.4, 143.3, 143.0, 139.5, 138.7, 137.7, 137.6, 136.4, 132.6, 131.9, 130.3, 129.8, 128.1, 127.8, 127.3, 125.5, 121.6, 120.0, 21.0, 20.8; HRMS (ESI, positive) *m/z* calcd for $C_{20}H_{16}N_3O_4$ $[M–C_7H_7O_3S]^+$: 362.1135, found: 362.1138; m.p.: 250-252 °C.
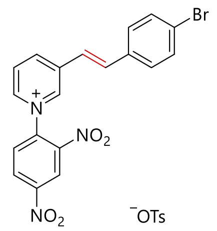<br>**1j**	**Compound 1j**<br>Reaction of 3-[2-(4-bromophenyl)ethenyl]pyridine (0.26 g, 1.0 mmol) with 2,4-dinitrophenyltosylate (0.50 g, 1.5 mmol) in toluene (5 mL) gave compound **1j** (0.58 g, 97%, pale yellow solid).<br>^{1}H NMR (400 MHz, DMSO-d_6): δ 9.65 (s, 1H), 9.25 (d, *J* = 6.0 Hz, 1H), 9.13 (d, *J* = 2.4 Hz, 1H), 9.06 (d, *J* = 8.4 Hz, 1H), 8.99 (dd, *J* = 8.6, 2.6 Hz, 1H), 8.46 (d, *J* = 8.8 Hz, 1H), 8.42 (dd, *J* = 8.4, 6.0 Hz, 1H), 7.77-7.65 (m, 3H), 7.61 (d, *J* = 8.4 Hz, 2H), 7.51 (d, *J* = 16.8 Hz, 1H), 7.44 (d, *J* = 8.0 Hz, 2H), 7.09 (d, *J* = 7.6 Hz, 2H), 2.28 (s, 3H); ^{13}C NMR (100 MHz, DMSO-d_6): δ 149.2, 145.7, 144.6, 143.8, 143.7, 142.9, 138.7, 137.6, 137.2, 135.1, 134.6, 132.1, 131.9, 130.3, 129.2, 128.0, 127.8, 125.5, 122.8, 121.9, 121.5, 20.8; HRMS (ESI, positive) *m/z* calcd for $C_{19}H_{13}BrN_3O_4$ $[M–C_7H_7O_3S]^+$: 426.0084, found: 426.0082; m.p.: 240-242 °C.
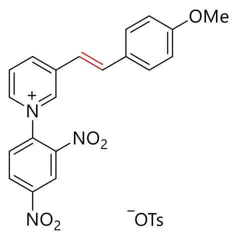<br>**1k**	**Compound 1k**<br>Reaction of 3-[2-(4-methoxyphenyl)ethenyl]pyridine (0.21 g, 1.0 mmol) with 2,4-dinitrophenyltosylate (0.51 g, 1.5 mmol) in toluene (5 mL) gave compound **1k** (0.50 g, 92%, yellow solid).<br>^{1}H NMR (400 MHz, DMSO-d_6): δ 9.58 (s, 1H), 9.17 (d, *J* = 6.0 Hz, 1H), 9.14 (s, 1H), 9.00 (t, *J* = 6.2 Hz, 2H), 8.46 (d, *J* = 8.8 Hz, 1H), 8.38 (t, *J* = 7.0 Hz, 1H), 7.68 (d, *J* = 16.4 Hz, 1H), 7.61 (d, *J* = 8.4 Hz, 2H), 7.45 (d, *J* = 7.6 Hz, 2H), 7.30 (d, *J* = 16.4 Hz, 1H), 7.10 (d, *J* = 7.2 Hz, 2H), 7.04 (d, *J* = 8.4 Hz, 2H), 3.81 (s, 3H), 2.28 (s, 3H); ^{13}C NMR (100 MHz, DMSO-d_6): δ 160.5, 149.2, 145.8, 144.0, 143.1, 143.0, 138.7, 138.0, 137.6, 136.2, 131.9, 130.3, 129.0, 128.05, 127.97, 127.8, 125.5, 121.5, 118.5, 114.6, 55.4, 20.8 (One peak is missing due to overlapping.); HRMS (ESI, positive) *m/z* calcd for $C_{20}H_{16}N_3O_5$ $[M–C_7H_7O_3S]^+$: 378.1084, found: 378.1086; m.p.: 198-200 °C.

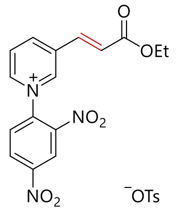<br>**1l**	**Compound 1l**<br>Reaction of 3-[2-(ethoxycarbonyl)ethenyl]pyridine (0.18 g, 1.0 mmol) with 2,4-dinitrophenyltosylate (0.51 g, 1.5 mmol) in toluene (5 mL) gave compound **1l** (0.37 g, 72%, yellow solid).<br>^{1}H NMR (400 MHz, DMSO-*d*$_6$): δ 9.79 (s, 1H), 9.38 (d, *J* = 6.0 Hz, 1H), 9.24 (d, *J* = 8.0 Hz, 1H), 9.13 (s, 1H), 9.00 (d, *J* = 8.0 Hz, 1H), 8.48 (t, *J* = 7.0 Hz, 1H), 8.42 (d, *J* = 8.8 Hz, 1H), 7.83 (d, *J* = 16.4 Hz, 1H), 7.44 (d, *J* = 7.6 Hz, 2H), 7.10 (d, *J* = 8.0 Hz, 3H), 4.24 (q, *J* = 6.9 Hz, 2H), 2.28 (s, 3H), 1.27 (t, *J* = 7.0 Hz, 3H); ^{13}C NMR (100 MHz, DMSO-*d*$_6$): δ 165.0, 149.3, 146.7, 146.0, 145.8, 145.7, 142.8, 138.4, 137.6, 136.6, 134.2, 132.0, 130.3, 128.1, 127.9, 125.7, 125.5, 121.5, 60.9, 20.8, 14.1; HRMS (ESI, positive) *m/z* calcd for $C_{16}H_{14}N_3O_6$ $[M–C_7H_7O_3S]^+$: 344.0877, found: 344.0876; m.p.: 195-197 °C.
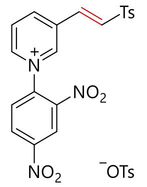<br>**1m**	**Compound 1m**<br>Reaction of 3-[2-(toluenesulfonyl)ethenyl]pyridine (0.26 g, 1.0 mmol) with 2,4-dinitrophenyltosylate (0.51 g, 1.5 mmol) in toluene (5 mL) gave compound **1m** (0.55 g, 93%, white solid).<br>^{1}H NMR (400 MHz, DMSO-*d*$_6$): δ 9.73 (s, 1H), 9.40 (d, *J* = 6.0 Hz, 1H), 9.19 (d, *J* = 8.4 Hz, 1H) , 9.12 (d, *J* = 2.8 Hz, 1H), 8.99 (dd, *J* = 8.6, 2.6 Hz, 1H), 8.48 (dd, *J* = 8.2, 6.2 Hz, 1H), 8.38 (d, *J* = 8.8 Hz, 1H), 7.98 (d, *J* = 15.2 Hz, 1H), 7.88-7.80 (m, 3H), 7.51 (d, *J* = 8.0 Hz, 2H), 7.45 (d, *J* = 8.0 Hz, 2H), 7.10 (d, *J* = 8.0 Hz, 2H), 2.42 (s, 3H), 2.28 (s, 3H); ^{13}C NMR (100 MHz, DMSO-*d*$_6$): δ 149.3, 147.4, 146.4, 146.3, 145.8, 145.2, 142.7, 138.3, 137.5, 136.2, 135.5, 133.9, 132.6, 131.9, 130.4, 130.3, 128.0, 127.7, 125.5, 121.5, 21.1, 20.8 (One peak is missing due to overlapping.); HRMS (ESI, positive) *m/z* calcd for $C_{20}H_{16}N_3O_6S$ $[M–C_7H_7O_3S]^+$: 426.0754, found: 426.0750; m.p.: 268-270 °C.
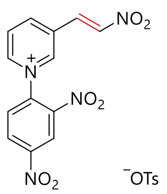<br>**1n**	**Compound 1n**<br>Reaction of 3-(2-nitroethenyl)pyridine (0.26 g, 1.5 mmol) with 2,4-dinitrophenyltosylate (0.76 g, 2.3 mmol) in toluene (5 mL) gave compound **1n** (0.42 g, 58%, brown solid).<br>^{1}H NMR (400 MHz, DMSO-*d*$_6$): δ 9.83 (s, 1H), 9.48 (d, *J* = 6.0 Hz, 1H), 9.29 (d, *J* = 8.4 Hz, 1H), 9.14 (d, *J* = 2.4 Hz, 1H), 9.01 (dd, *J* =8.6, 2.6 Hz, 1H), 8.55 (dd, *J* = 8.2, 6.2 Hz, 1H), 8.51-8.42 (m, 2H), 8.30 (d, *J* = 13.6 Hz, 1H), 7.44 (d, *J* = 8.0 Hz, 2H), 7.10 (d, *J* = 8.0 Hz, 2H), 2.28 (s, 3H); ^{13}C NMR (100 MHz, DMSO-*d*$_6$): δ 149.3, 147.8, 147.2, 146.6, 145.7, 143.0, 142.7, 138.3, 137.6, 131.9, 131.6, 130.8, 130.4, 128.04, 127.98, 125.5, 121.6, 20.8; HRMS (ESI, positive) *m/z* calcd for $C_{13}H_9N_4O_6$ $[M–C_7H_7O_3S]^+$: 317.0517, found: 317.0517; m.p.: 160-162 °C.

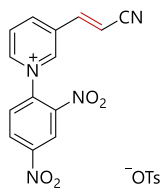<br>**1o**	**Compound 1o**<br>Reaction of 3-(2-cyanoethenyl)pyridine (0.13 g, 1.0 mmol) with 2,4-dinitrophenyltosylate (0.51 g, 1.5 mmol) in toluene (5 mL) gave compound **1o** (0.39 g, 82%, white solid).<br>^{1}H NMR (400 MHz, DMSO-*d*$_6$): δ 9.67 (s, 1H), 9.42 (d, *J* = 6.0 Hz, 1H), 9.17-9.10 (m, 2H), 9.00 (dd, *J* = 8.4, 2.4 Hz, 1H), 8.51 (dd, *J* = 8.4, 6.4 Hz, 1H), 8.42 (d, *J* = 8.4 Hz, 1H), 7.87 (d, *J* = 16.8 Hz, 1H), 7.44 (d, *J* = 8.0 Hz, 2H), 7.10 (d, *J* = 8.0 Hz, 2H), 6.91 (d, *J* = 16.8 Hz, 1H), 2.28 (s, 3H); ^{13}C NMR (100 MHz, DMSO-*d*$_6$): δ 149.3, 146.7, 146.0, 145.7, 145.4, 143.2, 142.8, 138.4, 137.6, 133.5, 131.9, 130.3, 128.0, 127.9, 125.5, 121.5, 117.2, 104.8, 20.8; HRMS (ESI, positive) *m/z* calcd for $C_{14}H_9N_4O_4$ [M–$C_7H_7O_3S$]$^+$: 297.0618, found: 297.0606; m.p.: 201-203 °C.
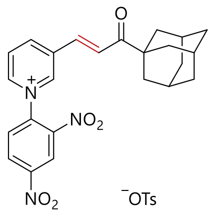<br>**1p**	**Compound 1p**<br>Reaction of 1-adamantyl 2-(3-pyridyl)vinyl ketone (0.27 g, 1.0 mmol) with 2,4-dinitrophenyltosylate (0.51 g, 1.5 mmol) in toluene (10 mL) gave compound **1o** (0.36 g, 59%, white solid).<br>^{1}H NMR (400 MHz, DMSO-*d*$_6$): δ 9.81 (s, 1H), 9.37 (d, *J* = 6.0 Hz, 1H), 9.31 (d, *J* = 8.4 Hz, 1H), 9.14 (d, *J* = 2.4 Hz, 1H), 9.01 (dd, *J* = 8.8, 2.4 Hz, 1H), 8.52-8.42 (m, 2H), 7.86 (d, *J* = 15.6 Hz, 1H), 7.67 (d, *J* = 15.6 Hz, 1H), 7.44 (d, *J* = 8.4 Hz, 2H), 7.10 (d, *J* = 8.0 Hz, 2H), 2.28 (s, 3H), 2.09-2.02 (m, 3H), 1.89-1.82 (m, 6H), 1.79-1.65 (m, 6H); ^{13}C NMR (100 MHz, DMSO-*d*$_6$): δ 202.4, 149.2, 146.9, 145.80, 145.75, 145.6, 142.8, 138.5, 137.5, 134.9, 134.2, 132.0, 130.3, 128.02, 127.98, 127.8, 125.5, 121.5, 45.2, 36.7, 35.9, 27.2, 20.8; HRMS (ESI, positive) *m/z* calcd for $C_{24}H_{24}N_3O_5$ [M-$C_7H_7O_3S$]$^+$: 434.1710, found: 434.1702; m.p.: 220-223 °C.
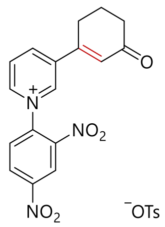<br>**1q**	**Compound 1q**<br>Reaction of 3-(3-pyridinyl)-2-cyclohexen-1-one (0.17 g, 1.0 mmol) with 2,4-dinitrophenyltosylate (0.51 g, 1.5 mmol) in toluene (5 mL) gave compound **1q** (0.47 g, 92%, pale yellow solid).<br>^{1}H NMR (400 MHz, DMSO-*d*$_6$): δ 9.72 (s, 1H), 9.39 (d, *J* = 6.0 Hz, 1H), 9.19 (d, *J* = 8.8 Hz, 1H), 9.14 (d, *J* = 2.4 Hz, 1H), 8.99 (dd, *J* = 8.6, 2.6 Hz, 1H), 8.49 (dd, *J* = 8.2, 6.2 Hz, 1H), 8.41 (d, *J* = 8.8 Hz, 1H), 7.46 (d, *J* = 8.0 Hz, 2H), 7.10 (d, *J* = 8.0 Hz, 2H), 6.74 (s, 1H), 2.93-2.70 (m, 2H), 2.49-2.40 (m, 2H), 2.28 (s, 3H), 2.11 (quint, *J* = 6.4 Hz, 2H); ^{13}C NMR (100 MHz, DMSO-*d*$_6$): δ 198.5, 151.9, 149.3, 145.9, 145.8, 145.4, 144.3, 142.9, 138.5, 137.8, 137.6, 132.2, 130.2, 128.6, 128.0, 127.7, 125.5, 121.3, 36.6, 26.5, 21.9, 20.8; HRMS (ESI, positive) *m/z* calcd for $C_{17}H_{14}N_3O_5$ [M–$C_7H_7O_3S$]$^+$: 340.0928, found: 340.0927; m.p.: 216-218 °C.

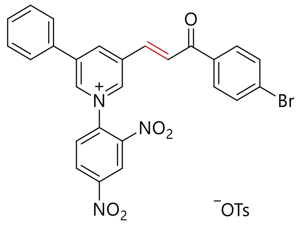<br>**1r**	**Compound 1r**<br>Reaction of **S1q** (0.36 g, 1.0 mmol) with 2,4-dinitrophenyltosylate (0.51 g, 1.5 mmol) in toluene (5 mL) gave compound **1r** (0.56 g, 81%, brown solid).<br>^{1}H NMR (400 MHz, DMSO-d_6): δ 9.86 (d, J = 8.8 Hz, 2H), 9.73 (s, 1H), 9.17 (d, J = 2.4 Hz, 1H), 9.05 (dd, J = 8.6, 2.2 Hz, 1H), 8.53 (d, J = 8.4 Hz, 1H), 8.46 (d, J = 16.0 Hz, 1H), 8.15 (d, J = 8.0 Hz, 2H), 8.04 (d, J = 7.6 Hz, 2H), 7.94 (d, J = 15.6 Hz, 1H), 7.86 (d, J = 8.4 Hz, 2H), 7.72-7.63 (m, 3H), 7.44 (d, J = 7.6 Hz, 2H), 7.10 (d, J = 8.0 Hz, 2H), 2.28 (s, 3H); ^{13}C NMR (100 MHz, DMSO-d_6): δ 187.7, 149.3, 145.7, 144.1, 143.8, 142.7, 139.3, 138.5, 137.6, 136.2, 135.7, 134.8, 132.4, 132.2, 132.1, 130.81, 130.77, 130.3, 129.6, 128.7, 128.3, 128.0, 127.8, 125.5, 121.5, 20.8 (One peak is missing due to overlapping.); HRMS (ESI, positive) *m/z* calcd for $C_{26}H_{17}BrN_3O_5$ [M–$C_7H_7O_3S$]$^+$: 530.0346, found: 530.0352; m.p.: 267-269 °C.
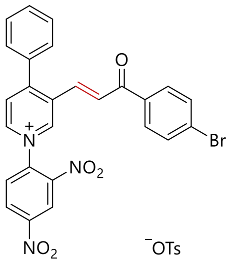<br>**1s**	**Compound 1s**<br>Reaction of **S1r** (0.20 g, 0.54 mmol) with 2,4-dinitrophenyltosylate (0.29 g, 0.85 mmol) in toluene (5 mL) gave compound **1s** (0.36 g, 94%, white solid).<br>^{1}H NMR (400 MHz, DMSO-d_6): δ 10.07 (d, J = 1.2 Hz, 1H), 9.46 (dd, J = 6.4, 1.2 Hz, 1H), 9.19 (d, J = 2.4 Hz, 1H), 9.05 (dd, J = 8.6, 2.6 Hz, 1H), 8.56 (d, J = 6.4 Hz, 1H), 8.52 (d, J = 8.4 Hz, 1H), 8.14 (d, J = 15.6 Hz, 1H), 8.03 (d, J = 8.0 Hz, 2H), 7.81 (d, J = 8.4 Hz, 2H), 7.78-7.72 (m, 5H), 7.63 (d, J = 15.6 Hz, 1H), 7.44 (d, J = 8.0 Hz, 2H), 7.09 (d, J = 7.6 Hz, 2H), 2.28 (s, 3H); ^{13}C NMR (100 MHz, DMSO-d_6): δ 187.6, 158.5, 149.3, 145.8, 145.5, 144.9, 142.9, 138.3, 137.5, 135.6, 135.4, 133.9, 132.3, 132.1, 131.7, 130.7, 130.4, 130.0, 129.5, 128.5, 128.3, 128.1, 128.0, 125.5, 121.5, 20.8 (One peak is overlapping due to overlapping.); HRMS (ESI, positive) *m/z* calcd for $C_{26}H_{17}BrN_3O_5$ [M–$C_7H_7O_3S$]$^+$: 530.0346, found: 530.0341; m.p.: 273-276 °C.
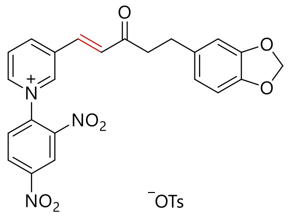<br>**1t**	**Compound 1t**<br>Reaction of **S1t** (0.29 g, 1.0 mmol) with 2,4-dinitrophenyltosylate (0.51 g, 1.5 mmol) in toluene (5 mL) gave compound **1t** (0.45 g, 72%, orange solid).<br>^{1}H NMR (400 MHz, DMSO-d_6): δ 9.78 (s, 1H), 9.38 (d, J = 6.0 Hz, 1H), 9.19 (d, J = 8.0 Hz, 1H), 9.11 (d, J = 2.4 Hz, 1H), 8.98 (dd, J = 8.6, 2.2 Hz, 1H), 8.52-8.39 (m, 2H), 7.78 (dd, J = 16.4, 1.6 Hz, 1H), 7.43 (dd, J = 8.0, 1.6 Hz, 2H), 7.28 (dd, J = 16.6, 2.2 Hz, 1H), 7.09 (d, J = 6.8 Hz, 2H), 6.85 (s, 1H), 6.80 (dd, J = 8.0, 2.0 Hz, 1H), 6.70 (d, J = 7.6 Hz, 1H), 5.95 (d, J = 2.4 Hz, 2H), 3.04 (t, J = 6.8 Hz, 2H), 2.82 (t, J = 7.0 Hz, 2H), 2.28 (s, 3H); ^{13}C NMR (100 MHz, DMSO-d_6): δ 198.8, 149.2, 147.2, 146.7, 145.9, 145.7, 145.5, 145.4, 142.8, 138.5, 137.6, 134.8, 134.7, 134.2, 132.5, 131.9, 130.3, 128.05, 127.95, 125.5, 121.5, 121.1, 108.8, 108.1, 100.7, 42.2, 28.9, 20.8; HRMS (ESI, positive) *m/z* calcd for $C_{23}H_{18}N_3O_7$ [M–$C_7H_7O_3S$]$^+$: 448.1139,

	found: 448.1134; m.p.: 204-207 °C.
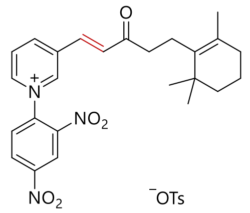<br><br>**1u**	**Compound 1u**<br>Reaction of **S1u** (0.29 g, 1.0 mmol) with 2,4-dinitrophenyltosylate (0.52 g, 1.5 mmol) in toluene (5 mL) gave compound **1u** (0.20 g, 31%, brown solid).<br>^{1}H NMR (400 MHz, DMSO-*d*$_6$): δ 9.76 (s, 1H), 9.37 (d, *J* = 6.0 Hz, 1H), 9.21 (d, *J* = 8.0 Hz, 1H), 9.14 (d, *J* = 2.4 Hz, 1H), 9.01 (dd, *J* = 8.8, 2.4 Hz, 1H), 8.48 (dd, *J* = 8.0, 6.4 Hz, 1H), 8.43 (d, *J* = 8.8 Hz, 1H), 7.76 (d, *J* = 16.4 Hz, 1H), 7.45 (d, *J* = 7.6 Hz, 2H), 7.26 (d, *J* = 16.4 Hz, 1H), 7.10 (d, *J* = 8.0 Hz, 2H), 2.79 (t, *J* = 8.0 Hz, 2H), 2.35-2.22 (m, 5H), 1.89 (t, *J* = 6.0 Hz, 2H), 1.62-1.51 (m, 5H), 1.45-1.37 (m, 2H), 0.98 (s, 6H); ^{13}C NMR (100 MHz, DMSO-*d*$_6$): δ 199.2, 149.3, 146.6, 145.9, 145.8, 145.6, 142.8, 138.5, 137.5, 136.0, 134.9, 133.9, 132.6, 131.9, 130.3, 128.0, 127.9, 127.3, 125.5, 121.5, 41.3, 34.6, 32.2, 28.3, 21.7, 20.8, 19.5, 19.0 (One peak is missing due to overlapping.); HRMS (ESI, positive) *m/z* calcd for $C_{25}H_{28}N_3O_5$ [M–$C_7H_7O_3S$]$^+$: 450.2023, found: 450.2018; m.p.: 110-113 °C.
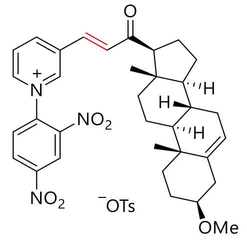<br><br>**1v**	**Compound 1v**<br>Reaction of **S1v** (0.23 g, 0.55 mmol) with 2,4-dinitrophenyltosylate (0.28 g, 0.83 mmol) in toluene (5 mL) gave compound **1v** (0.16 g, 39%, white solid).<br>^{1}H NMR (400 MHz, DMSO-*d*$_6$): δ 9.75 (s, 1H), 9.37 (d, *J* = 6.0 Hz, 1H), 9.24 (d, *J* = 8.0 Hz, 1H), 9.14 (d, *J* = 2.4 Hz, 1H), 9.01 (dd, *J* = 8.8, 2.4 Hz, 1H), 8.51-8.39 (m, 2H), 7.66 (dd, *J* = 14.8, 2.8 Hz, 1H), 7.45 (d, *J* = 8.0 Hz, 2H), 7.39-7.26 (m, 1H), 7.10 (d, *J* = 8.0 Hz, 2H), 5.38-5.30 (m, 1H), 3.25-3.20 (m, 3H), 3.05-2.91 (m, 2H), 2.28 (s, 3H), 2.06-1.22 (m, 17H), 1.03-0.91 (m, 5H), 0.55 (s, 3H); ^{13}C NMR (100 MHz, DMSO-*d*$_6$): δ 149.3, 146.6, 145.9, 145.8, 145.6, 142.9, 140.5, 138.5, 137.5, 134.8, 133.3, 132.0, 130.3, 129.5, 128.0, 127.9, 125.5, 121.5, 120.9, 119.6, 79.5, 60.8, 56.3, 54.9, 49.6, 44.7, 44.6, 38.3, 38.1, 36.6, 36.4, 31.5, 31.4, 31.3, 27.7, 24.2, 22.3, 20.8, 20.6, 20.0, 19.1, 13.4; HRMS (ESI, positive) *m/z* calcd for $C_{34}H_{40}N_3O_6$ [M–$C_7H_7O_3S$]$^+$: 586.2912, found: 586.2905; m.p.: 256-258 °C.
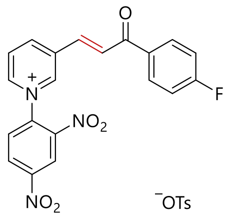<br><br>**1w**	**Compound 1w**<br>Reaction of 4-fluorophenyl 2-(3-pyridyl)vinyl ketone (1.1 g, 5.0 mmol) with 2,4-dinitrophenyltosylate (2.5 g, 7.5 mmol) in toluene (15 mL) gave compound **1w** (2.6 g, 92%, pale yellow solid).<br>^{1}H NMR (400 MHz, DMSO-*d*$_6$): δ 9.91 (s, 1H), 9.44-9.38 (m, 2H), 9.15 (d, *J* = 2.4 Hz, 1H), 9.02 (dd, *J* = 8.8, 2.4 Hz, 1H), 8.54 (dd, *J* = 8.0, 6.0 Hz, 1H), 8.48 (d, *J* = 8.8 Hz, 1H), 8.36 (d, *J* = 15.6 Hz, 1H), 8.28 (dd, *J* = 8.4, 5.6 Hz, 2H), 7.88 (d, *J* = 15.6 Hz, 1H), 7.50-7.42 (m, 4H), 7.09 (d, *J* = 7.6 Hz, 1H), 2.28 (s, 3H); ^{13}C NMR (100 MHz, DMSO-*d*$_6$): δ 187.0, 165.5 (d, *J* = 251.5 Hz), 149.3, 146.9, 146.1, 145.8, 142.8, 138.5, 137.6, 135.7, 134.9, 133.4, 131.9 (d, *J* = 9.5 Hz), 130.5, 130.3, 128.7,

	128.0, 125.5, 121.5, 116.2 (d, J = 22.9 Hz), 20.8; ^{19}F NMR (376 MHz, DMSO-d_6): δ –104.5 (s, 1F); HRMS (ESI, positive) m/z calcd for $C_{20}H_{13}FN_3O_5$ [M–$C_7H_7O_3S$]$^+$: 394.0834, found: 394.0834; m.p.: 292-294 °C.

4. Procedure of benzene ring formation via amine-catalyzed activation of pyridine rings

General procedure

A 40 mL pressure tube equipped with a magnetic stirring bar and a septum was dried under vacuum with heating. After the tube was cooled to room temperature, it was purged with argon gas. Pyridinium **1** (1 equiv.), THF solution (30 mL/1 mmol of **1**) of piperidine (20 mol%), K_2CO_3 (2.0 equiv.), and H_2O (100 equiv.) were added to the tube, and it was sealed by Teflon cap. The tube was heated at 120 ºC with magnetic stirring for 42 hours. The reaction mixture was cooled to room temperature and the tube was opened to air. After removal of the solvent under vacuum, the crude product was purified with flash chromatography or GPC to obtain the desired product **2**.

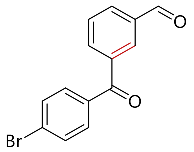<br>**2a**	**3-(4-bromobenzoyl)benzaldehyde (2a)**[17]<br>Reaction of **1a** (63 mg, 0.10 mmol) gave the title compound (22 mg, 77%, white solid).<br>Column solvent: Hexane/EtOAc = 100/0 to 4/1<br>^{1}H NMR (400 MHz, $CDCl_3$): δ 10.09 (s, 1H), 8.24 (s, 1H), 8.13 (dd, J = 7.6, 1.2 Hz, 1H), 8.05 (dd, J = 7.8, 1.0 Hz, 1H), 7.72-7.65 (m, 5H); ^{13}C NMR (100 MHz, $CDCl_3$): δ 194.6, 191.4, 138.2, 136.5, 135.6, 135.4, 133.2, 132.1, 131.6, 131.2, 129.5, 128.4.
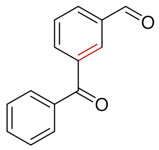<br>**2b**	**3-benzoylbenzaldehyde (2b)**[18]<br>Reaction of **1b** (55 mg, 0.10 mmol) gave the title compound (17 mg, 79%, yellow solid).<br>Column solvent: Hexane/EtOAc = 100/0 to 4/1<br>^{1}H NMR (400 MHz, $CDCl_3$): δ 10.10 (s, 1H), 8.28 (s, 1H), 8.12 (d, J = 7.6 Hz, 1H), 8.09 (d, J = 7.6 Hz, 1H), 7.81 (d, J = 7.6 Hz, 2H), 7.72-7.60 (m, 2H), 7.52 (t, J = 7.2 Hz, 2H); ^{13}C NMR (100 MHz, $CDCl_3$): δ 195.6, 191.6, 138.6, 137.0, 136.5, 135.6, 133.1, 132.8, 131.5, 130.2, 129.4, 128.7.
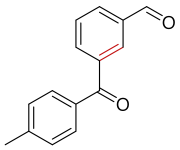<br>**2c**	**3-(4-methylbenzoyl)benzaldehyde (2c)**[19]<br>Reaction of **1c** (56 mg, 0.10 mmol) gave the title compound (18 mg, 82%, yellow solid).<br>Column solvent: Hexane/EtOAc = 100/0 to 4/1<br>^{1}H NMR (400 MHz, $CDCl_3$): δ 10.09 (s, 1H), 8.26 (s, 1H), 8.10 (d, J = 7.6 Hz,

[17] Y. Yamazaki, M. Sumikura, Y. Masuda, Y. Hayashi, H. Yasui, Y. Kiso, T. Chinen, T. Usui, F. Yakushiji, B. Potts, S. Neuteboom, M. Palladino, G. K. Lloyd and Y. Hayashi, *Bioorg. Med. Chem.*, 2012, **20**, 4279-4289.
[18] P. Miziak, J. Zoń, N. Amrhein and R. Gancarz, *Phytochemistry*, 2007, **68**, 407-415.
[19] Z. Zhang, M. G. Lindale and L. S. Liebeskind, *J. Am. Chem. Soc.*, 2011, **133**, 6403-6410.

	1H), 8.06 (d, *J* = 7.6 Hz, 1H), 7.72 (d, *J* = 7.2 Hz, 2H), 7.67 (t, *J* = 7.8 Hz, 1H), 7.31 (d, *J* = 7.6 Hz, 2H), 2.46 (s, 3H); ^{13}C NMR (100 MHz, $CDCl_3$): δ 195.4, 191.6, 144.1, 139.0, 136.4, 135.5, 134.3, 132.6, 131.4, 130.4, 129.4, 129.3, 21.9.
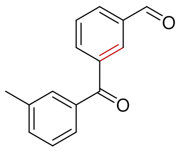<br>**2d**	**3-(3-methylbenzoyl)benzaldehyde (2d)**<br>Reaction of **1d** (57 mg, 0.10 mmol) gave the title compound (19 mg, 83%, yellow solid).<br>Column solvent: Hexane/EtOAc = 100/0 to 4/1<br>^{1}H NMR (400 MHz, $CDCl_3$): δ 10.09 (s, 1H), 8.27 (s, 1H), 8.12 (d, *J* = 7.6 Hz, 1H), 8.07 (d, *J* = 7.6 Hz, 1H), 7.68 (t, *J* = 7.6 Hz, 1H), 7.63 (s, 1H), 7.57 (d, *J* = 7.2 Hz, 1H), 7.48-7.36 (m, 2H). 2.44 (s, 3H); ^{13}C NMR (100 MHz, $CDCl_3$): δ 195.9, 191.6, 138.8, 138.7, 137.0, 136.5, 135.6, 133.9, 132.7, 131.5, 130.5, 129.4, 128.5, 127.5, 21.5; HRMS (ESI, positive) *m/z* calcd for $C_{15}H_{13}O_2$ $[M+H]^+$: 225.0910, found: 225.0904; m.p.: 47-50 °C.
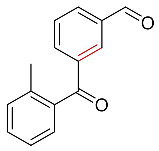<br>**2e**	**3-(2-methylbenzoyl)benzaldehyde (2e)**<br>Reaction of **1e** (60 mg, 0.11 mmol) gave the title compound (18 mg, 76%, yellow solid).<br>Column solvent: Hexane/EtOAc = 100/0 to 4/1<br>^{1}H NMR (400 MHz, $CDCl_3$): δ 10.06 (s, 1H), 8.26 (t, *J* = 1.6 Hz, 1H), 8.14-8.05 (m, 2H), 7.65 (t, *J* = 7.6 Hz, 1H), 7.43 (dt, *J* = 7.2, 1.6 Hz, 1H), 7.37-7.23 (m, 3H), 2.36 (s, 3H); ^{13}C NMR (100 MHz, $CDCl_3$): δ 197.4, 191.6, 138.9, 137.7, 137.3, 136.7, 135.6, 133.3, 131.7, 131.5, 131.0, 129.5, 128.9, 125.6, 20.2; HRMS (ESI, positive) *m/z* calcd for $C_{15}H_{13}O_2$ $[M+H]^+$: 225.0910, found: 225.0904; m.p.: 115-117 °C.
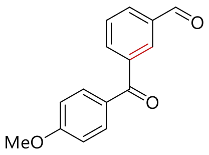<br>**2f**	**3-(4-methoxylbenzoyl)benzaldehyde (2f)[20]**<br>Reaction of **1f** (58 mg, 0.10 mmol) gave the title compound (18 mg, 76%, yellow solid).<br>Column solvent: Hexane/EtOAc = 100/0 to 4/1<br>^{1}H NMR (400 MHz, $CDCl_3$): δ 10.09 (s, 1H), 8.23 (s, 1H), 8.10 (d, *J* = 7.6 Hz, 1H), 8.04 (d, *J* = 7.2 Hz, 1H), 7.83 (d, *J* = 8.8 Hz, 2H), 7.67 (t, *J* = 7.6 Hz, 1H), 6.99 (d, *J* = 8.8 Hz, 2H), 3.91 (s, 3H); ^{13}C NMR (100 MHz, $CDCl_3$): δ 194.3, 191.7, 163.8, 139.3, 136.4, 135.3, 132.7, 132.4, 131.2, 129.5, 129.3, 114.0, 55.7.
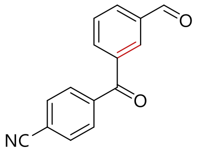<br>**2g**	**3-(4-cyanobenzoyl)benzaldehyde (2g)**<br>Reaction of **1g** (58 mg, 0.10 mmol) gave the title compound (23 mg, 95%, yellow solid).<br>Column solvent: Hexane/EtOAc = 100/0 to 4/1<br>^{1}H NMR (400 MHz, $CDCl_3$): δ 10.09 (s, 1H), 8.25 (s, 1H), 8.16 (d, *J* = 7.6 Hz,

[20] A. M. Echavarren and J. K. Stille, *J. Am. Chem. Soc.*, 1988, **110**, 1557-1565.

	1H), 8.07 (d, *J* = 7.6 Hz, 1H), 7.89 (d, *J* = 8.4 Hz, 2H), 7.83 (d, *J* = 8.4 Hz, 2H), 7.72 (t, *J* = 7.8 Hz, 1H); ^{13}C NMR (100 MHz, $CDCl_3$): δ 194.0, 191.2, 140.5, 137.3, 136.7, 135.4, 133.9, 132.6, 131.1, 130.3, 129.8, 117.9, 116.4; HRMS (ESI, positive) *m/z* calcd for $C_{15}H_{10}NO_2$ [M-$C_7H_7O_3S$]$^+$: 236.0706, found: 236.0711; m.p.: 168-171 °C.
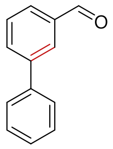<br>**2h**	**3-phenylbenzaldehyde (2h)**[21]<br>Reaction of **1h** (54 mg, 0.10 mmol) gave the title compound (16 mg, 82%, yellow oil).<br>Column solvent: Hexane/EtOAc = 100/0 to 4/1<br>^{1}H NMR (400 MHz, $CDCl_3$): δ 10.10 (s, 1H), 8.11 (t, *J* = 1.6 Hz, 1H), 7.87 (dd, *J* = 7.8, 1.8 Hz, 2H), 7.71-7.59 (m, 3H), 7.51-7.47 (m, 2H), 7.45-7.37 (m, 1H); ^{13}C NMR (100 MHz, $CDCl_3$): δ 192.5, 142.3, 139.8, 137.1, 133.2, 129.6, 129.1, 128.8, 128.3, 128.2, 127.3.
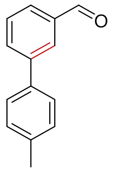<br>**2i**	**3-(4-methylphenyl)benzaldehyde (2i)**[22]<br>Reaction of **1i** (56 mg, 0.10 mmol) gave the title compound (15 mg, 69%, white solid).<br>Column solvent: Hexane/EtOAc = 100/0 to 4/1<br>^{1}H NMR (400 MHz, $CDCl_3$): δ 10.09 (s, 1H), 8.09 (s, 1H), 7.85 (t, *J* = 5.4 Hz, 2H), 7.60 (t, *J* = 7.6 Hz, 1H), 7.54 (d, *J* = 8.0 Hz, 2H), 7.29 (d, *J* = 7.6 Hz, 2H), 2.42 (s, 3H); ^{13}C NMR (100 MHz, $CDCl_3$): δ 192.6, 142.2, 138.1, 137.0, 136.9, 133.0, 129.9, 129.6, 128.5, 128.1, 127.1, 21.3.
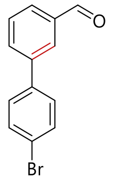<br>**2j**	**3-(4-bromophenyl)benzaldehyde (2j)**[23]<br>Reaction of **1j** (61 mg, 0.10 mmol) gave the title compound (17 mg, 63%, yellow solid).<br>Column solvent: Hexane/EtOAc = 100/0 to 4/1<br>^{1}H NMR (400 MHz, $CDCl_3$): δ 10.09 (s, 1H), 8.07 (s, 1H), 7.88 (d, *J* = 7.6 Hz, 1H), 7.82 (d, *J* = 7.6 Hz, 1H), 7.66-7.57 (m, 3H), 7.50 (d, *J* = 8.0 Hz, 2H); ^{13}C NMR (100 MHz, $CDCl_3$): δ 192.3, 141.1, 138.7, 137.1, 132.9, 132.3, 129.8, 129.3, 128.9, 127.9, 122.6.
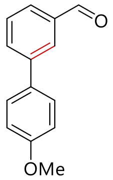<br>**2k**	**3-(4-methoxyphenyl)benzaldehyde (2k)**[24]<br>Reaction of **1k** (56 mg, 0.10 mmol) gave the title compound (14 mg, 62%, yellow solid).<br>Column solvent: Hexane/EtOAc = 100/0 to 4/1<br>^{1}H NMR (400 MHz, $CDCl_3$): δ 10.08 (s, 1H), 8.06 (s, 1H), 7.84-7.78 (m, 2H),

[21] B. Tao and D. W. Boykin, *J. Org. Chem.*, 2004, **69**, 4330-4335.
[22] B. Karimi, D. Elhamifar, J. H. Clark and A. J. Hunt, *Chem. Eur. J.*, 2010, **16**, 8047-8053.
[23] H. Bonin, D. Delbrayelle, P. Demonchaux and E. Gras, *Chem. Commun.*, 2010, **46**, 2677.
[24] C. A. Hunter, M. C. Misuraca and S. M. Turega, *J. Am. Chem. Soc.*, 2011, **133**, 582-594.

	7.62-7.55 (m, 3H), 7.01 (d, J = 8.4 Hz, 2H), 3.87 (s, 3H); ^{13}C NMR (100 MHz, $CDCl_3$): δ 192.6, 159.8, 141.9, 137.0, 132.7, 132.3, 129.6, 128.3, 128.2, 127.8, 114.6, 55.5.
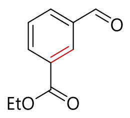<br>**2l**	**3-(ethoxycarbonyl)benzaldehyde (2l)**[25]<br>Reaction of **1l** (106 mg, 0.21 mmol) gave the title compound (22 mg, 60%, yellow oil).<br>Column solvent: Hexane/EtOAc = 100/0 to 4/1<br>^{1}H NMR (400 MHz, $CDCl_3$): δ 10.07 (s, 1H), 8.52 (t, J = 1.6 Hz, 1H), 8.30 (dt, J = 7.9, 1.4 Hz, 1H), 8.07 (dt, J = 7.6, 1.2 Hz, 1H), 7.62 (t, J = 7.6 Hz, 1H), 4.42 (q, J = 7.2 Hz, 2H), 1.42 (t, J = 7.2 Hz, 3H); ^{13}C NMR (100 MHz, $CDCl_3$): δ 191.6, 165.6, 136.6, 135.3, 133.1, 131.7, 131.3, 129.3, 61.6, 14.4.
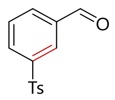<br>**2m**	**3-(toluenesulfonyl)benzaldehyde (2m)**<br>Reaction of **1m** (62 mg, 0.10 mmol) gave the title compound (18 mg, 66%, yellow solid).<br>Column solvent: Hexane/EtOAc = 100/0 to 4/1<br>^{1}H NMR (400 MHz, $CDCl_3$): δ 10.05 (s, 1H), 8.40 (s, 1H), 8.18 (d, J = 7.6 Hz, 1H), 8.06 (d, J = 7.6 Hz, 1H), 7.86 (d, J = 8.0 Hz, 2H), 7.68 (t, J = 7.6 Hz, 1H), 7.33 (d, J = 8.0 Hz, 2H), 2.41 (s, 3H); ^{13}C NMR (100 MHz, $CDCl_3$): δ 190.4, 145.0, 143.7, 137.9, 137.2, 133.4, 132.9, 130.33, 130.31, 128.9, 128.0, 21.7; HRMS (ESI, positive) m/z calcd for $C_{14}H_{13}O_3S$ $[M+H]^+$: 261.0580, found: 261.0580; m.p.: 109-111 °C.
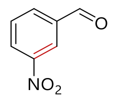<br>**2n**	**3-nitrobenzaldehyde (2n)**[26]<br>Reaction of **1n** (97 mg, 0.20 mmol) gave the title compound (15 mg, 51%, yellow solid).<br>Column solvent: Hexane/EtOAc = 100/0 to 4/1<br>^{1}H NMR (400 MHz, $CDCl_3$): δ 10.12 (s, 1H), 8.71 (s, 1H), 8.49 (d, J = 7.2 Hz, 1H), 8.23 (d, J = 7.6 Hz, 1H), 7.77 (t, J = 7.8 Hz, 1H); ^{13}C NMR (100 MHz, $CDCl_3$): δ 189.9, 148.9, 137.5, 134.8, 130.5, 128.7, 124.6.
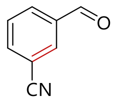<br>**2o**	**3-cyanobenzaldehyde (2o)**[27]<br>Reaction of **1o** (94 mg, 0.20 mmol) gave the title compound (12 mg, 47%, yellow solid).<br>Column solvent: Hexane/EtOAc = 100/0 to 4/1<br>^{1}H NMR (400 MHz, $CDCl_3$): δ 10.05 (s, 1H), 8.18 (s, 1H), 8.13 (dt, J = 8.0, 1.4 Hz, 1H), 7.92 (dt, J = 7.6, 1.4 Hz, 1H), 7.70 (t, J = 7.6 Hz, 1H); ^{13}C NMR (100 MHz, $CDCl_3$): δ 190.1, 137.4, 137.0, 133.4, 133.3, 130.2, 117.7, 113.9.

[25] Y. Lin, L. Zhu, Y. Lan and Y. Rao, *Chem. Eur. J.*, 2015, **21**, 14937-14942.
[26] S. Wertz and A. *Studer, Adv. Synth. Catal.*, 2011, **353**, 69-72.
[27] B. T. Gregg, K. C. Golden and J. F. Quinn, *J. Org. Chem.*, 2007, **72**, 5890-5893.

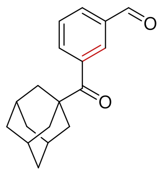 **2p**	**3-(1-adamantylcarbonyl)benzaldehyde (2p)**[28] Reaction of **1p** (60 mg, 0.10 mmol) gave the title compound (19 mg, 73%, yellow oil). Column solvent: Hexane/EtOAc = 100/0 to 3/1 ^{1}H NMR (400 MHz, $CDCl_3$): δ 10.05 (d, *J* = 1.2 Hz, 1H), 8.03 (d, *J* = 1.2 Hz, 1H), 7.95 (dd, *J* = 7.6, 1.2 Hz, 1H), 7.78 (dd, *J* = 7.6, 1.2 Hz, 1H), 7.58 (t, *J* = 7.6 Hz, 1H), 2.12-1.98 (m, 9H), 1.82-1.68 (m, 6H); ^{13}C NMR (100 MHz, $CDCl_3$): δ 209.2, 191.8, 140.5, 136.1, 133.0, 131.2, 129.0, 128.3, 47.2, 39.1, 38.4, 36.5, 28.1.
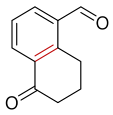 **2q**	**5-formyltetralone (2q)**[29] Reaction of **1q** (52 mg, 0.10 mmol) gave the title compound (13 mg, 72%, yellow solid). The reaction was carried out at 40 °C for 16 hours. Column solvent: Hexane/EtOAc = 100/0 to 4/1 ^{1}H NMR (400 MHz, $CDCl_3$): δ 10.30 (s, 1H), 8.33 (d, *J* = 7.6 Hz, 1H), 8.01 (d, *J* = 7.2 Hz, 1H), 7.52 (t, *J* = 7.6 Hz, 1H), 3.45 (t, *J* = 5.8 Hz, 2H), 2.71 (t, *J* = 6.4 Hz, 2H), 2.19 (quint, *J* = 6.2 Hz, 2H); ^{13}C NMR (100 MHz, $CDCl_3$): δ 197.5, 192.2, 146.2, 137.9, 134.1, 134.0, 133.0, 126.9, 38.7, 26.1, 22.6.
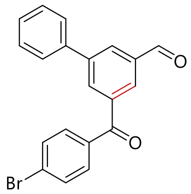 **2r**	**3-(4-bromobenzoyl)-5-phenylbenzaldehyde (2r)** Reaction of **1r** (70 mg, 0.10 mmol) gave the title compound (29 mg, 79%, yellow oil). Column solvent: Hexane/EtOAc = 100/0 to 3/1 ^{1}H NMR (400 MHz, $CDCl_3$): δ 10.14 (s, 1H), 8.33 (t, *J* = 1.8 Hz, 1H), 8.26 (t, *J* = 1.6 Hz, 1H), 8.19 (t, *J* = 1.4 Hz, 1H), 7.76-7.62 (m, 6H), 7.48 (t, *J* = 7.6 Hz, 2H), 7.44 (t, *J* = 8.0 Hz, 1H); ^{13}C NMR (100 MHz, $CDCl_3$): δ 194.6, 191.4, 142.9, 138.8, 138.7, 137.1, 135.7, 133.8, 132.1, 131.6, 131.4, 129.9, 129.3, 128.8, 128.4, 127.3; HRMS (ESI, positive) *m/z* calcd for $C_{20}H_{14}BrO_2$ $[M+H]^+$: 365.0172, found: 365.0176.
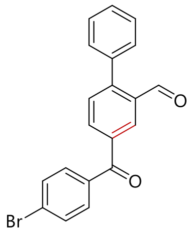 **2s**	**3-(4-bromobenzoyl)-6-phenylbenzaldehyde (2s)** Reaction of **1s** (70 mg, 0.10 mmol) gave the title compound (35 mg, 97%, yellow solid). Column solvent: Hexane/EtOAc = 100/0 to 3/1 ^{1}H NMR (400 MHz, $CDCl_3$): δ 10.02 (s, 1H), 8.35 (d, *J* = 1.6 Hz, 1H), 8.11 (dd, *J* = 8.0, 1.6 Hz, 1H), 7.72 (d, *J* = 8.8 Hz, 2H), 7.67 (d, *J* = 8.8 Hz, 2H), 7.63 (d, *J* = 8.0 Hz, 1H), 7.54-7.49 (m, 3H), 7.46-7.41 (m, 2H); ^{13}C NMR (100 MHz, $CDCl_3$): δ 194.5, 191.6, 149.6, 136.8, 136.7, 135.8, 134.3, 133.6, 132.1, 131.6, 131.5, 130.1, 129.5, 129.1, 128.9, 128.3; HRMS (ESI, positive) *m/z* calcd for $C_{20}H_{14}BrO_2$

[28] Y. Lin, L. Zhu, Y. Lan and Y. Rao, *Chem. Eur. J.*, 2015, **21**, 14937.
[29] Y. Ning, T. Fukuda, H. Ikeda, Y. Otani, M. Kawahata, K. Yamaguchi and T. Ohwada, *Org. Biomol. Chem.*, 2017, **15**, 1381-1392.

	$[M+H]^+$: 365.0172, found: 365.0175; m.p.: 168-170 °C.
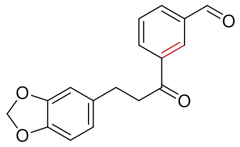<br>**2t**	**Compound 2t**<br>Reaction of **1t** (62 mg, 0.10 mmol) gave compound **2t** (22 mg, 78%, yellow oil).<br>Column solvent: Hexane/EtOAc = 100/0 to 3/1<br>^{1}H NMR (400 MHz, $CDCl_3$): δ 10.08 (s, 1H), 8.42 (t, J = 1.6 Hz, 1H), 8.22 (dt, J = 7.9, 1.5 Hz, 1H), 8.08 (dt, J = 7.6, 1.4 Hz, 1H), 7.65 (t, J = 7.8 Hz, 1H), 6.79-6.62 (m, 3H), 5.93 (s, 2H), 3.32 (t, J = 7.6 Hz, 2H), 3.02 (t, J = 7.6 Hz, 2H); ^{13}C NMR (100 MHz, $CDCl_3$): δ 198.2, 191.6, 147.8, 146.1, 137.7, 136.8, 134.8, 133.7, 133.6, 129.7, 129.4, 121.3, 109.1, 108.5, 101.0, 41.0, 29.8; HRMS (ESI, positive) *m/z* calcd for $C_{17}H_{15}O_4$ $[M+H]^+$: 283.0965, found: 283.0969.
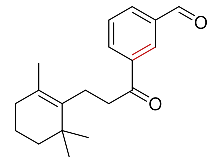<br>**2u**	**Compound 2u**<br>Reaction of **1u** (60 mg, 0.096 mmol) gave compound **2u**(19 mg, 68%, yellow oil).<br>Column solvent: Hexane/EtOAc = 100/0 to 3/1<br>^{1}H NMR (400 MHz, $CDCl_3$): δ 10.10 (s, 1H), 8.45 (s, 1H), 8.25 (d, J = 7.8 Hz, 1H), 8.08 (d, J = 7.6 Hz, 1H), 7.66 (t, J = 7.8 Hz, 1H), 3.14-3.05 (m, 2H), 2.51-2.39 (m, 2H), 1.94 (t, J = 6.2 Hz, 2H), 1.68-1.54 (m, 5H), 1.50-1.42 (m, 2H), 1.03 (s, 6H); ^{13}C NMR (100 MHz, $CDCl_3$): δ 199.3, 191.7, 137.8, 136.8, 136.1, 133.7, 133.6, 129.6, 129.3, 128.4, 39.9, 39.7, 35.2, 32.9, 28.6, 22.6, 20.0, 19.6; HRMS (ESI, positive) *m/z* calcd for $C_{19}H_{25}O_2$ $[M+H]^+$: 285.1849, found: 285.1861.
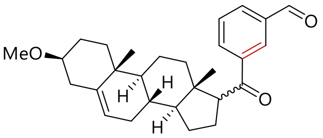<br>**2v**	**Compound 2v**<br>Reaction of **1v** (77 mg, 0.10 mmol) gave compound **2v** (34 mg, 80%, yellow solid) as a mixture of two diastereomers (2.2/1).<br>Column solvent: Hexane/EtOAc = 100/0 to 3/1<br>^{1}H NMR (400 MHz, $CDCl_3$): δ 10.10 (s, 1H minor), 10.09 (s, 1H, major), 8.43 (s, 1H, minor), 8.32 (t, J = 1.4 Hz, 1H, major), 8.21 (dt, J = 7.9, 1.5 Hz, 1H, minor), 8.15 (dt, J = 7.9, 1.4 Hz, 1H, major), 8.09-8.02 (m, 1H), 7.69-7.59 (m, 1H), 5.39-5.32 (m, 1H), 3.80-3.73 (m, 1H, minor), 3.54 (t, J = 8.6 Hz, 1H, major), 3.34 (s, 3H, major), 3.33 (s, 3H, minor), 3.09-2.95 (m, 1H), 2.50-2.31 (m, 2H), 2.18-1.99 (m, 2H), 1.98-1.72 (m, 5H), 1.58-1.28 (m, 9H), 1.11-0.81 (m, 4H), 0.63 (s, 3H); ^{13}C NMR (100 MHz, $CDCl_3$): δ 202.9, 201.1, 191.8, 141.0, 140.7, 140.1, 139.5, 136.7, 136.5, 134.0, 133.4, 133.3, 129.7, 129.6, 129.5, 121.6, 121.4, 80.3, 57.7, 57.6, 55.8, 55.2, 51.2, 50.1, 49.6, 46.7, 45.3, 39.4, 38.8, 37.3, 37.2, 37.0, 36.9, 36.1, 32.2, 32.0, 28.1, 26.3, 25.1, 24.9, 24.0, 21.2, 21.1, 20.6, 19.5, 13.7; HRMS (ESI, positive) *m/z* calcd for $C_{28}H_{37}O_3$ $[M+H]^+$: 421.2737, found: 421.2748; m.p.: 107-110 °C.

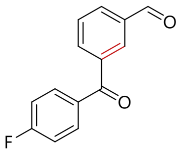<br>**2w**	**3-(4-fluorobenzoyl)benzaldehyde (2w)**[7]<br>Reaction of **1w** (57 mg, 0.10 mmol) gave the title compound (21 mg, 90%, yellow solid).<br>Column solvent: Hexane/EtOAc = 100/0 to 4/1<br>^{1}H NMR (400 MHz, $CDCl_3$): δ 10.10 (s, 1H), 8.25 (s, 1H), 8.12 (d, *J* = 7.6 Hz, 1H), 8.05 (d, *J* = 7.6 Hz, 1H), 7.85 (t, *J* = 6.8 Hz, 2H), 7.69 (t, *J* = 7.6 Hz, 1H), 7.20 (t, *J* = 8.4 Hz, 2H); ^{13}C NMR (100 MHz, $CDCl_3$): δ 194.1, 191.5, 165.8 (d, *J* = 253.7 Hz), 138.5, 136.5, 135.4, 133.2 (d, *J* = 3.0 Hz), 133.0, 132.8 (d, *J* = 9.2 Hz), 131.1, 129.5, 116.0 (d, *J* = 21.8 Hz); ^{19}F NMR (376 MHz, $CDCl_3$): δ –104.7 (s, 1F).

5. Unsuccessful substrates

These pyridiniums could not be synthesized because *N*-arylation did not occur.

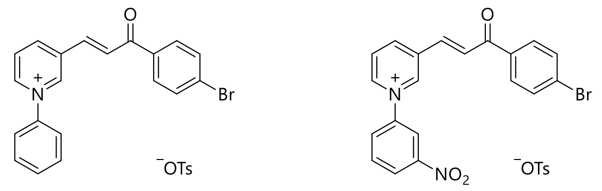

These pyridiniums did not afford the desired benzene derivatives under the optimized reaction conditions.

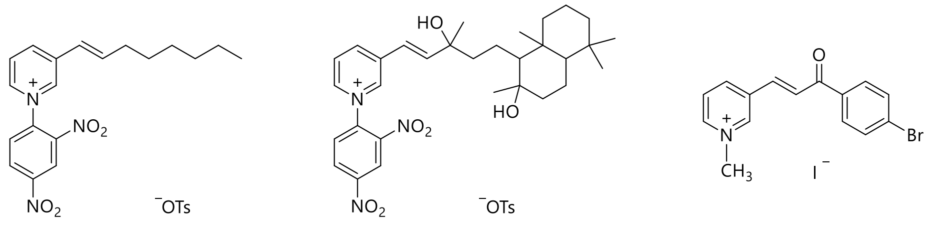

5. NMR and HRMS spectra

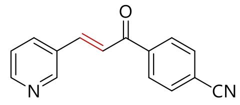

S1g

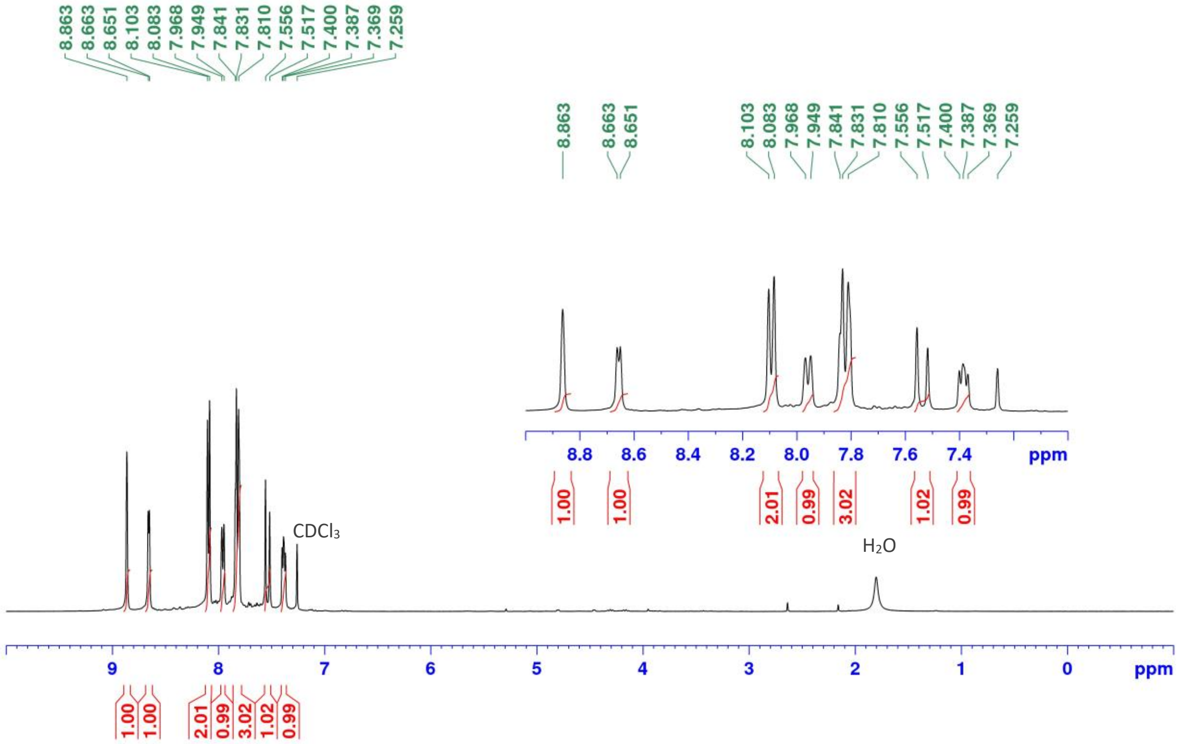

Figure S1. ^1H NMR (400 MHz) spectrum of **S1g** in $CDCl_3$.

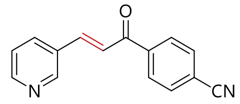

S1g

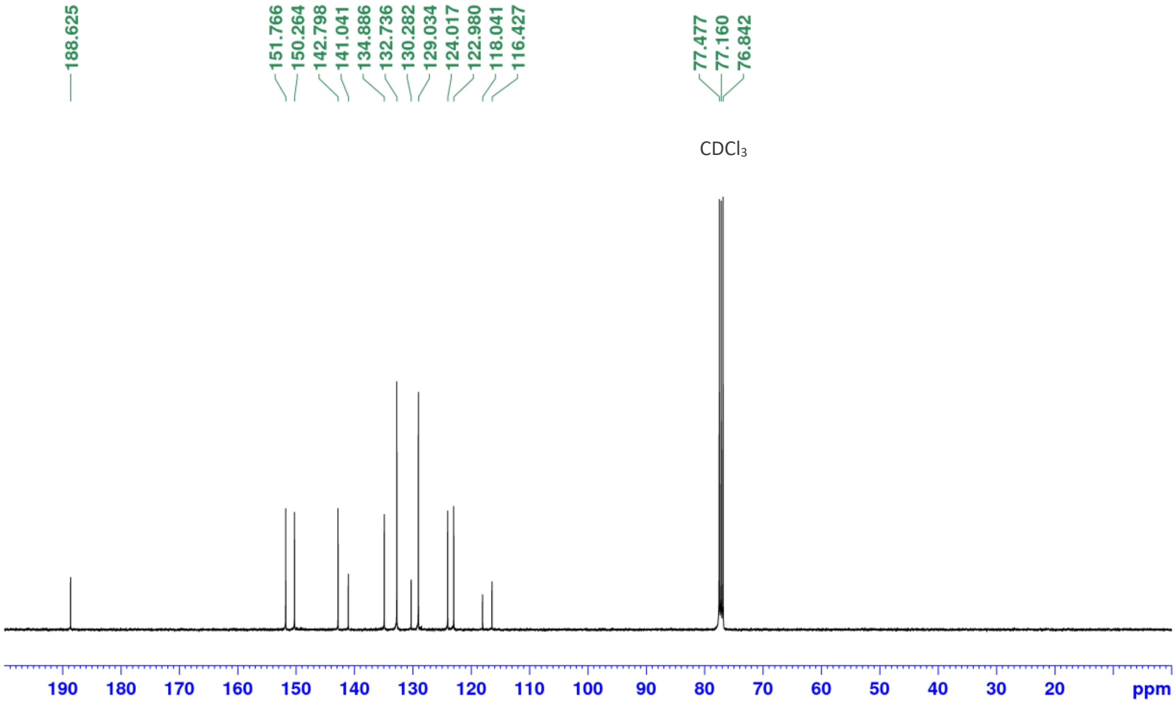

Figure S2. ^{13}C NMR (100 MHz) spectrum of **S1g** in $CDCl_3$.

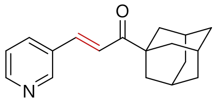

S1p

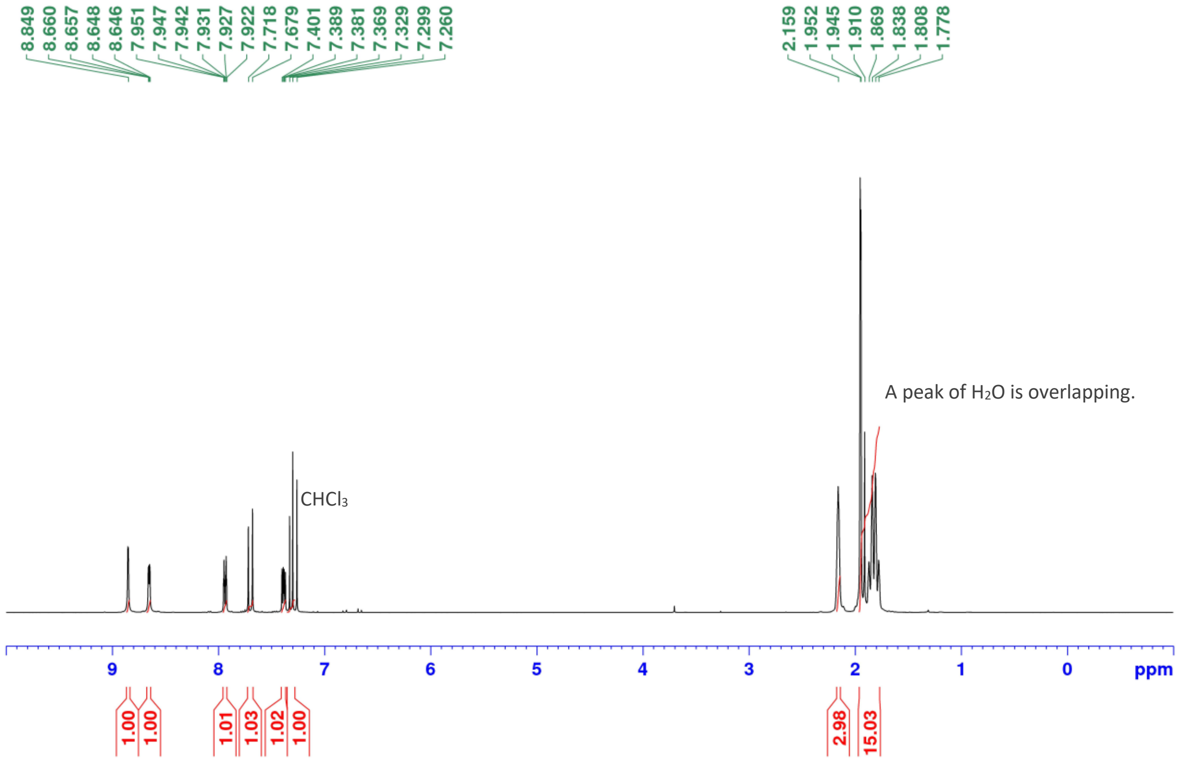

Figure S3. ^{1}H NMR (400 MHz) spectrum of **S1p** in $CDCl_3$.

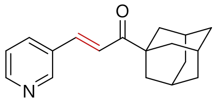

S1p

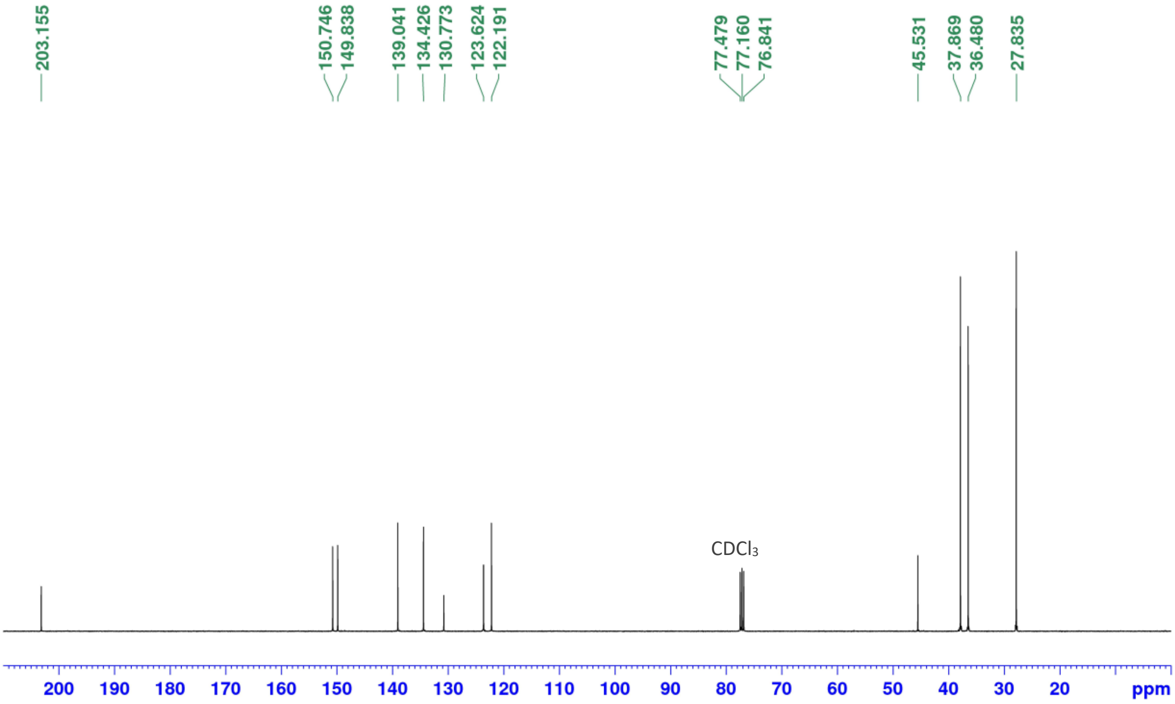

Figure S4. ^{13}C NMR (100 MHz) spectrum of **S1p** in $CDCl_3$.

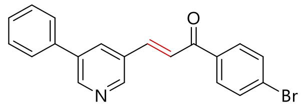

S1r

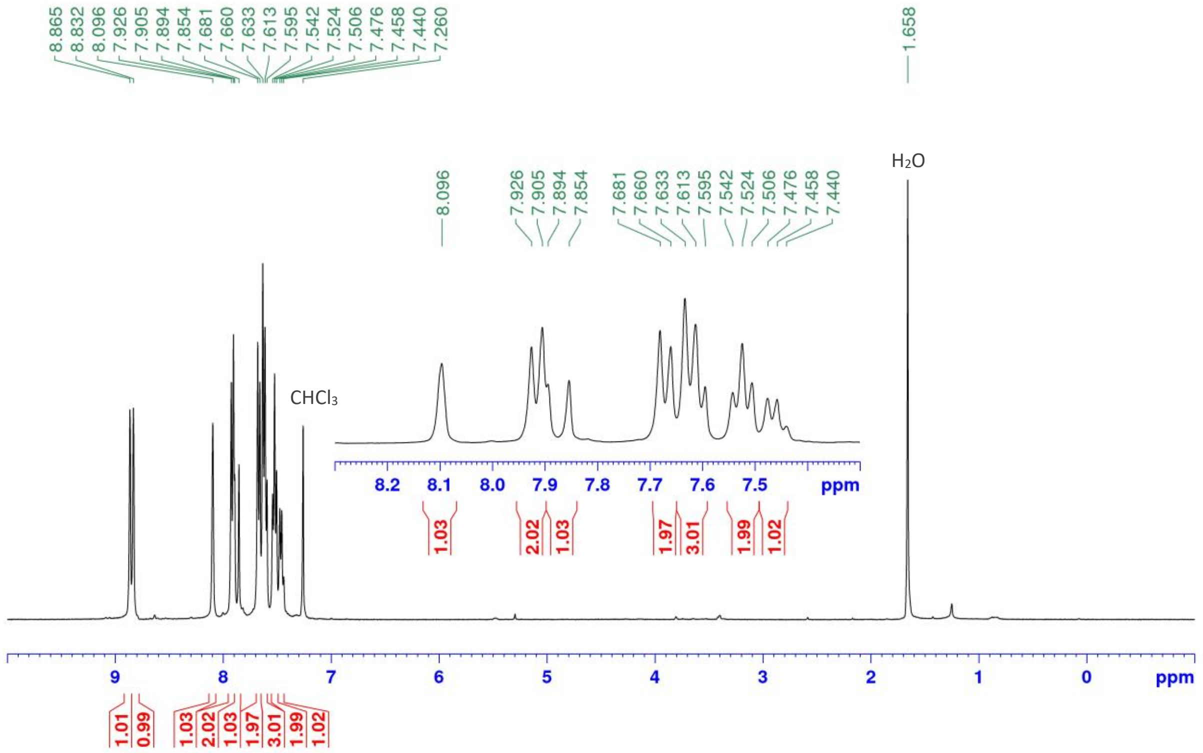

Figure S5. ^{1}H NMR (400 MHz) spectrum of **S1r** in $CDCl_3$.

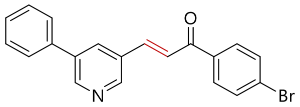

S1r

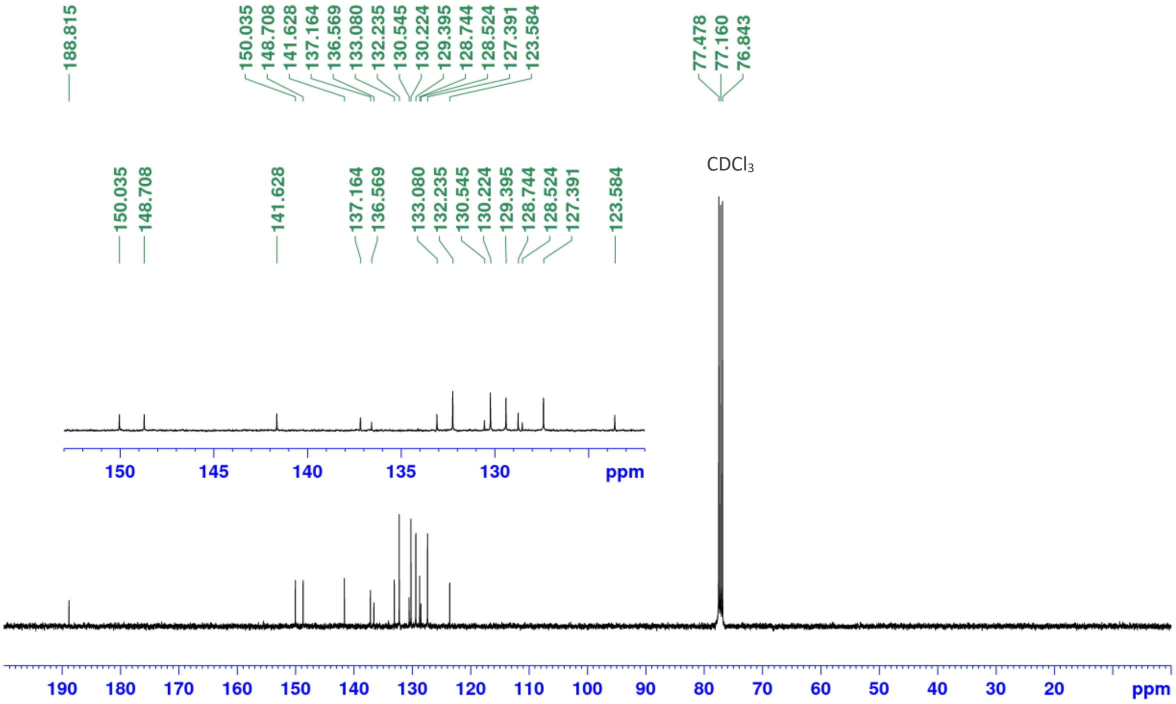

Figure S6. ^{13}C NMR (100 MHz) spectrum of **S1r** in $CDCl_3$.

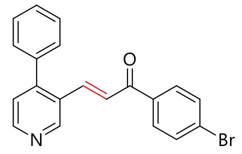

S1s

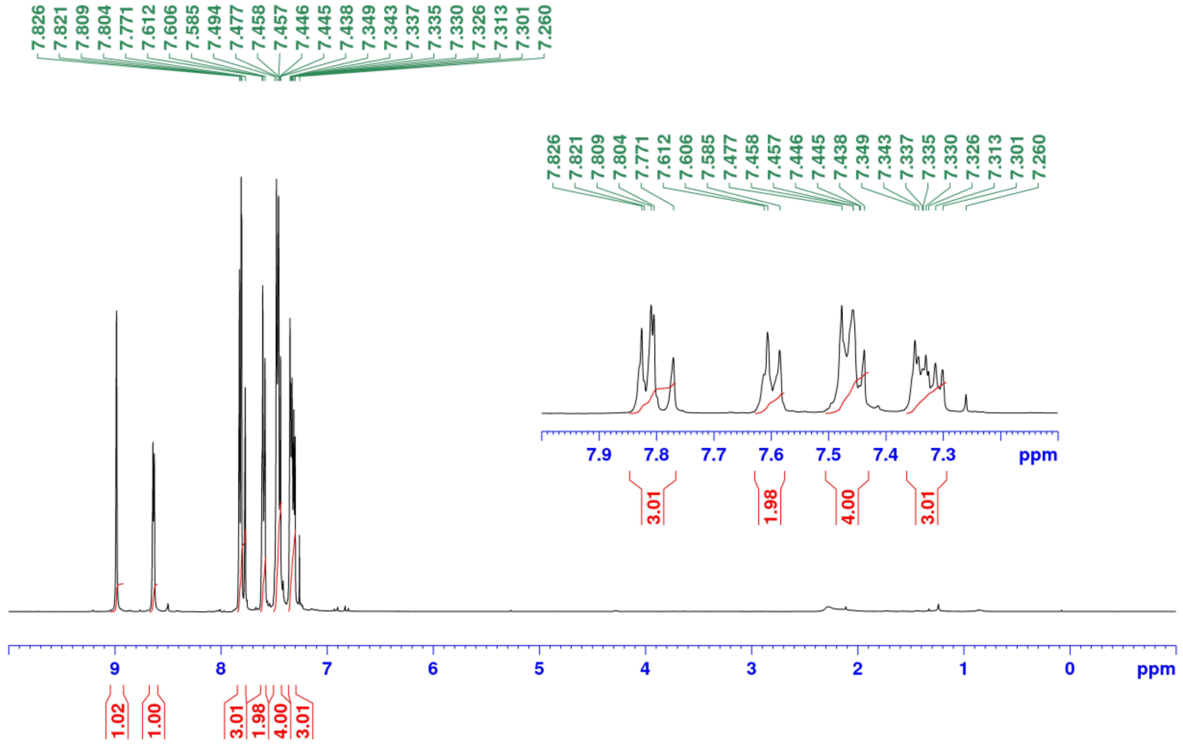

Figure S7. ^{1}H NMR (400 MHz) spectrum of **S1s** in $CDCl_3$.

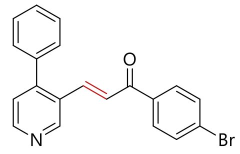

S1s

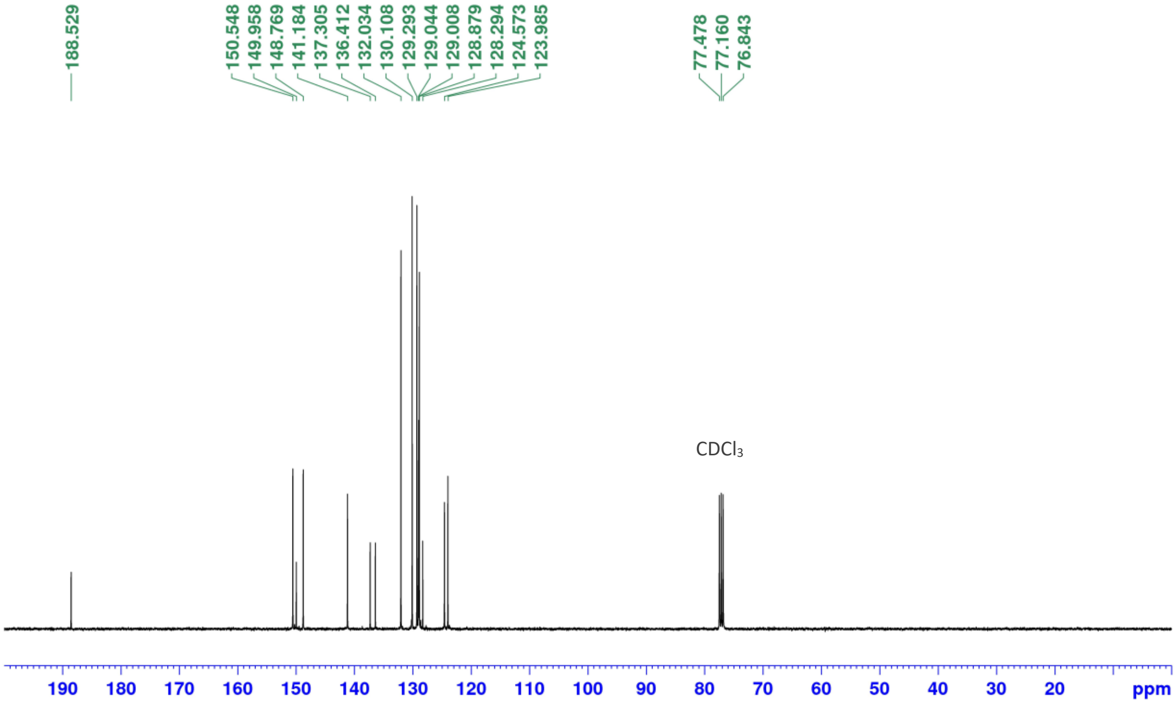

Figure S8. ^{13}C NMR (100 MHz) spectrum of **S1s** in $CDCl_3$.

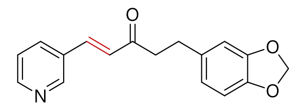

S1t

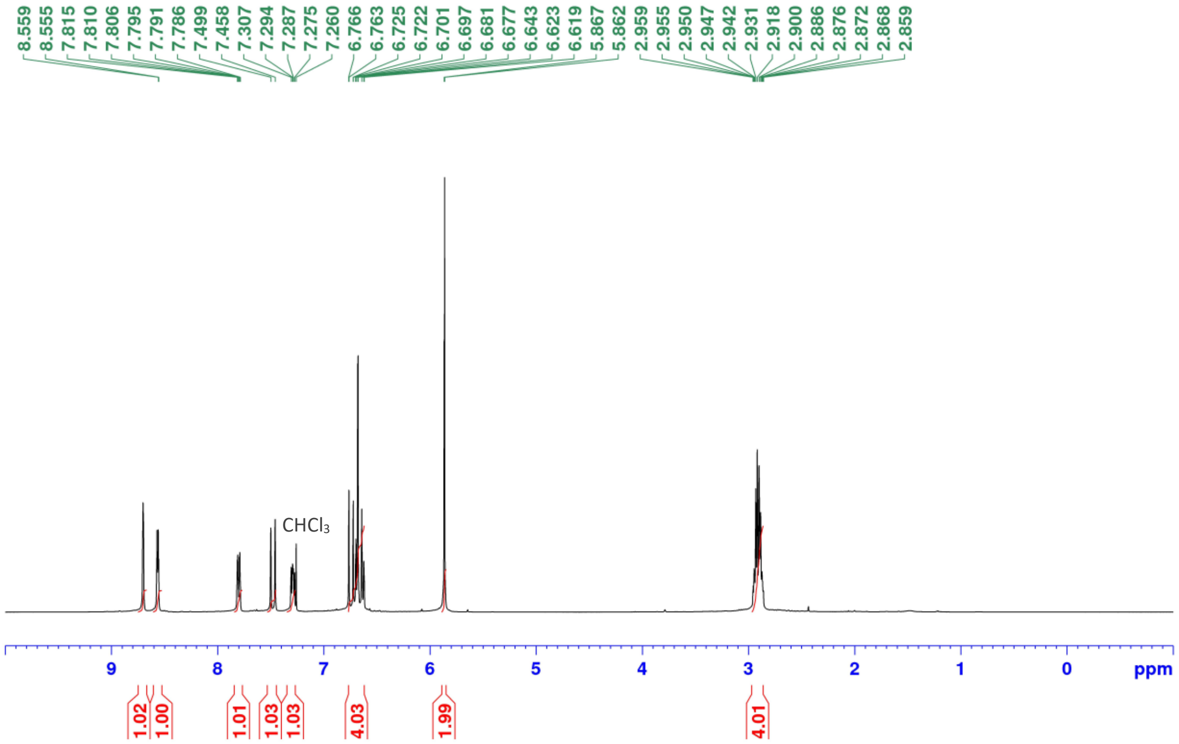

Figure S9. ^{1}H NMR (400 MHz) spectrum of **S1t** in $CDCl_3$.

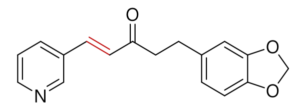

S1t

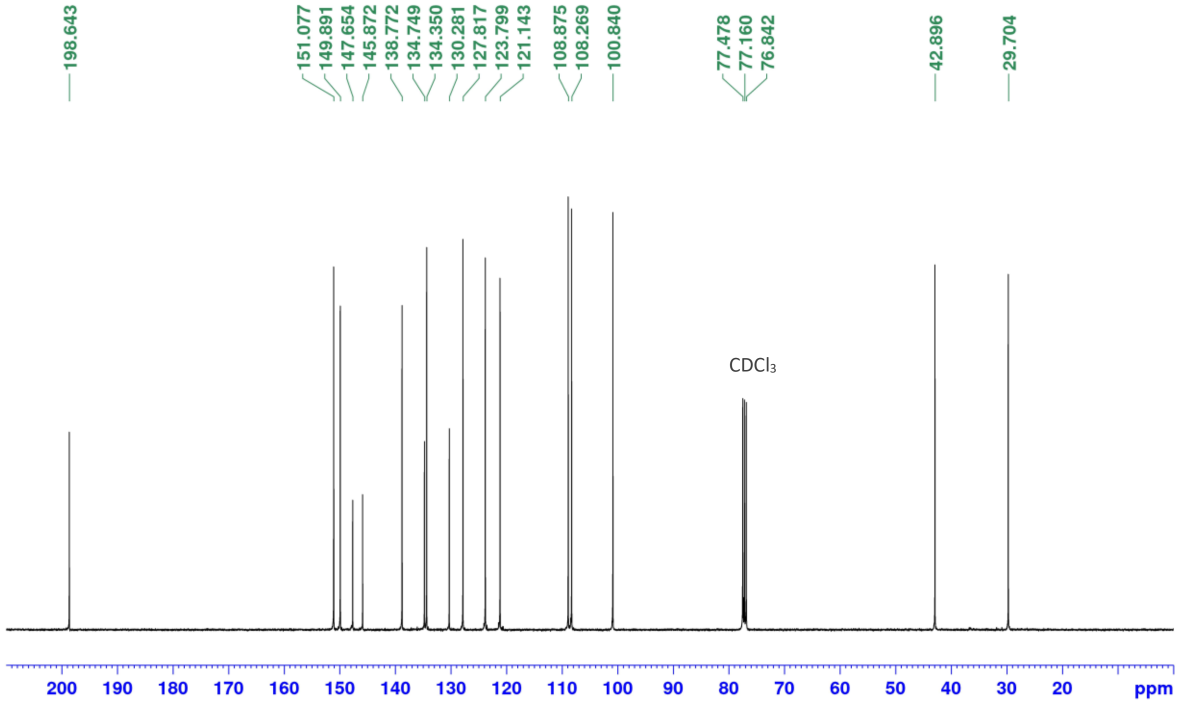

Figure S10. ^{13}C NMR (100 MHz) spectrum of **S1t** in $CDCl_3$.

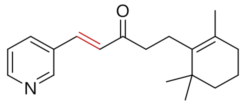

S1u

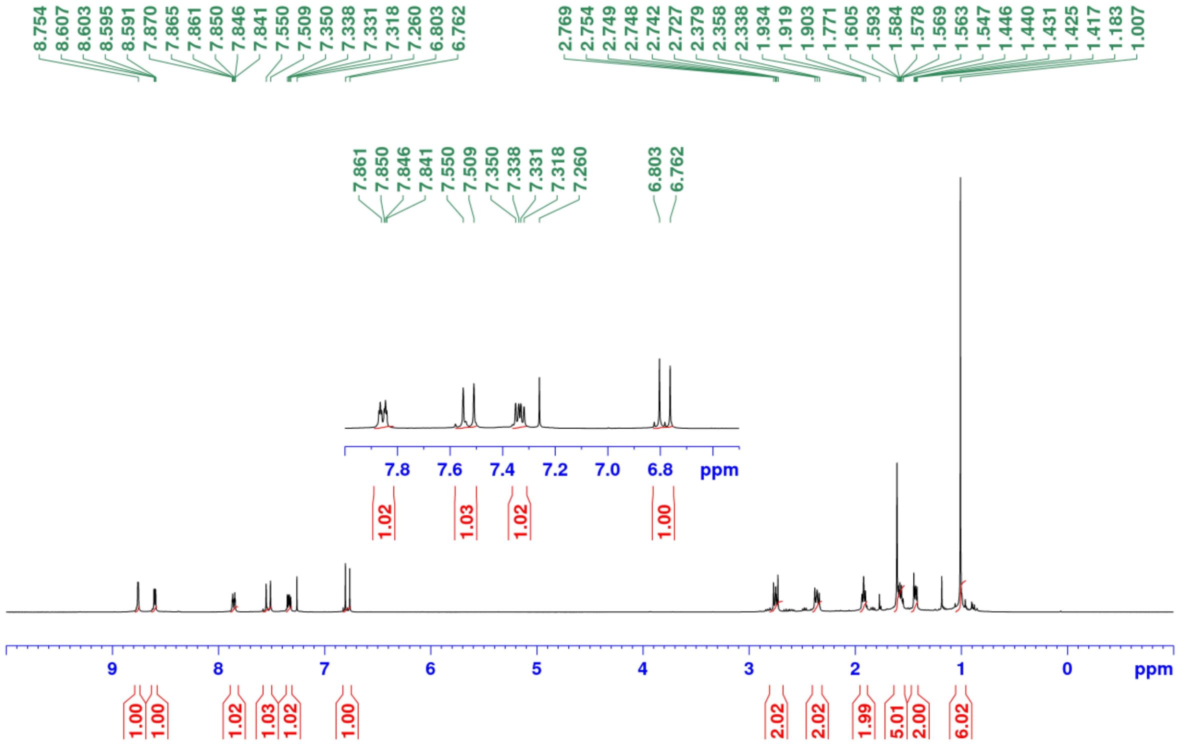

Figure S11. ^1H NMR (400 MHz) spectrum of **S1u** in $CDCl_3$.

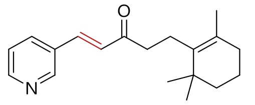

S1u

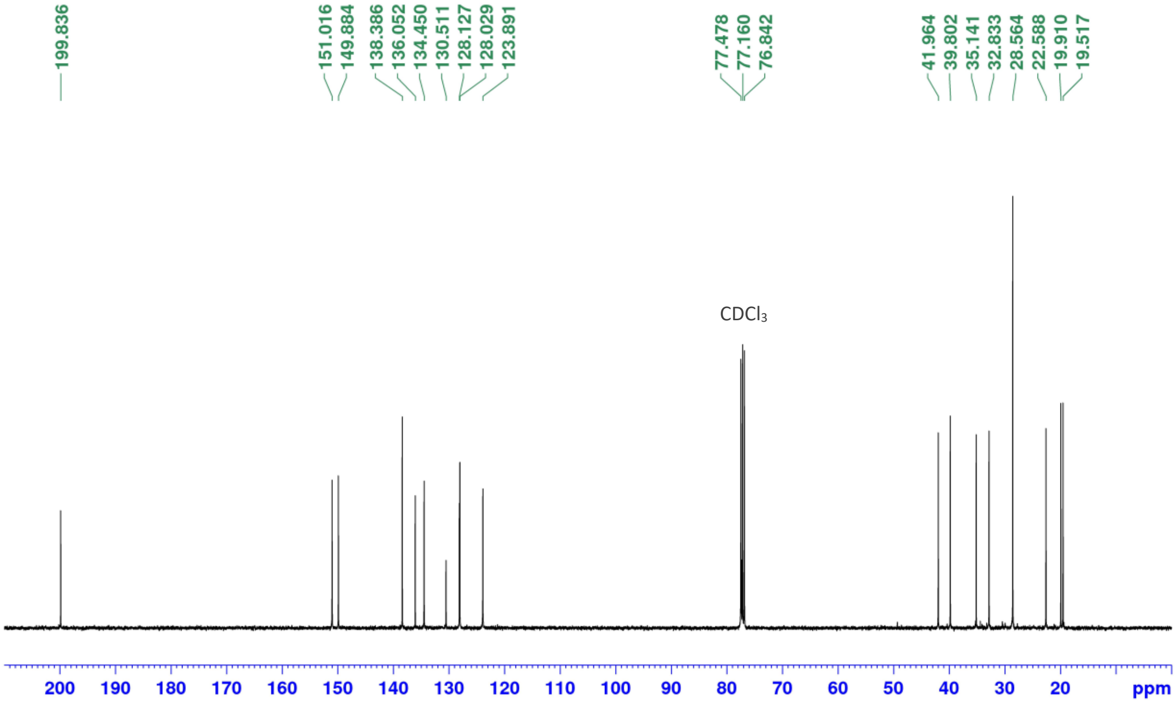

Figure S12. ^{13}C NMR (100 MHz) spectrum of **S1u** in $CDCl_3$.

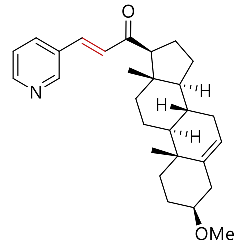

S1v

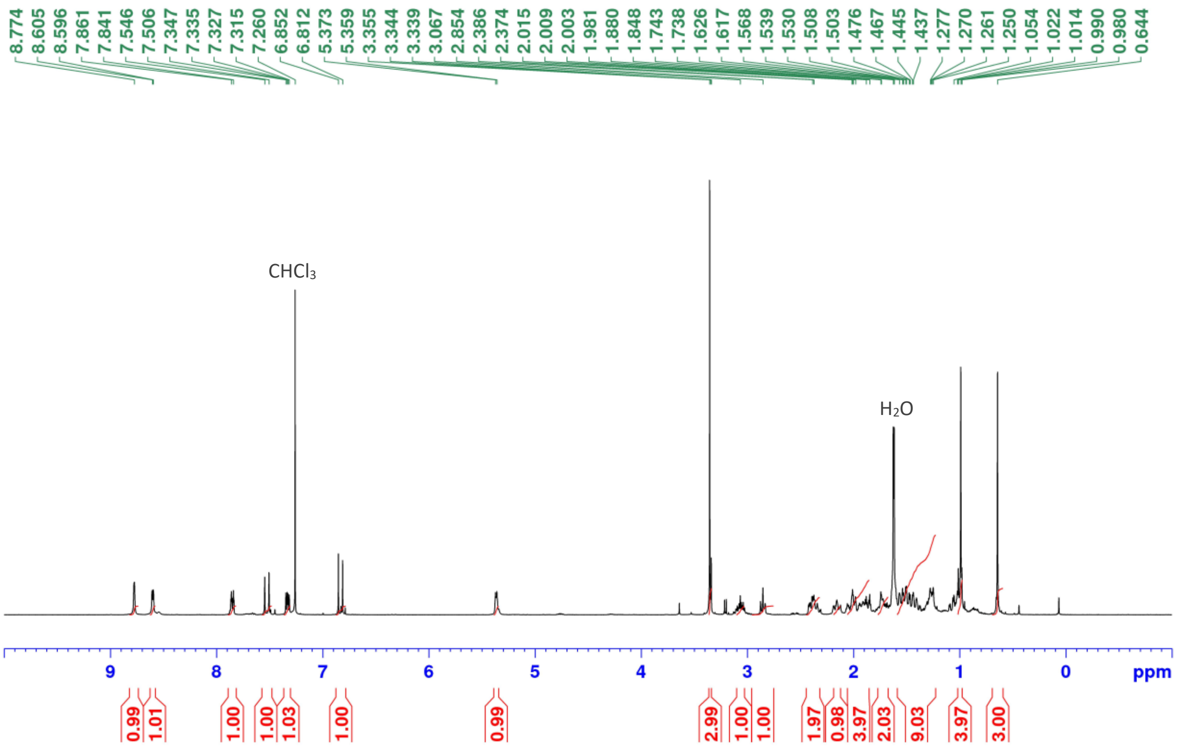

Figure S13. ^{1}H NMR (400 MHz) spectrum of **S1v** in $CDCl_3$.

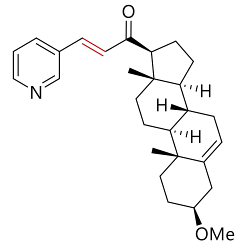

S1v

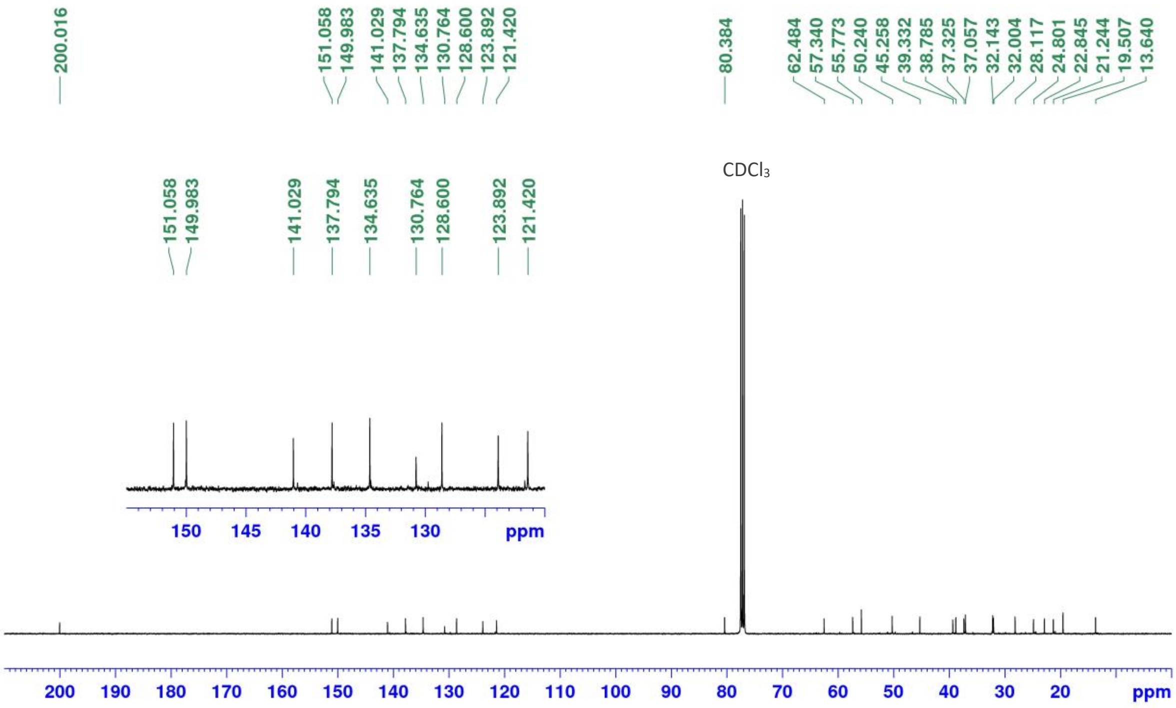

Figure S14. ^{13}C NMR (100 MHz) spectrum of **S1v** in $CDCl_3$.

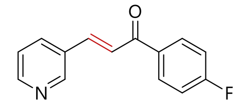

5

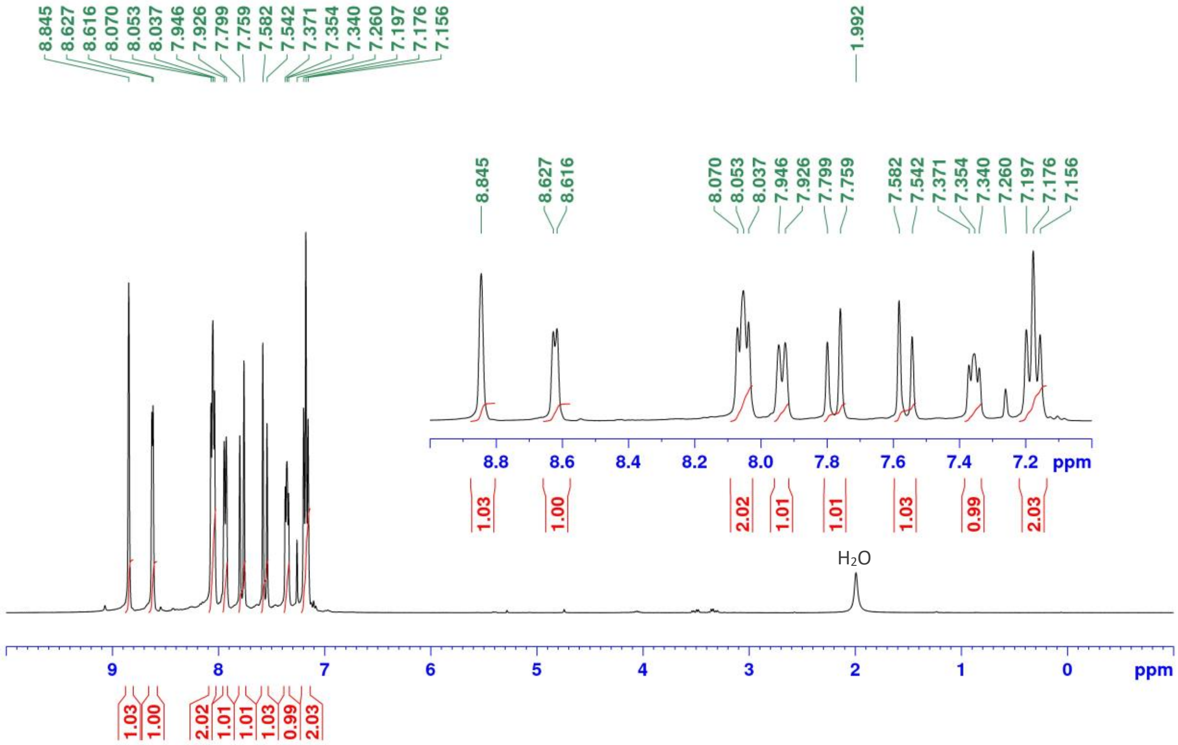

Figure S15. ^{1}H NMR (400 MHz) spectrum of **5** in $CDCl_3$.

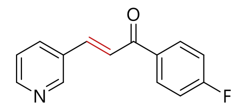

5

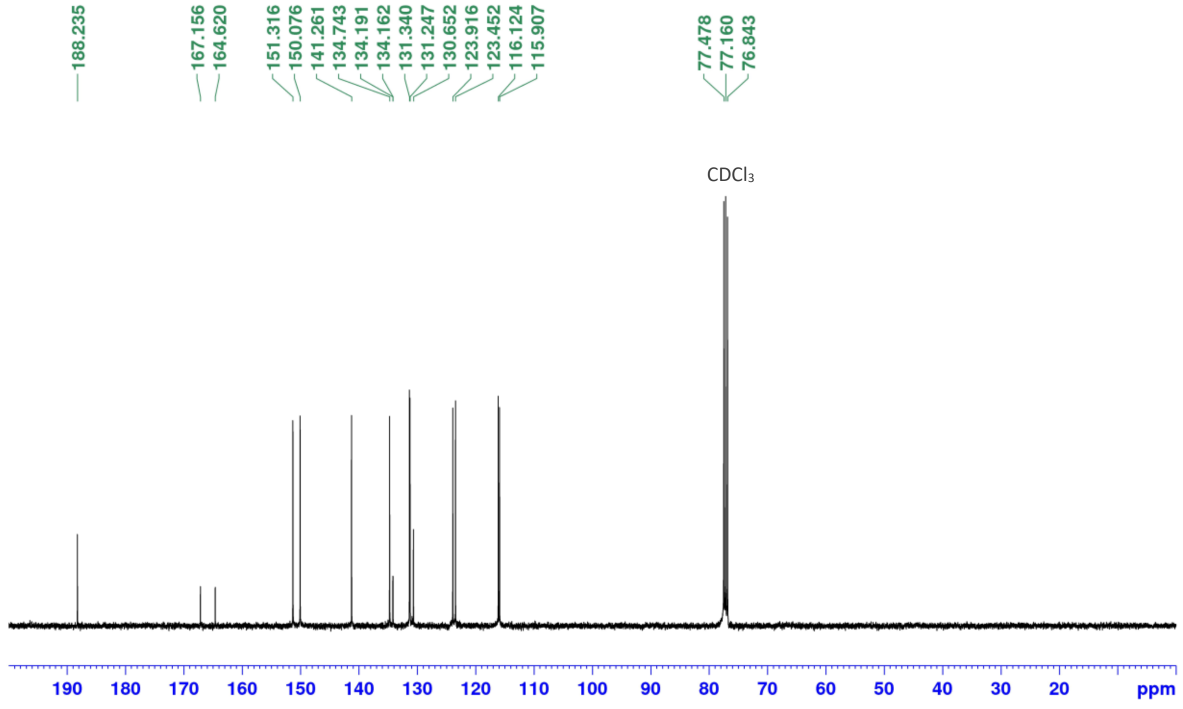

Figure S16. ^{13}C NMR (100 MHz) spectrum of **5** in $CDCl_3$.

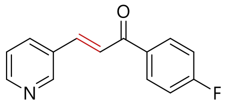

5

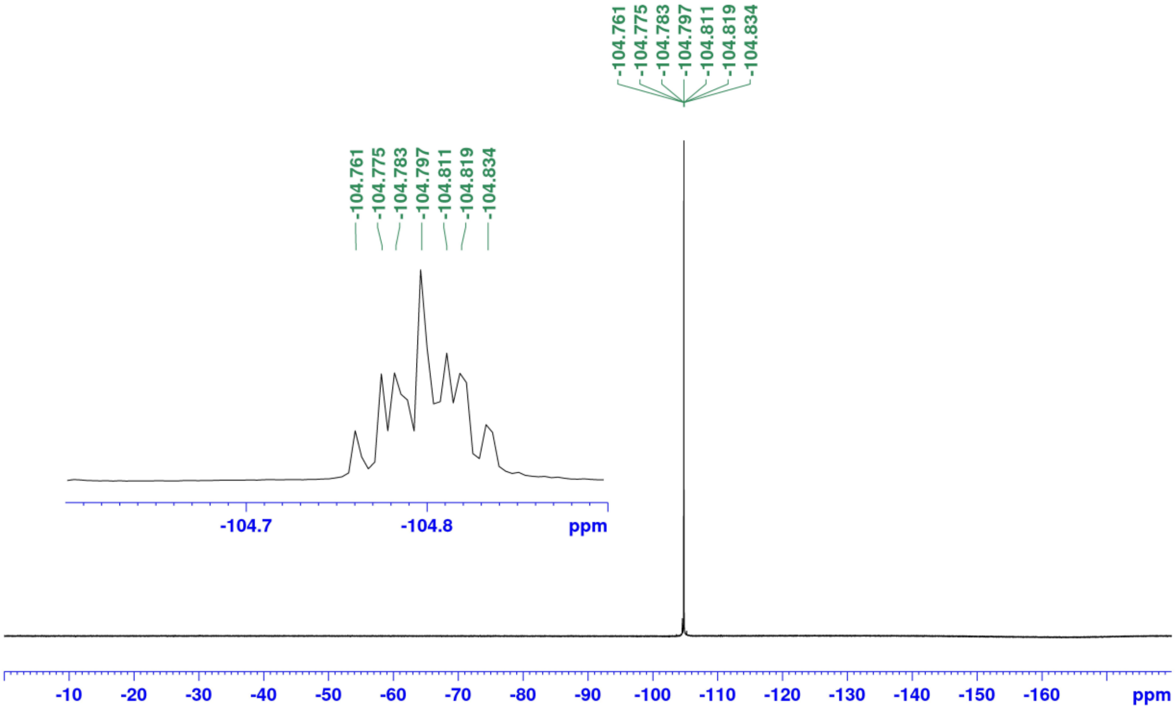

Figure S17. ^{19}F NMR (376 MHz) spectrum of **5** in $CDCl_3$.

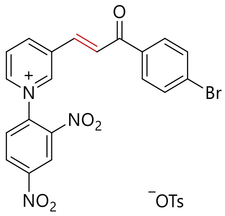

1a

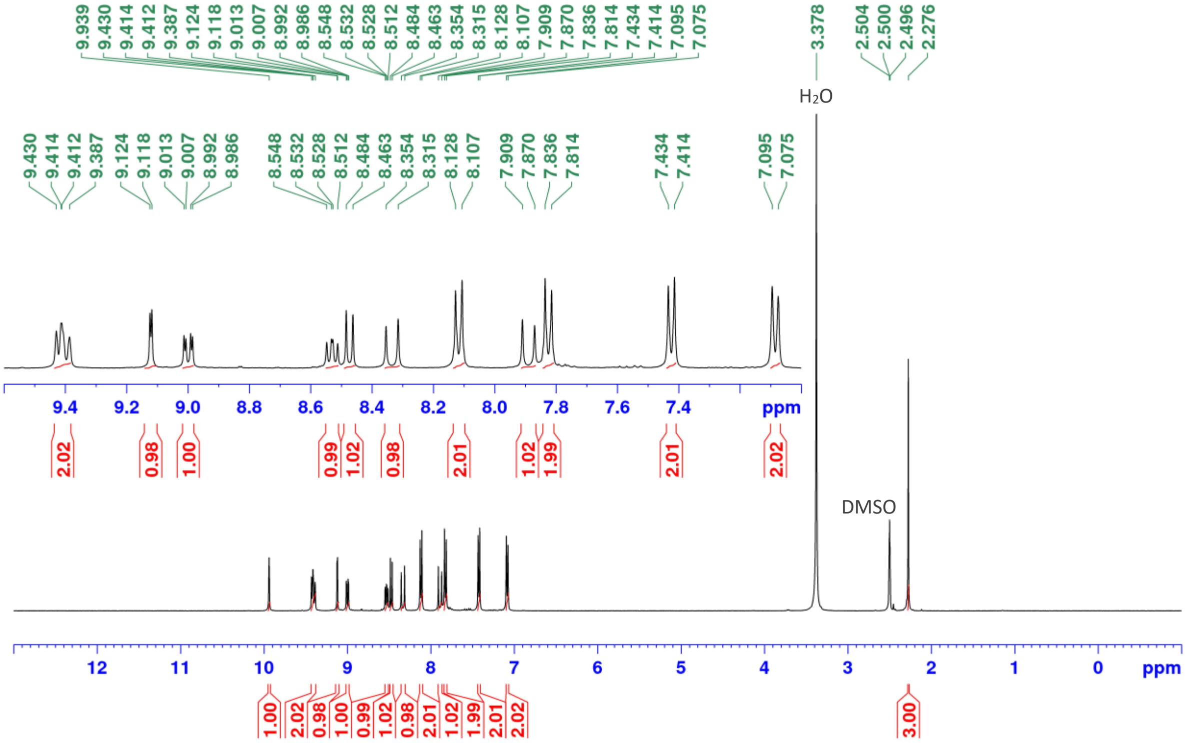

Figure S18. ^{1}H NMR (400 MHz) spectrum of **1a** in DMSO-d_6.

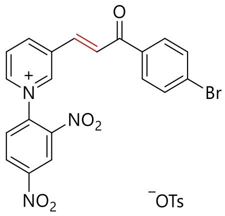

1a

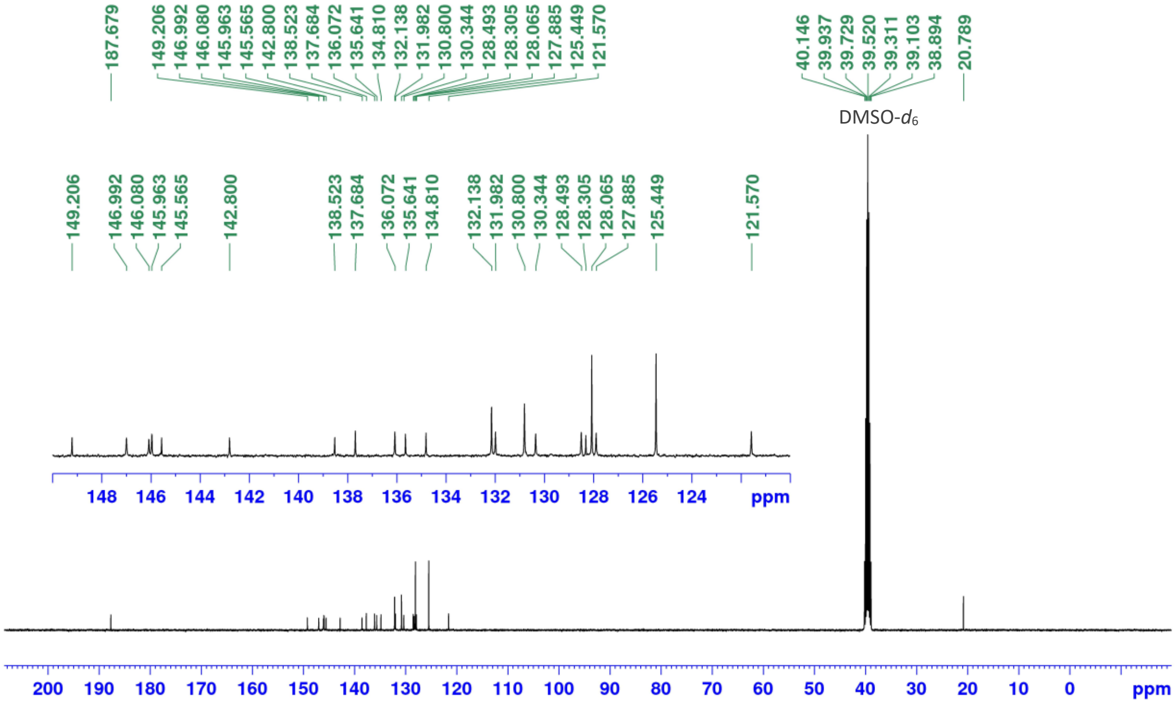

Figure S19. ^{13}C NMR (100 MHz) spectrum of **1a** in DMSO-d_6.

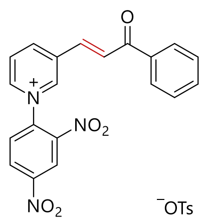

1b

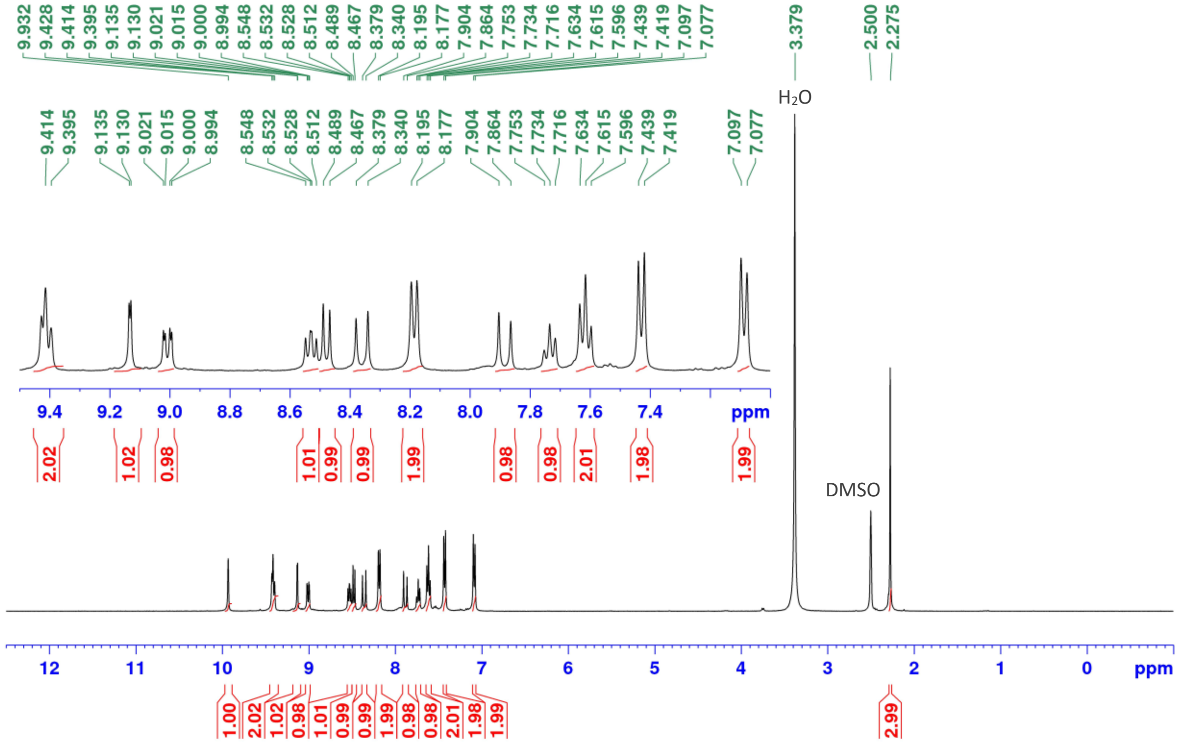

Figure S20. ^{1}H NMR (400 MHz) spectrum of **1b** in DMSO-d_6.

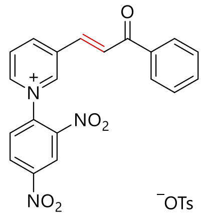

1b

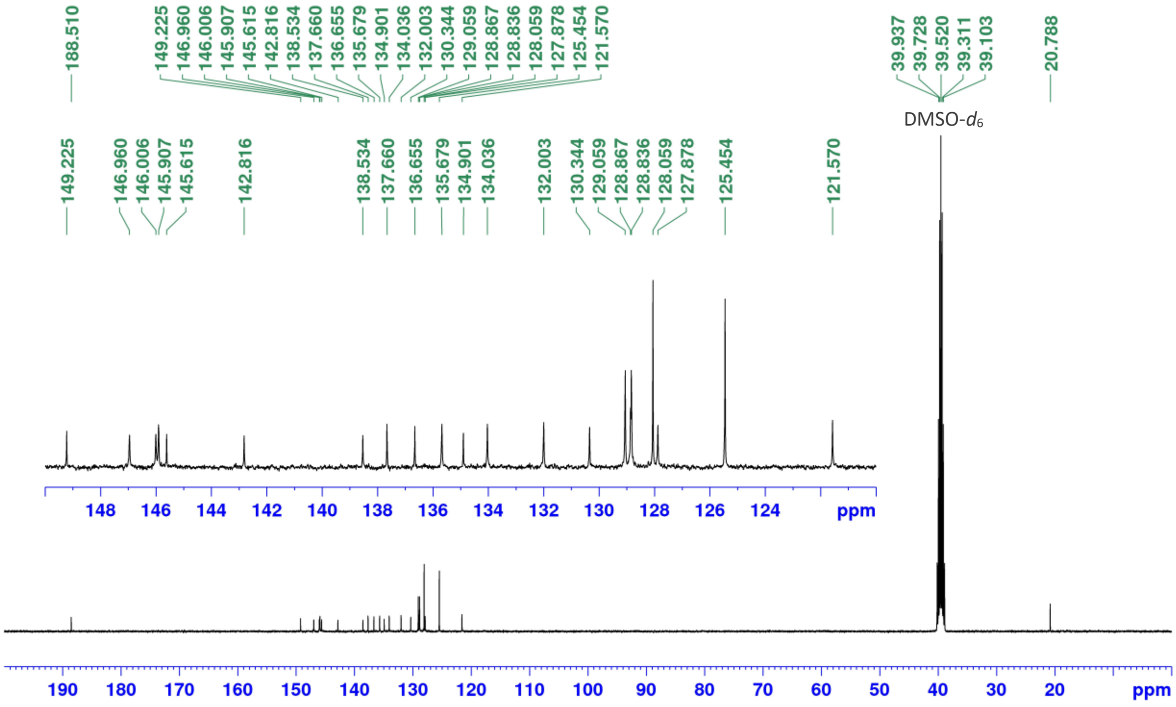

Figure S21. ^{13}C NMR (100 MHz) spectrum of **1b** in DMSO-d_6.

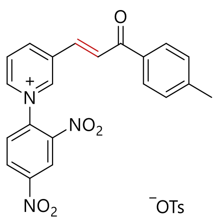

1c

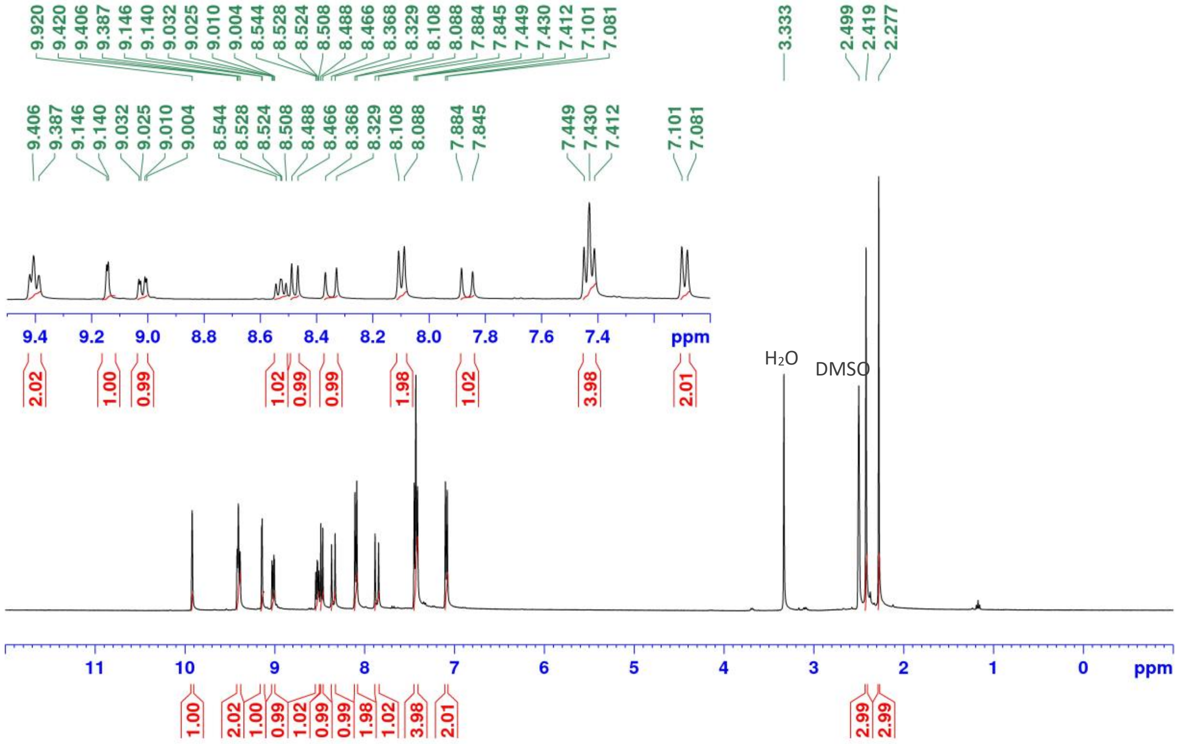

Figure S22. ^{1}H NMR (400 MHz) spectrum of **1c** in DMSO-d_6.

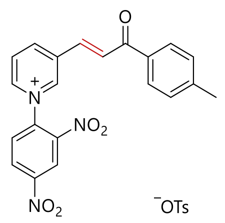

1c

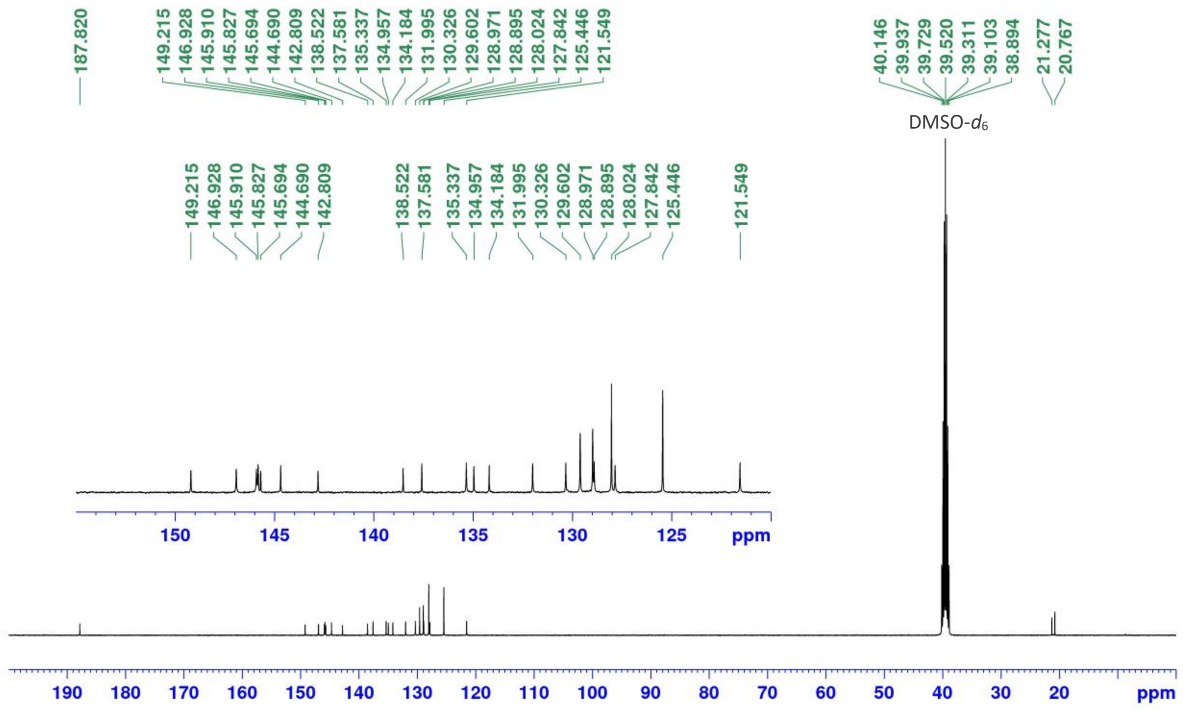

Figure S23. ^{13}C NMR (100 MHz) spectrum of **1c** in DMSO-d_6.

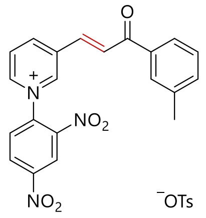

1d

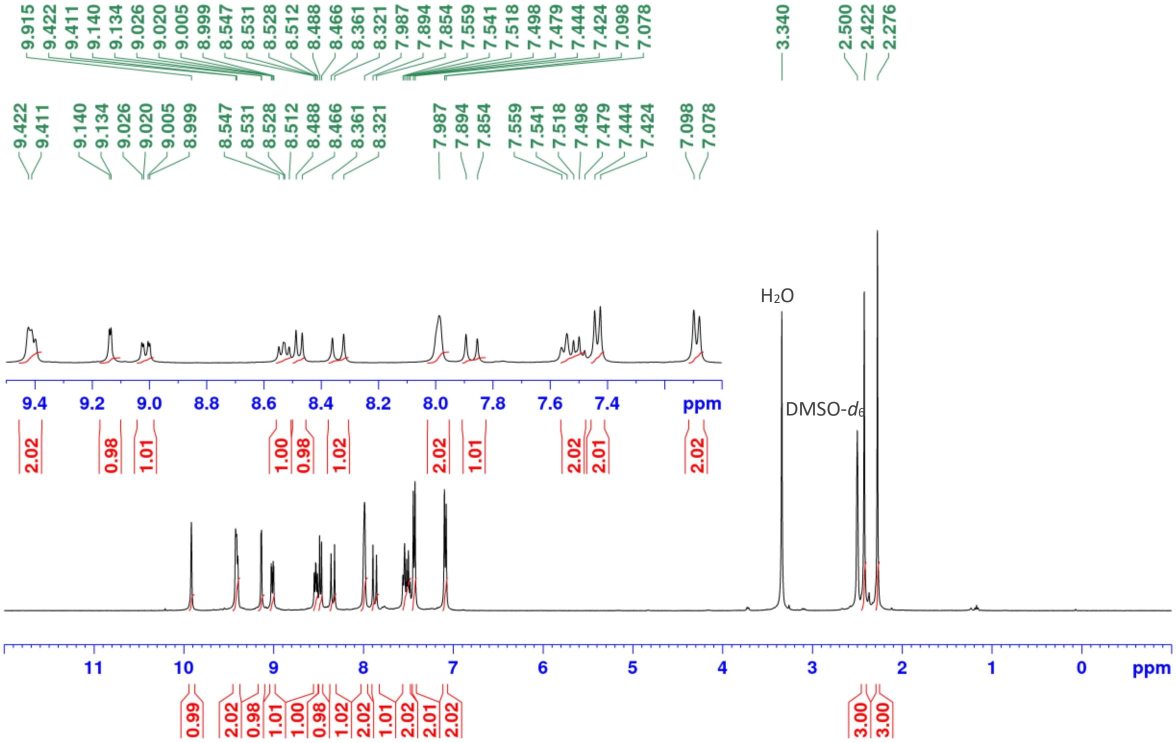

Figure S24. ^{1}H NMR (400 MHz) spectrum of **1d** in DMSO-d_6.

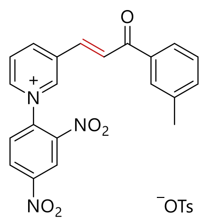

1d

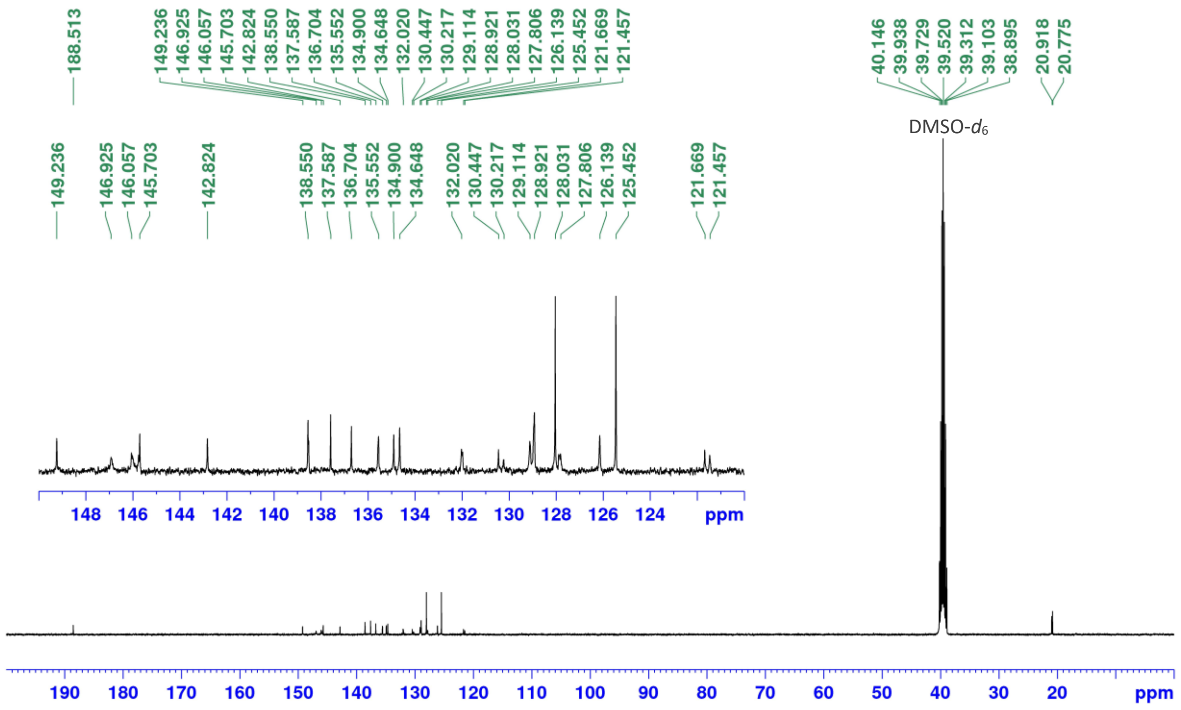

Figure S25. ^{13}C NMR (100 MHz) spectrum of **1d** in DMSO-d_6.

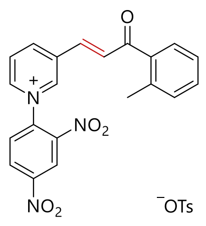

1e

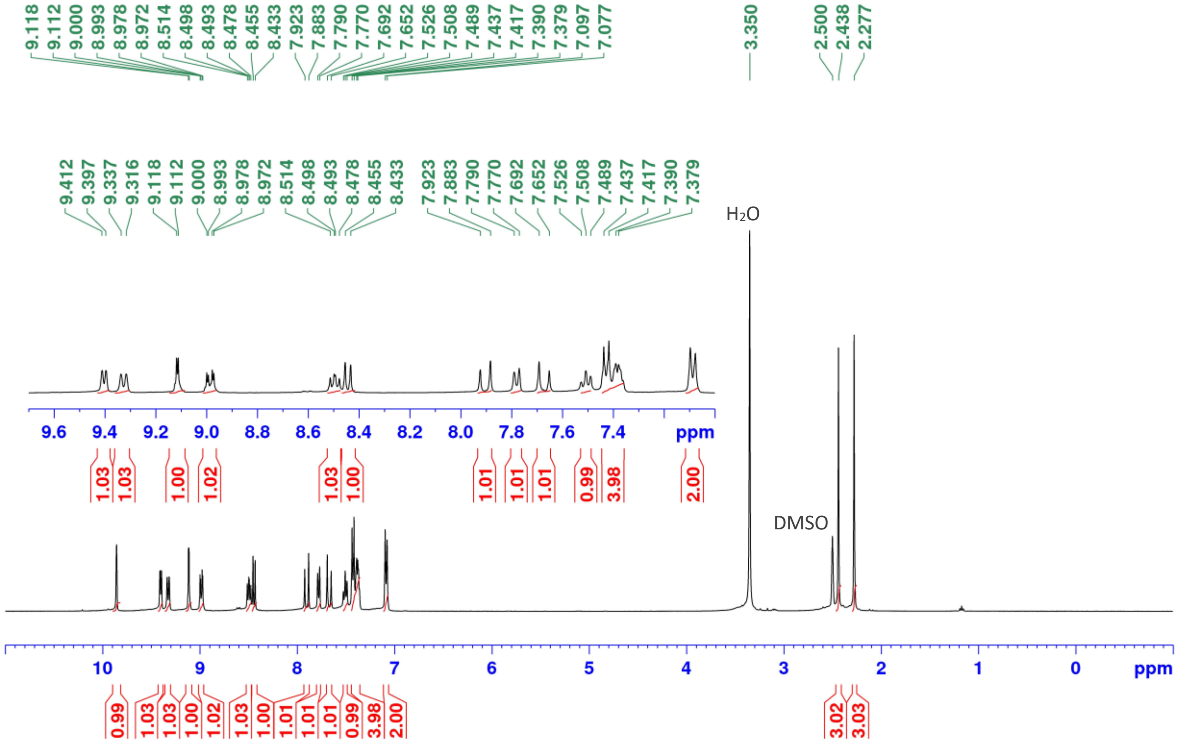

Figure S26. ^{1}H NMR (400 MHz) spectrum of **1e** in DMSO-d_6.

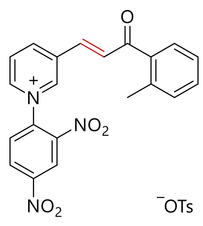

1e

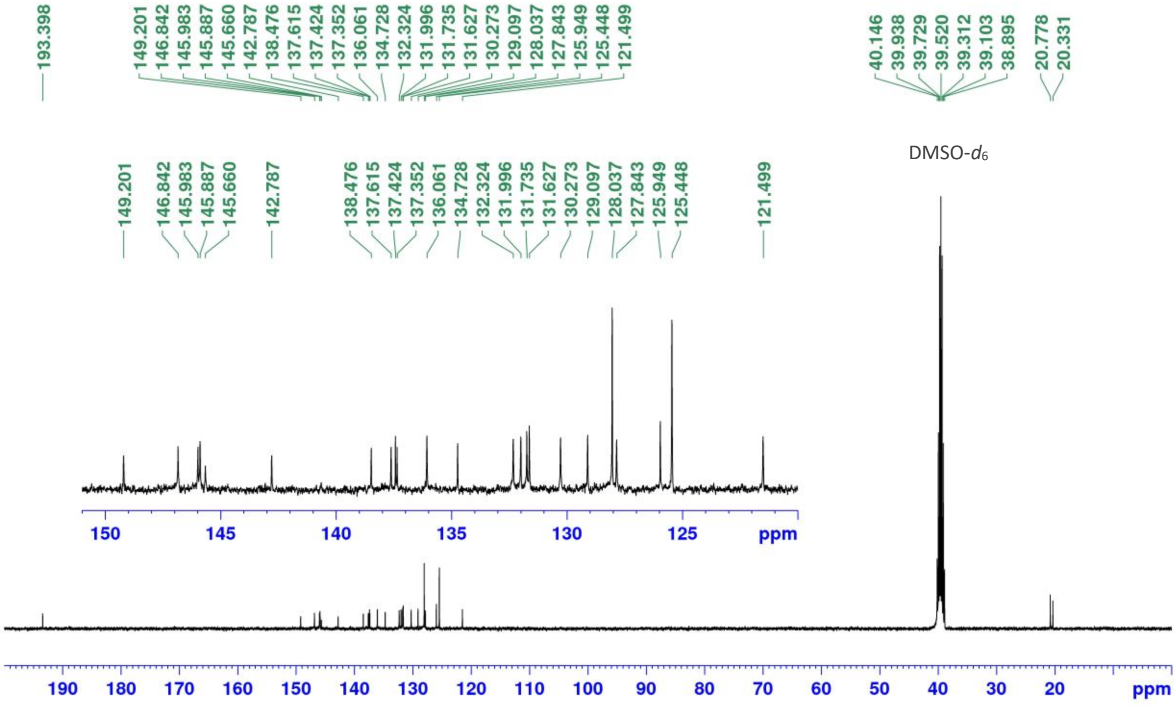

Figure S27. ^{13}C NMR (100 MHz) spectrum of **1e** in DMSO-d_6.

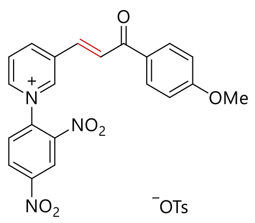

1f

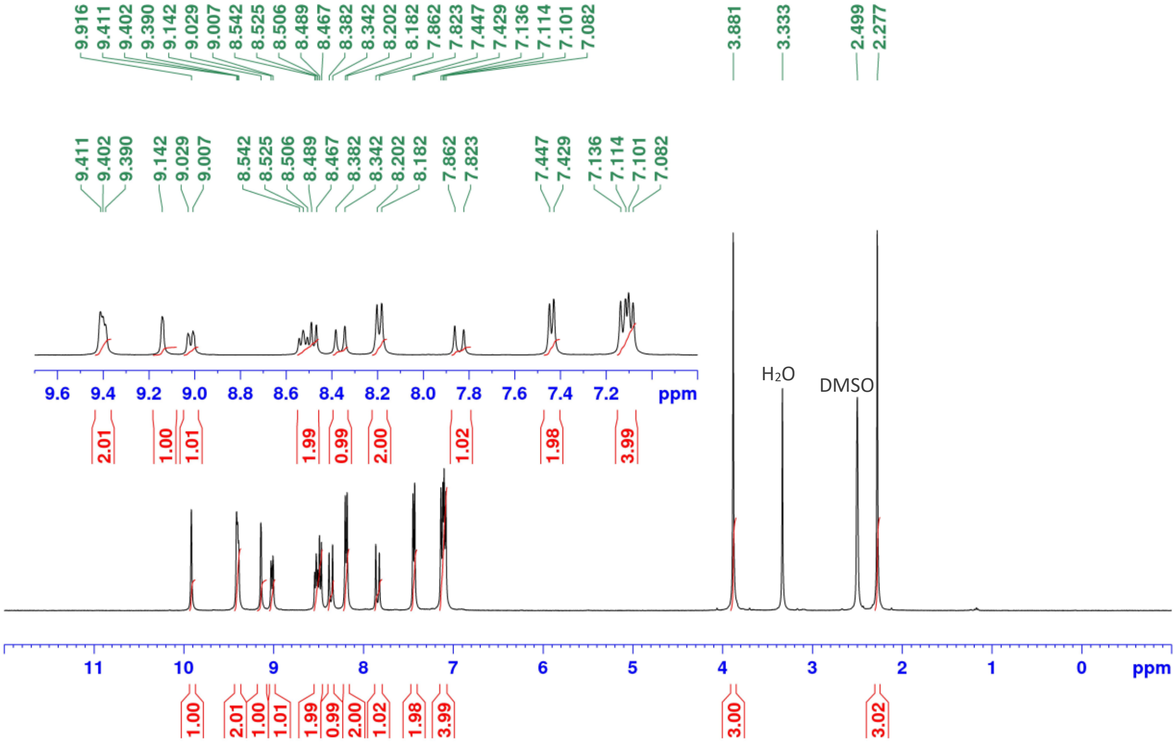

Figure S28. ^{1}H NMR (400 MHz) spectrum of **1f** in DMSO-d_6.

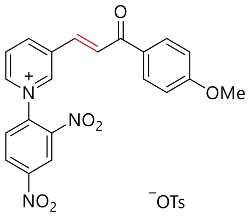

1f

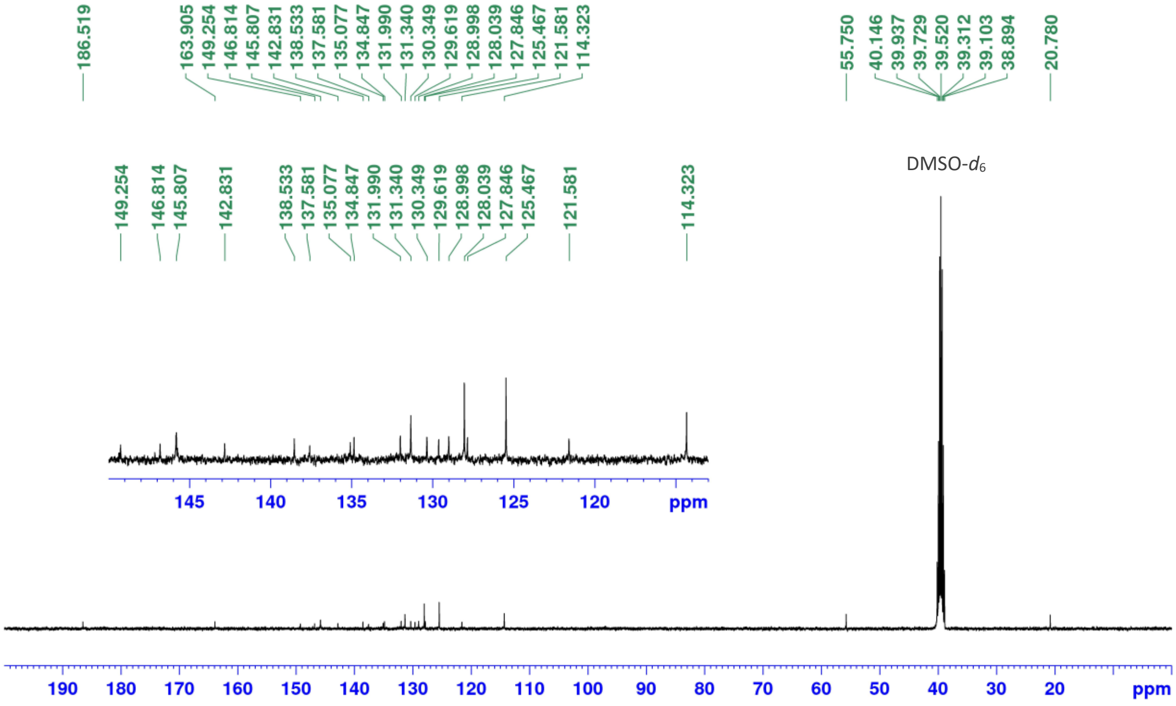

Figure S29. ^{13}C NMR (100 MHz) spectrum of **1f** in DMSO-d_6.

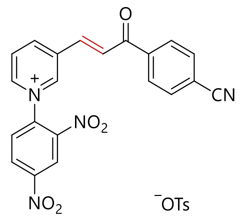

1g

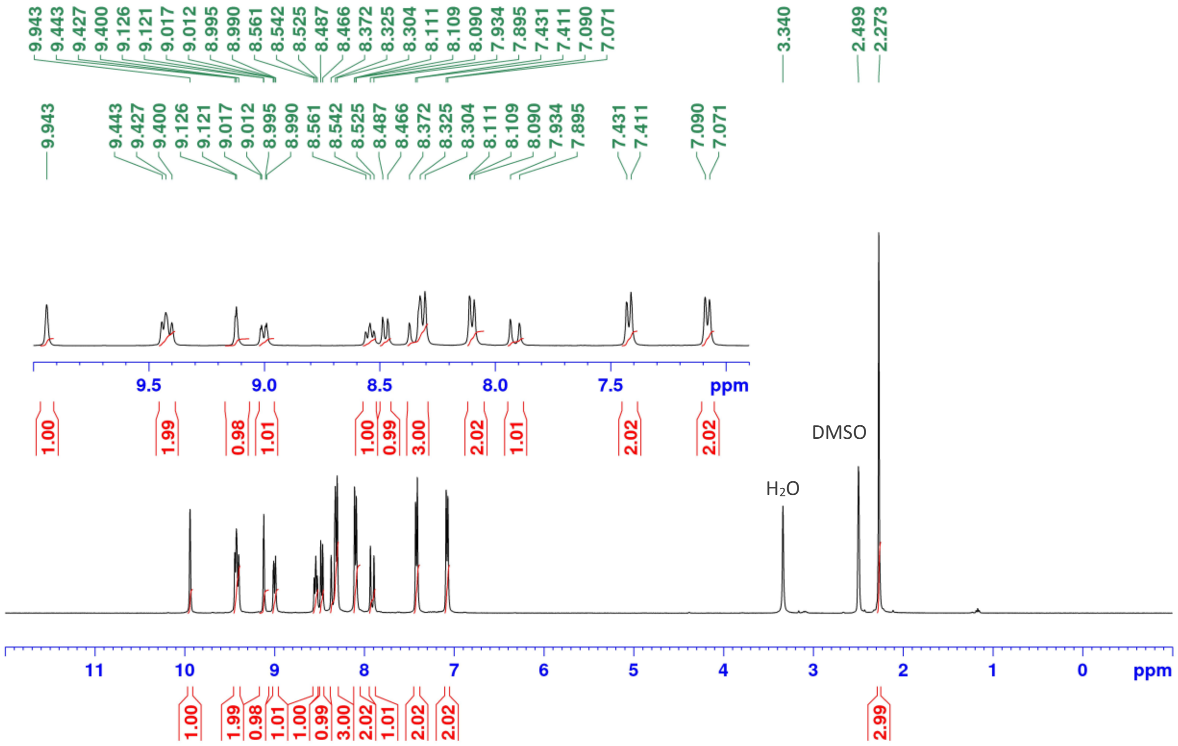

Figure S30. ^{1}H NMR (400 MHz) spectrum of **1g** in DMSO-d_6.

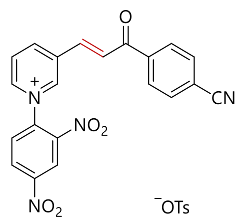

1g

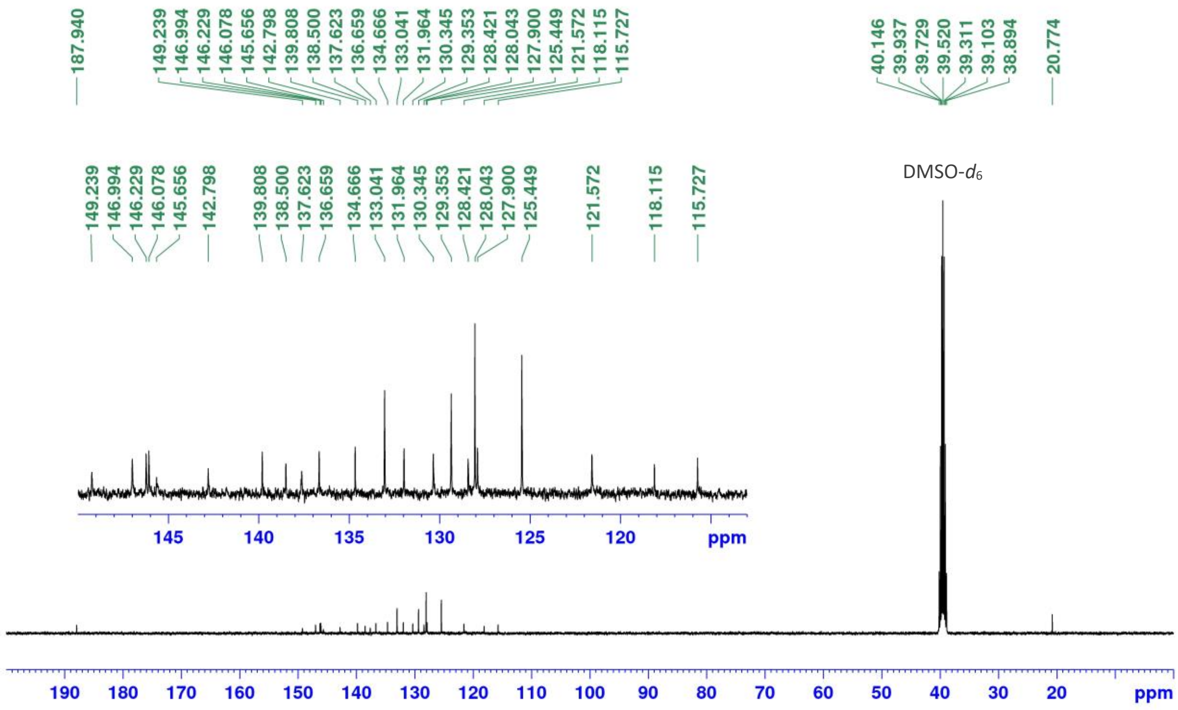

Figure S31. ^{13}C NMR (100 MHz) spectrum of **1g** in DMSO-d_6.

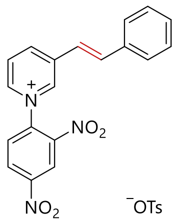

1h

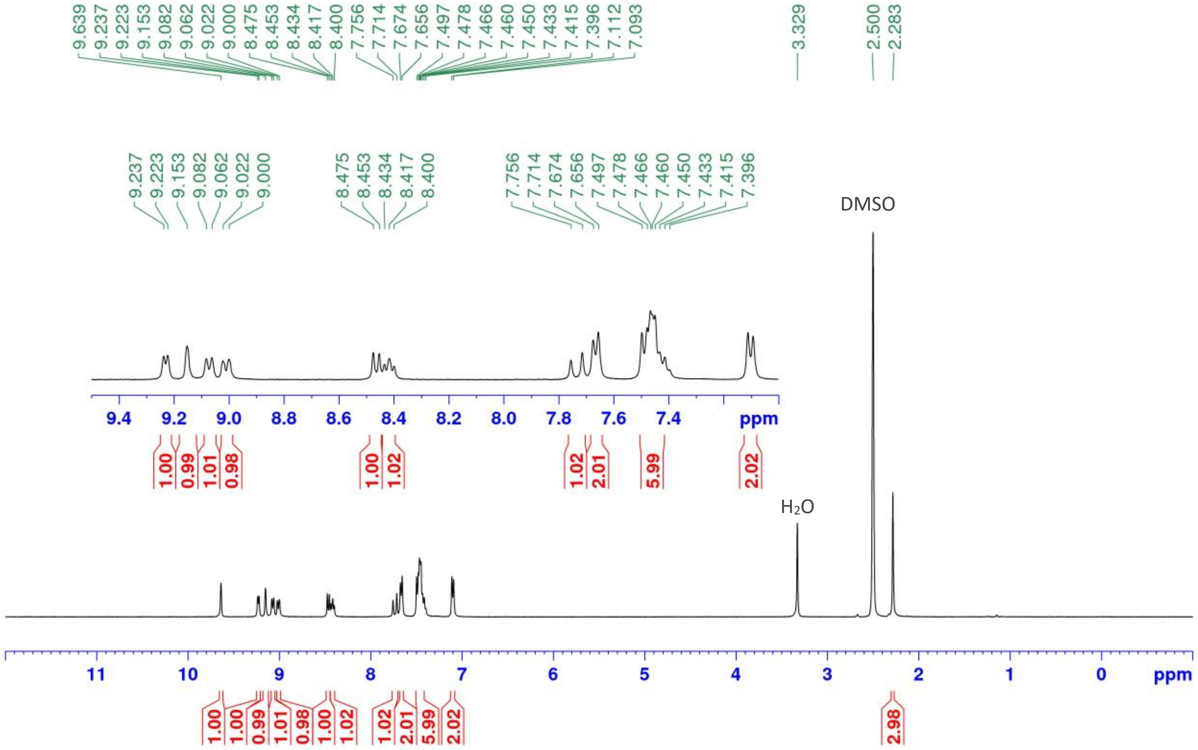

Figure S32. ^{1}H NMR (400 MHz) spectrum of **1h** in DMSO-d_6.

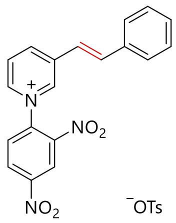

1h

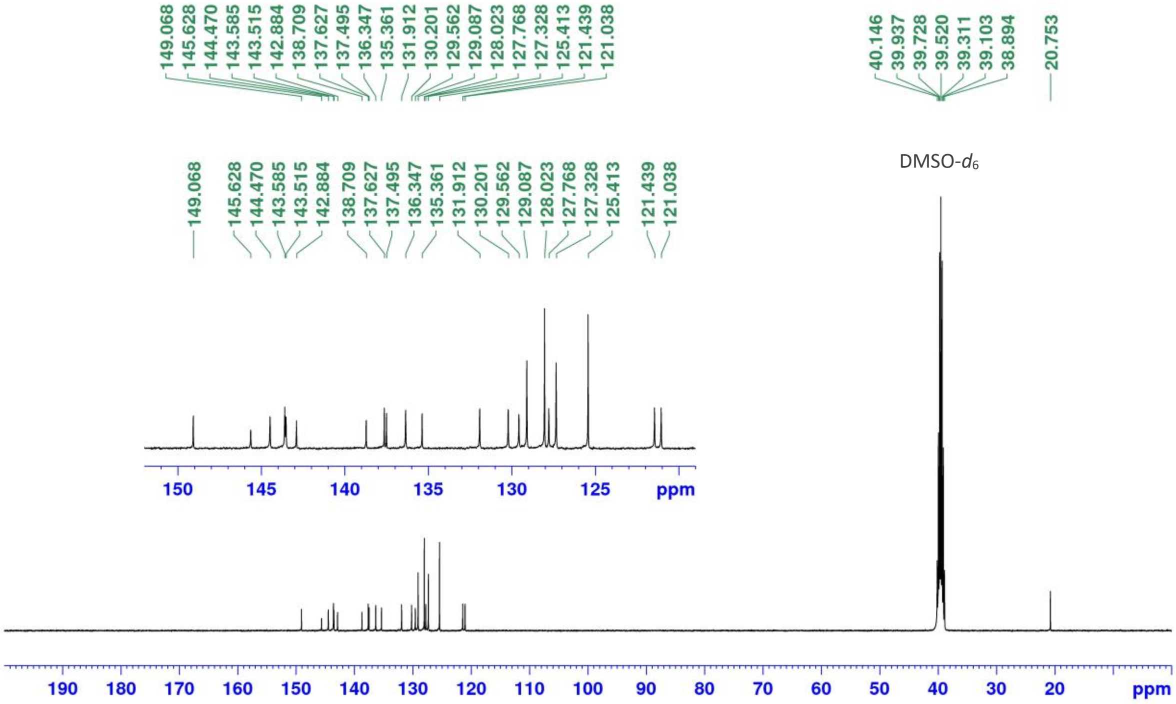

Figure S33. ^{13}C NMR (100 MHz) spectrum of **1h** in DMSO-d_6.

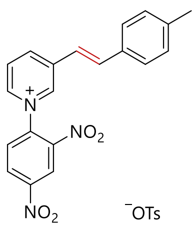

1i

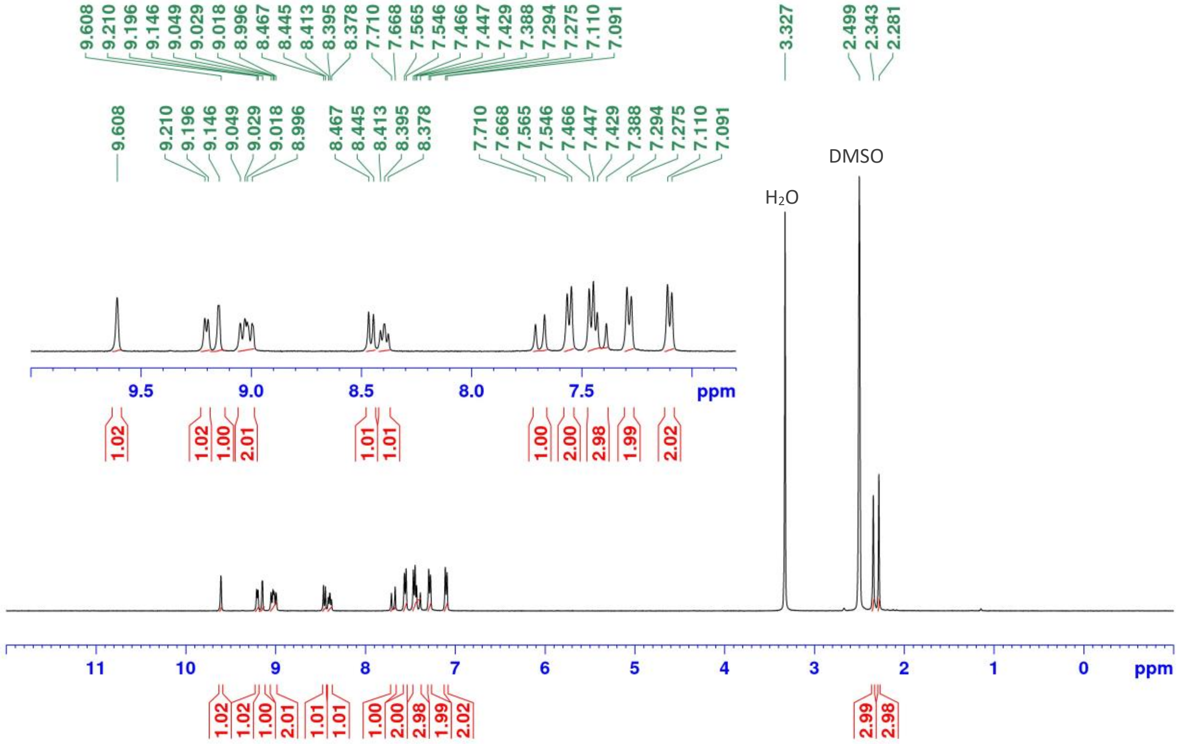

Figure S34. ^{1}H NMR (400 MHz) spectrum of **1i** in DMSO-d_6.

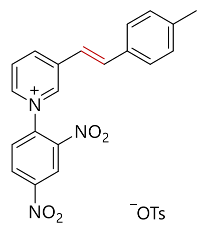

1i

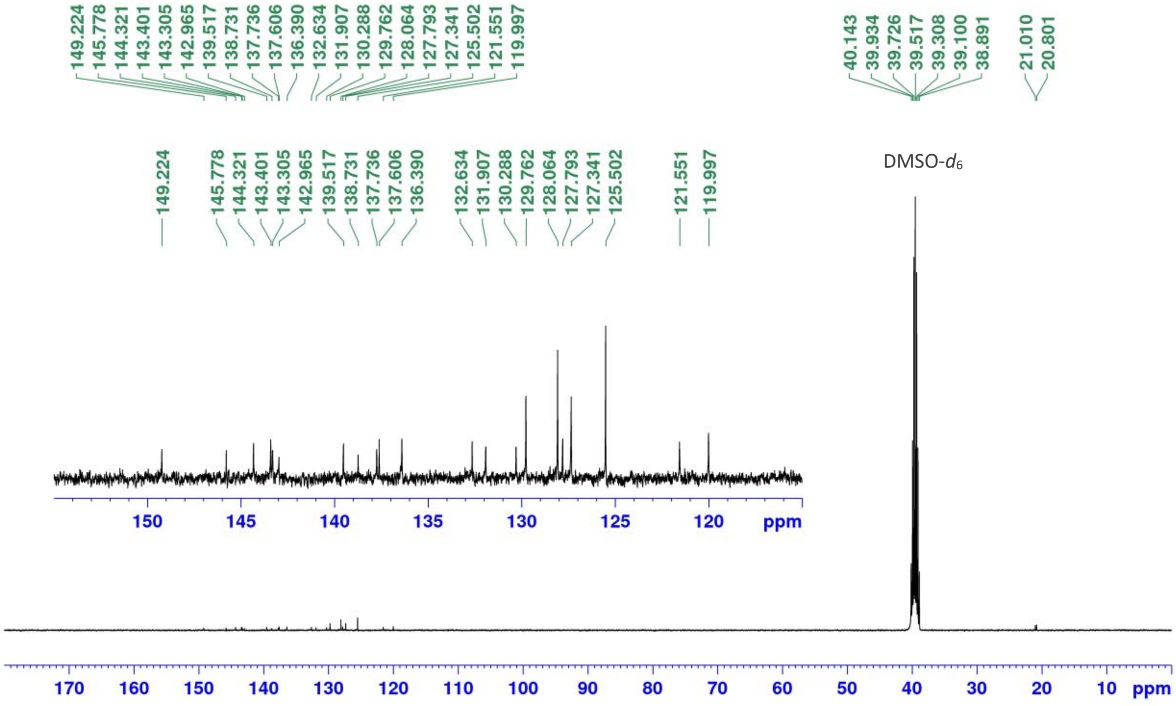

Figure S35. ^{13}C NMR (100 MHz) spectrum of **1i** in DMSO-d_6.

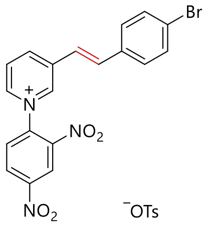

1j

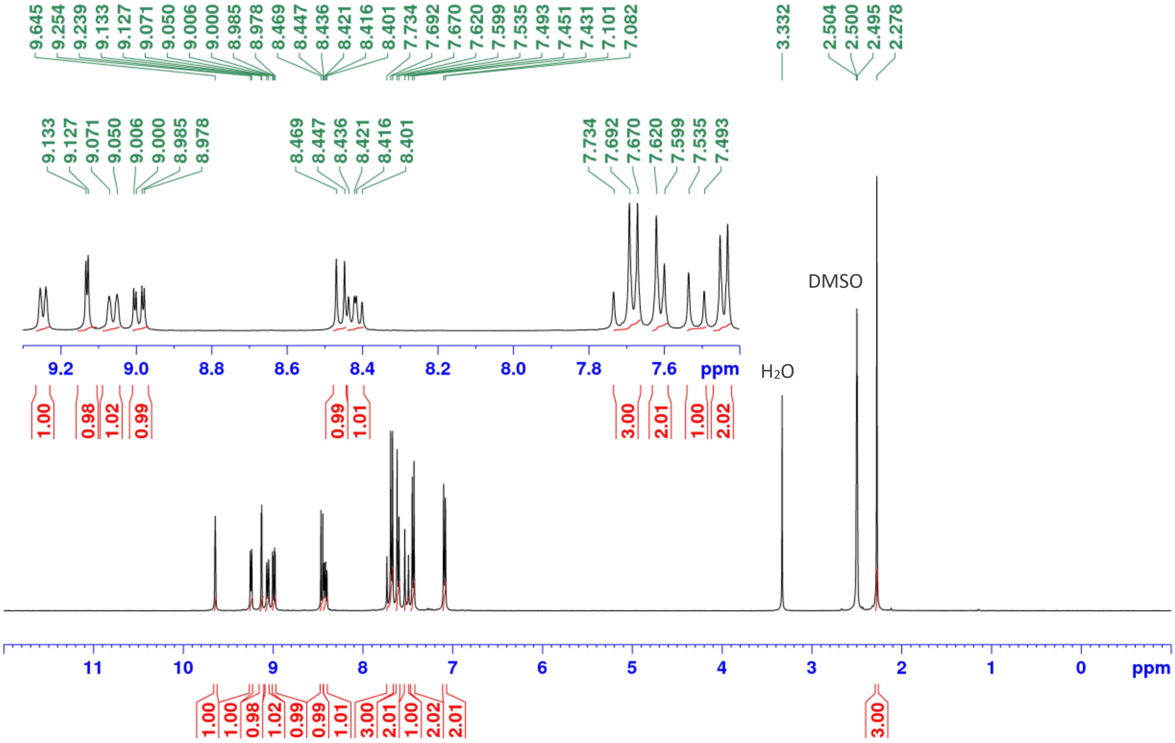

Figure S36. ^{1}H NMR (400 MHz) spectrum of **1j** in DMSO-d_6.

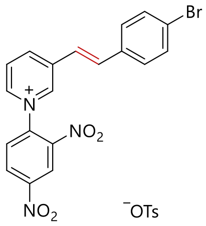

1j

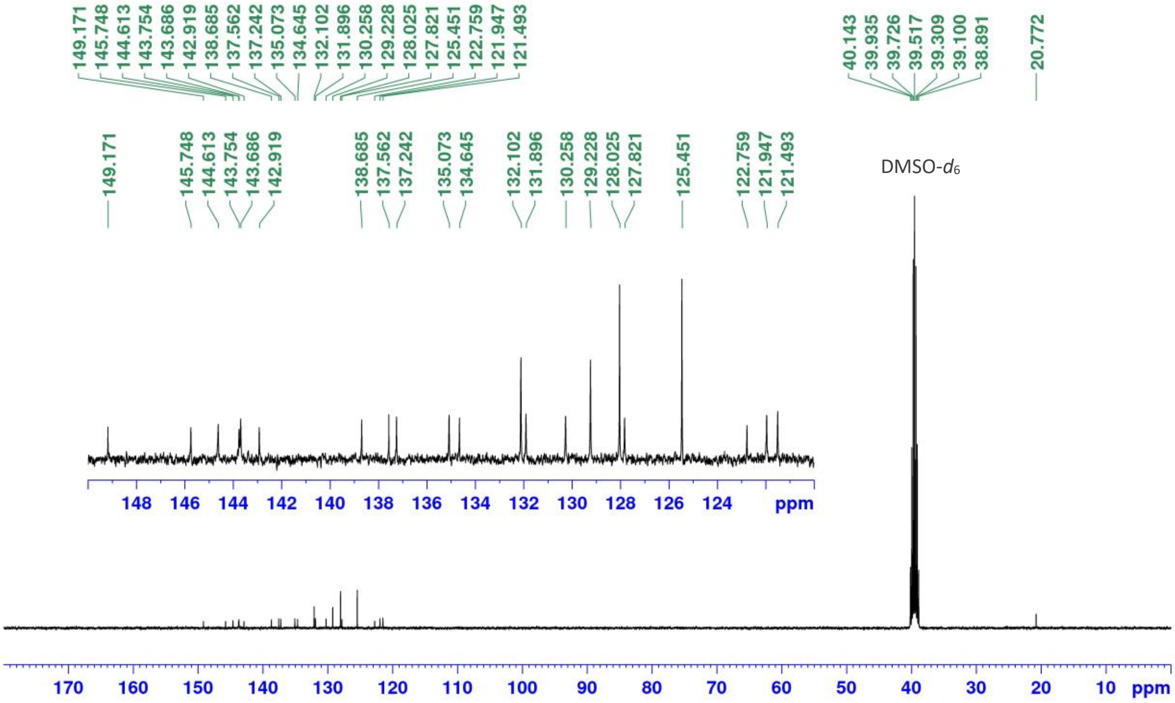

Figure S37. ^{13}C NMR (100 MHz) spectrum of **1j** in DMSO-d_6.

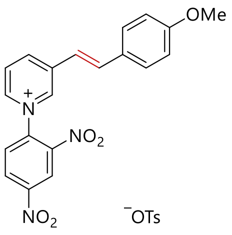

1k

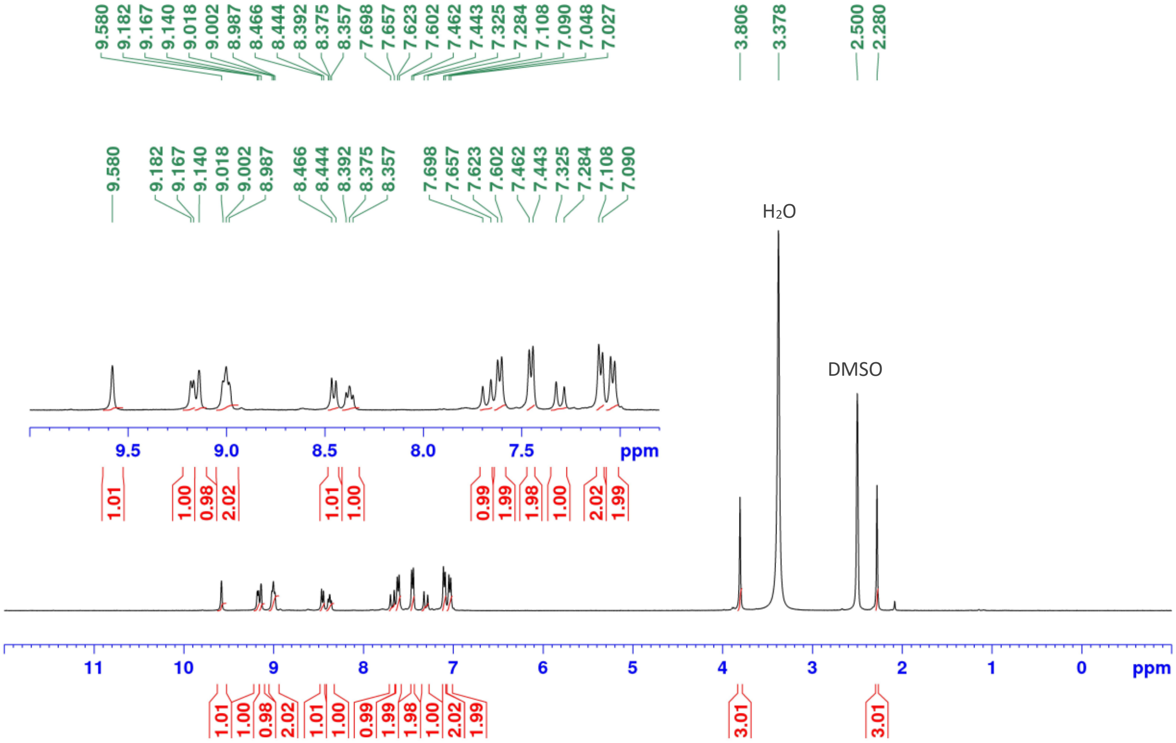

Figure S38. ^{1}H NMR (400 MHz) spectrum of **1k** in DMSO-d_6.

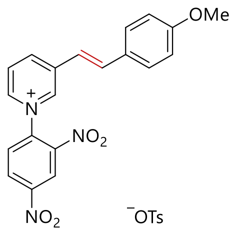

1k

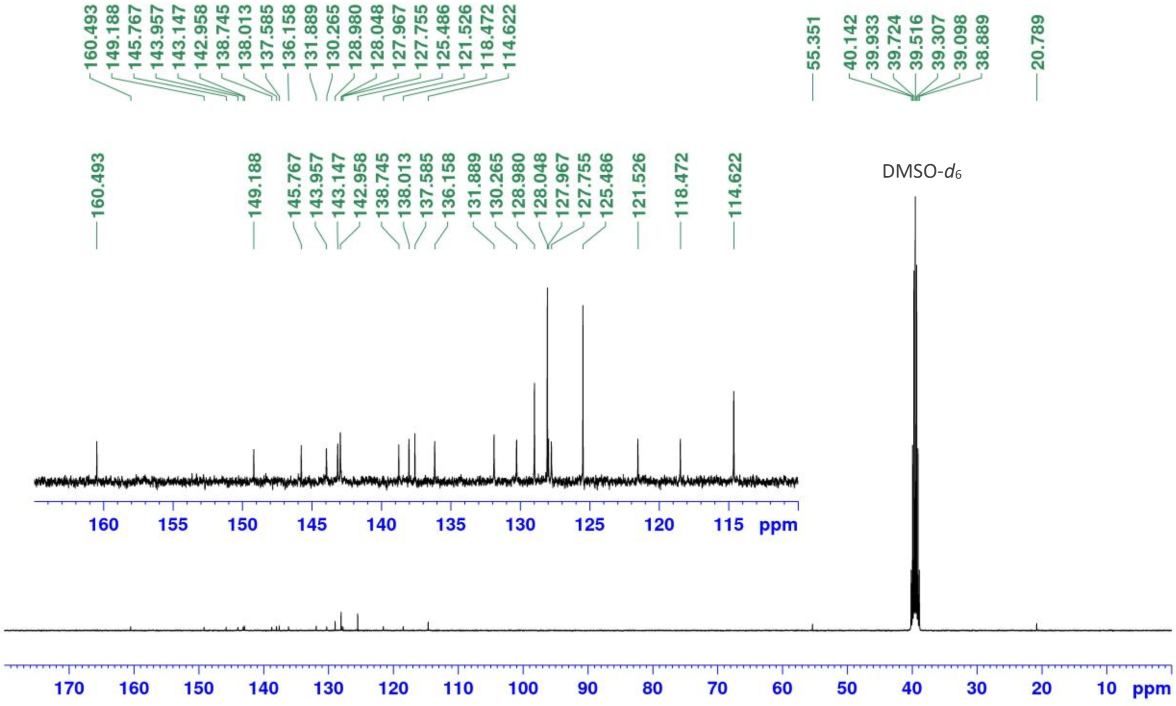

Figure S39. ^{13}C NMR (100 MHz) spectrum of **1k** in DMSO-d_6.

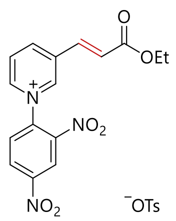

1l

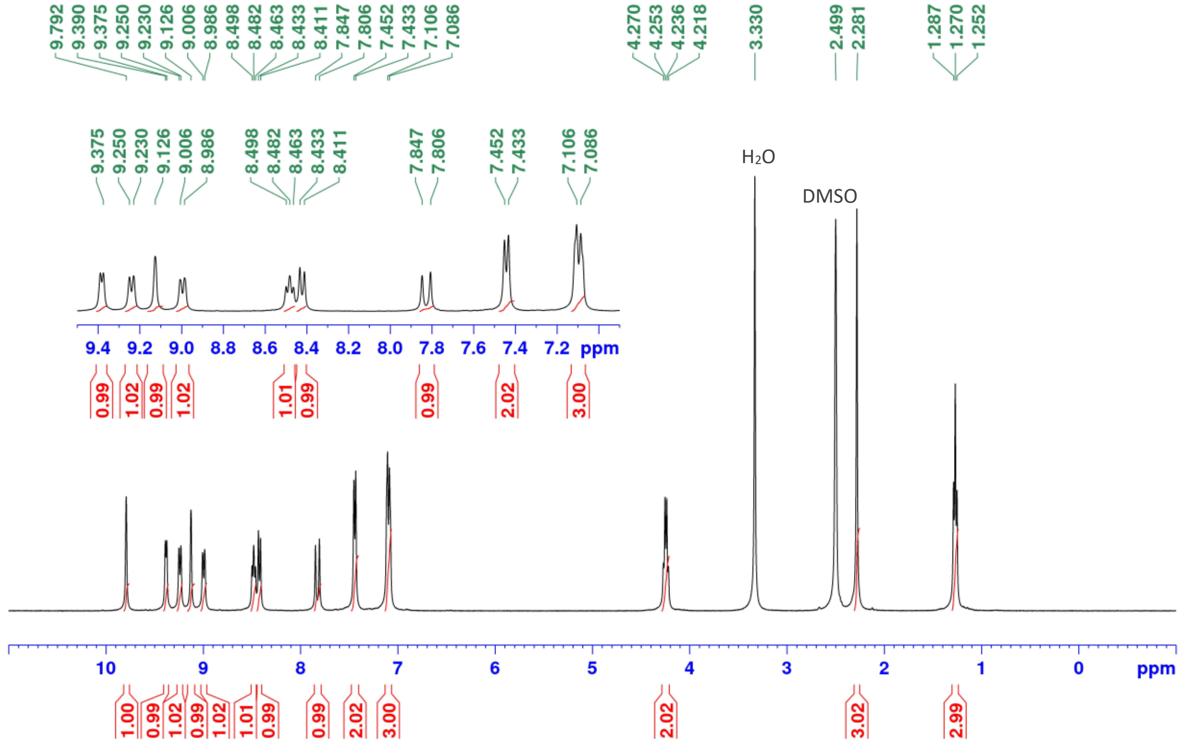

Figure S40. ^{1}H NMR (400 MHz) spectrum of **1l** in DMSO-d_6.

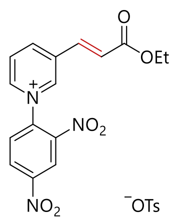

1l

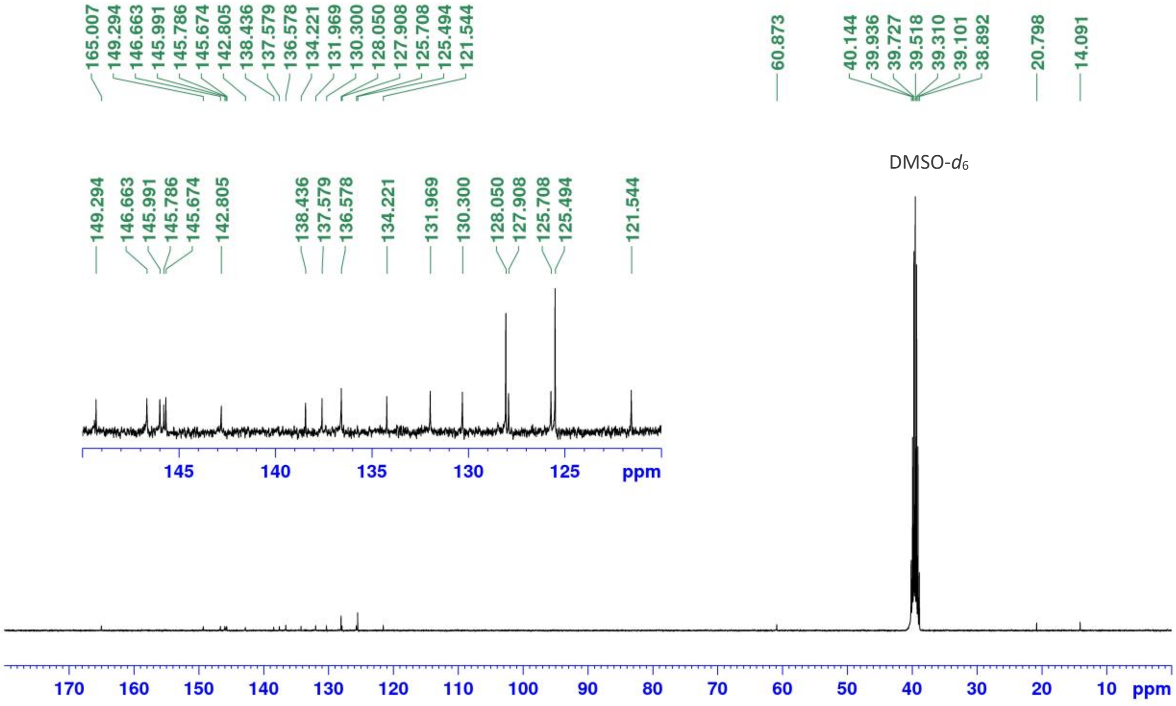

Figure S41. ^{13}C NMR (100 MHz) spectrum of **1l** in DMSO-d_6.

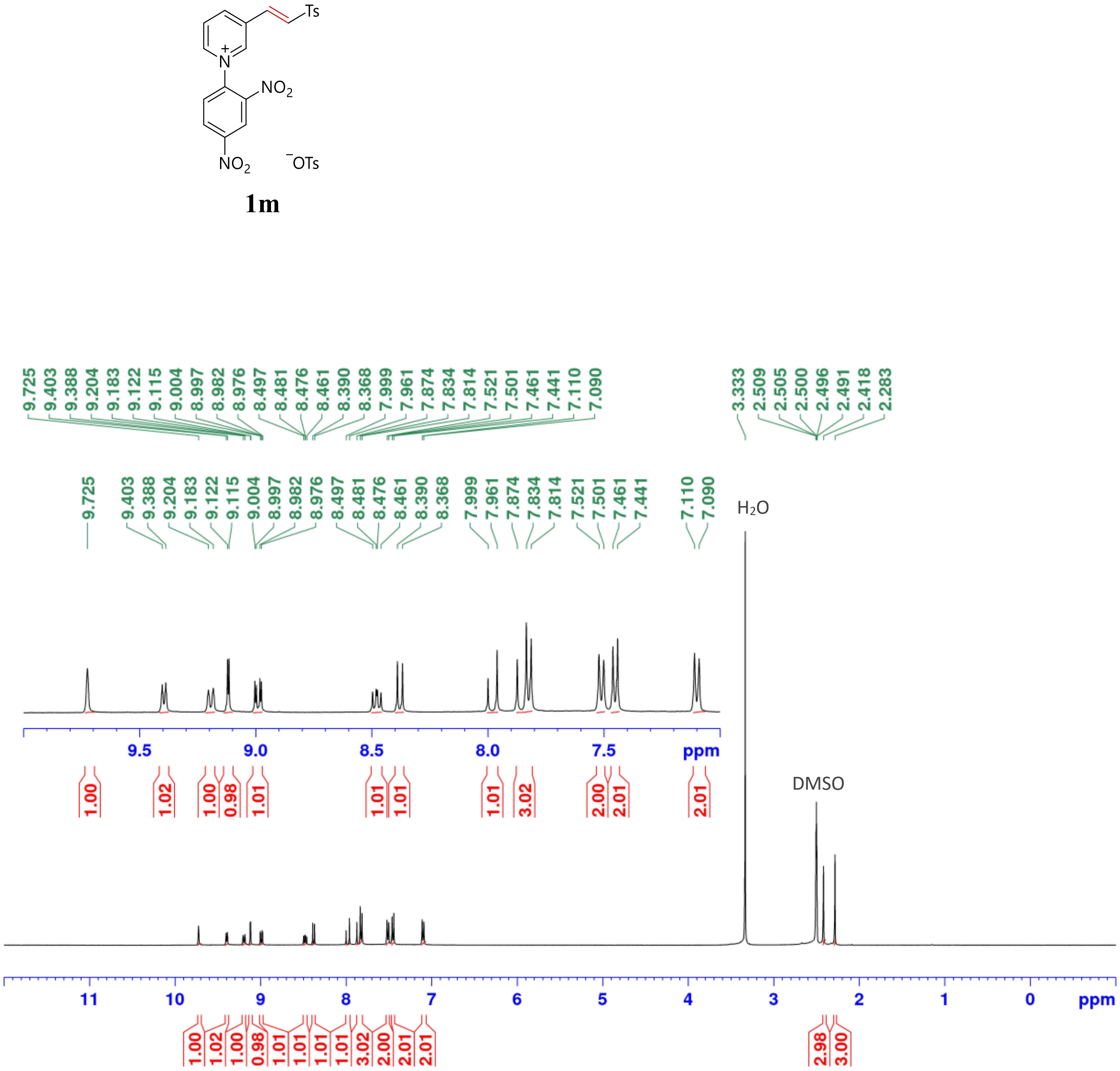

1m

Figure S42. ^{1}H NMR (400 MHz) spectrum of **1m** in DMSO-d_6.

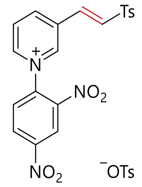

1m

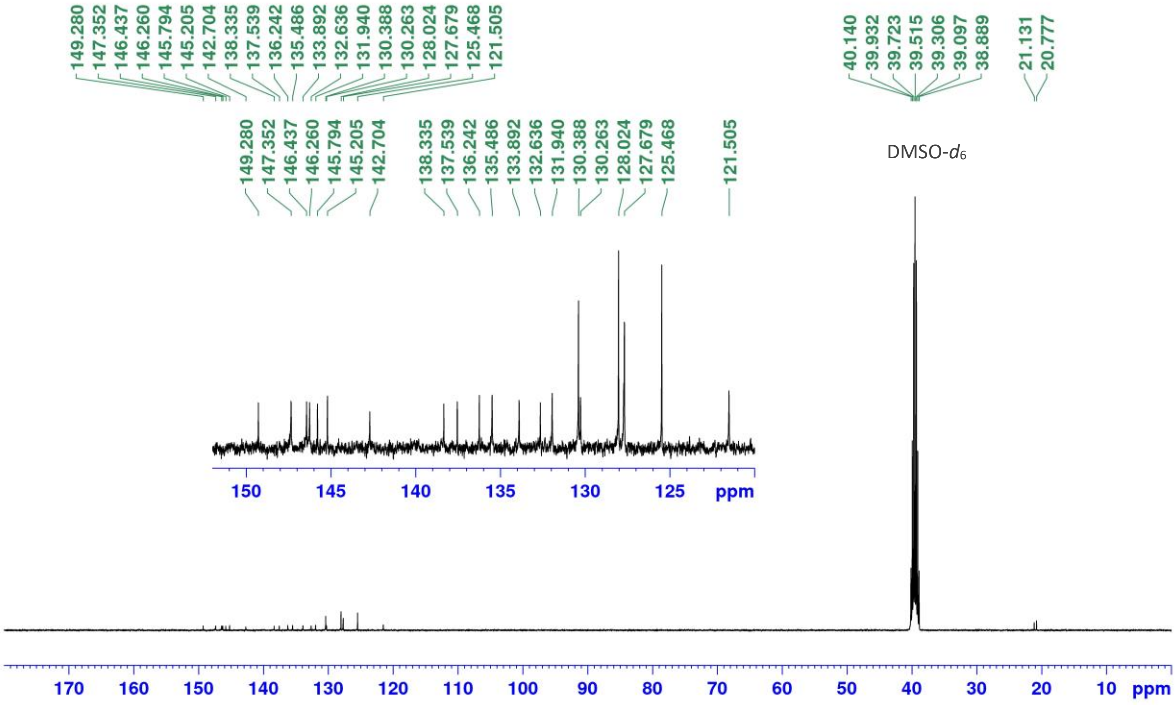

Figure S43. ^{13}C NMR (100 MHz) spectrum of **1m** in DMSO-d_6.

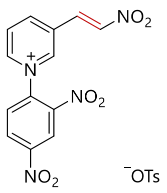

1n

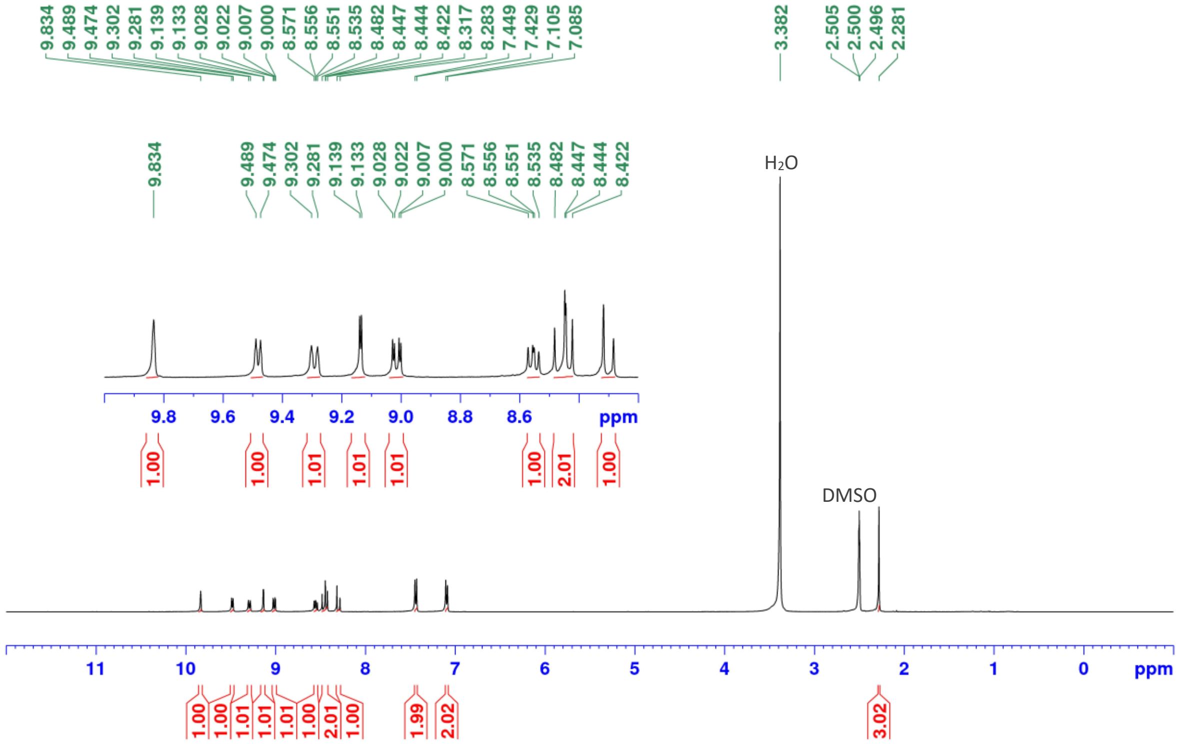

Figure S44. ¹H NMR (400 MHz) spectrum of **1n** in DMSO-d_6.

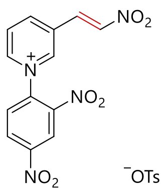

1n

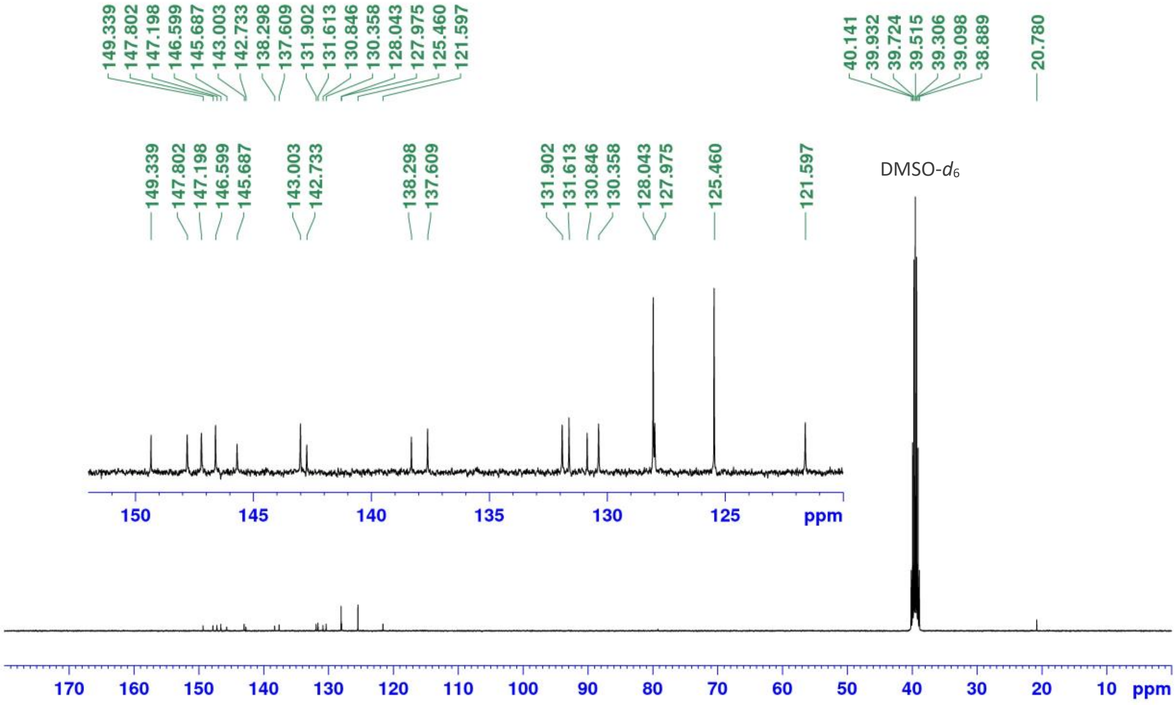

Figure S45. ^{13}C NMR (100 MHz) spectrum of **1n** in DMSO-d_6.

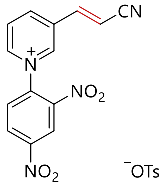

1o

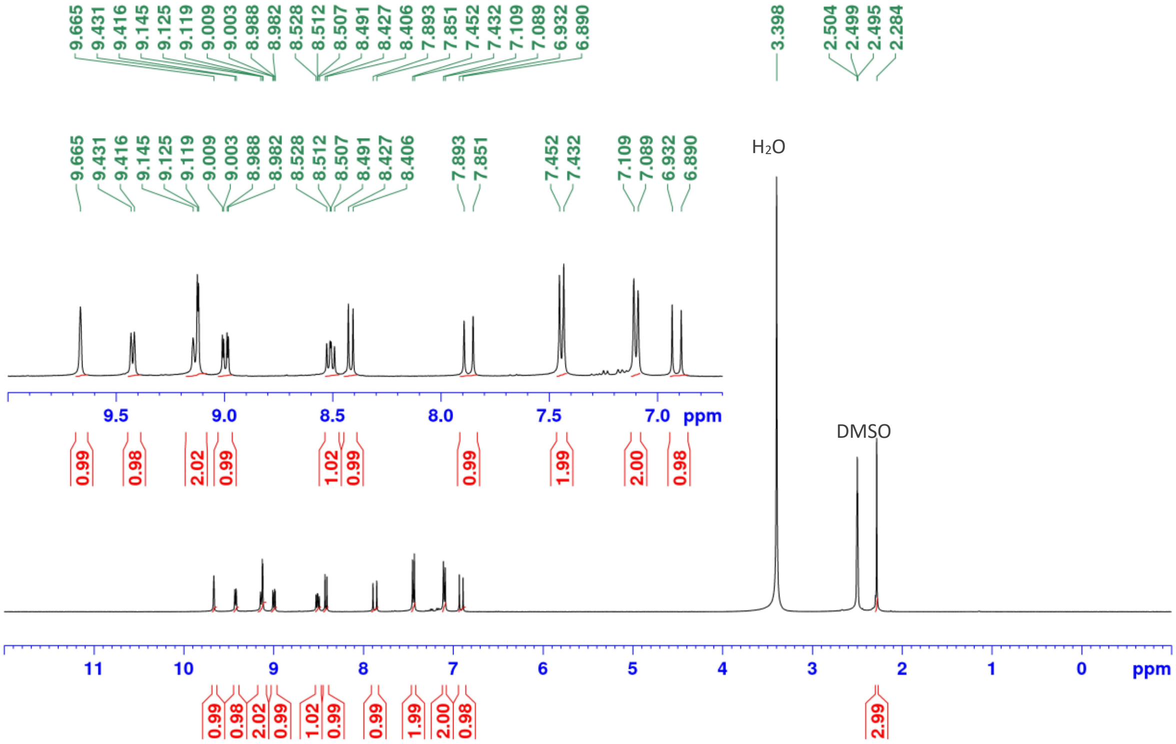

Figure S46. ^{1}H NMR (400 MHz) spectrum of **1o** in DMSO-d_6.

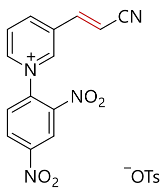

1o

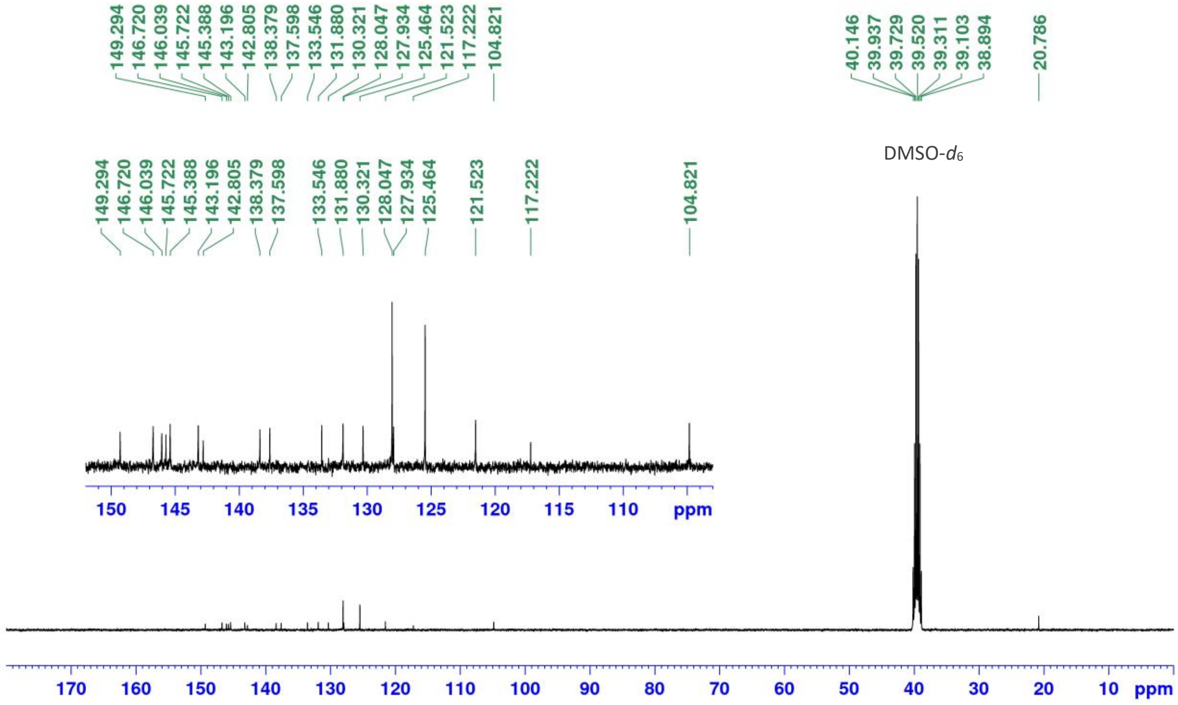

Figure S47. ^{13}C NMR (100 MHz) spectrum of **1o** in DMSO-d_6.

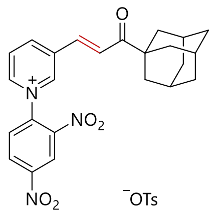

1p

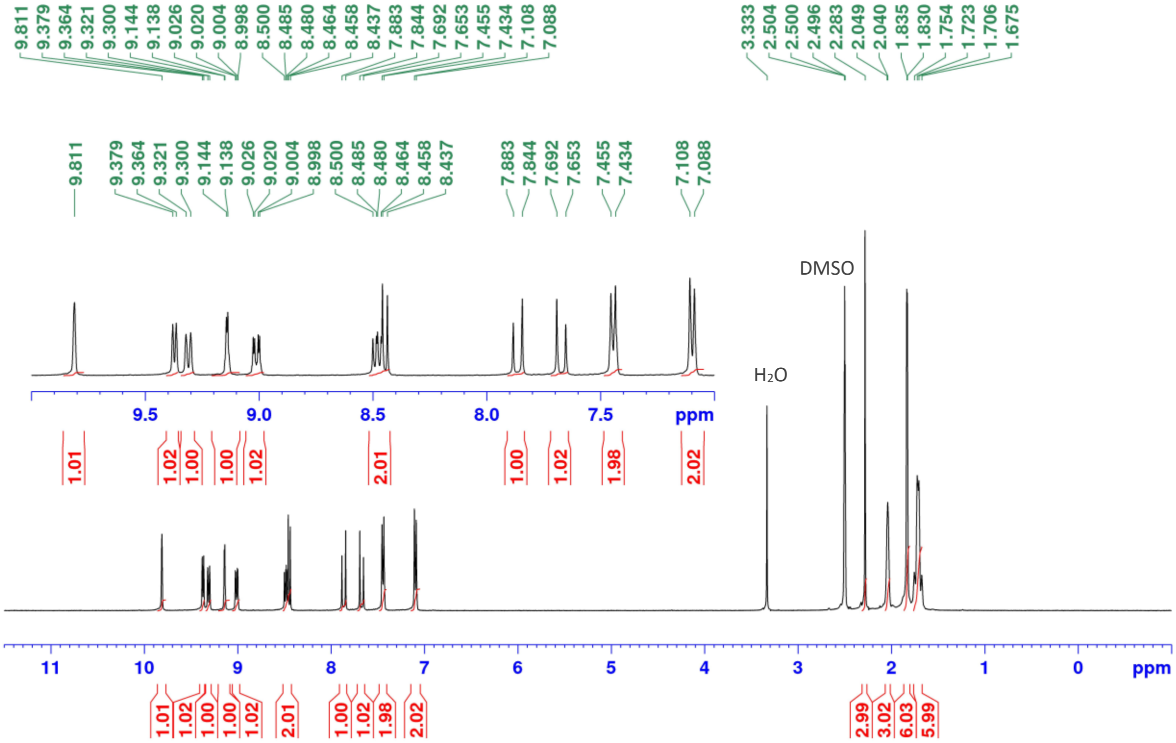

Figure S48. ^{1}H NMR (400 MHz) spectrum of **1p** in DMSO-d_6.

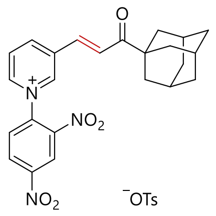

1p

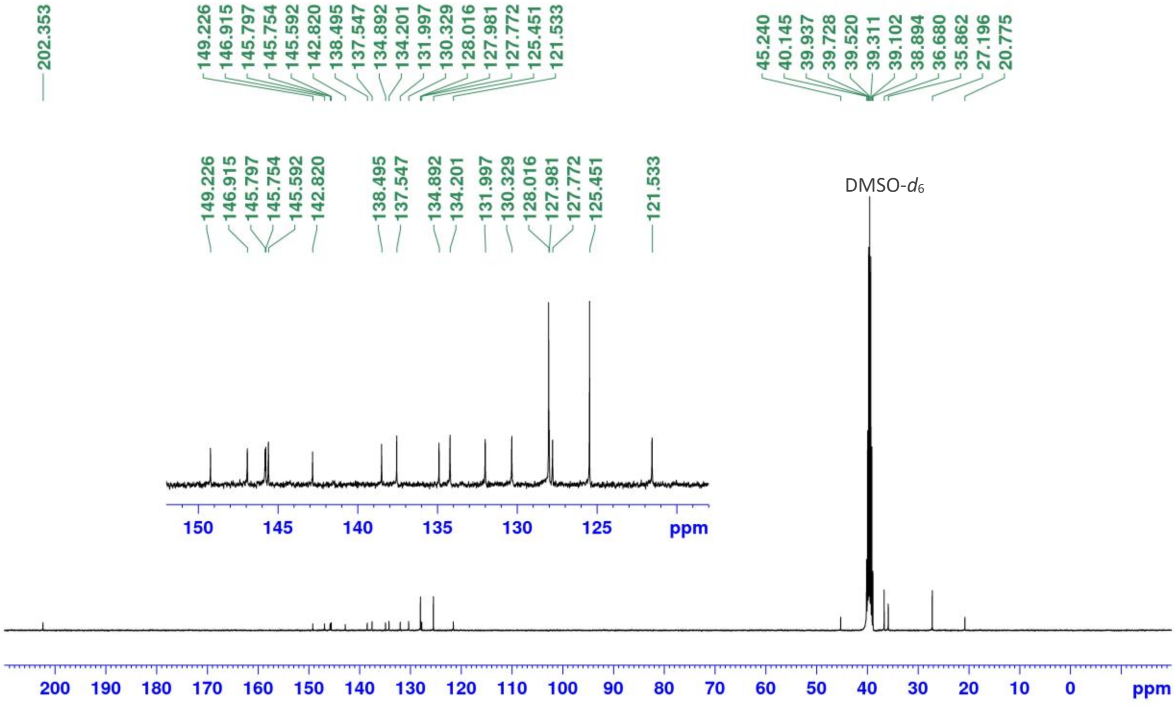

Figure S49. ^{13}C NMR (100 MHz) spectrum of **1p** in DMSO-d_6.

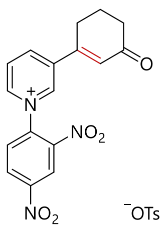

1q

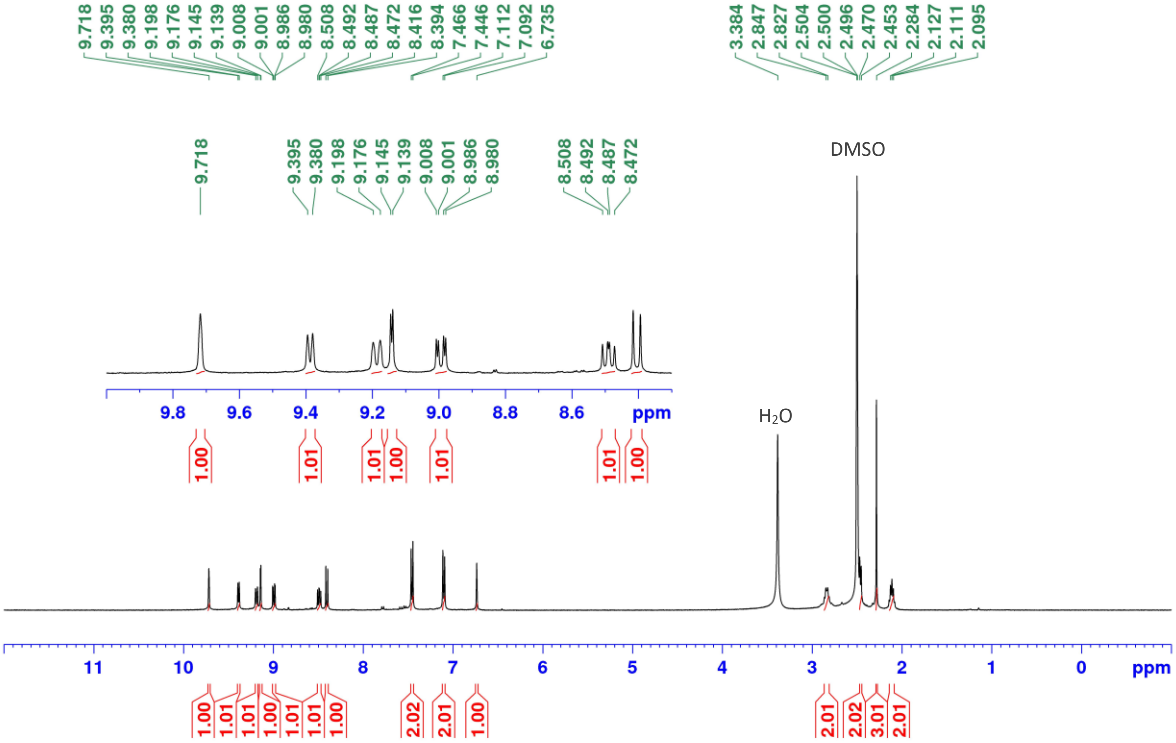

Figure S50. ^{1}H NMR (400 MHz) spectrum of **1q** in DMSO-d_6.

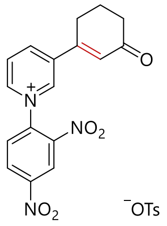

1q

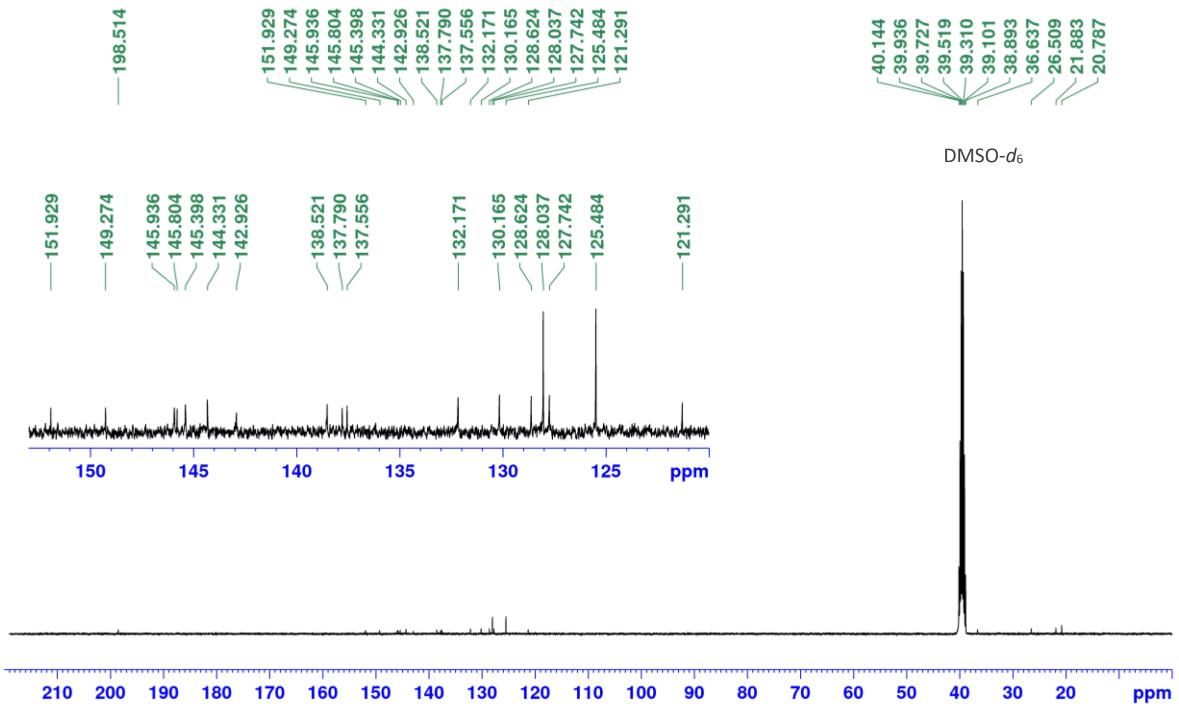

Figure S51. ^{13}C NMR (100 MHz) spectrum of **1q** in DMSO-d_6.

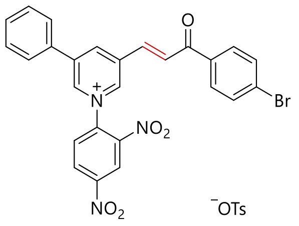

1r

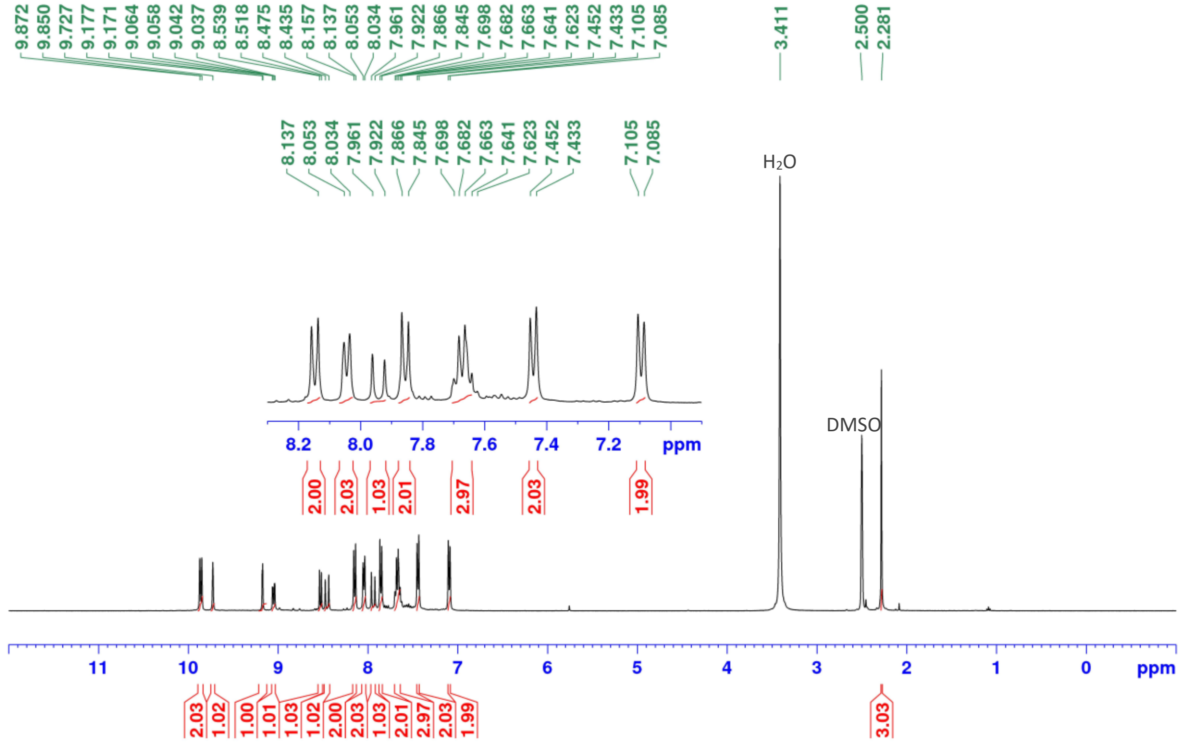

Figure S52. ^{1}H NMR (400 MHz) spectrum of **1r** in DMSO-d_6.

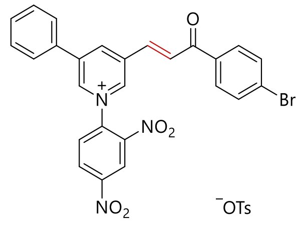

1r

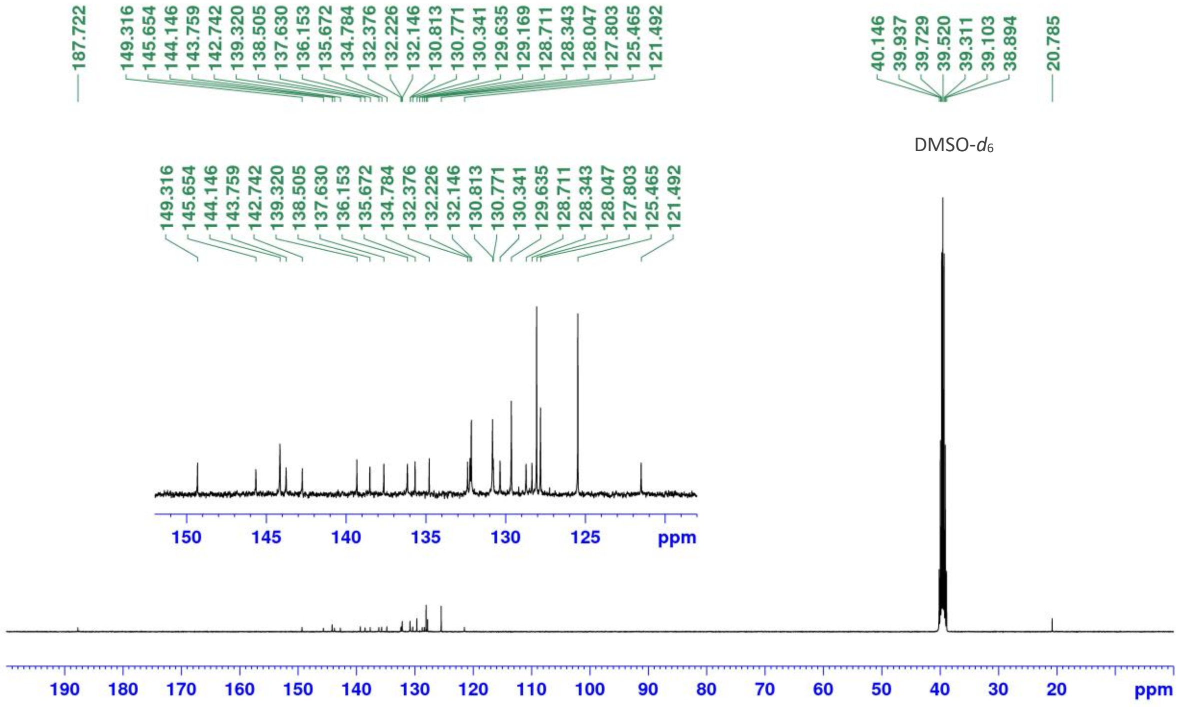

Figure S53. ^{13}C NMR (100 MHz) spectrum of **1r** in DMSO-d_6.

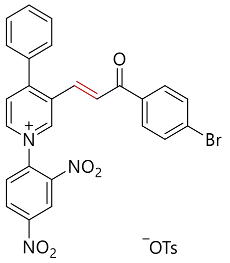

1s

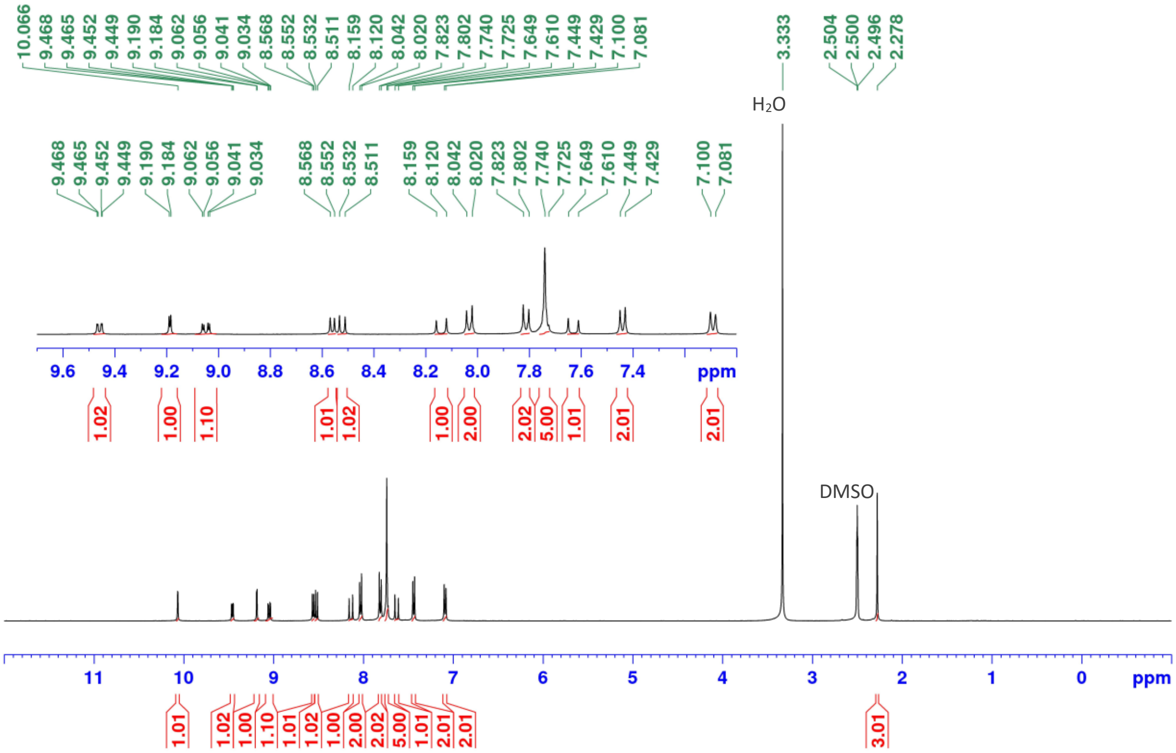

Figure S54. ^{1}H NMR (400 MHz) spectrum of **1s** in DMSO-d_6.

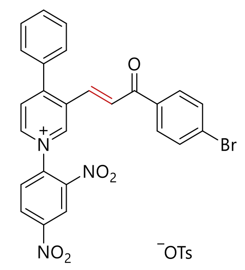

1s

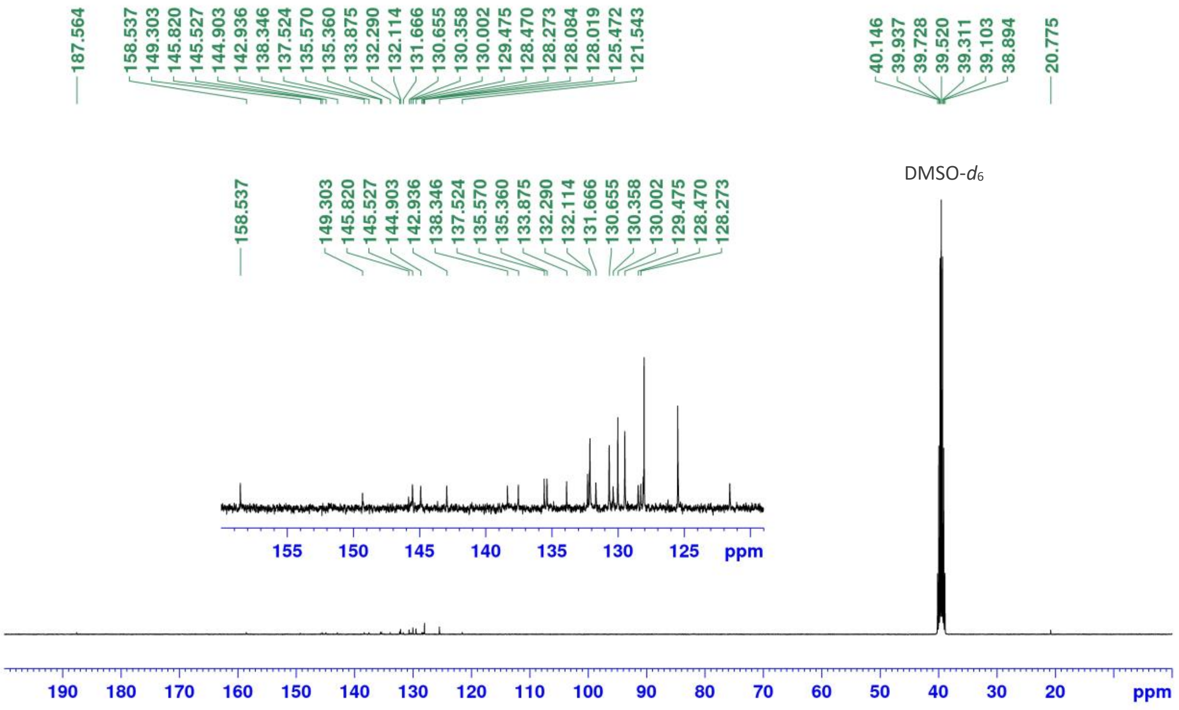

Figure S55. ^{13}C NMR (100 MHz) spectrum of **1s** in DMSO-d_6.

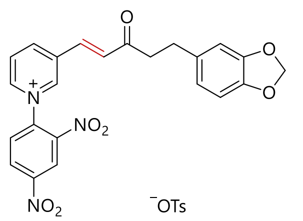

1t

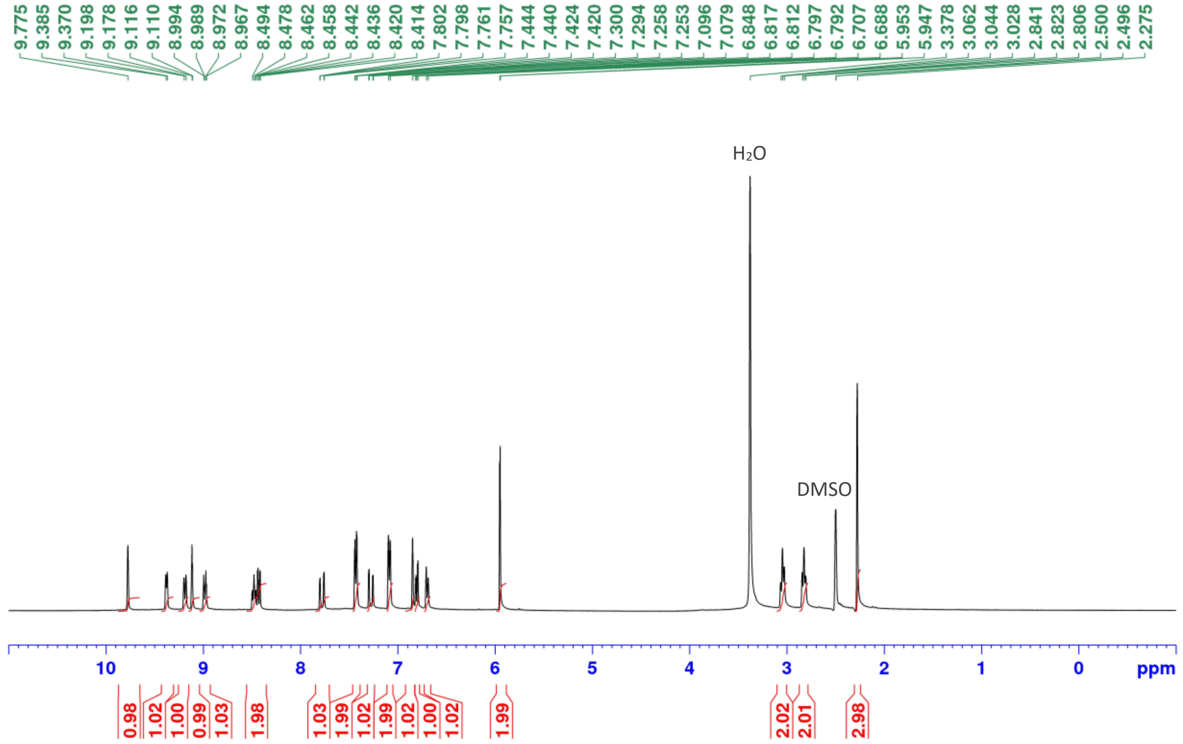

Figure S56. ^{1}H NMR (400 MHz) spectrum of **1t** in DMSO-d_6.

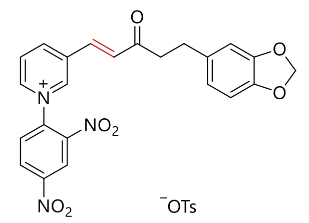

1t

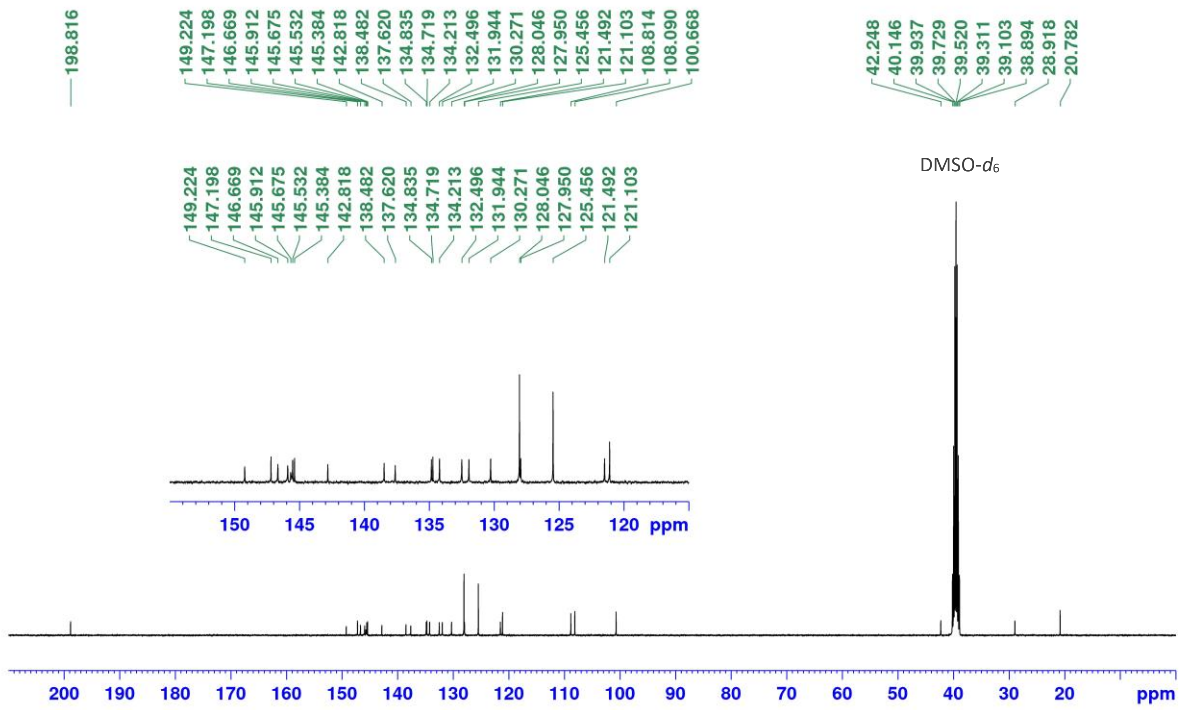

Figure S57. ^{13}C NMR (100 MHz) spectrum of **1t** in DMSO-d_6.

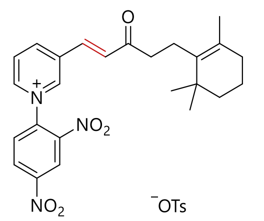

1u

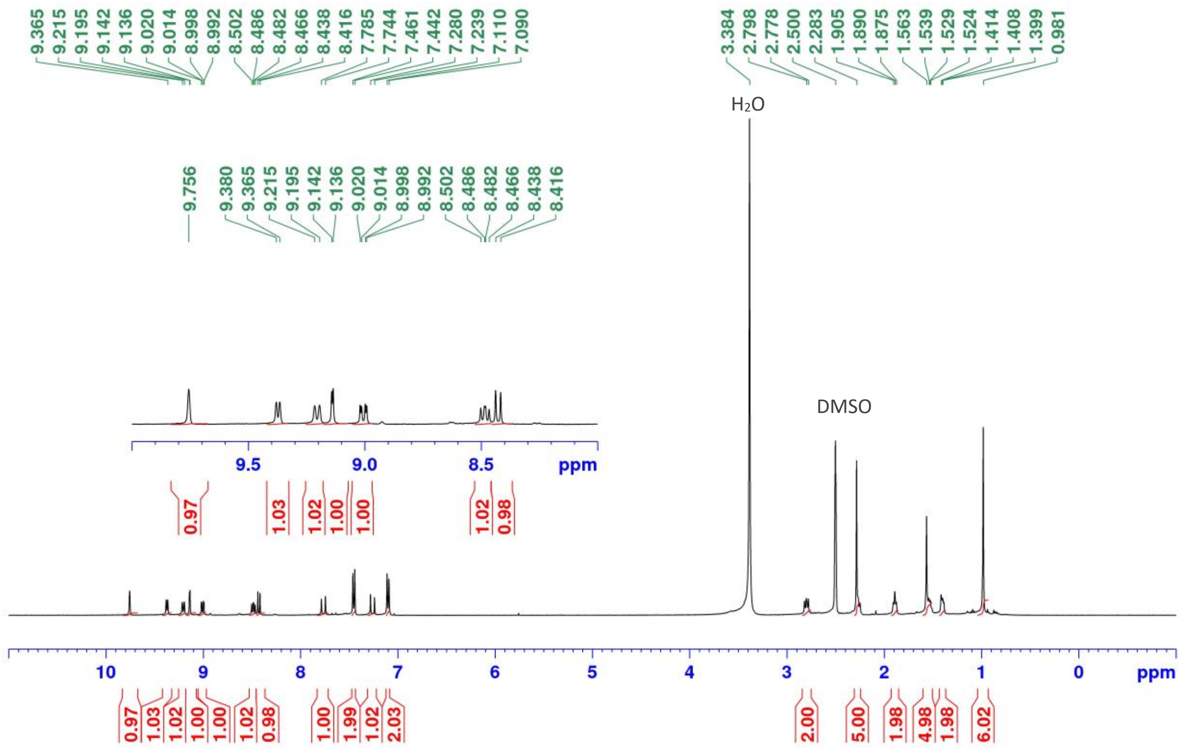

Figure S58. ^{1}H NMR (400 MHz) spectrum of **1u** in DMSO-d_6.

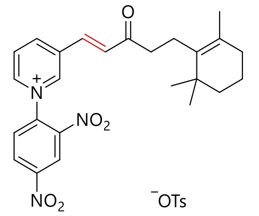

1u

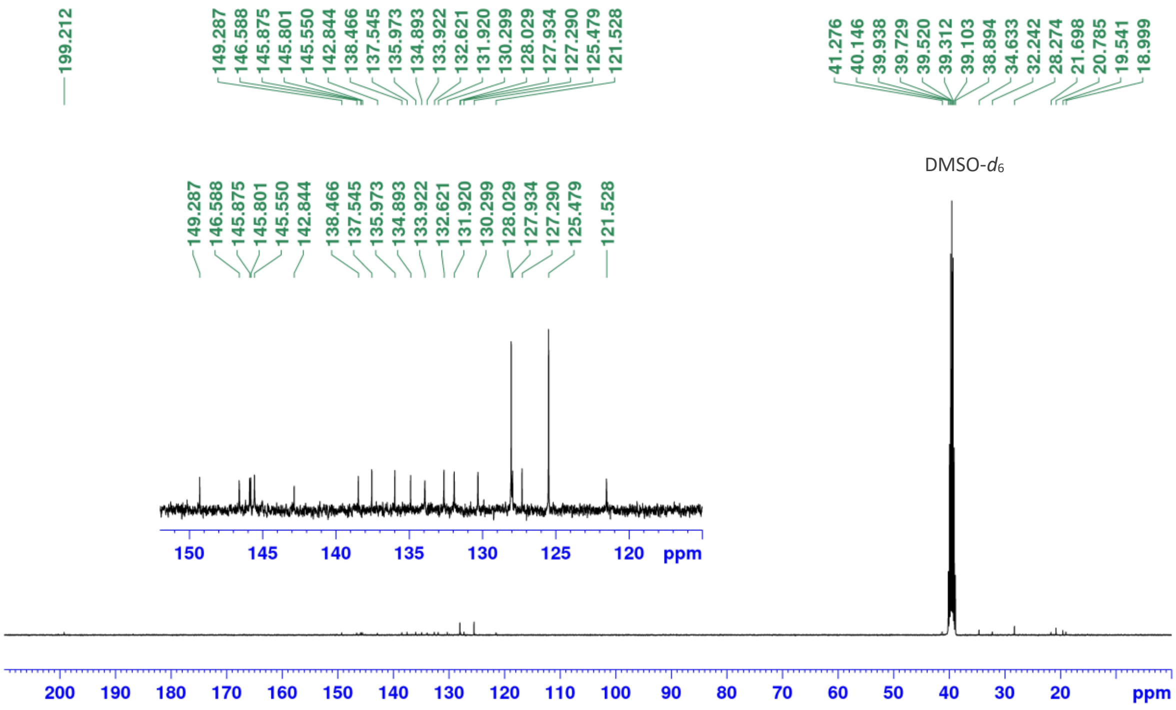

Figure S59. ^{13}C NMR (100 MHz) spectrum of **1u** in DMSO-d_6.

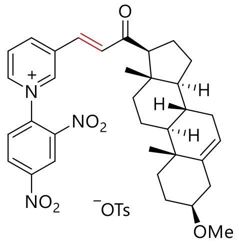

1v

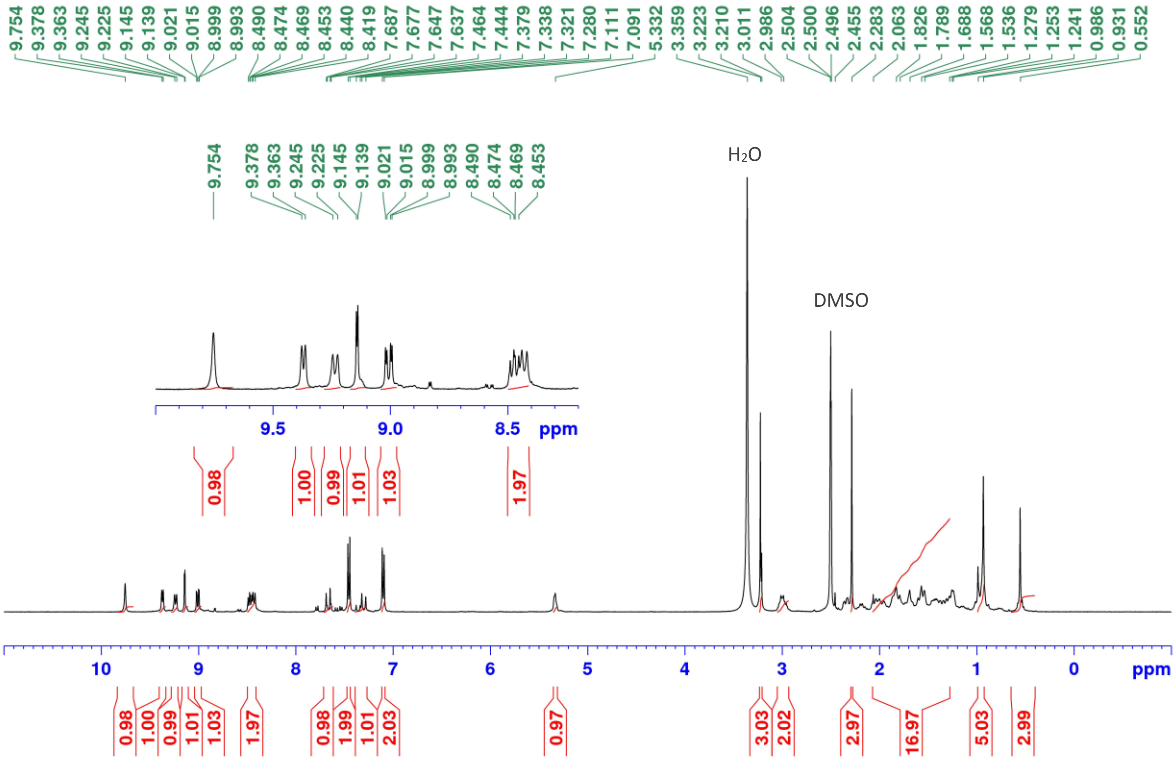

Figure S60. ^{1}H NMR (400 MHz) spectrum of **1v** in DMSO-d_6.

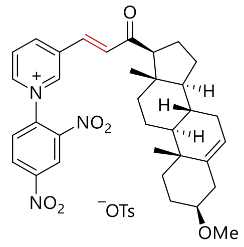

1v

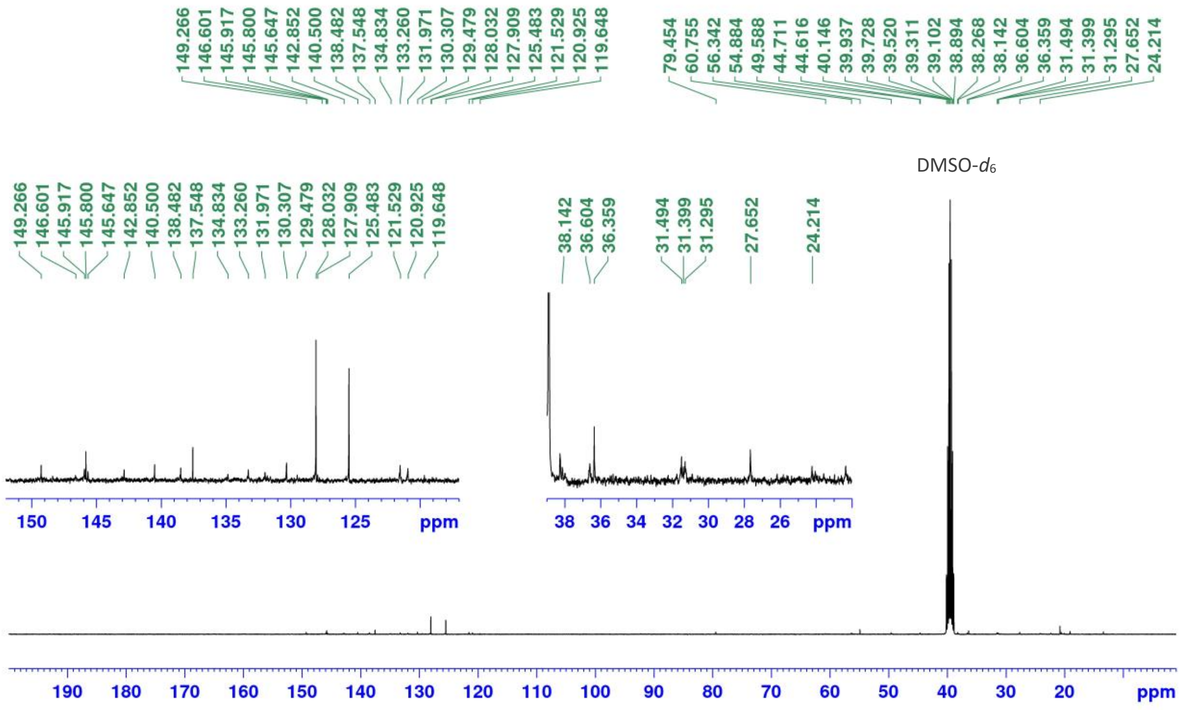

Figure S61. ^{13}C NMR (100 MHz) spectrum of **1v** in DMSO-d_6.

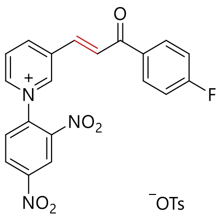

1w

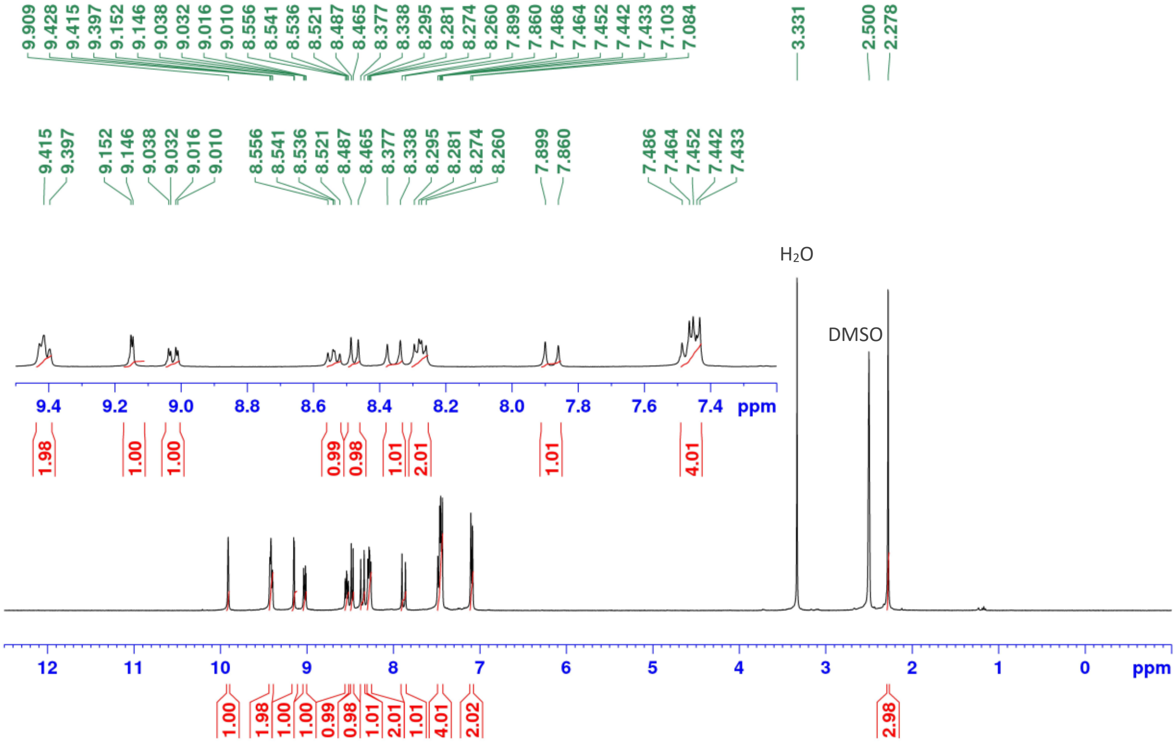

Figure S62. ^{1}H NMR (400 MHz) spectrum of **1w** in DMSO-d_6.

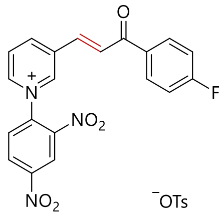

1w

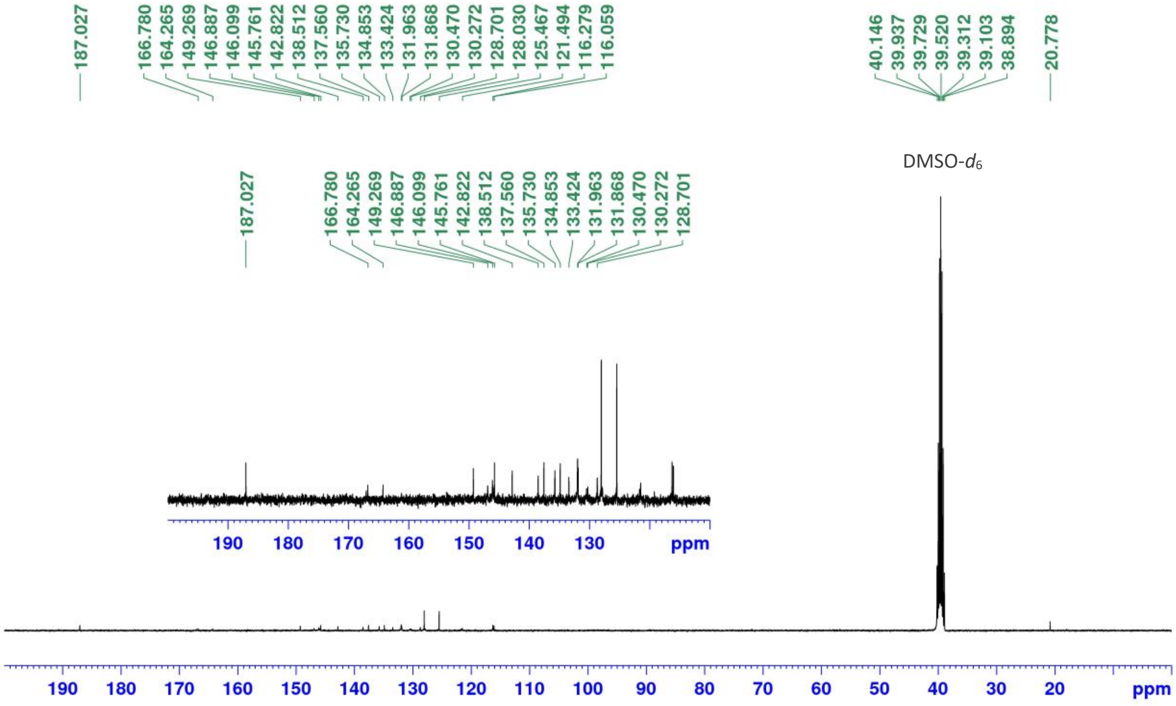

Figure S63. ^{13}C NMR (100 MHz) spectrum of **1w** in DMSO-d_6.

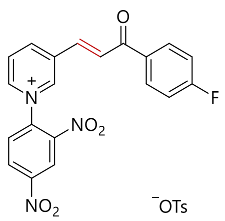

1w

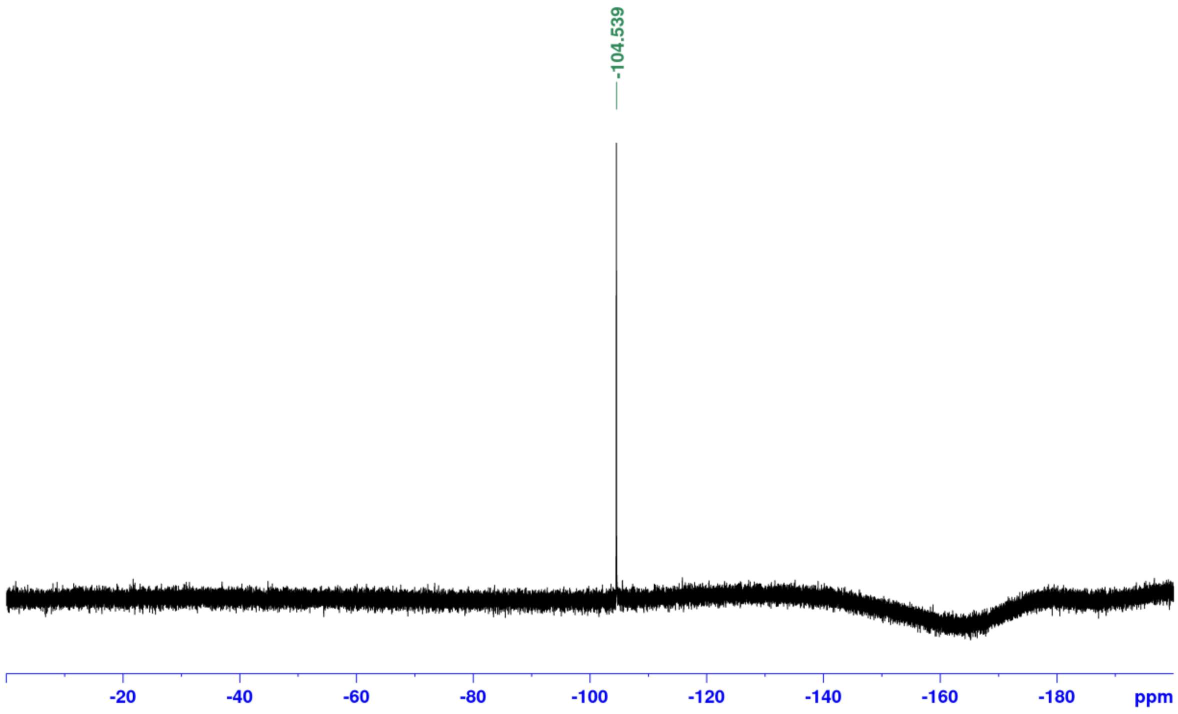

Figure S64. ^{19}F NMR (376 MHz) spectrum of **1w** in DMSO-d_6.

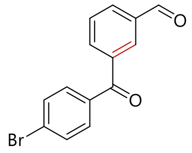

2a

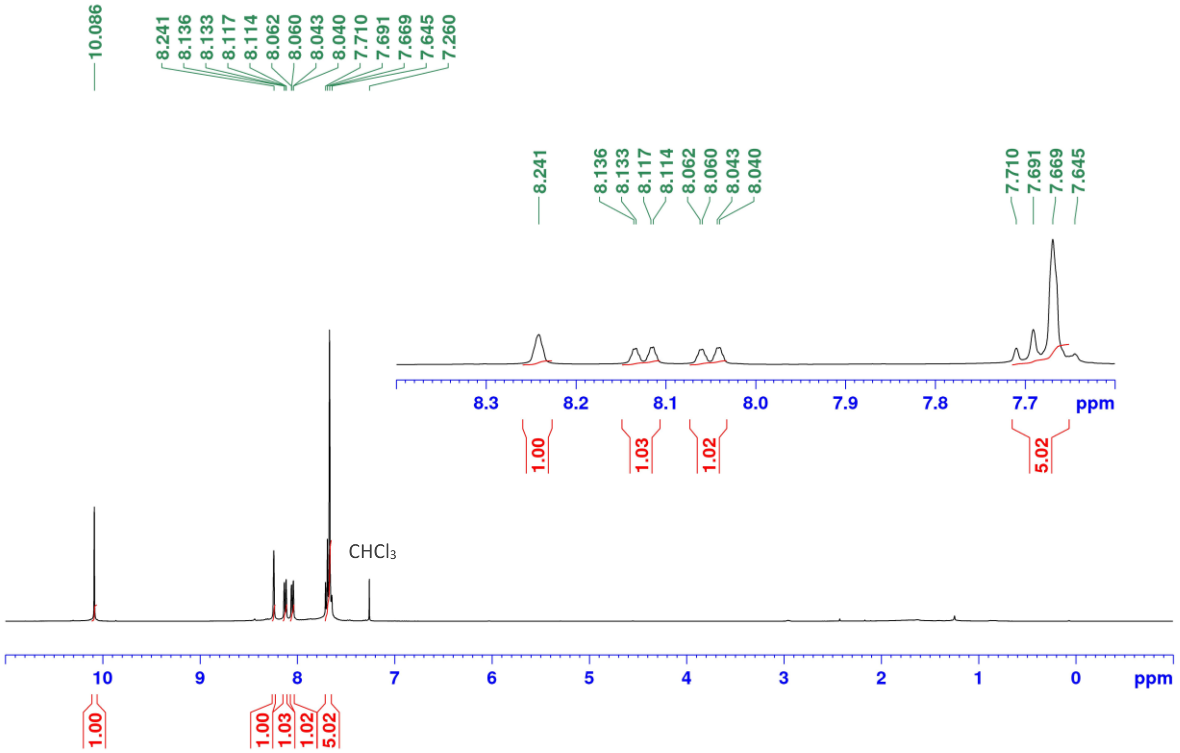

Figure S65. ^{1}H NMR (400 MHz) spectrum of **2a** in $CDCl_3$.

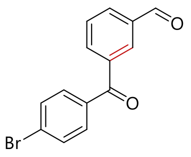

2a

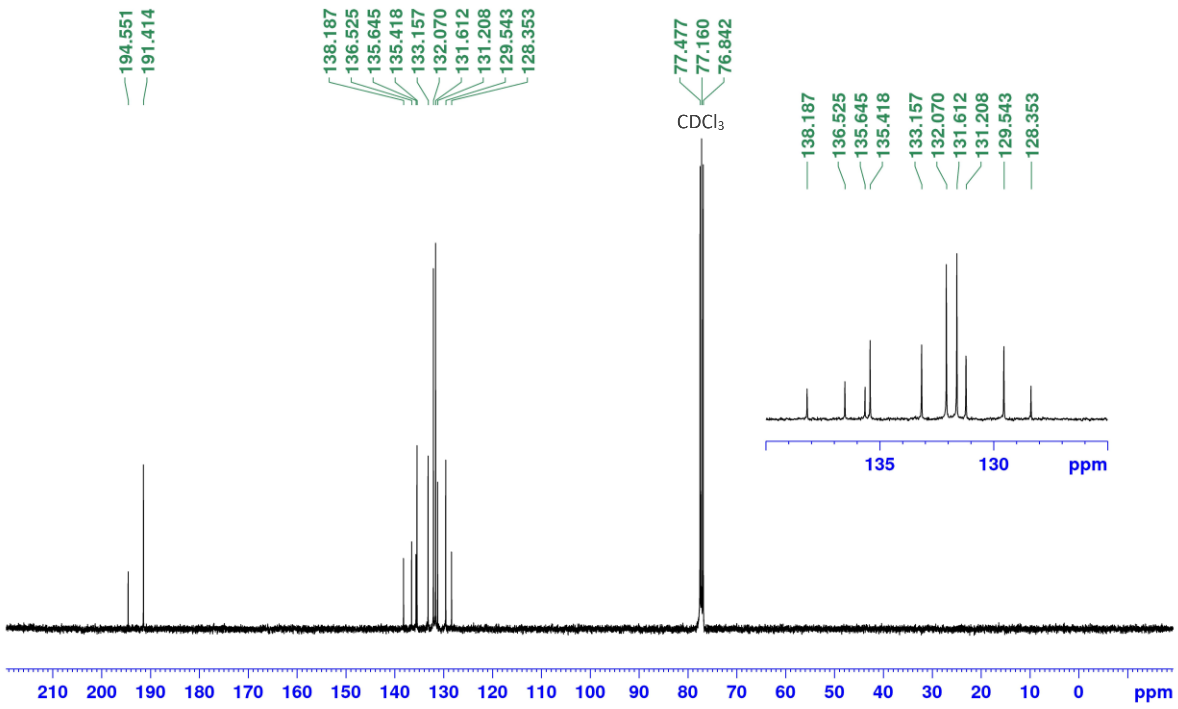

Figure S66. ^{13}C NMR (100 MHz) spectrum of **2a** in $CDCl_3$.

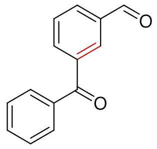

2b

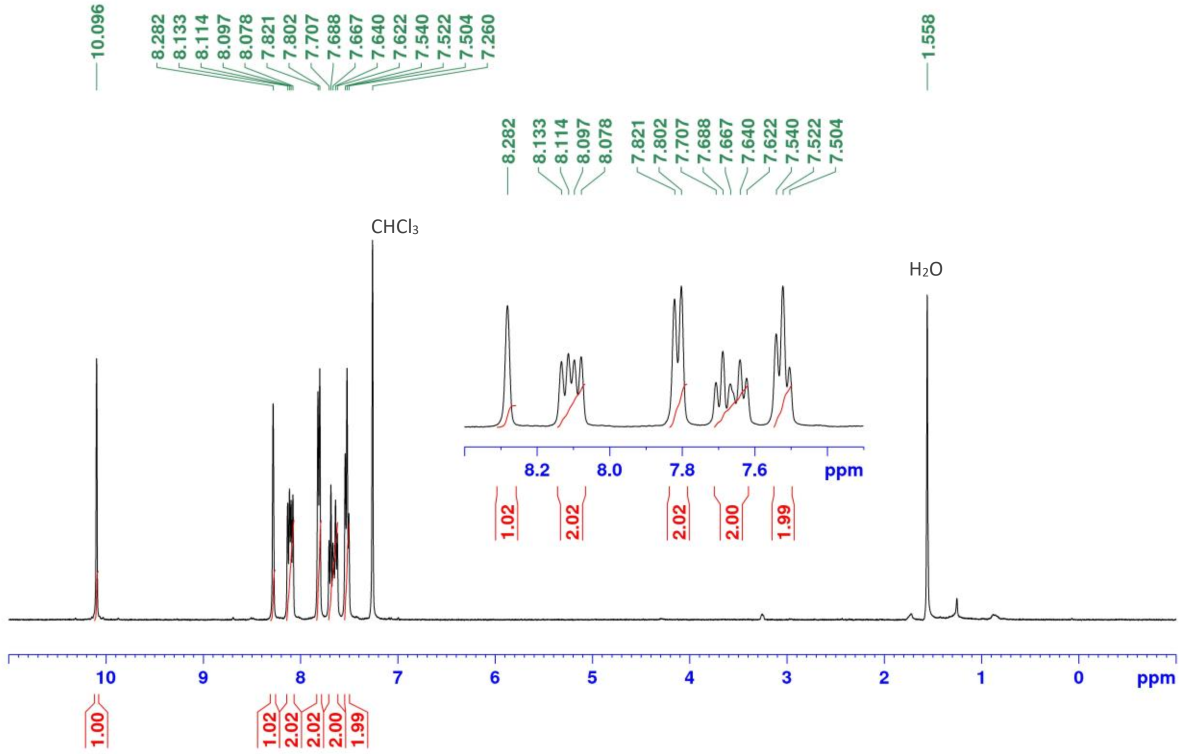

Figure S67. ^{1}H NMR (400 MHz) spectrum of **2b** in $CDCl_3$.

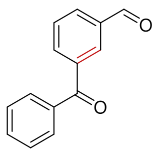

2b

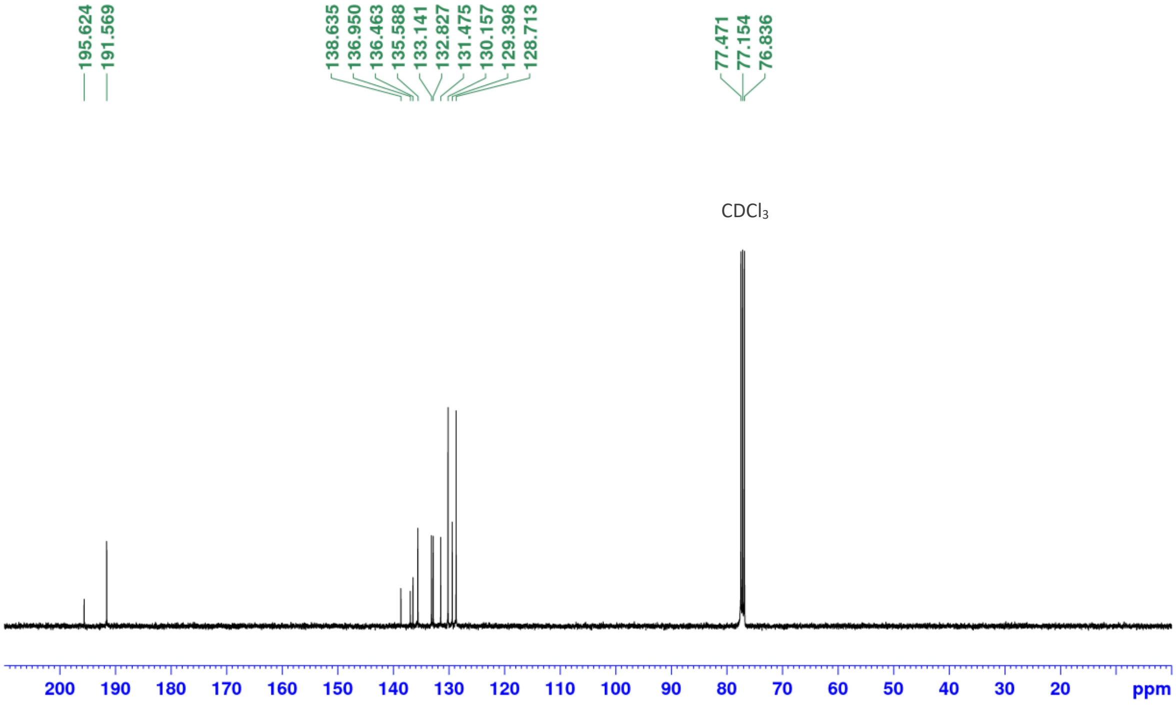

Figure S68. ^{13}C NMR (100 MHz) spectrum of **2b** in $CDCl_3$.

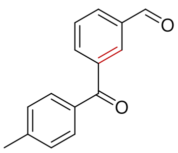

2c

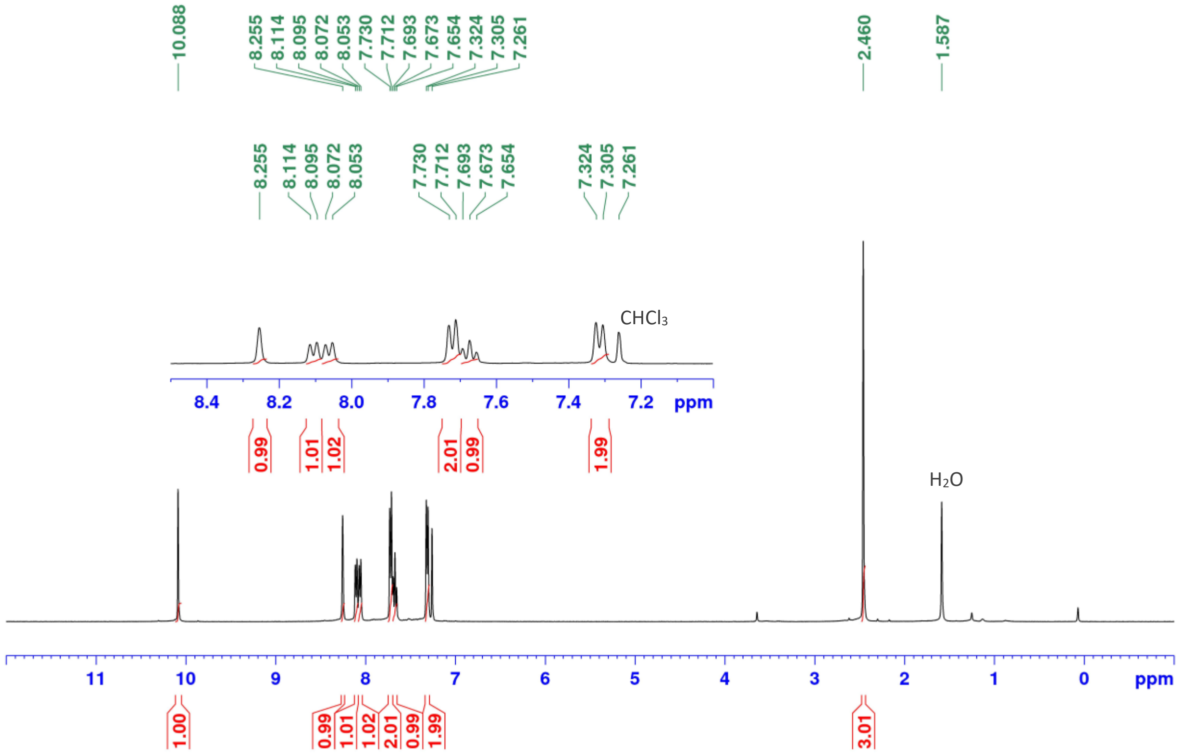

Figure S69. ^{1}H NMR (400 MHz) spectrum of **2c** in $CDCl_3$.

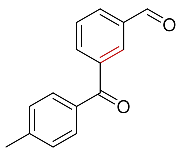

2c

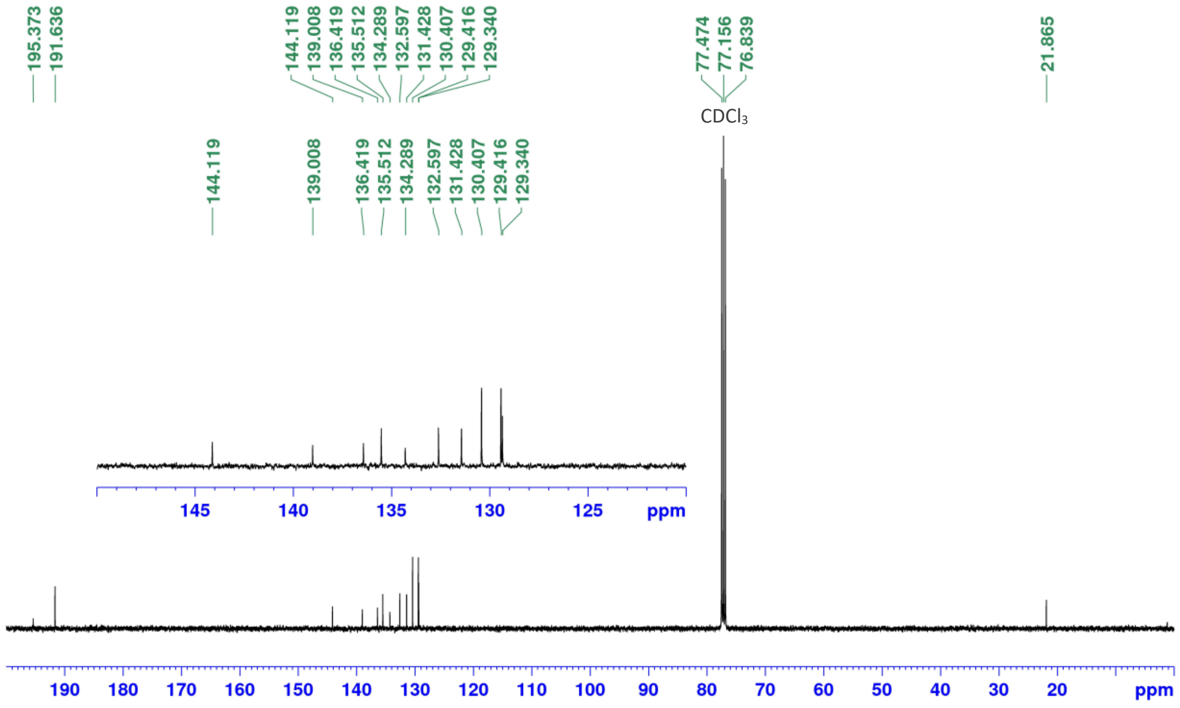

Figure S70. ^{13}C NMR (100 MHz) spectrum of **2c** in $CDCl_3$.

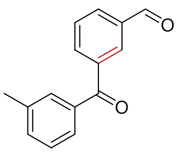

2d

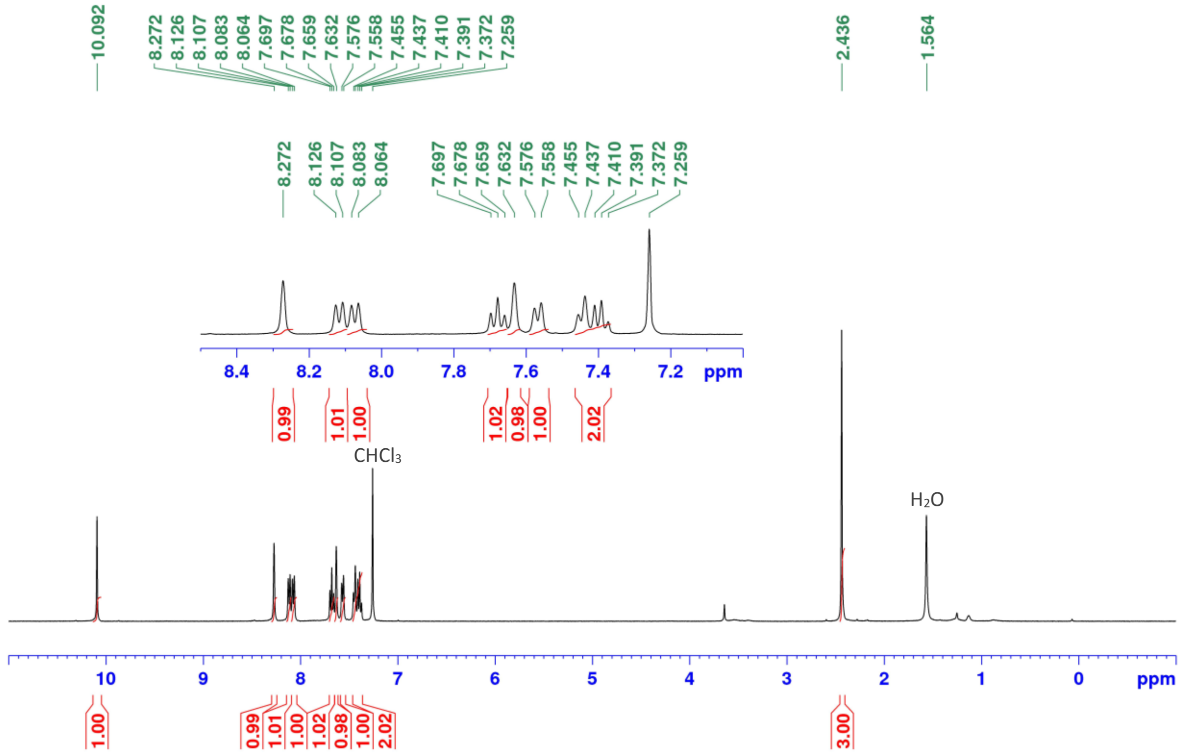

Figure S71. ^{1}H NMR (400 MHz) spectrum of **2d** in $CDCl_3$.

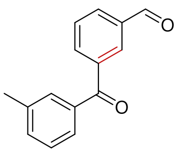

2d

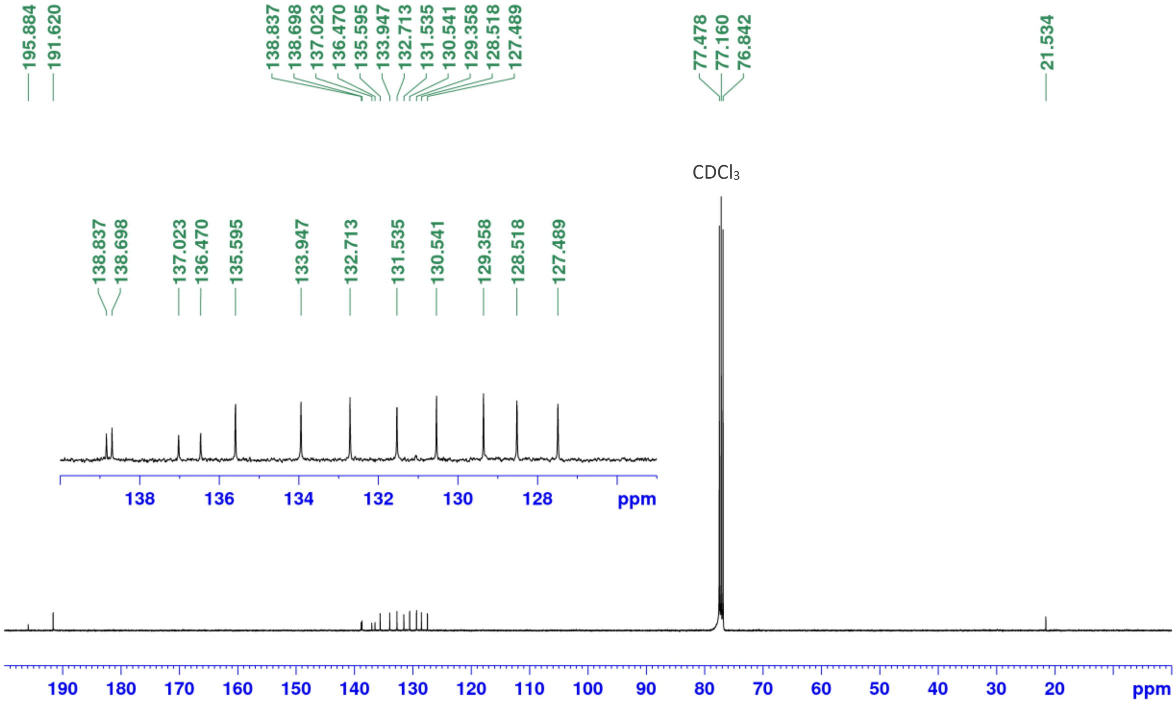

Figure S72. ^{13}C NMR (100 MHz) spectrum of **2d** in $CDCl_3$.

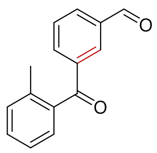

2e

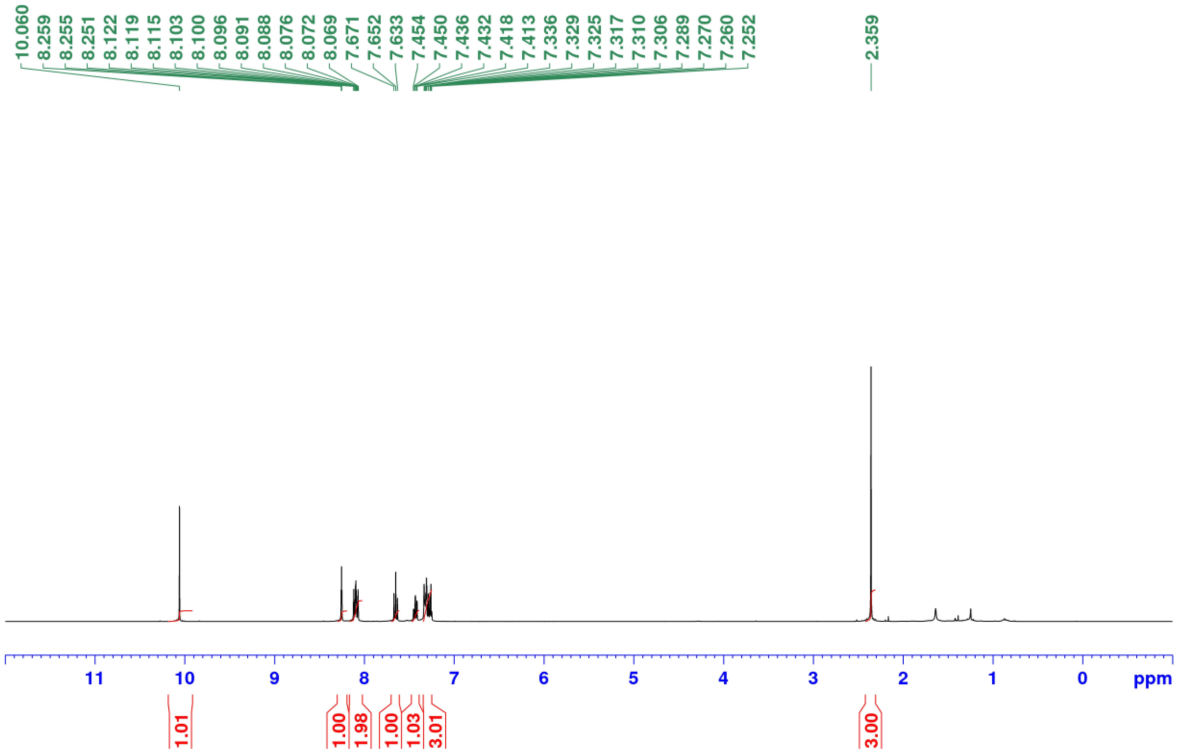

Figure S73. ^{1}H NMR (400 MHz) spectrum of **2e**n $CDCl_3$.

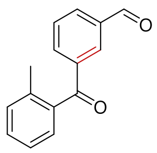

2e

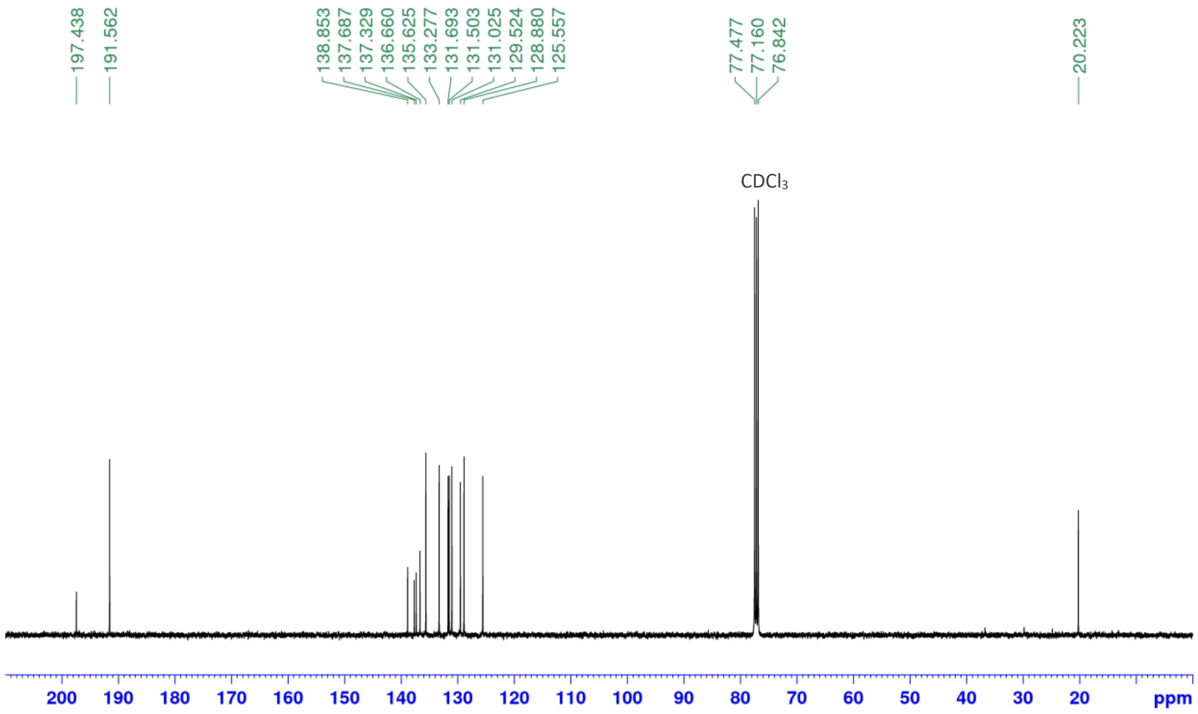

Figure S74. ^{13}C NMR (100 MHz) spectrum of **2e** $CDCl_3$.

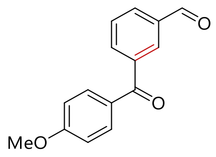

2f

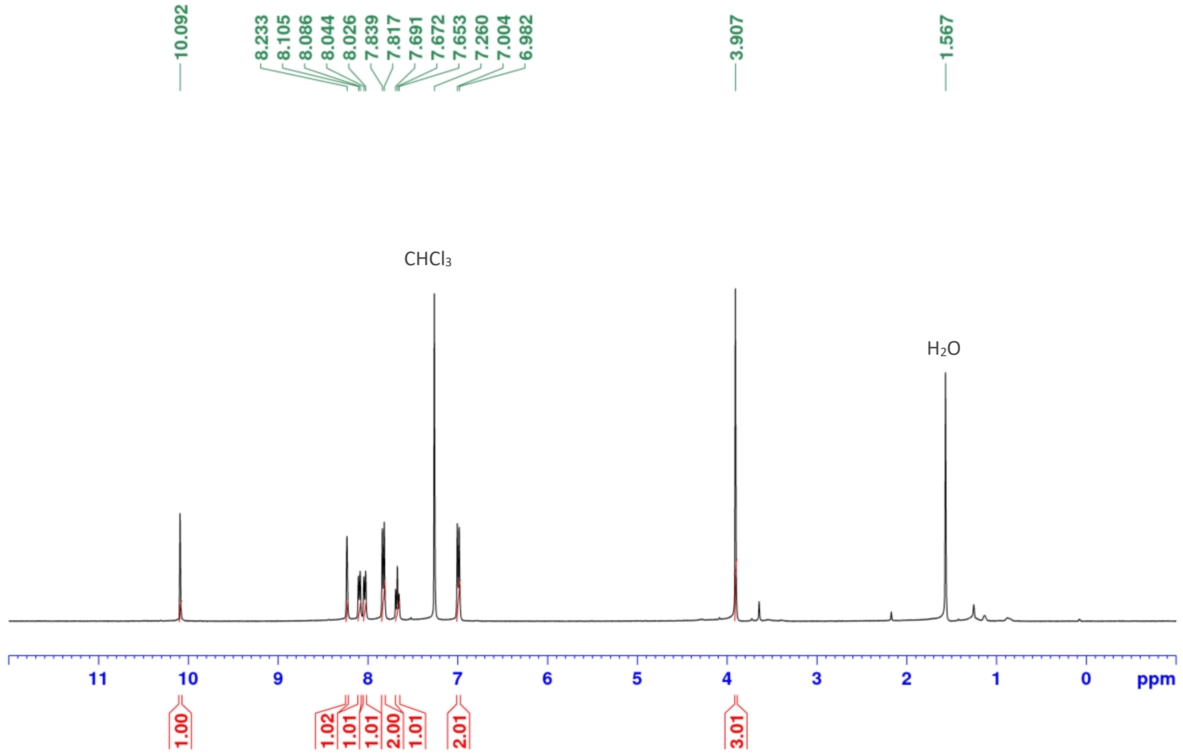

Figure S75. ^{1}H NMR (400 MHz) spectrum of **2f** $CDCl_3$.

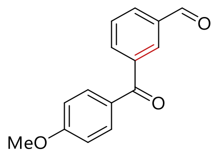

2f

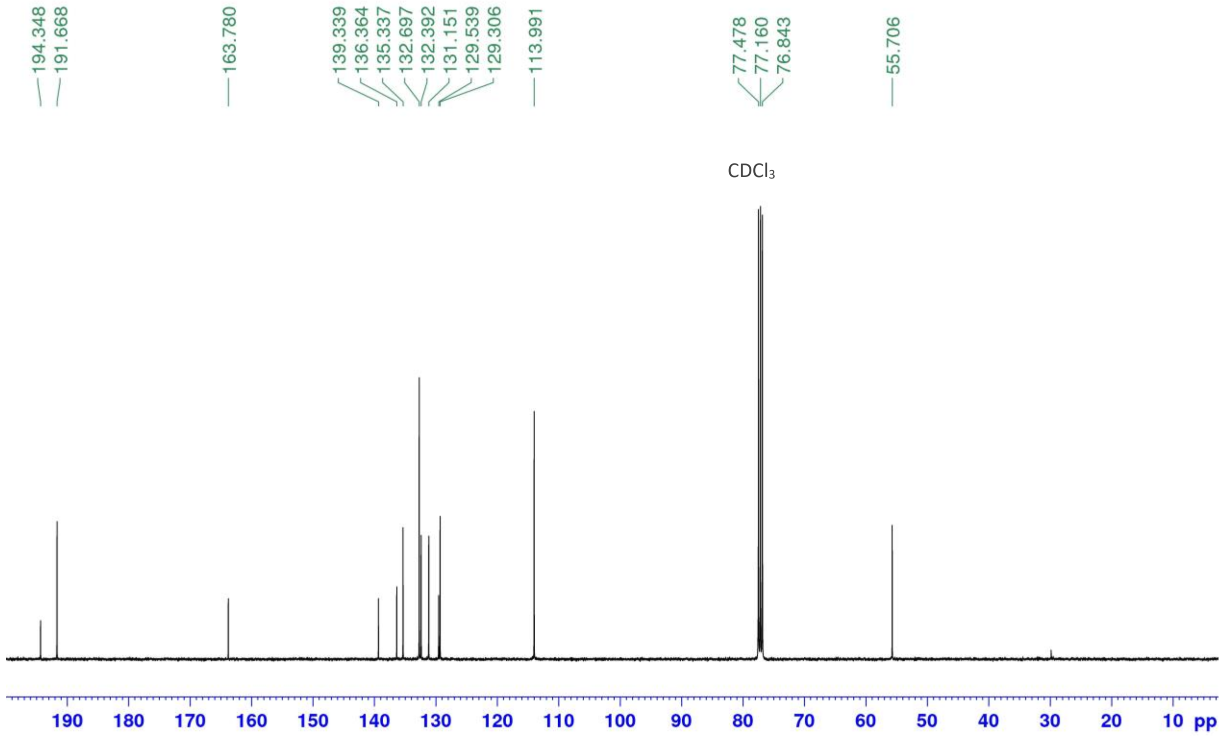

Figure S76. ^{13}C NMR (100 MHz) spectrum of **2f** $CDCl_3$.

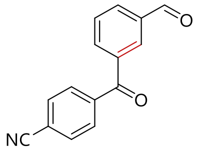

2g

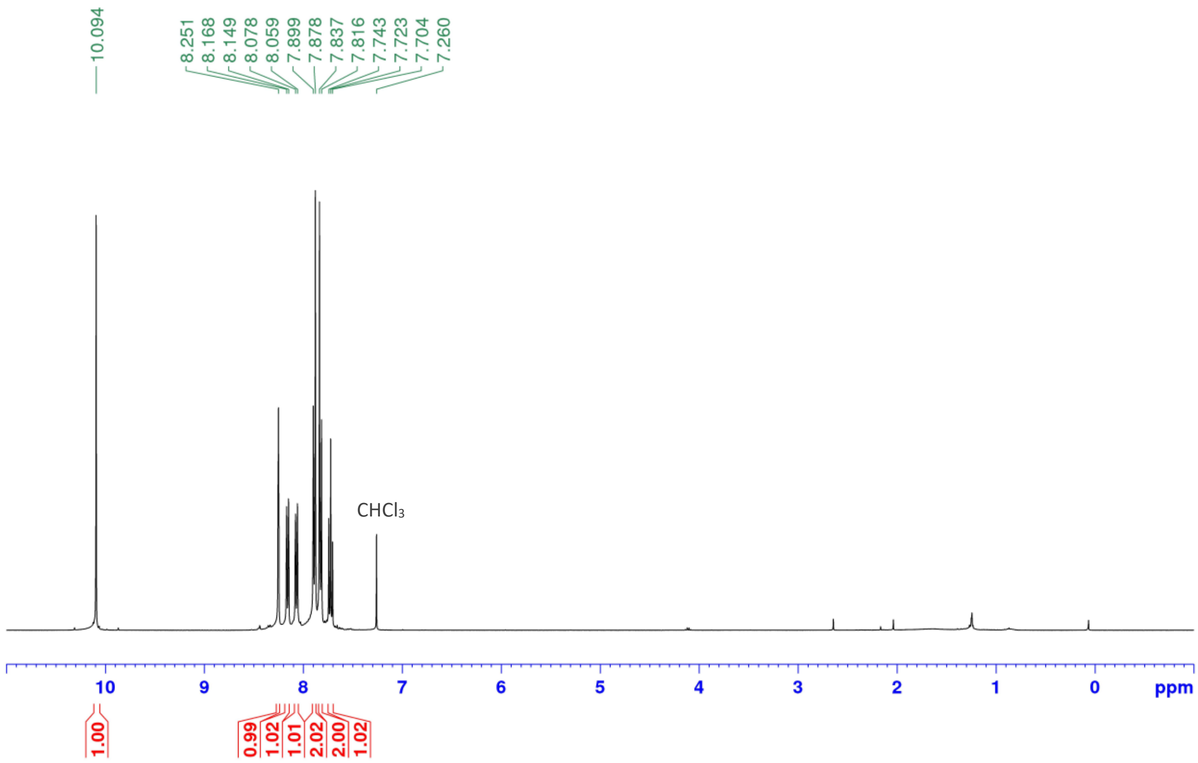

Figure S77. ^{1}H NMR (400 MHz) spectrum of **2g** in $CDCl_3$.

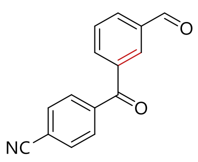

2g

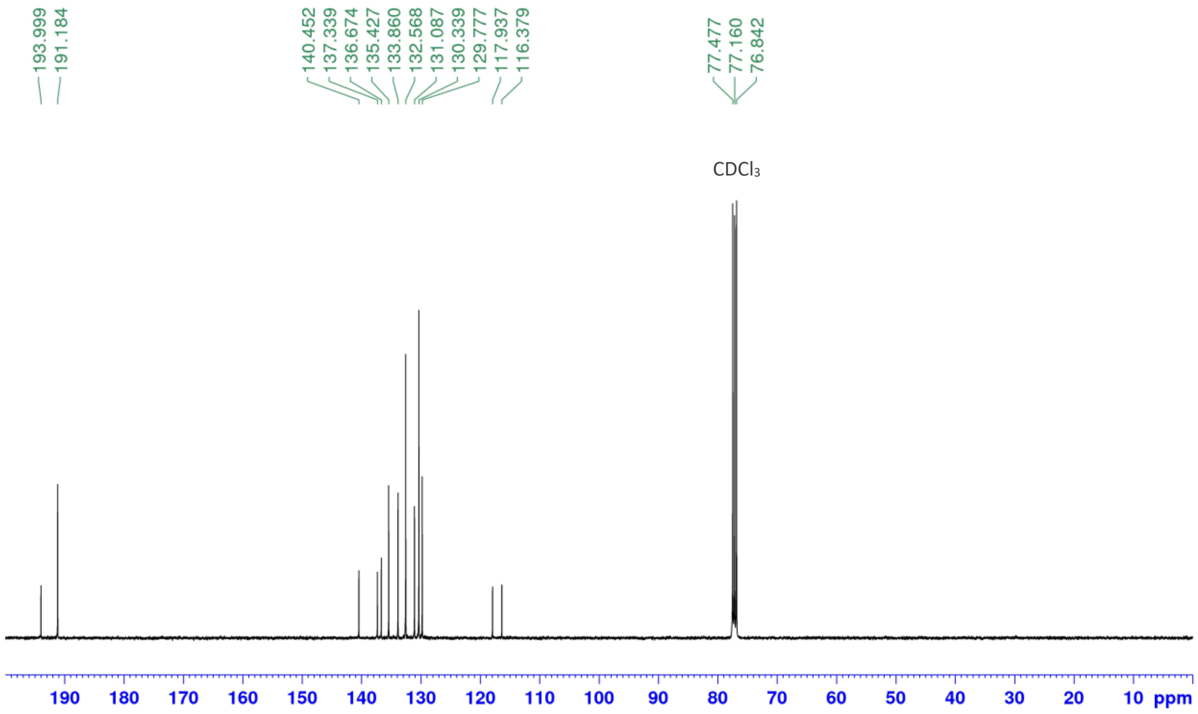

Figure S78. ^{13}C NMR (100 MHz) spectrum of **2g** in $CDCl_3$.

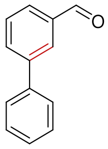

2h

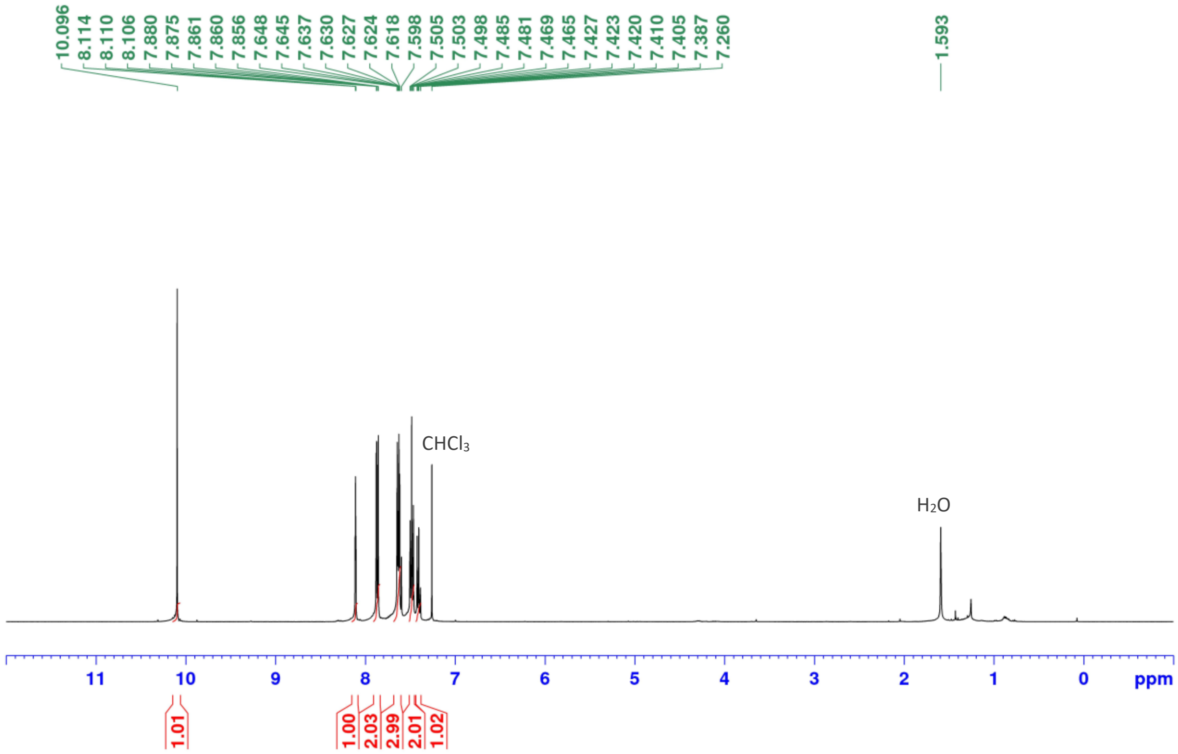

Figure S79. ^{1}H NMR (400 MHz) spectrum of **2h** in $CDCl_3$.

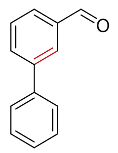

2h

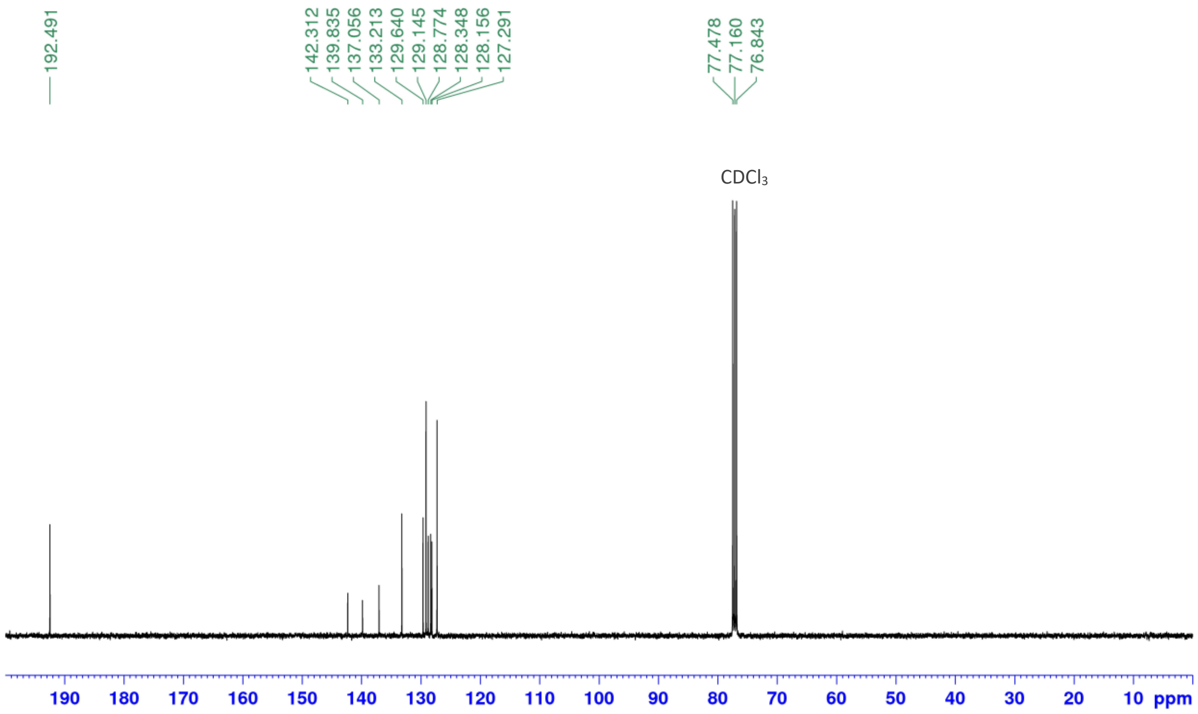

Figure S80. ^{13}C NMR (100 MHz) spectrum of **2h** in $CDCl_3$.

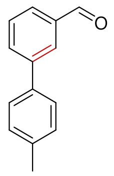

2i

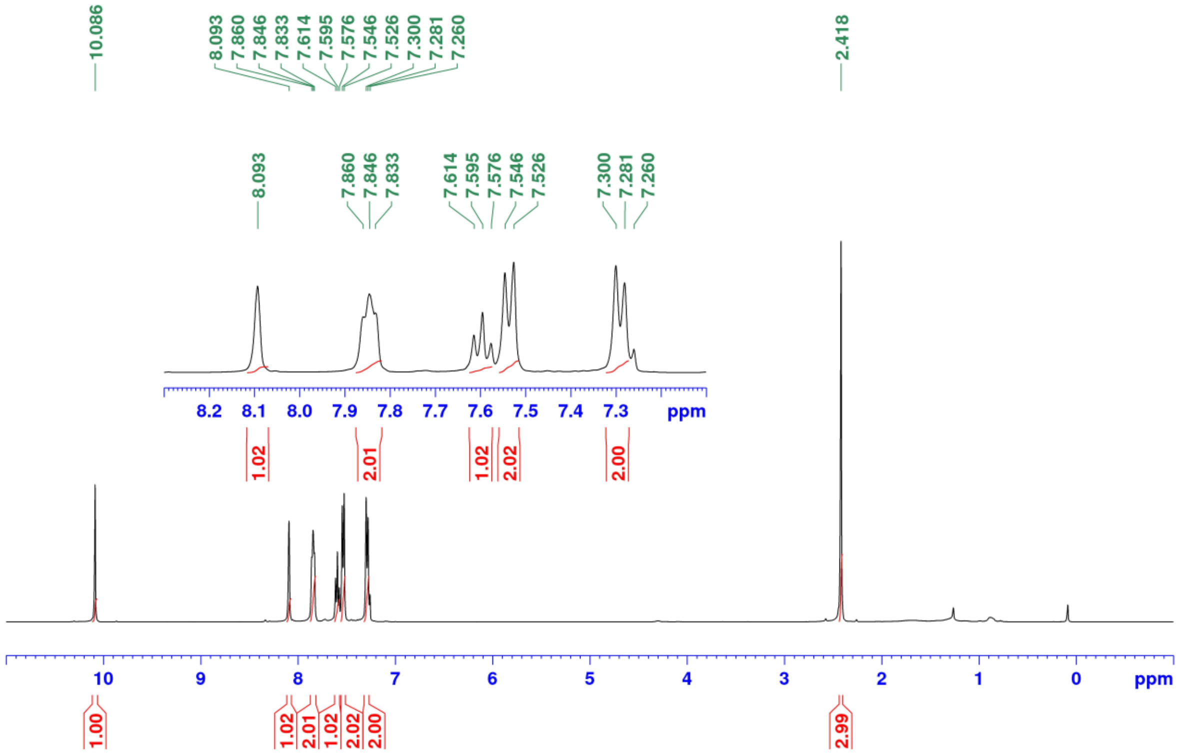

Figure S81. ^{1}H NMR (400 MHz) spectrum of **2i** in $CDCl_3$.

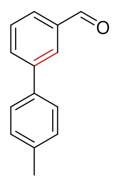

2i

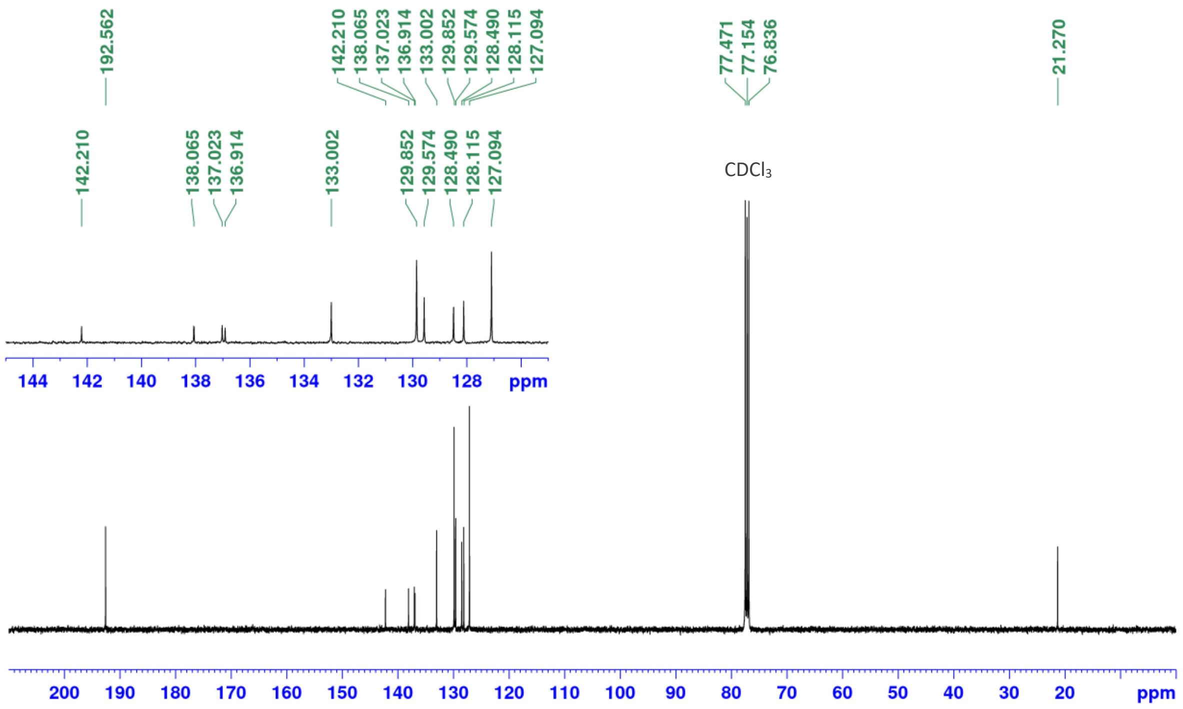

Figure S82. ^{13}C NMR (100 MHz) spectrum of **2i** in $CDCl_3$.

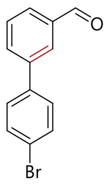

2j

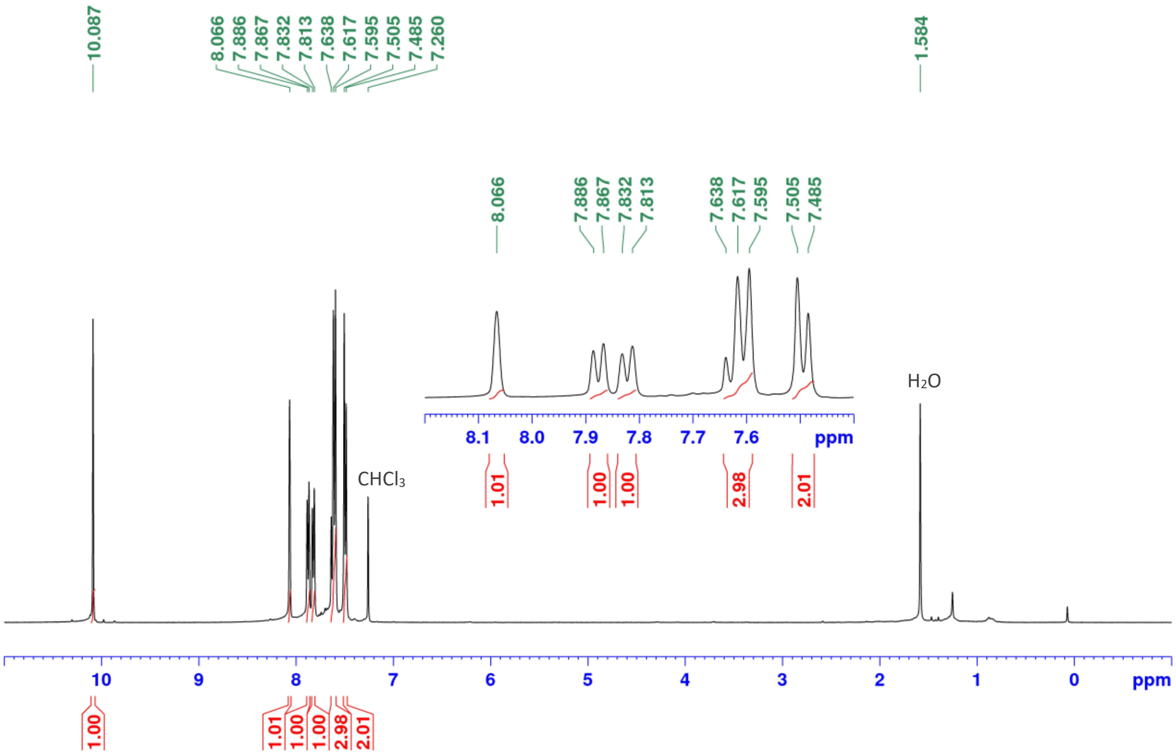

Figure S83. ^{1}H NMR (400 MHz) spectrum of **2j** in $CDCl_3$.

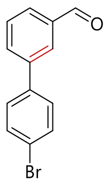

2j

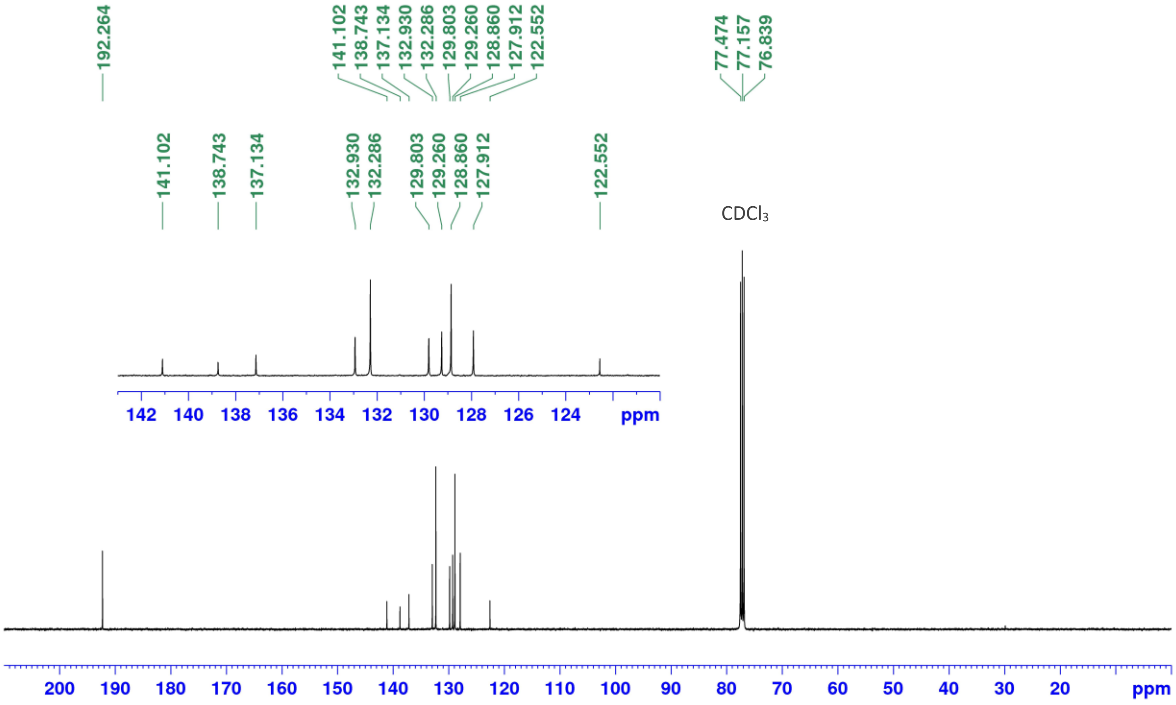

Figure S84. ^{13}C NMR (100 MHz) spectrum of **2j** in $CDCl_3$.

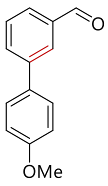

2k

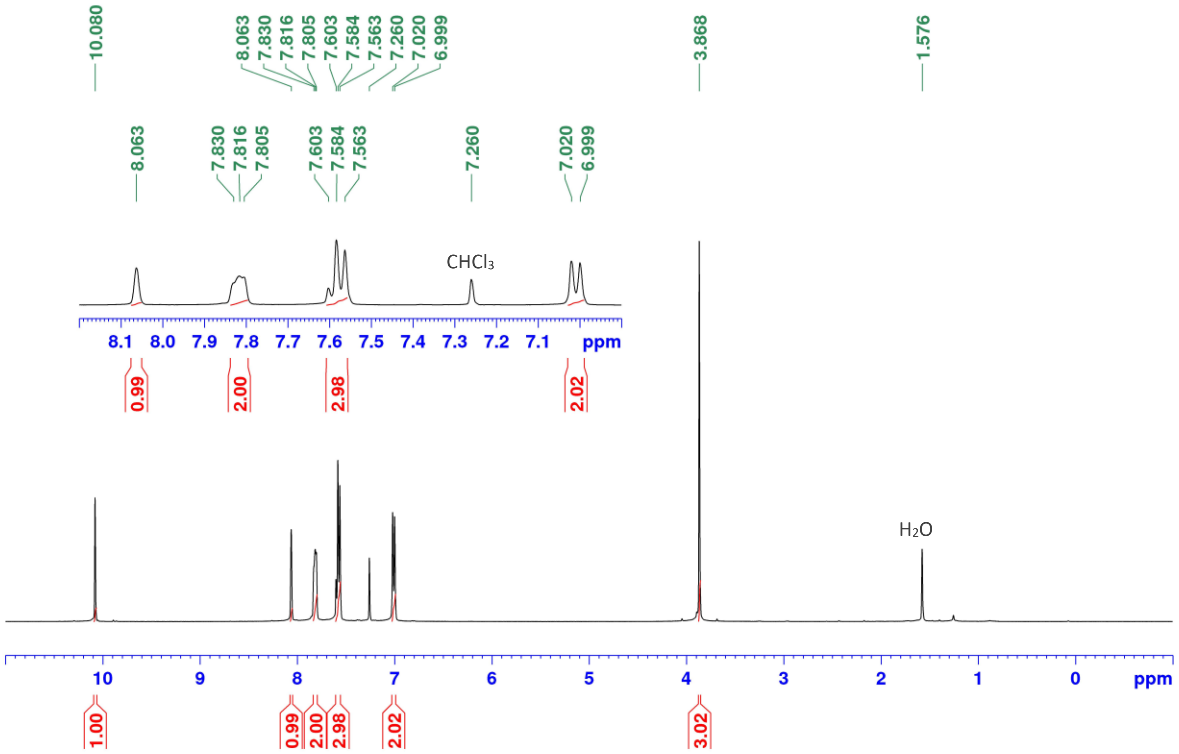

Figure S85. ^{1}H NMR (400 MHz) spectrum of **2k** in $CDCl_3$.

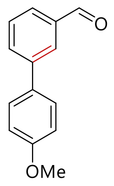

2k

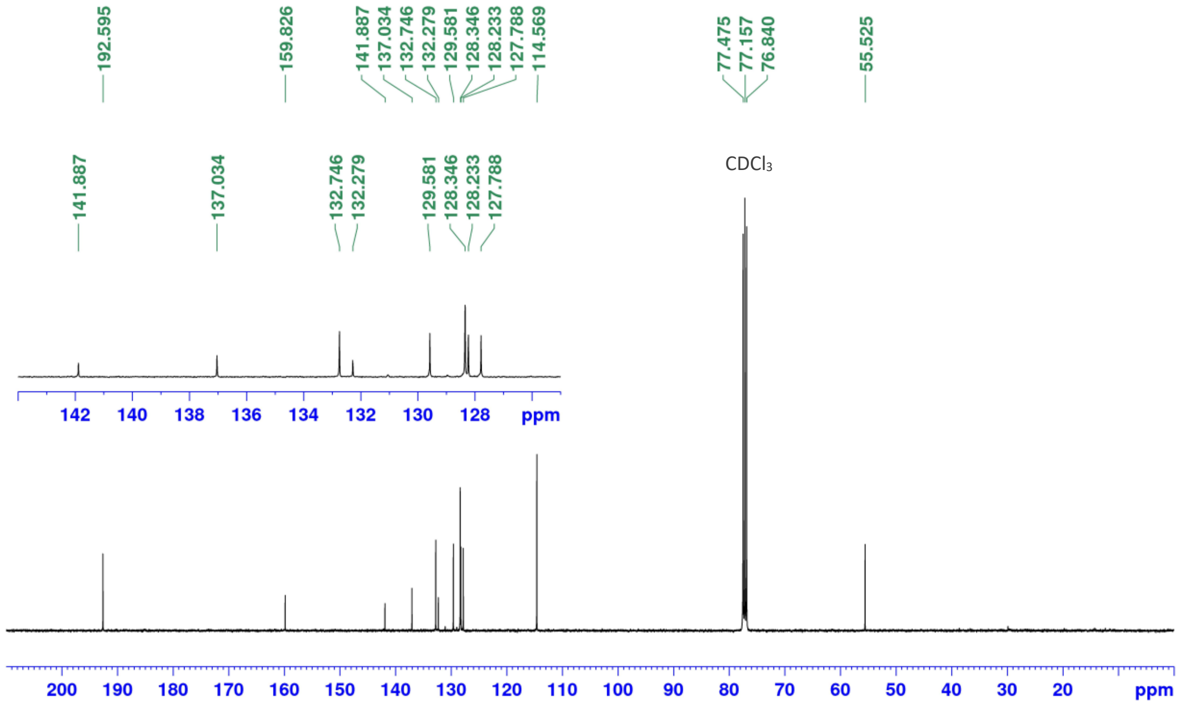

Figure S86. ^{13}C NMR (100 MHz) spectrum of **2k** in $CDCl_3$.

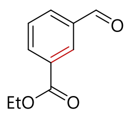

2l

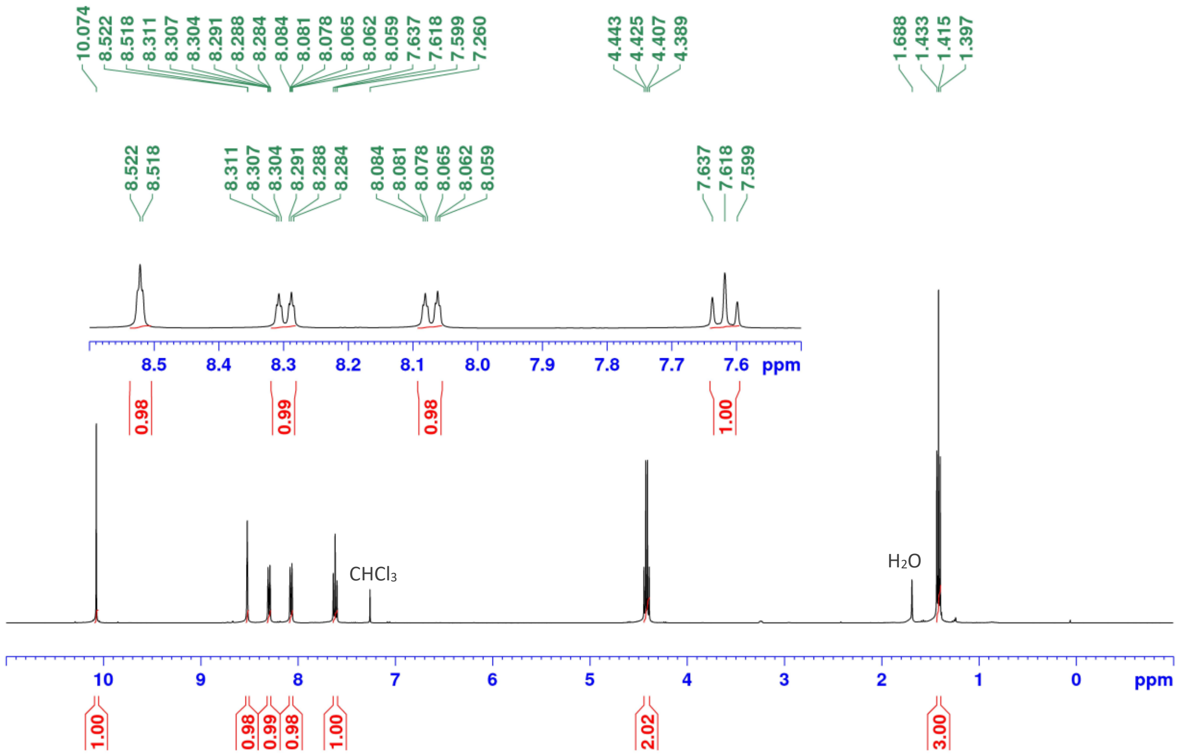

Figure S87. ^{1}H NMR (400 MHz) spectrum of **2l** in $CDCl_3$.

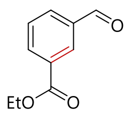

2l

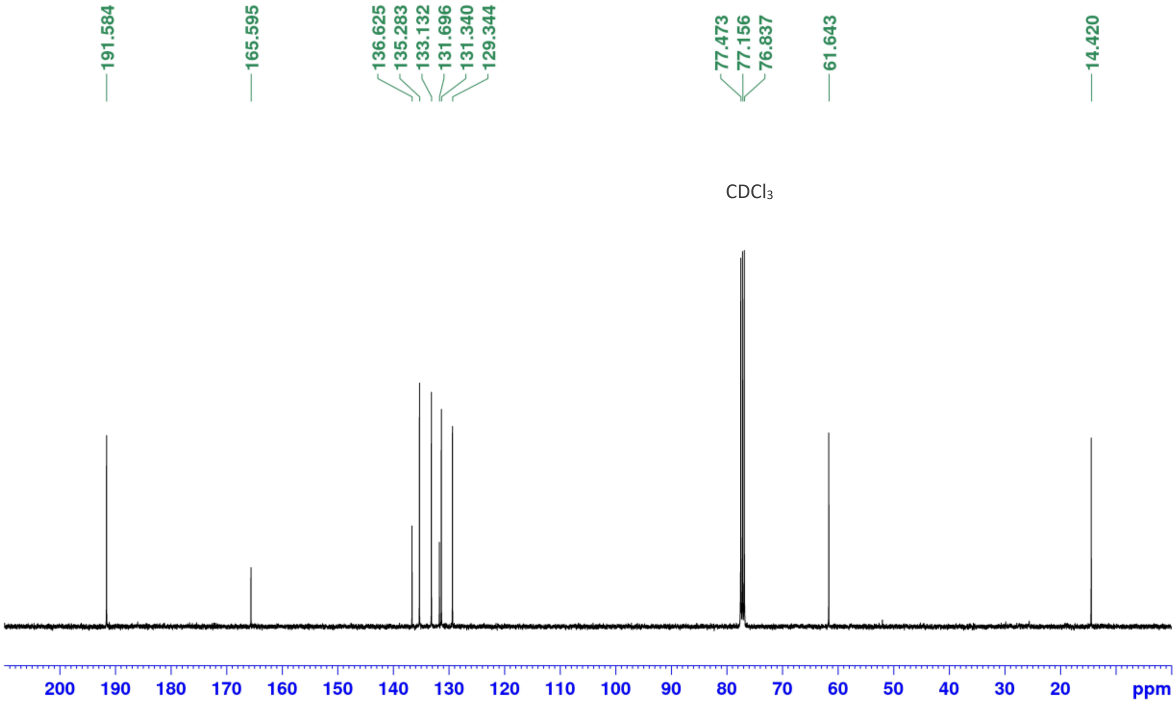

Figure S88. ^{13}C NMR (100 MHz) spectrum of **2l** in $CDCl_3$.

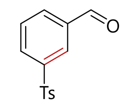

2m

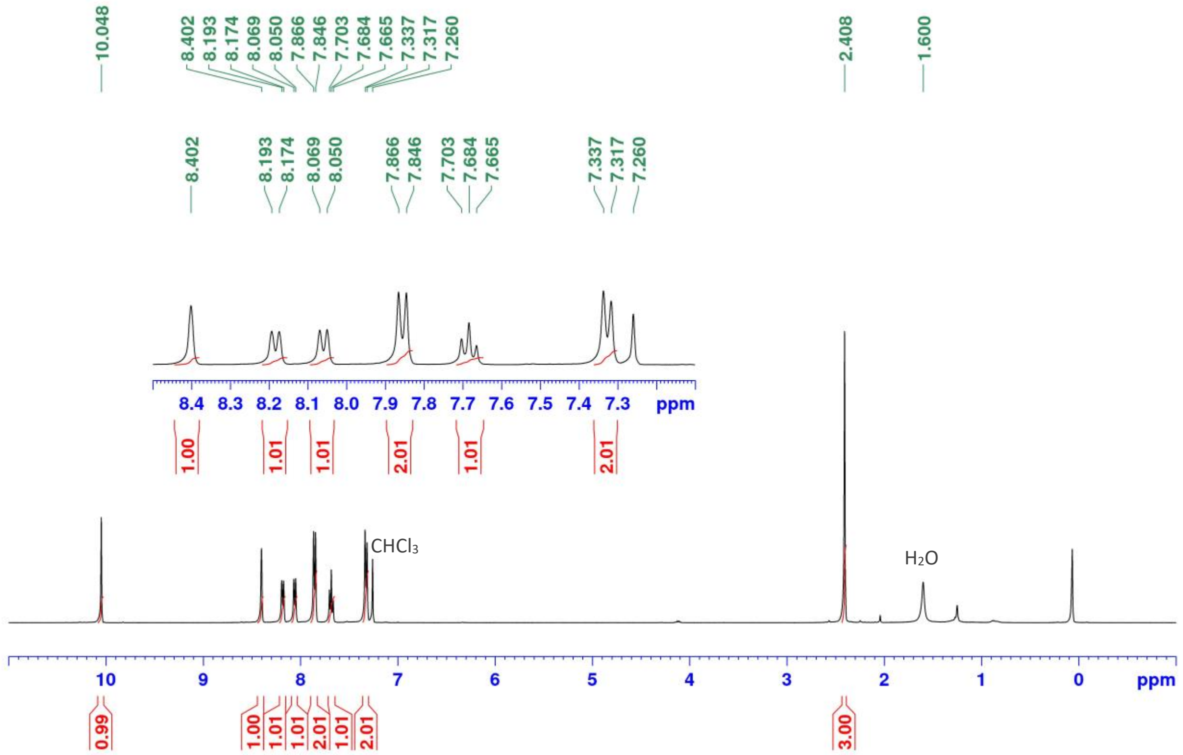

Figure S89. ^{1}H NMR (400 MHz) spectrum of **2m** in $CDCl_3$.

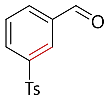

2m

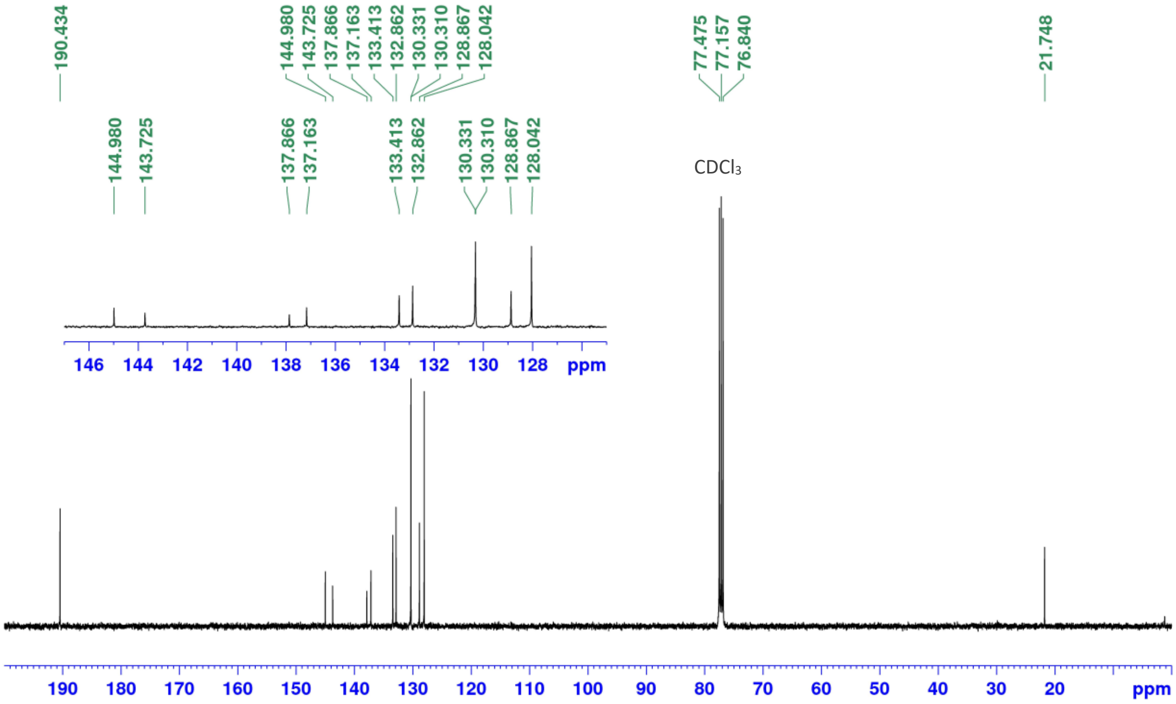

Figure S90. ^{13}C NMR (100 MHz) spectrum of **2m** in $CDCl_3$.

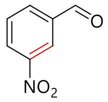

2n

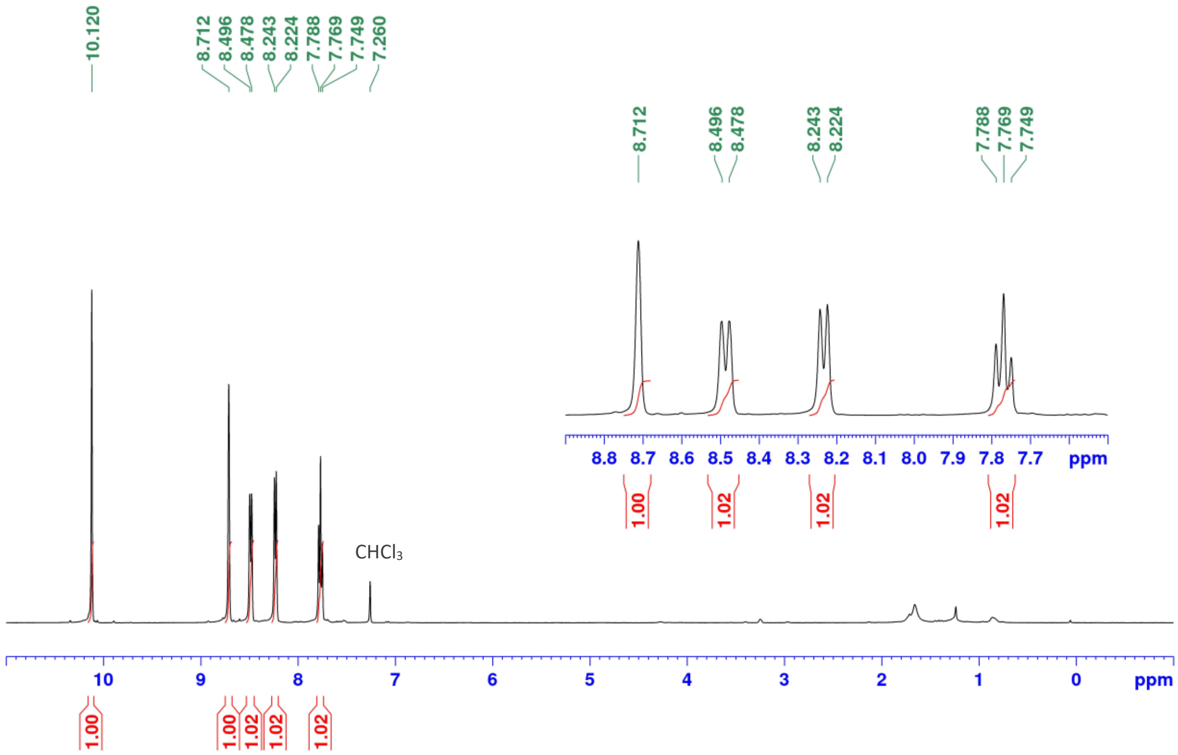

Figure S91. ^1H NMR (400 MHz) spectrum of **2n** in $CDCl_3$.

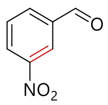

2n

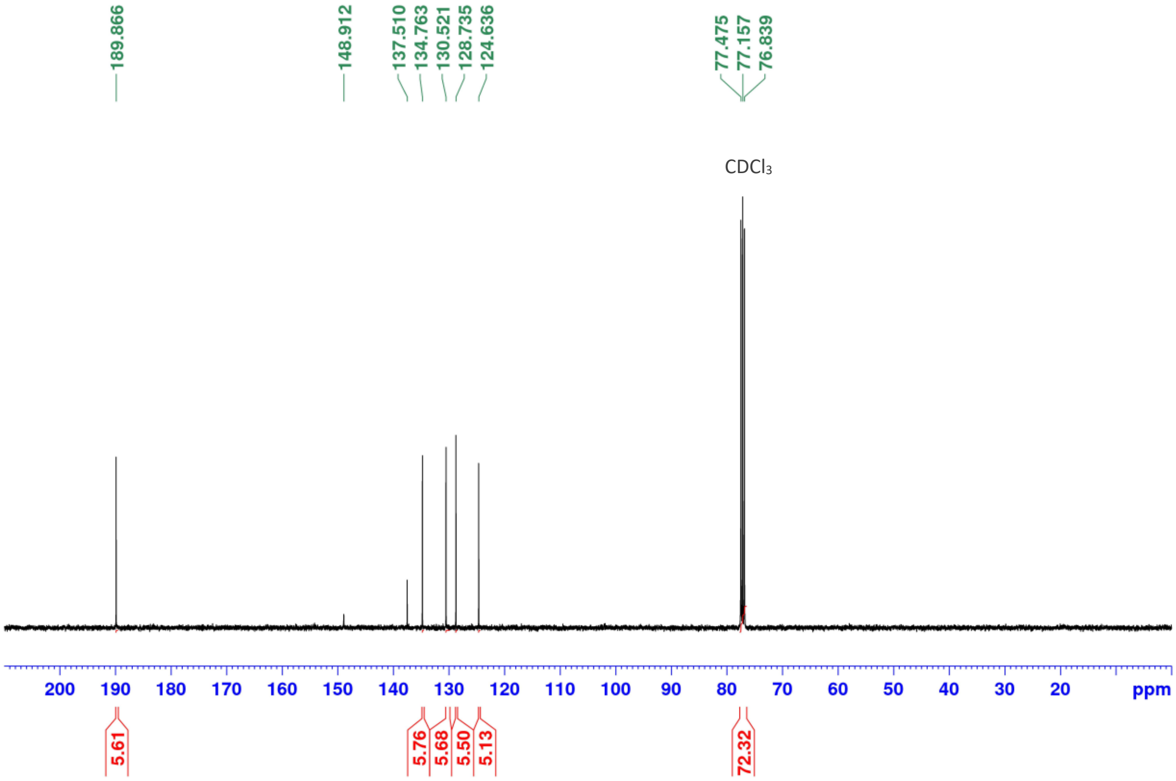

Figure S92. ^{13}C NMR (100 MHz) spectrum of **2n** in $CDCl_3$.

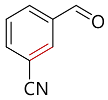

2o

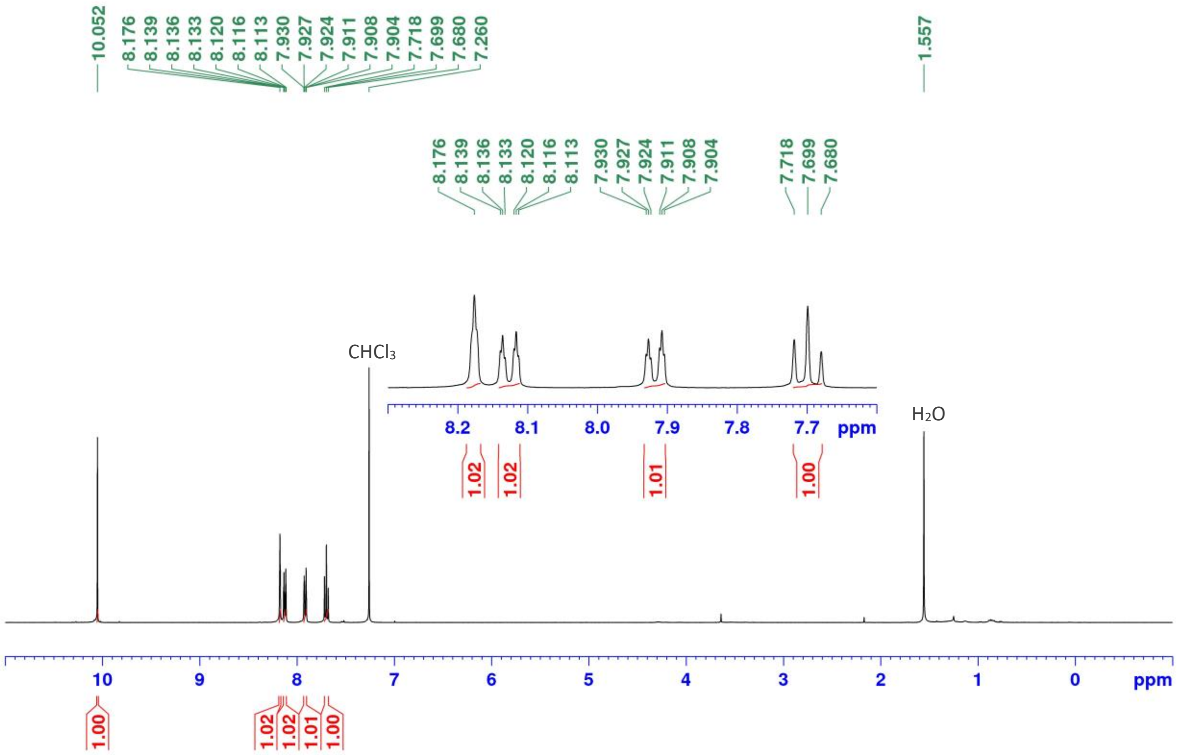

Figure S93. ^{1}H NMR (400 MHz) spectrum of **2o** in $CDCl_3$.

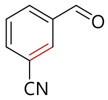

2o

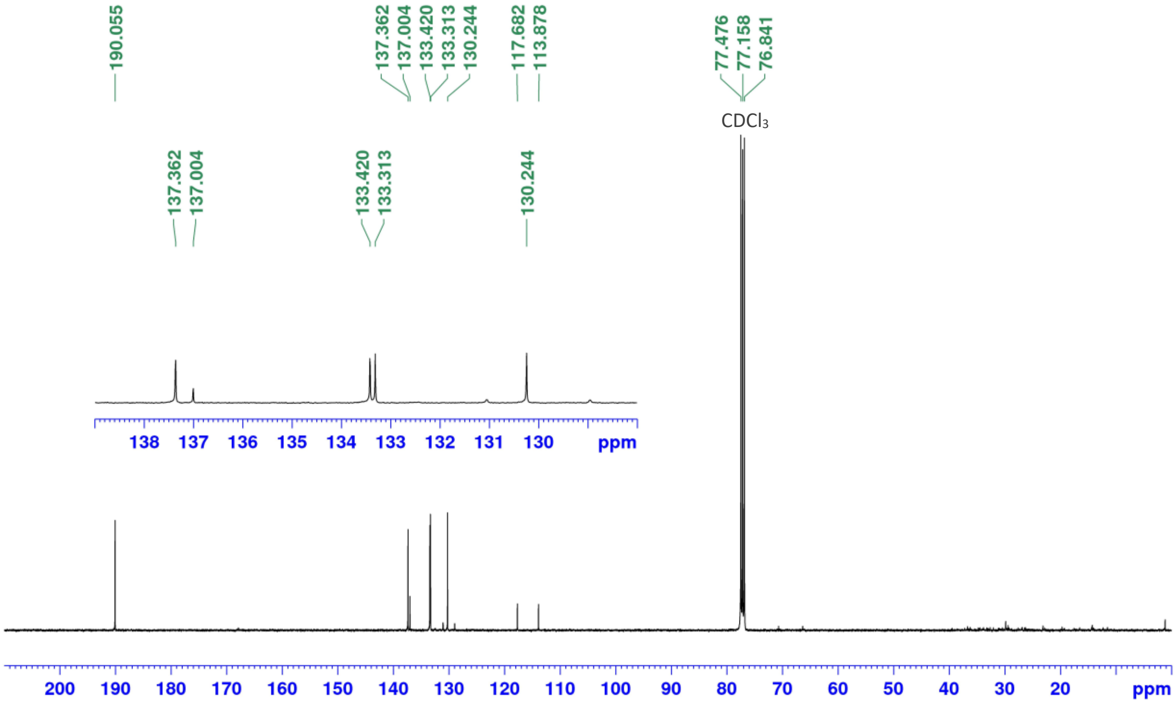

Figure S94. ^{13}C NMR (100 MHz) spectrum of **2o** in $CDCl_3$.

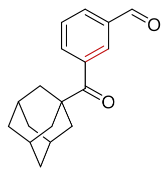

2p

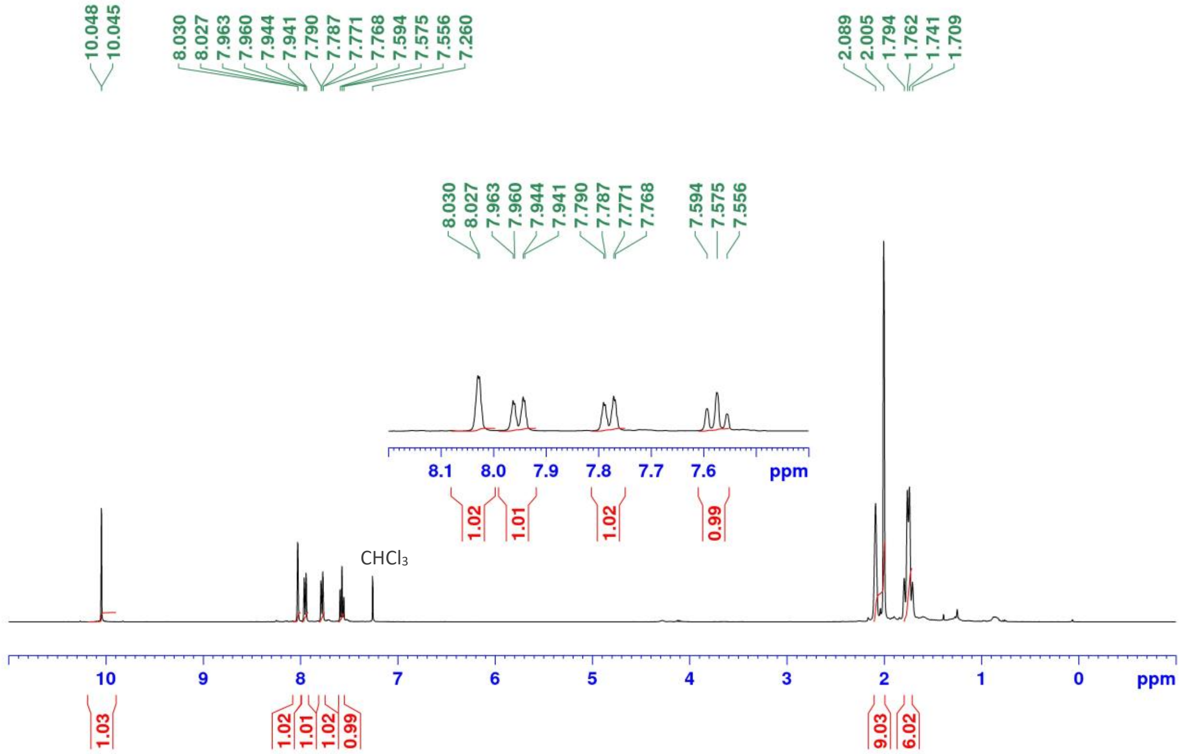

Figure S95. ^{1}H NMR (400 MHz) spectrum of **2p** in $CDCl_3$.

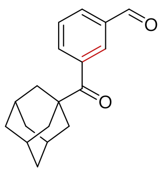

2p

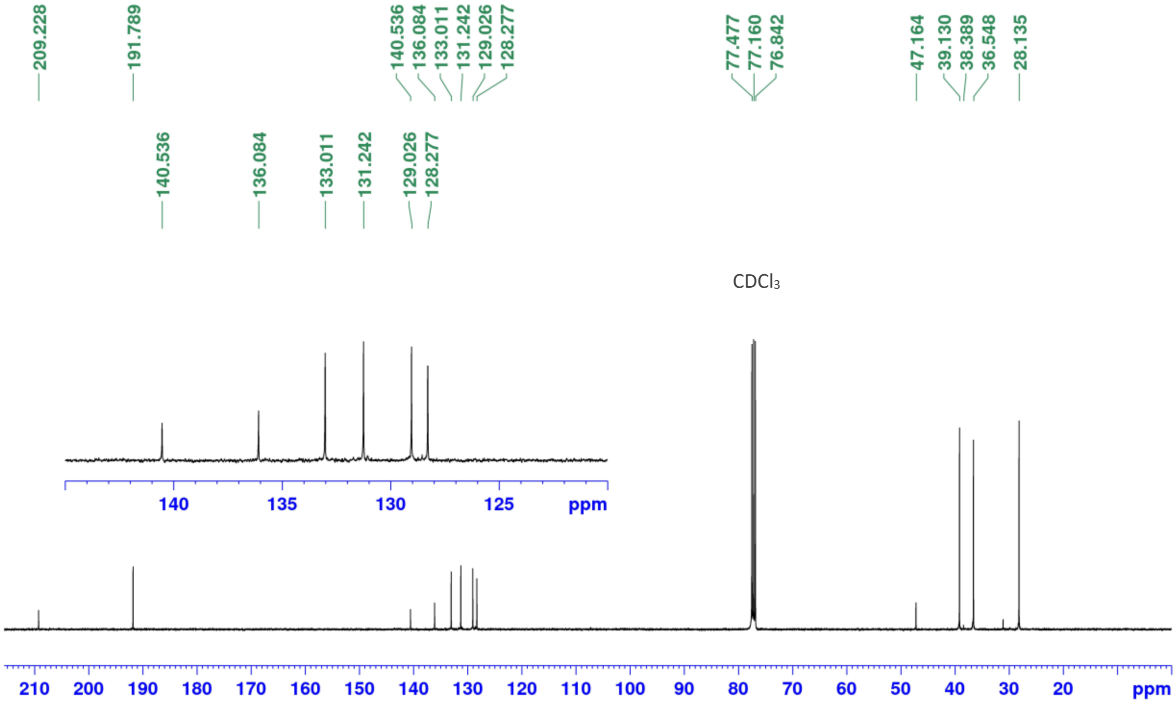

Figure S96. ^{13}C NMR (100 MHz) spectrum of **2p** in $CDCl_3$.

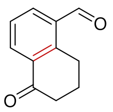

2q

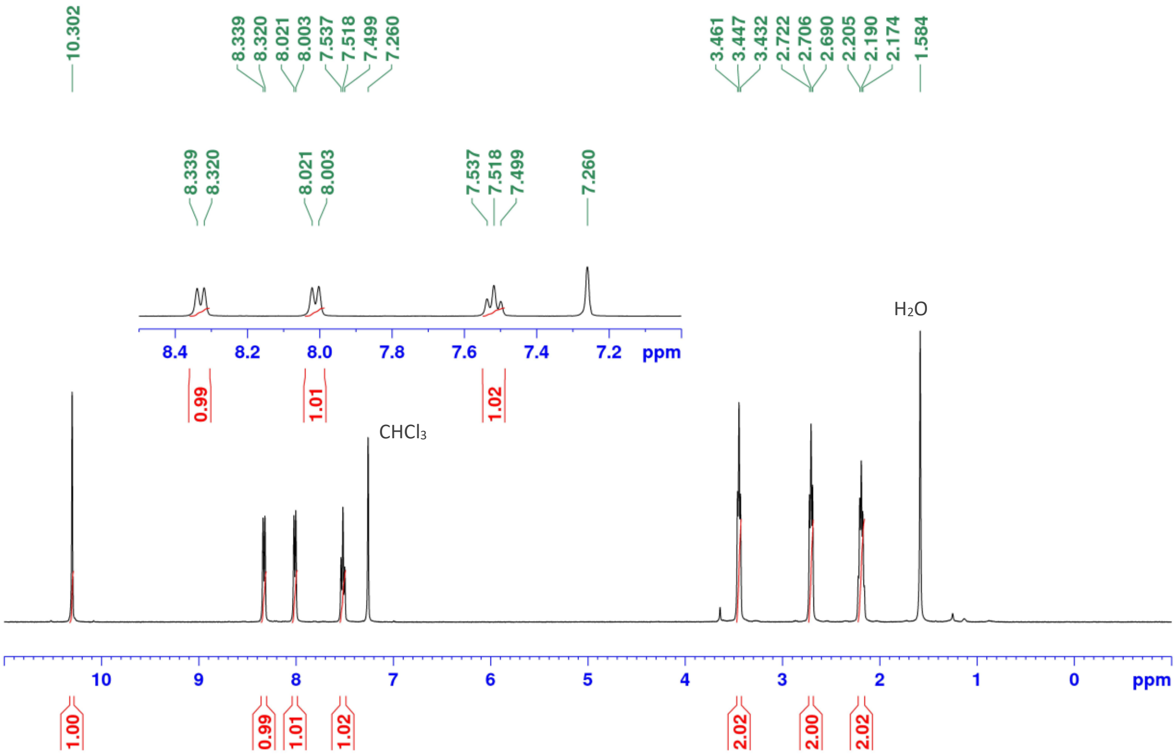

Figure S97. ^{1}H NMR (400 MHz) spectrum of **2q** in $CDCl_3$.

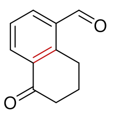

2q

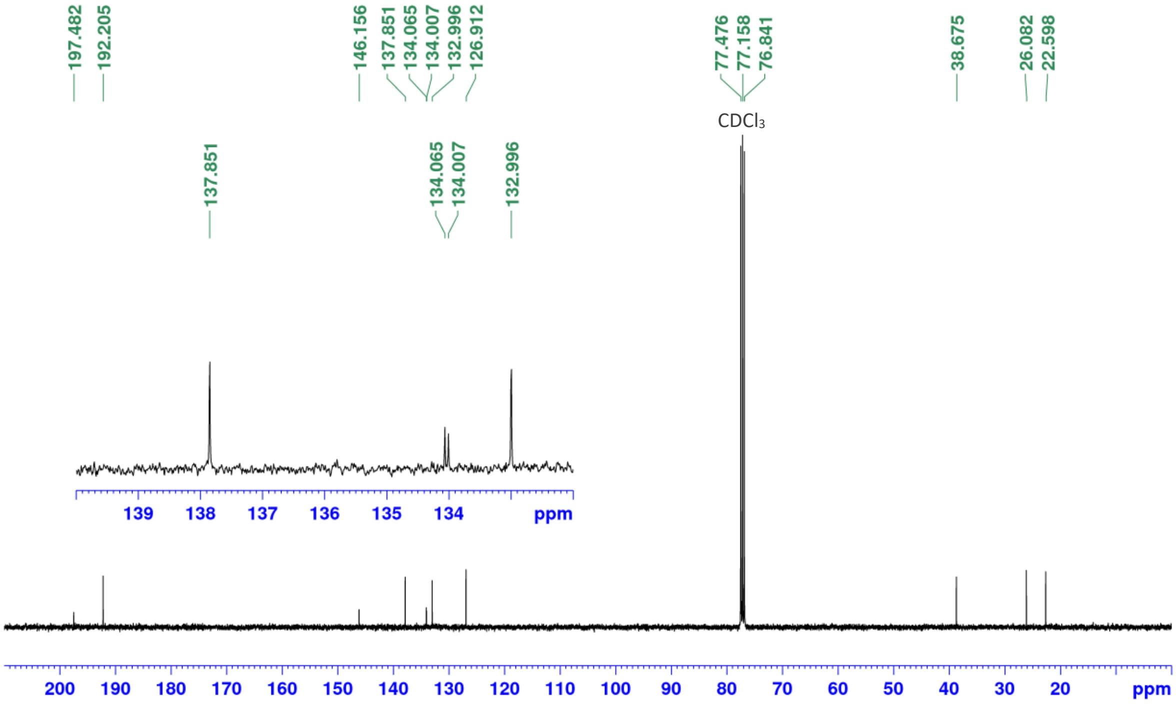

Figure S98. ^{13}C NMR (100 MHz) spectrum of **2q** in $CDCl_3$.

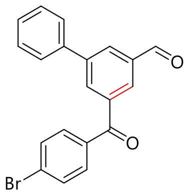

2r

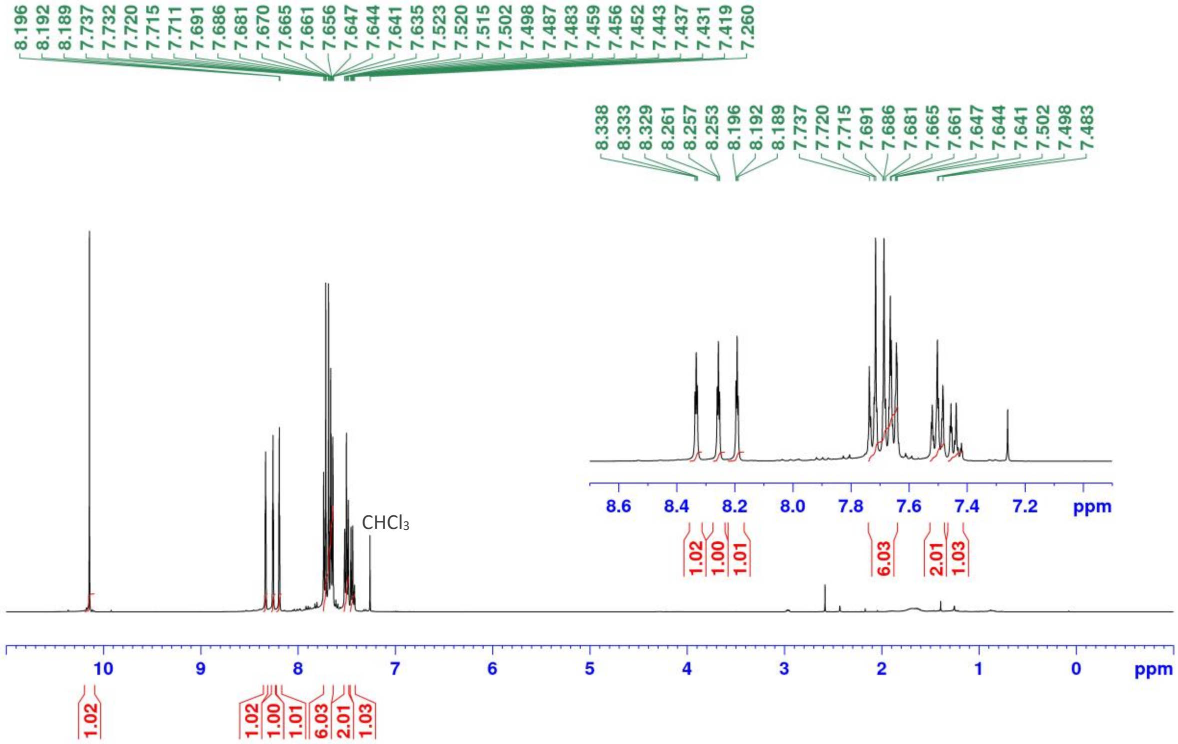

Figure S99. ^{1}H NMR (400 MHz) spectrum of **2r** in $CDCl_3$.

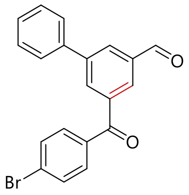

2r

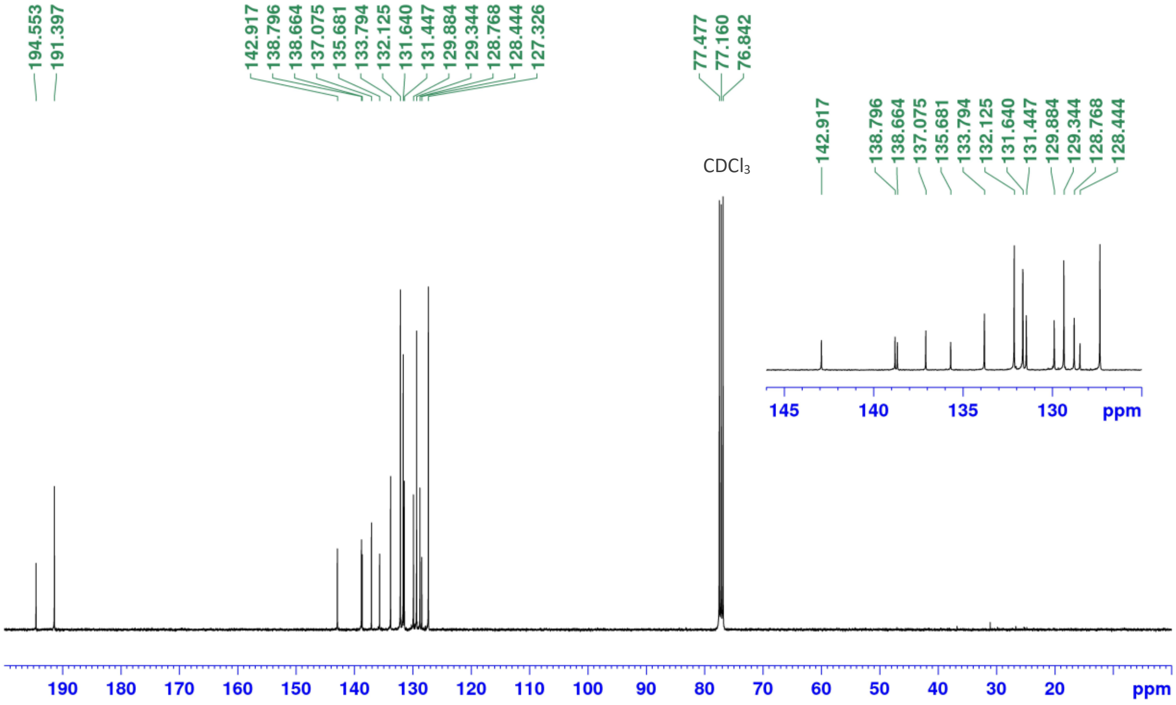

Figure S100. ^{13}C NMR (100 MHz) spectrum of **2r** in $CDCl_3$.

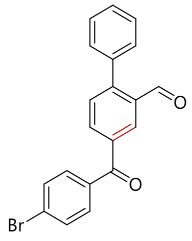

2s

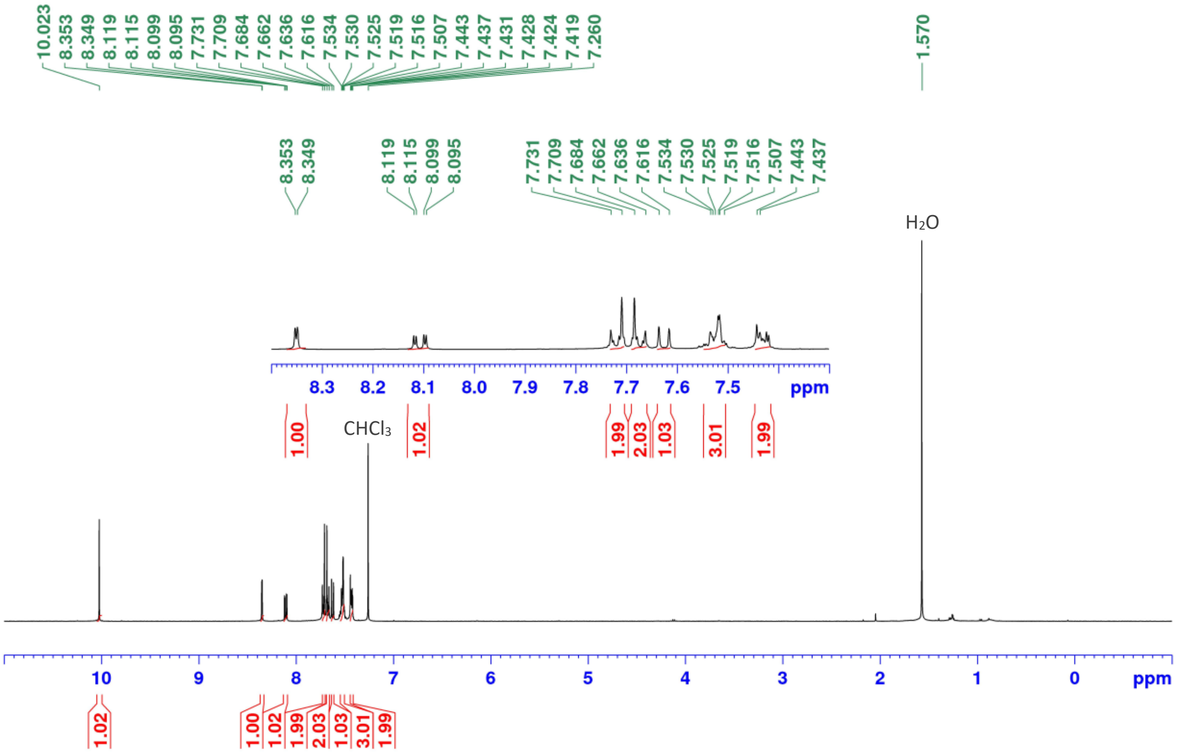

Figure S101. ^{1}H NMR (400 MHz) spectrum of **2s** in $CDCl_3$.

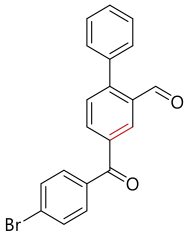

2s

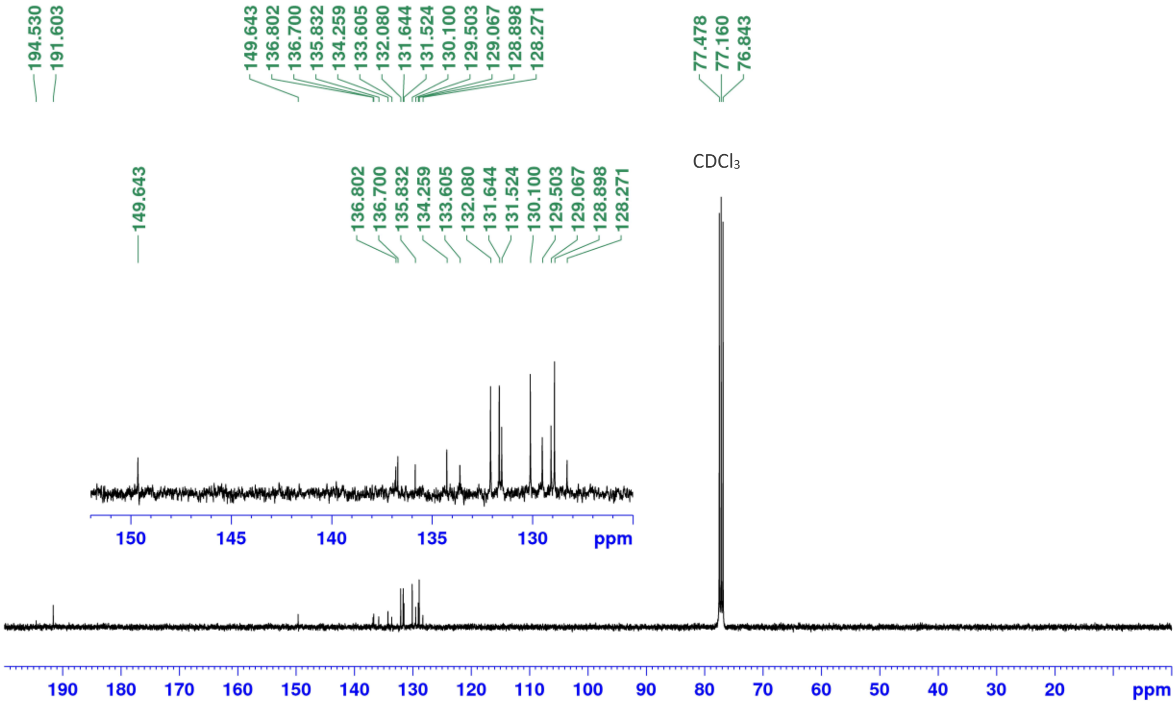

Figure 102. ^{13}C NMR (100 MHz) spectrum of **2s** in $CDCl_3$.

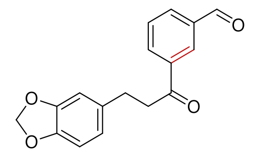

2t

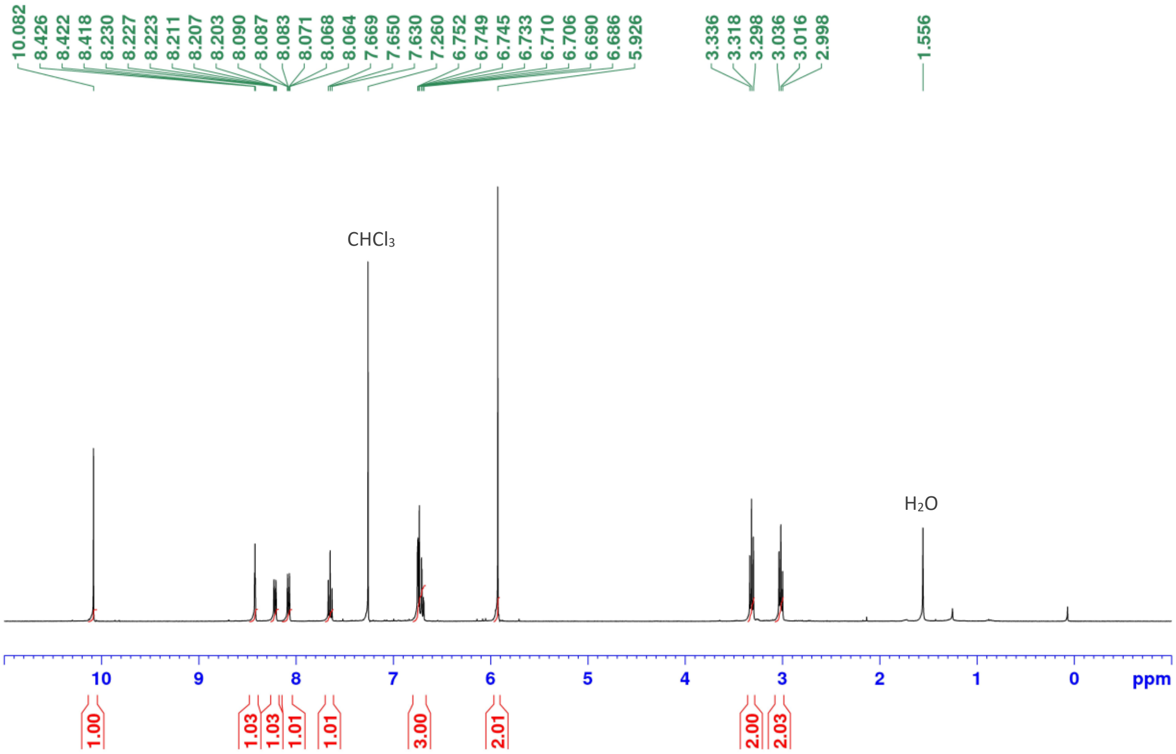

Figure S103. ^{1}H NMR (400 MHz) spectrum of **2t** in $CDCl_3$.

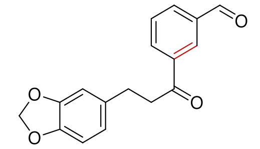

2t

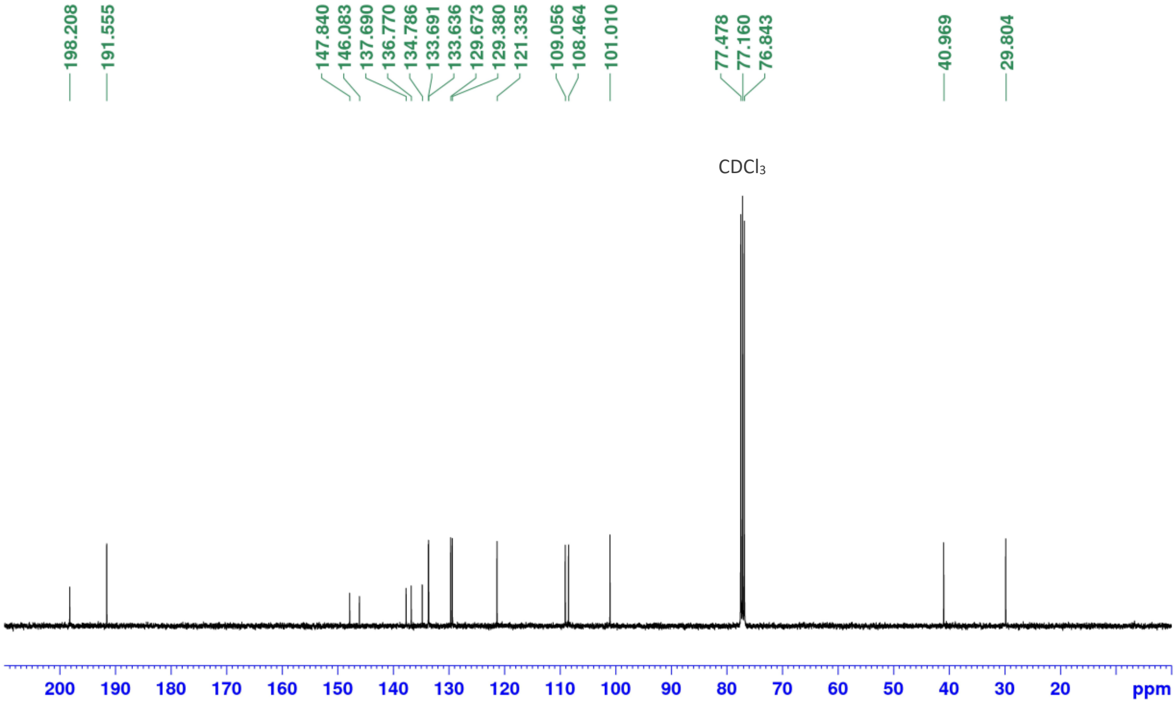

Figure S104. ^{13}C NMR (100 MHz) spectrum of **2t** in $CDCl_3$.

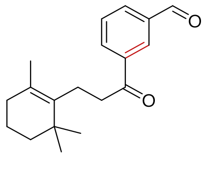

2u

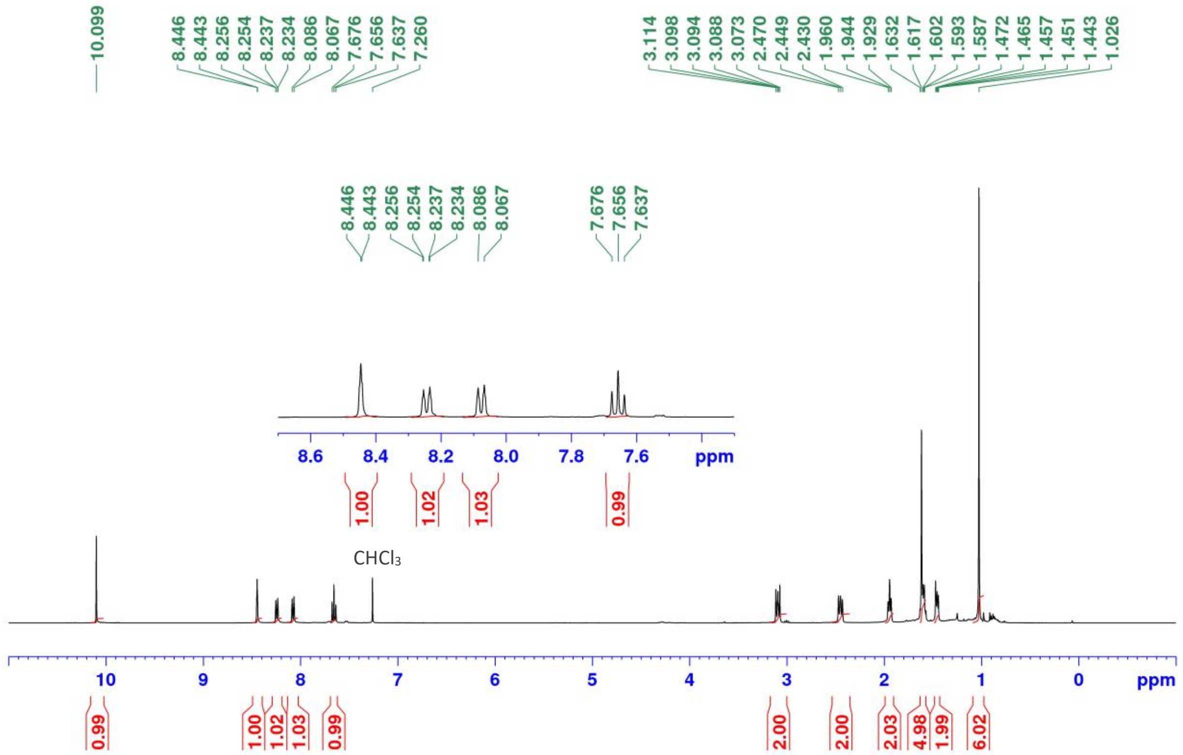

Figure S105. ^{1}H NMR (400 MHz) spectrum of **2u** in $CDCl_3$.

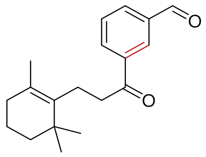

2u

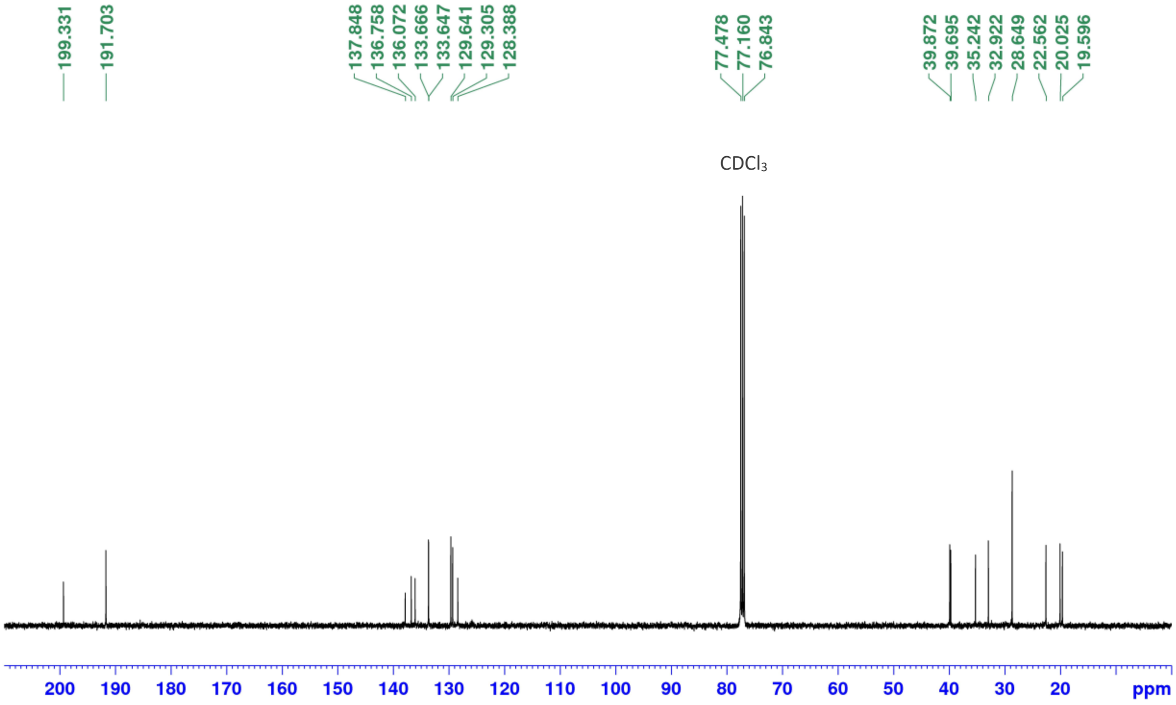

Figure S106. ^{13}C NMR (100 MHz) spectrum of **2u** in $CDCl_3$.

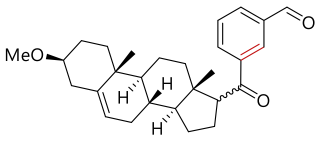

2v

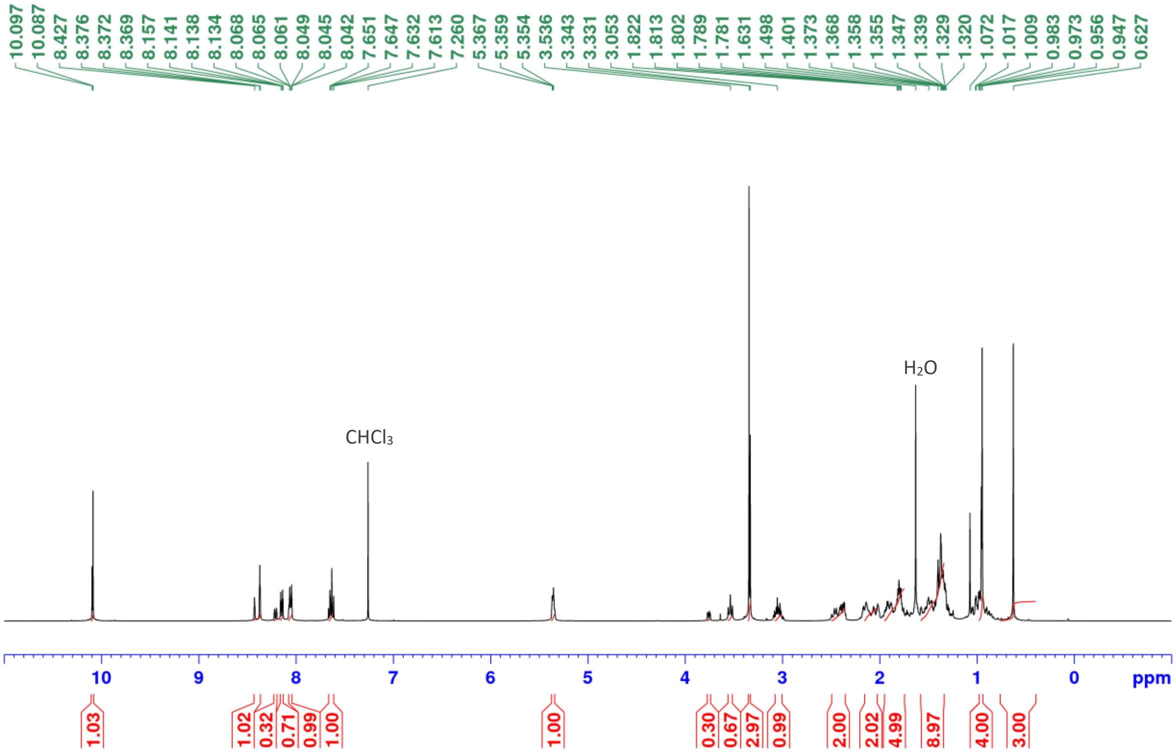

Figure S107. ^{1}H NMR (400 MHz) spectrum of **2v** in $CDCl_3$.

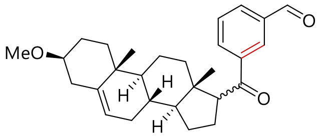

2v

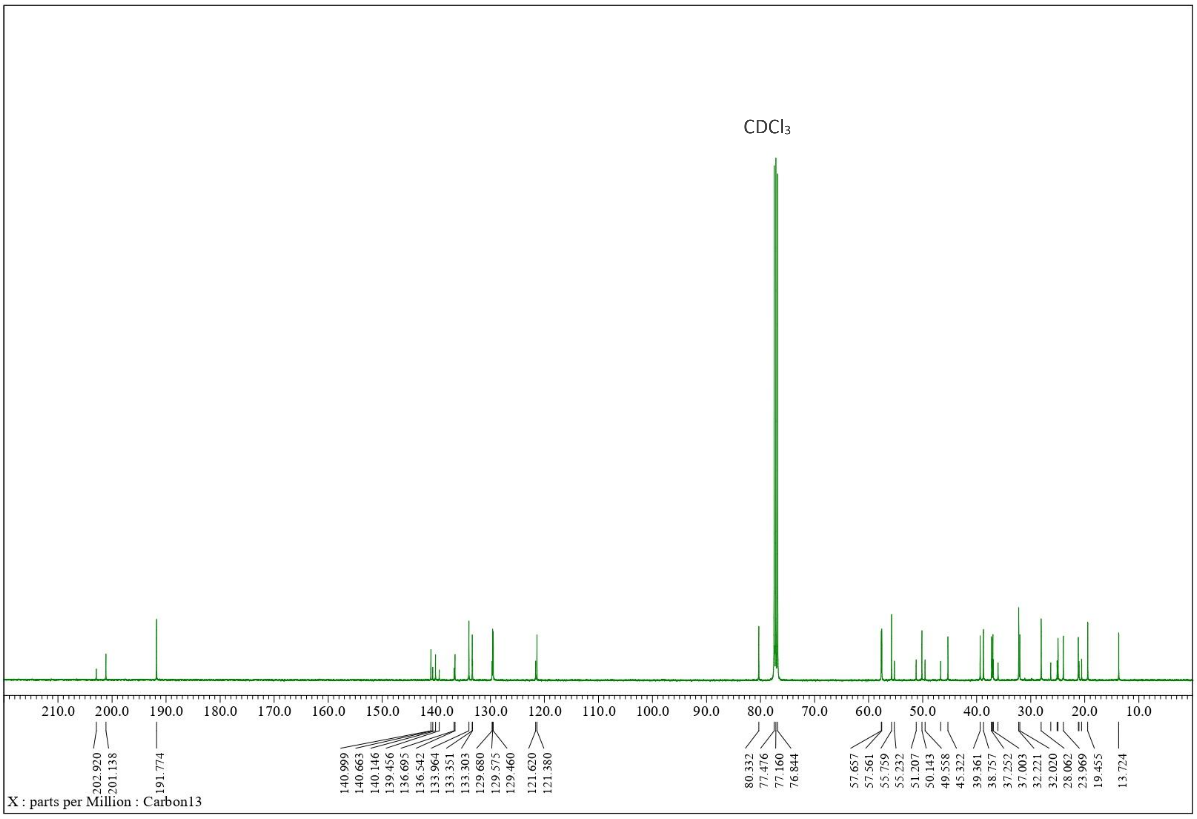

Figure S108. ^{13}C NMR (100 MHz) spectrum of **2v** in $CDCl_3$.

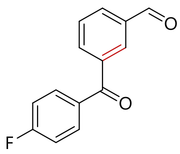

2w

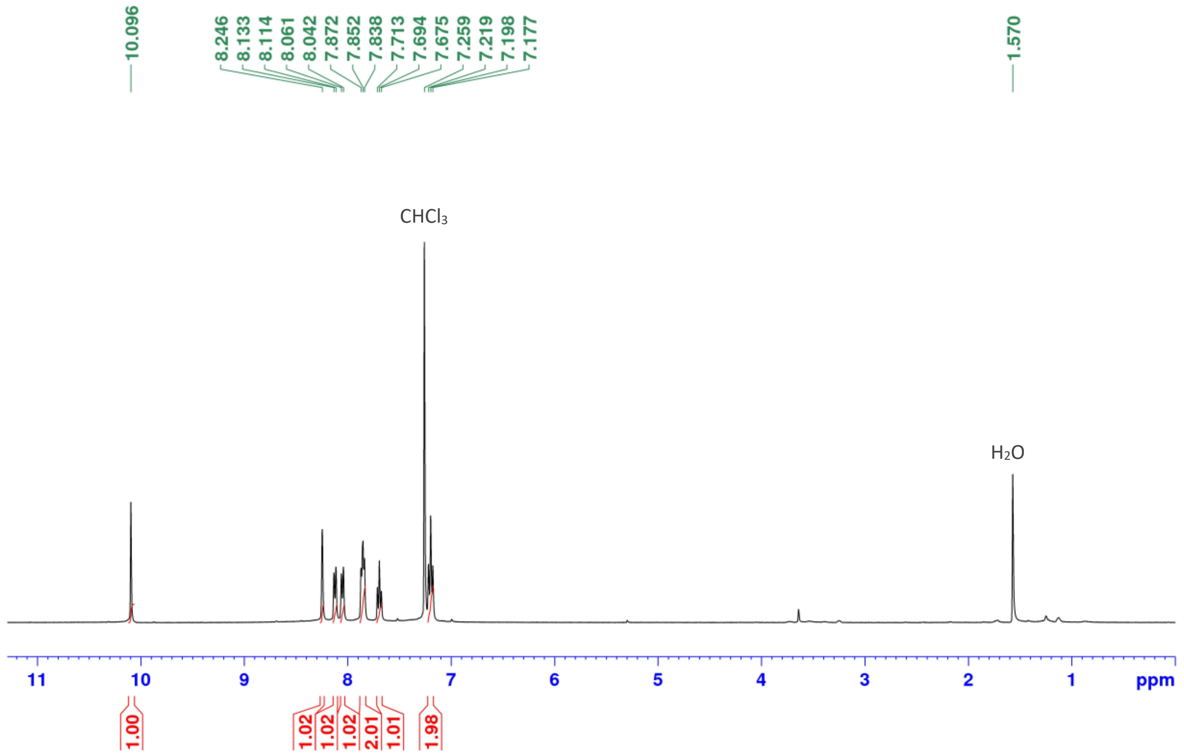

Figure S109. ^{1}H NMR (400 MHz) spectrum of **2w** in $CDCl_3$.

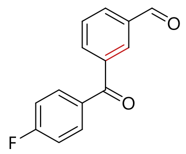

2w

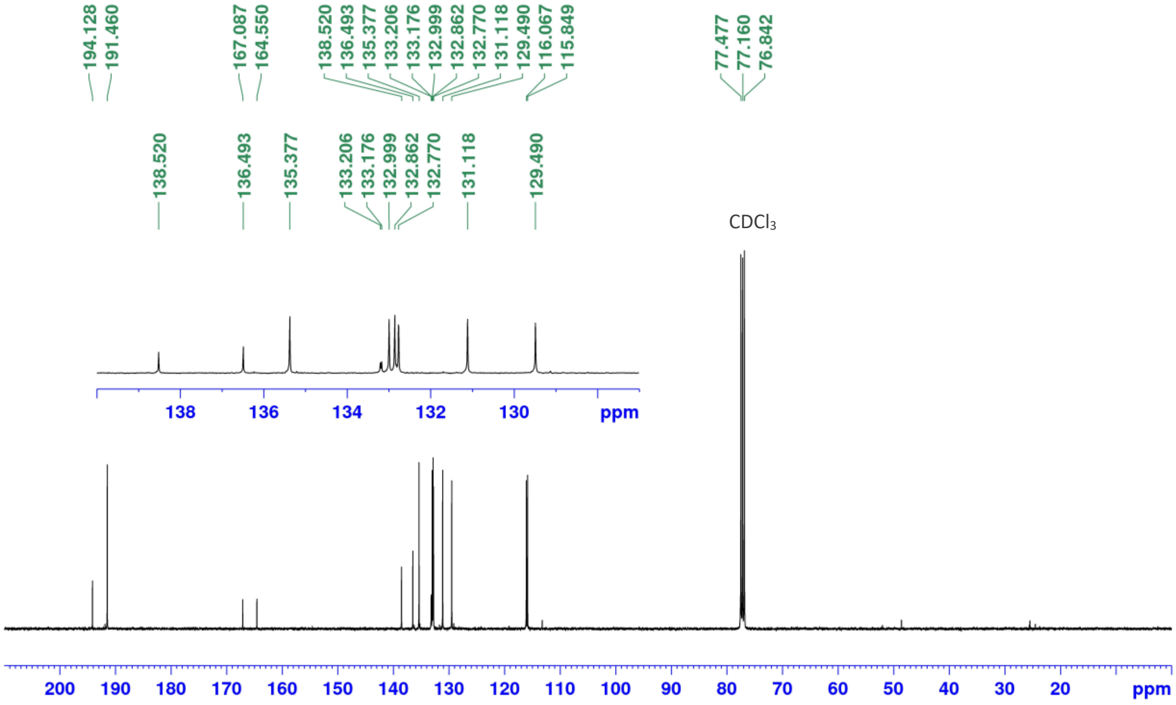

Figure S110. ^{13}C NMR (100 MHz) spectrum of **2w** in $CDCl_3$.

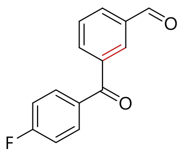

2w

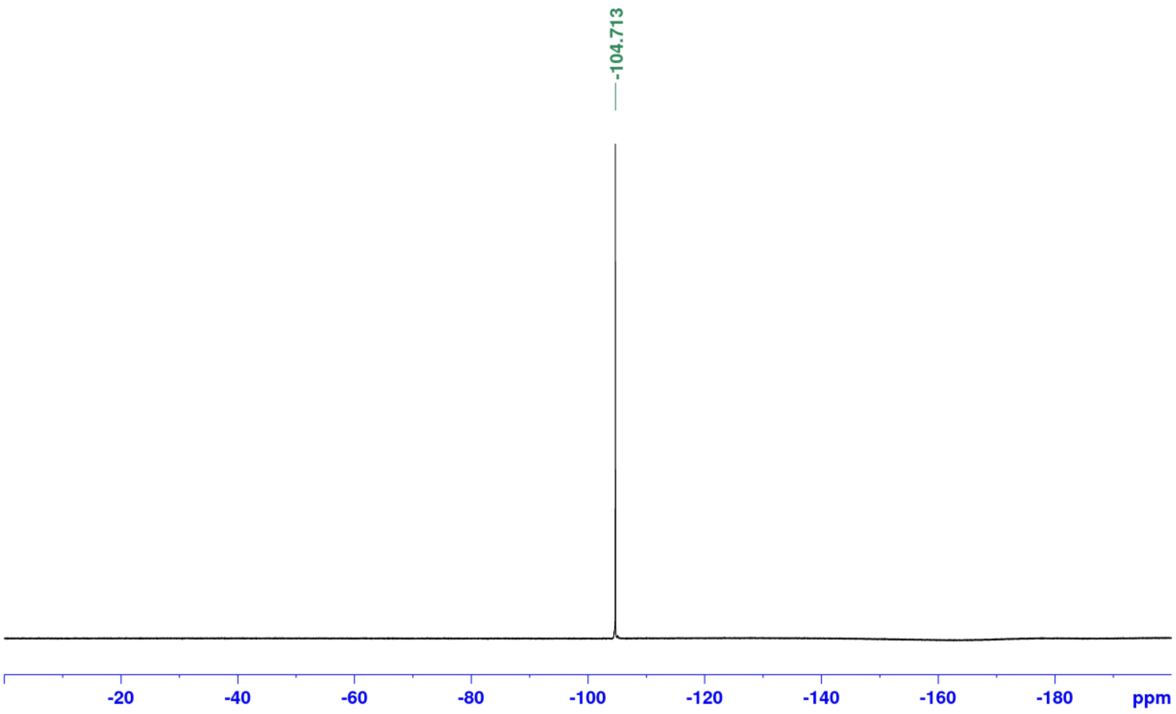

Figure S111. ^{19}F NMR (376 MHz) spectrum of **2w** in $CDCl_3$.

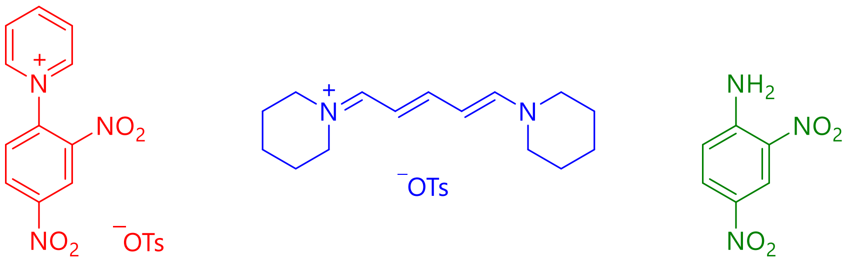

6 **7** 2,4-dinitroaniline

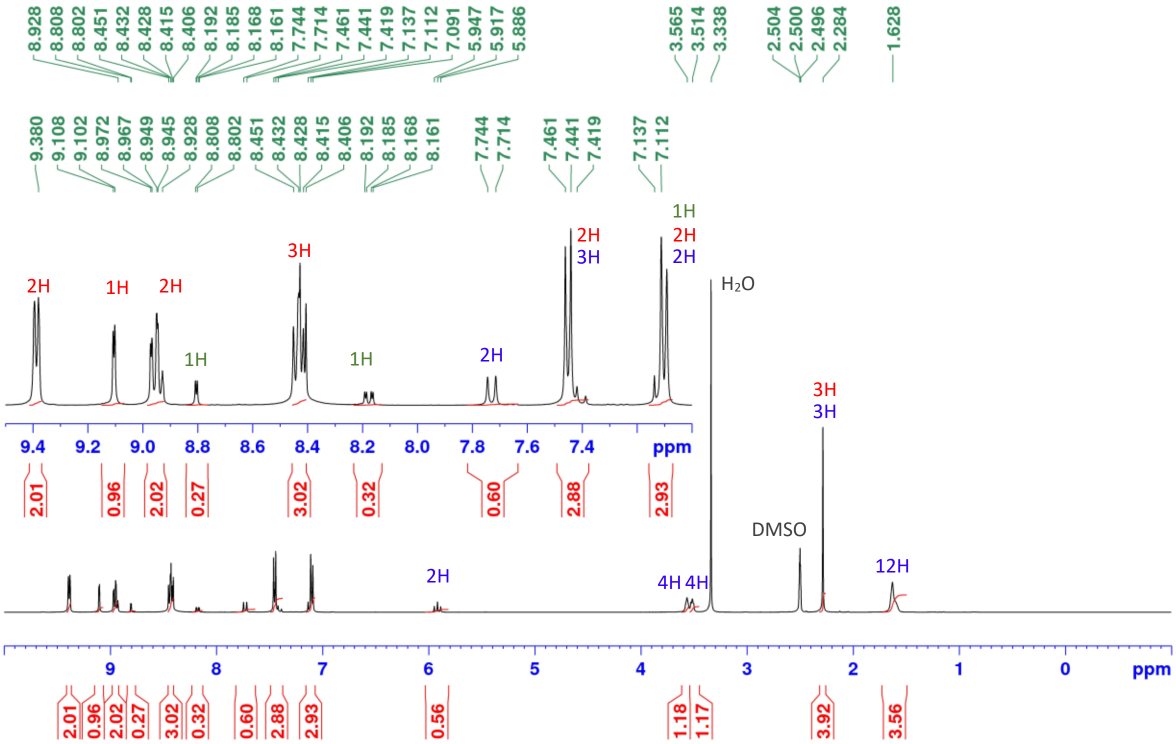

Figure S112. ^{1}H NMR (400 MHz) spectrum of the reaction mixture of *N*-arylpyridinium **6** with 0.5 equiv. of piperidine. DMSO-d_6 was used. The peaks with proton numbers in red were assigned to **6**. The peaks with proton numbers in blue were assigned to **7**. The peaks with proton numbers in green were assigned to 2,4-dinitroaniline.

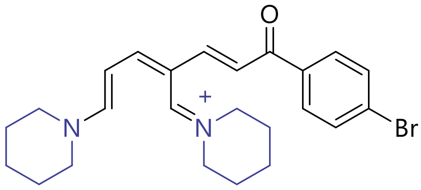

9

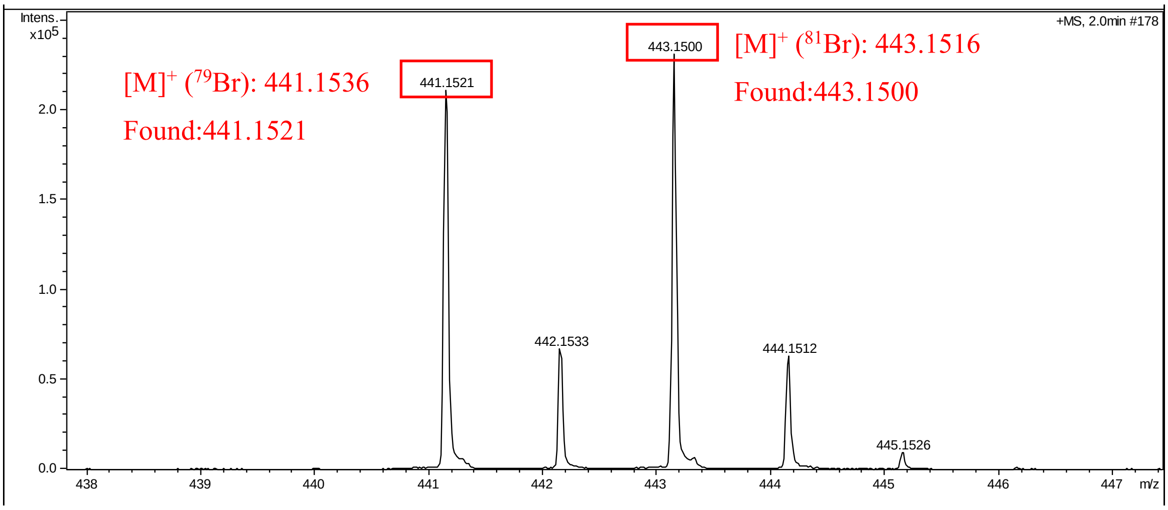

Figure S113. HRMS spectrum of **9**.

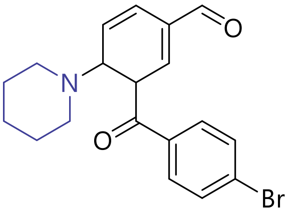

10

× unidentified peak

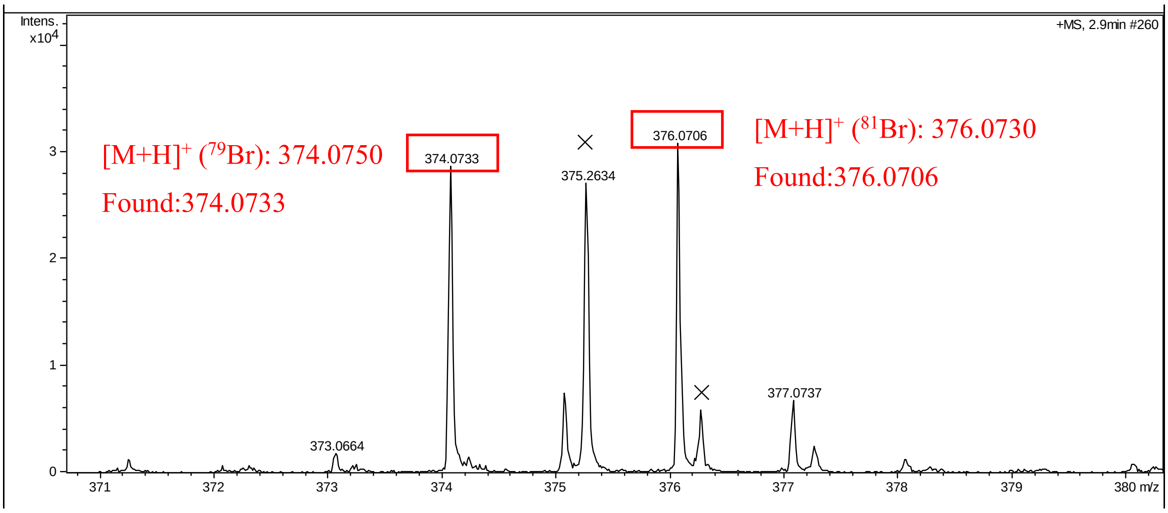

Figure S114. HRMS spectrum of **10**.
